# The Optimal Adjuvant Strategy of Aidi Injection With Gemcitabine and Cisplatin in Advanced Non–small Cell Lung Cancer: A Meta-analysis of 70 Randomized Controlled Trials

**DOI:** 10.3389/fphar.2021.582447

**Published:** 2021-05-28

**Authors:** Cheng-Qiong Wang, Xiao-Tian Zheng, Xiao-Fan Chen, Hong Jiang, Jun Huang, Yuan Jiang, Shan-Shan Hu, Xiao-Rong Huang, Shi-Yu Liu, Qi-Hai Gong, Ji-Hong Feng, Xue Xiao, Xiao-Fei Li, Zheng Xiao

**Affiliations:** ^1^ Department of General Practice, Affiliated Hospital of Zunyi Medical University, Zunyi, China; ^2^ Evidence-Based Medicine Center, MOE Virtual Research Center of Evidence-based Medicine at Zunyi Medical College, Affiliated Hospital of Zunyi Medical University, Zunyi, China; ^3^ Evidence-Based Medicine Research Centre, Jiangxi University of Traditional Chinese Medicine, Nanchang, China; ^4^ Department of Pharmacy, Affiliated Hospital of Zunyi Medical University, Zunyi, China; ^5^ School of Management, Zunyi Medical University, Zunyi, China; ^6^ GCP Center, Affiliated Hospital of Zunyi Medical University, Zunyi, China; ^7^ School of Pharmacy, Zunyi Medical University, Zunyi, China; ^8^ Department of Oncology, Lishui People’s Hospital, Sixth Affiliated Hospital of Wenzhou Medical University, Lishui, China; ^9^ Special Key Laboratory of Special Antitumor Drugs of Guizhou Province, Zunyi Medical University, Zunyi, China

**Keywords:** aidi injection, non-small cell lung cancer, gemcitabine and cisplatin, randomized controlled trial, optimal adjuvant strategy

## Abstract

**Introduction:** Aidi injection (Aidi) is composed of cantharidin, astragaloside, ginsenoside, and elentheroside E. As an important adjuvant therapy, Aidi in combination with gemcitabine and cisplatin (GP) is often used in the treatment of non-small cell lung cancer (NSCLC).

**Objectives:** We performed a new evaluation to demonstrate the clinical efficacy and safety of the Aidi and GP combination and further explored an optimal strategy for achieving an ideal response and safety level in advanced NSCLC.

**Methodology:** We collected all the related trials from Chinese and English-language databases, analyzed their methodological bias risk using the Cochrane evaluation Handbook for Systematic Reviews of Interventions Version 5.1.0, extracted all the data using a predefined data extraction form, pooled the data using a series of meta-analyses, and finally summarized the quality of evidence using the Grading of Recommendations Assessment, Development, and Evaluation (GRADE) approach.

**Results:** We included 70 trials with 5,509 patients. Compared with GP alone, the Aidi and GP combination showed a significant improvement in the objective response rate (ORR) [1.82 (1.62–2.04)], disease control rate (DCR) [2.29 (1.97–2.67)], and quality of life (QOL) [3.03 (2.55–3.60)] and a low incidence of hematotoxicity and gastrointestinal and hepatorenal toxicity. Aidi might be more suitable for patients who are first-treated, elderly, or patients with a Karnofsky Performance Status (KPS) score ≥ 60 or anticipated survival time (AST) ≥3 months. An Aidi (50 ml/day, 7–14 days/cycle for one to two cycles), gemcitabine (1000 mg/m^2^), and cisplatin (20–30 mg/m^2^, 40–50 mg/m^2^, or 60–80 mg/m^2^) might be an optimal regimen for realizing an ideal response and safety level. Most results were robust and of moderate quality.

**Conclusion:** Current evidence indicates that Aidi's value in adjuvant chemotherapy may be broad-spectrum, not just for some regimens. The Aidi and GP combination may show a good short-term response, antitumor immunity, and safety level in patients with NSCLC. Aidi (50 ml/day, 7–14 days/cycle for one and two cycles) with GEM (1000 mg/m^2^) and DDP (20–30 mg/m^2^ or 40–50 mg/m^2^) may be an optimal regimen for realizing an ideal goal in patients who are first-treatment, elderly, or have a KPS score ≥ 60 or AST≥3 months.

## Introduction

Lung cancer continues to be the most commonly diagnosed cancer and the leading cause of cancer death because of its poor prognosis (Chen et al., 2016; Torre et al., 2016; Siegel et al., 2017). Approximately 85% of lung cancers are non-small cell lung cancer. The combined use of cisplatin and gemcitabine is a standard regimen in the treatment of advanced NSCLC (Scagliotti et al., 2008; Scagliotti et al., 2012; Association et al., 2018). However, systemic chemotherapy often leads to multiple adverse drug reactions (ADRs), such as hematotoxicity, gastrointestinal toxicity, hepatorenal toxicity, and chemotherapy-induced immunosuppression (Pollera et al., 1987; Conroy et al., 2002; Waissbluth and Daniel, 2013; Shahid et al., 2018), which result in poor survival and quality of life.

In China, Chinese herb injections (CHIs) show important antitumor functions, upregulate antitumor immunity, and reduce chemotherapy-related ADRs in multiple malignant tumors (Cao et al., 2017; Duan et al., 2018a; Xiao et al., 2020a; Xiao et al., 2020b). As an important CHI, Aidi injection is composed of multiple active ingredients from Ginseng Radix Et Rhizoma, Astmgali Radix, Acanthopanacis Senticosi Radix Et Rhizoma Seu Caulis, and Mylabris (Supplementary Table S1; Xie et al., 2019). The active ingredients comprise the following main components: astragaloside (Re, Rb1, and Rg1), ginsenoside, cantharidin, elentheroside E, and syringin (Zhang et al., 2012; Zeng et al., 2016). These are purported to induce tumor cell apoptosis and to inhibit tumor cell proliferation and invasion (Duan et al., 2018b; Chen et al., 2019; Li et al., 2019), to reduce chemotherapy-related ADRs through anti-inflammation and antioxidative stress (Farag et al., 2019; Qu et al., 2019; Zhang et al., 2019), and to repair the host’s antitumor immunity though upregulating the levels of peripheral blood lymphocytes (PBLs) (Li et al., 2018; Zhou et al., 2018; Huang et al., 2019). In clinic, Aidi in combination with GP has been widely used in the treatment of NSCLC (Lv et al., 2018; Zhang, 2018; Zhou, 2018; Liu et al., 2019). According to the Cochrane systematic evaluation, three studies (Yang and Ding, 2012; Han et al., 2016; Xiao et al., 2017) including 36 trials evaluated the clinical efficacy and safety of Aidi injection with GP. However, there are many unacceptable methodological defects in previous systematic reviews (SRs) and meta-analyses. None of these evaluations ultimately demonstrate whether Aidi and GP combination shows a good clinical efficacy and safety levels. Moreover, no evaluation provides answers on the relationship between Aidi and GP, the optimal combination of Aidi and GP, optimal indication, treatment doses, or time and cycle. All these questions have become new obstacles to developing an optimal treatment strategy against advanced NSCLC and need to be confirmed by new evaluation.

Recently, new trials have been published (Geng et al., 2020; Guo, 2020; Tan et al., 2020; Xu, 2020; Xu and Li, 2020). Therefore, in accordance with the Preferred Reporting Items for Systematic Reviews and Meta-Analyses (PRISMA) guidelines, we performed a new evaluation to demonstrate the clinical efficacy and safety of the Aidi and GP combination and to explore further its therapeutic threshold and optimal strategy for achieving an ideal response and safety level in advanced NSCLC.

## Methods

### Inclusion Criteria

According to the PICOS guidelines, all included trials met the following criteria. Patients with inoperable NSCLC (stages III–IV) were diagnosed using histopathological and cytological diagnostic criteria and the tumor node metastasis (TNM) staging system (Mountain, 1989). None of the restrictions were set on the Karnofsky Performance Status score, anticipated survival time, treatment process (primary treatment, PT/retreatment, and RT), age of patients, usages of Aidi and GP, or follow-up. The experimental group received the Aidi and GP combination and the control group received the GP alone; one month before therapy onset, no patients received chemotherapy, radiotherapy, targeted therapy, or traditional Chinese medicine (TCM). We analyzed the clinical efficacy using tumor responses, survival, QOL, and antitumor immunity, and the ADRs using hematotoxicity, gastrointestinal toxicity, hepatorenal toxicity, neurotoxicity, alopecia, and oral mucositis. The study design was a randomized controlled trial (RCT).

### Exclusion Criteria

We excluded any study meeting the following criteria: duplicates; patients with non–NSCLC, non–Aidi, or Aidi alone; Aidi in combination with other chemotherapy, targeted therapy, radiotherapy, or other TCM; cohorts and case-control studies, and case series reports; meeting abstracts and reviews without available data; unrelated SRs/meta-analyses; and studies without data on tumor responses, survival, QOL, ADRs, or antitumor immunity.

### Literature Search

Based on the principle of patients (P) and intervention (I), two reviewers (Cheng–Qiong Wang and Xiao-Tian Zheng) used standard medical subject headings and free-text words to build the search strategies and searched all records independently. The terms were “Lung Neoplasms” [Mesh], Pulmonary Neoplasms, Lung Neoplasm, Pulmonary Neoplasm, Lung Cancer, Lung Cancers, Pulmonary Cancer, Pulmonary Cancers, Lung carcinoma, Pulmonary carcinoma, NSCLC, Aidi, Aidi injection, Addie, and Compound Cantharis Injection. A systematic search of the literature published until November 2020 was conducted using the following databases: PubMed, Embase, Science Citation Index, the Cochrane Central Register of Controlled Trials (CENTRAL) database, China National Knowledge Infrastructure (CNKI) database, Chinese Scientific Journals Full-Text database (VIP), Wanfang database, and China Biological Medicine (CBM) database. In addition, the two reviewers read all the related SRs/meta-analyses about Aidi and GP combinations for NSCLC and collected eligible trials from their references.

### Study Selection

Two independent reviewers (Shan-Shan Hu and Hong Jiang) selected eligible trials using predefined inclusion and exclusion criteria. Disputes of selections were resolved by discussion with each other or a third reviewer (Zheng Xiao).

### Methodological Bias Risk

Two independent reviewers (Cheng–Qiong Wang and Xiao–Tian Zheng) critically assessed the methodological bias risk of all included trials using the Cochrane evaluation Handbook for SRs of Interventions version 5.1.0 (Higgins, 2011 JPT). The bias risk was appraised according to the following features: random sequence generation (selection bias), allocation concealment (selection bias), blinding of patients and researchers (performance bias), blinding of indicator measurement (detection bias), incomplete outcome data (attrition bias), selective reporting (reporting bias), and other biases, (e.g., whether the baseline was comparable). Each item was categorized into one of three levels—a low risk of bias, a high risk, or an unclear risk. If any domain was considered high risk, the trial was defined as poor quality. Disputes of assessments were resolved by discussion with each other or a third reviewer (Zheng Xiao).

### Indicator Definition

We analyzed the clinical efficacy using tumor responses, survival, QOL, and levels of PBLs. In accordance with World Health Organization (WHO) criteria for solid tumor responses (Miller et al., 1981) or Response Evaluation Criteria in Solid Tumors (RECIST) guidelines (Watanabe et al., 2003), the indicators used were complete response (CR), partial response (PR), no change (NC), and progressive disease (PD). We analyzed the tumor response using the objective response rate (ORR, ORR = CR + PR) and disease control rate (DCR, DCR = CR + PR + NC). We analyzed the survival using overall survival (OS), progression-free survival (PFS), OS, and PFS rates. In accordance with the Karnofsky Performance Status (KPS) Scale (Yates et al., 1980; Clancey, 1995), the scores increased by ≥ 10 points after treatment, and the QOL demonstrated an improvement. We analyzed antitumor immunity using the levels of CD3^+^ T cells, CD3^+^ CD4^+^ T cells, and CD3^+^ CD8^+^ T cells, and CD4^+^/CD8^+^ T cell ratios and natural killer cell (NK cell) activity, which were measured by using flow cytometry (FCM) or indirect immunofluorescence tests before and after treatment.

In accordance with WHO (Miller et al., 1981) or National *Cancer* Institute-Common Terminology Criteria for Adverse Events (NCI-CTCAE) (Trotti et al., 2003), we analyzed ADRs using hematotoxicity, gastrointestinal toxicity, liver or renal toxicity, neurotoxicity, alopecia, and oral mucositis. Hematotoxicity included myelosuppression, neutropenia (granulocytes <2×10^9^/L), thrombocytopenia (platelets <100×10^9^/L), and anemia (hemoglobin <110 g/L). Liver toxicity was detected using a level of serum aminotransferase or alkaline phosphatase >1.25 × N, and renal toxicity was detected using a level of serum urea nitrogen or creatinine >1.25 × N.

### Data Extraction

Using a predefined data extraction form, two independent reviewers (Yuan Jiang and Xiao–Rong Huang) extracted the title, author, year, study design, and nationality; the KPS score, AST, PT/RT, and age of patients; the sample size; the usage of Aidi and GP combination; the measurement method of tumor response, ADRs, and PBLs; follow-up; and tumor response (ORR and DCR), OS, PFS, OS rate, PFS rate, QOL, ADRs, and PBLs. If articles provided the details, we directly extracted the data. Otherwise, we directly requested information from the author via email. If no author replied, we reconstructed the graphed data into analyzable data using a software graph digitizer scout (Guyot et al., 2012).

### Statistical Analysis

We analyzed the ORR, DCR, OS, PFS, OS rate, PFS rate, QOL, and ADRs using odds ratios (ORs) and their 95% confidence intervals (CIs), the OS and PFS using hazard ratios (HRs) and their 95% Cis, and the levels of PBLs using standardized mean differences (SMDs) and their 95% CIs. If *p* < 0.05, the results were considered significant. We analyzed the potential statistical heterogeneity using Cochran’s χ^2^ test and I^2^ statistic, and I^2^ > 50% indicated statistical heterogeneity. Two independent reviewers (Cheng–Qiong Wang and Jun Huang) performed a series of meta-analyses using Review Manager 5.3 (as recommended by Cochrane Collaboration). If *p* > 0.1 and I^2^ ≤ 50%, we pooled the OR, HR, SMD, and their 95% CIs using a fixed-effects model (FEM); if I^2^ > 50% and without significant clinical heterogeneity, we pooled the data using a random-effects model (REM), and with significant clinical heterogeneity, we abandoned the pooling of data and described the data.

If the trials were greater than 10, we analyzed the potential publication bias using a funnel plot and Egger/Begg’s tests. Trials with poor quality, overestimated efficacy, and underestimated ADRs showed a negative influence on the outcome robustness. If the result was significantly different and beneficial to Aidi use, we defined it as an under- or overestimated trial. Then, we summarized the OR, HR, SMD, and their 95% CIs under extreme conditions, which rejected all poor trials, and trials with overestimated efficacy or underestimated ADRs (Xiao et al., 2018b; Xiao et al., 2019b). If the result before and after rejection had good consistency the result was robust; if not, the result was poorly robust.

According to variables such as KPS score, AST, treatment process (PT/RT), age of patients, and usage of Aidi and GP, we developed a subgroup analysis model to analyze the clinical heterogeneity and to reveal the effects of variables between trials on tumor response, ADRs, and PBL levels (Sun et al., 2010; Xiao et al., 2020a; Xiao et al., 2020b). In addition, we implemented a univariate random effects meta-regression to analyze the relationship between each variable and tumor response/ADRs and a post hoc multiple regression analysis to adjust for the OR of tumor response and ADRs of the variables.

### Evidence Quality

According to the Grading of Recommendations Assessment, Development, and Evaluation guideline (Guyatt et al., 2008), two independent reviewers (Xiao-Fan Chen and Cheng–Qiong Wang) summarized the quality of evidence using the following five criteria (i) limitations in trial design (if most trials had unclear risk and no high risk, or if some trials had high risk and the result of sensitivity analysis was robust, we downgraded the quality by one level; if some trials had high risk and the result of sensitivity analysis was poorly robust, or all trials had high risk, we downgraded the quality by two levels. If neither of these applied, we downgraded them by one level) (ii) inconsistency (with heterogeneity, and the result was poorly robust) (iii) indirectness (the patients, interventions, outcomes, or controls did not meet the themes) (iv) imprecision (the sample size for outcome <300 cases); and (v) reporting bias (with reporting bias, and the result was poorly robust). Because of the (ii)–(v) domains, we downgraded the quality by one level. Finally, we summarized the quality into four grades: high, moderate, low, and very low.

## Results

### Search Results

**FIGURE 1:**
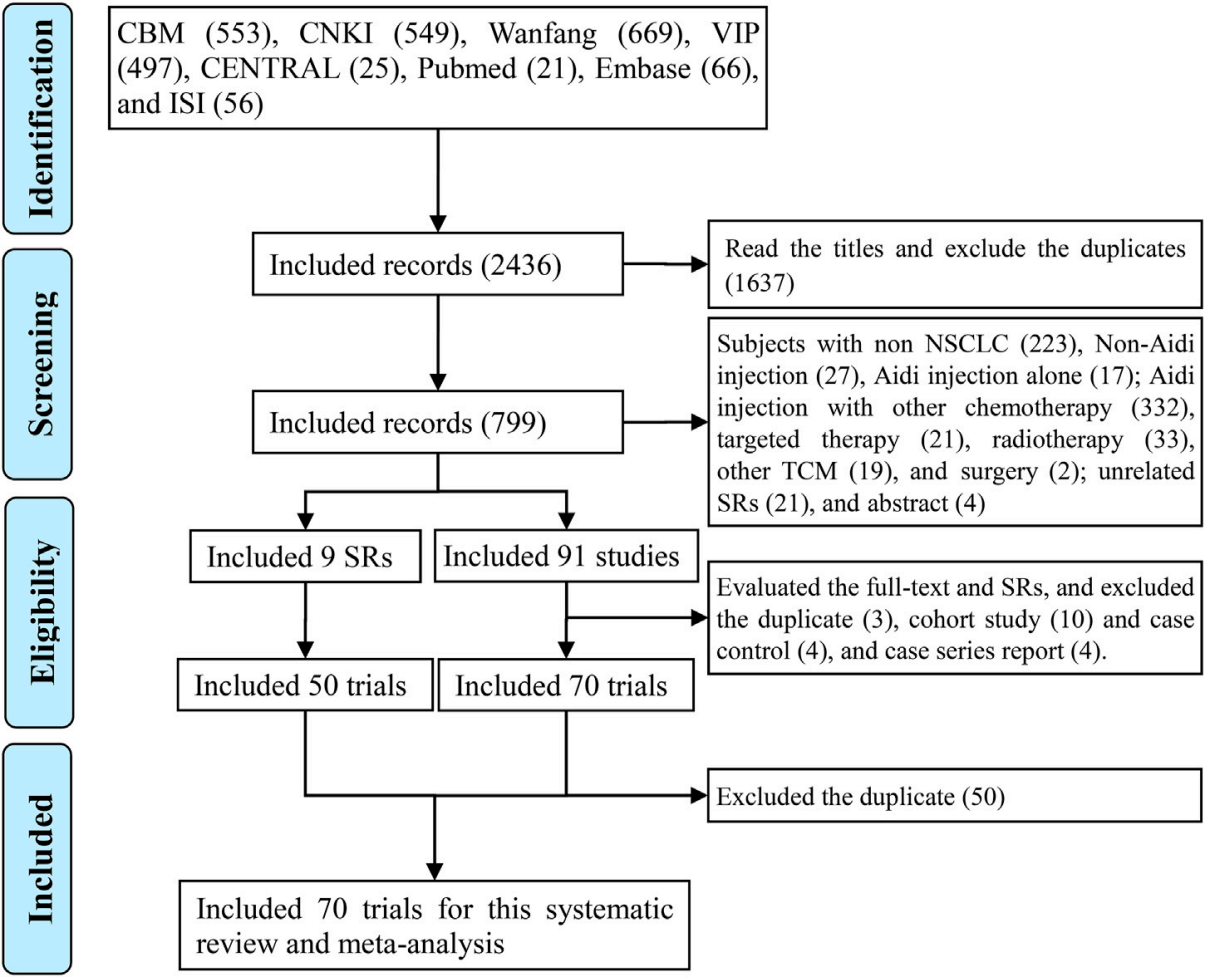
PRISMA 2009 flow diagram for the eligible trials We identified 70 eligible trials for this analysis.

We collected 2,436 records by searching. After scanning the titles, we collected 799 records. After scanning the abstracts and excluding the studies, such as those with non-NSCLC, non-Aidi injection, Aidi injection alone, and Aidi injection with other chemotherapy, we collected 91 original studies, three SRs/meta-analyses, and six related-SRs/meta-analyses. After evaluating original studies and excluding duplicates, cohort and case control studies, and case series reports, we included 70 eligible trials. In addition, we collected 36 eligible trials from the three SRs/meta-analyses (Yang and Ding, 2012; Han et al., 2016; Xiao et al., 2017) and 42 trials from the six related SRs/meta-analyses (Ma et al., 2009; Tian et al., 2014; Xiao et al., 2016; Wu et al., 2017a; Xiao et al., 2018b; Wang et al., 2018). Finally, we identified 70 eligible trials for this meta-analysis (Supplementary Tables S2–S5; Figure 1).

### Basic Features of the Included Trials

In this meta-analysis, we identified 70 trials from China, which involved 5,509 NSCLC patients including 3,278 males and 1,995 females with ages ranging from 21–86 years old (Table 1). The intervention was Aidi injection which was intravenously injected with 30–100 ml/day, 7–28 days per cycle for one to four cycles. The experimental group with 2,783 cases received Aidi and GP combination, and the control with 2,726 cases received GP alone. GEM (1000 mg/m^2^) was used in combinations with DDP (20–100 mg/m^2^). The efficacy and safety were evaluated after follow-up of six weeks to two years. Sixty-three trials with 4,851 patients reported the tumor response rate (ORR and DCR) according to WHO (Miller et al., 1981) or RECIST guidelines (Watanabe et al., 2003); four trials with 320 patients reported the survival (Sun et al., 2008; Cheng et al., 2014; Li and Yang, 2014; Guo, 2020) and no trials reported the PFS0 .31 trials with 2,485 patients reported the QOL; 18 trials with 1,688 patients reported the antitumor immunity before and after therapy which were detected using a FCM (Feng et al., 2008; Jiang et al., 2011; Xu et al., 2013; Zhang, 2014; Han et al., 2015; Li, 2015; Zhao et al., 2015; Li et al., 2016; Wu et al., 2017b; Huang et al., 2017; Ma, 2017; Su, 2017; Lv et al., 2018; Liu et al., 2019; Zhao and Li, 2019; Guo, 2020; Tan et al., 2020; Xu, 2020). 58 trials with 4,596 patients reported ADRs according to WHO (Miller et al., 1981) or Common Terminology Criteria for Adverse Events (CTCAE) (Trotti et al., 2003).

**TABLE 1:** Basic features of the included trials.

First author. Year	Non-small cell lung cancer (NSCLC)							Interventions		Fellow up	Criteria	Outcomes
	TNM	KPS	AST	TP	E/C	M/F	Age	Aidi injection (usages)	GP (dosages)			
Zou et al. (2006)	IIIb–IV	≥60	>3 m	Un	42/39	56/25	35–73	80 ml, 14 days, 1cycle	G:1 g/m^2^; P:30 mg/m^2^	6–12 w	WHO,WHO	O1,2,4
Feng et al. (2008)	III–IV	≥70	Un	PT	68/62	88/42	38–74	50 ml, 15 days, 2cycles	G:1 g/m^2^; P:75 mg/m^2^	6 w	WHO,WHO	O1,2,4,5
Sun et al. (2008)	IIIb–IV	≥60	>3 m	PT/RT	33/30	54/9	34–73	100 ml,14 days,2cycles	G:1 g/m^2^; P:30 mg/m^2^	1 year	WHO, Un	O1,3
Yang et al. (2008)	III–IV	≥60	≥3 m	Un	30/27	39/18	34–82	80 ml,8 days,2cycles	G:1 g/m^2^; P:75 mg/m^2^	6 w	WHO, No	O1,2
Zhao et al. (2008)	III–IV	Un	>3 m	Un	30/20	31/19	29–73	30 ml,21 days,3cycles	G:1 g/m^2^;P:60–80 mg/m^2^	9 w	WHO,WHO	O1,2,4
Lv et al. (2009)	IIIb–IV	≥60	≥3 m	Un	30/30	42/18	45–70	80 ml,10 days,2cycles	G:1 g/m^2^; P:60–80 mg/m^2^	6 w	WHO,WHO	O1,2,4
Song et al. (2009)	III–IV	>60	>3 m	PT	30/30	36/24	53–76	50 ml,14 days,2cycles	G:1 g/m^2^; P:30 mg/m^2^	6 w	WHO,WHO	O1,2,4
Wang. (2009)	IIIa–IV	≥60	>3 m	Un	32/27	48/11	Un	Un,10 days,2cycles	G:1 g/m^2^; P:20 mg/m^2^	6 w	WHO,WHO	O1,O4
Wen et al. (2009)	IIIa–IV	Un	>3 m	PT/RT	38/38	52/24	32–77	50 ml,8–10 days,2cycles	G:1 g/m^2^; P:75 mg/m^2^	6 w	WHO,WHO	O1,2,4
Zhang. (2009)	IIIb–IV	≥60	>3 m	PT/RT	32/31	44/19	31–79	80 ml,14 days,2cycles	G:1 g/m^2^; P:80 mg/m^2^	6 w	WHO,WHO	O1,2,4
Hong et al. (2010)	IIIb–IV	≥60	≥3 m	PT	90/70	82/78	38–70	60 ml,14 days,2cycles	G:1 g/m^2^; P:25 mg/m^2^	6 w	WHO,WHO	O1,2,4
Hou and Zhang. (2010)	III–IV	≥60	≥3 m	Un	40/38	49/29	32–79	50 ml,14 days,2cycles	G:1 g/m^2^; P:25 mg/m^2^	6 w	WHO,WHO	O1,2,4
Li et al. (2010)	III–IV	>60	>3 m	Un	36/36	39/33	29–75	50–100 ml,15 days,2cycles	G:1 g/m^2^; P:30 mg/m^2^	6 w	WHO,WHO	O1,2,4
Liu et al. (2010)	III–IV	>60	>3 m	Un	32/32	37/27	45–75	50 ml,14days,4cycles	G:1 g/m^2^; P:30 mg/m^2^	12 w	WHO, No	O1,4
Shi et al. (2010)	IIIa–IV	≥60	≥3 m	PT	28/28	47/9	48–72	50 ml,14days,2cycles	G:1 g/m^2^; P:30 mg/m^2^	6 w	WHO,WHO	O1,2,4
Ding et al. (2011)	III–IV	>50	>3 m	Un	18/22	27/13	Un	50 ml,10days,2cycles	G:1.4 g/m^2^; P:40 mg/m^2^	8 w	Un, WHO	O1,2,4
Fan et al. (2011)	IIIb–IV	≥60	>3 m	Un	41/38	54/25	39–73	50 ml,21 days,2-4cycles	G:1 g/m^2^; P:30 mg/m^2^	6–12 w	WHO, No	O1,2
He et al. (2011)	IIIb–IV	≥60	>3 m	Un	29/23	29/23	21–74	50–100 ml,15 days,2-3cycles	G:1 g/m^2^; P:75 mg/m^2^	8–11 w	WHO,WHO	O1,2,4
Jiang et al. (2011)	IIIb–IV	≥60	>3 m	PT	32/30	39/23	60–75	100 ml,14days,2cycles	G:1 g/m^2^; P:80 mg/m^2^	6 w	WHO, No	O1,2,5
Lu et al. (2011)	IIIb–IV	≥60	>6 m	Un	34/34	39/29	40–76	100 ml,14 days,2cycles	G:1 g/m^2^; P:80 mg/m^2^	6 w	WHO, Un	O1,2,4
Wu and He. (2011)	III–IV	Un	Un	Un	30/30	41/19	45–77	100 ml,16 days,2cycles	G:1 g/m^2^; P:30 mg/m^2^	6 w	WHO, No	O1
Fu. (2012)	IIIb–IV	Un	>3 m	Un	35/35	Un	61–84	50 ml,14 days,2cycles	G:1 g/m^2^; P:30 mg/m^2^	6 w	WHO, Un	O1,4
Pei. (2012)	IIIb–IV	Un	Un	PT	40/40	47/33	39–72	50 ml,8 days,2cycles	G:1 g/m^2^; P:20 mg/m^2^	6 w	RECIST	O1
Sun et al. (2012)	IIIb–IV	Un	>3 m	Un	34/34	42/26	60–86	50 ml,10 days,2cycles	G:1 g/m^2^; P:30 mg/m^2^	6 w	RECIST, CTCAE	O1,2,4
Wang. (2012)	III–IV	≥60	>3 m	Un	25/24	35/14	56.8 ± 9.1/57.8 ± 10.2	60 ml,14 days,3cycles	G:1 g/m^2^; P:25 mg/m^2^	9 w	WHO, Un	O1,4
Wang and Peng. (2012)	IIIb–IV	≥70	≥3 m	Un	36/36	46/26	32–74	80 ml,10 days,2-4cycles	G:1 g/m^2^; P:40 mg/m^2^	6–12 w	RECIST, WHO	O1,2,4
Xu et al. (2012)	IIIb–IV	≥70	Un	Un	33/33	36/30	Un	80 mg,10 days,4cycles	G:1.25 g/m^2^;P:100 mg/m^2^	13 w	RECIST, WHO	O1,4
Zhang. (2012)	IIIb–IV	≥60	>3 m	Un	41/42	63/20	57.2 ± 9.4/58.2 ± 10.3	60 ml,14 days,3cycles	G:1 g/m^2^; P:25 mg/m^2^	9 w	WHO, Un	O1,4
Cai et al. (2013)	IIIa–IV	≥60	Un	Un	19/19	21/17	36–68	50–100 ml,15 days,2cycles	G:1 g/m^2^; P:30mg/m2	6 w	Un	O4
Ju et al. (2013)	IIIb–IV	Un	>3 m	Un	34/34	36/32	61–81	50 ml,14 days,2cycles	G:1 g/m^2^; P:50 mg/m^2^	6 w	WHO, Un	O1,2,4
Lai. (2013)	IIIb–IV	Un	Un	Un	70/70	73/67	45–79	50 ml,14 days,>2cycles	G:1 g/m^2^; P:30 mg/m^2^	Un	WHO,WHO	O1,2,4
Xu et al. (2013)	IIIb–IV	Un	Un	Un	38/42	55/25	39–81	50 ml/14 days/3cycles	G:1 g/m^2^; P:30 mg/m^2^	9 w	WHO,WHO	O1,2,4,5
Li and Yang. (2014)	IIIb–IV	Un	>3 m	PT	27/27	32/22	34–68	50ml/8–10 days/4cycles	G:1 g/m^2^; P:75 mg/m^2^	12 w	RECIST, CTCAE	O1,3,4
Liu and Zhao. (2014)	IIIb–IV	≥60	≥3 m	Un	43/43	53/33	39–73	50ml/8–10 days/2cycles	G:1 g/m^2^; P:50 mg/m^2^	6 w	WHO,WHO	O1,4
Liu and Zhang. (2014)	IIIb–IV	Un	Un	Un	24/24	30/18	35–80	60 ml/21 days/2cycles	G:0.2 g/m^2^; P:25 mg/m^2^	6 w	Un, Un	O2,4
Wen. (2014)	IIIb–IV	≥60	≥3 m	Un	45/45	64/26	61–81	50 ml/21 days/2cycles	G:1 g/m^2^; P:50 mg/m^2^	6 w	RECIST, CTCAE	O1,2,4
Cheng et al. (2014)	IIIb–IV	≥70	≥3 m	PT	49/52	78/23	27–74	50–100 ml/10 days/2cycles	G:1–1.25 g/m^2^;P:25 mg/m^2^	2 years	RECIST, WHO	O1-4
Li et al. (2014)	IIIb–IV	>60	>3 m	Un	30/30	28/32	40–81	50 ml/10 days/2cycles	G:1 g/m^2^; P:50 mg/m^2^	Un	WHO, Un	O1,2,4
Zhang. (2014)	III–IV	Un	Un	Un	64/64	68/60	57–79	50 ml/10days/Un	G:1 g/m^2^; P:30 mg/m^2^	8 w	ELISA,FCM	O5
Han et al. (2015)	IIIb–IV	Un	Un	Un	36/36	39/33	48–67	50 ml,Un,3cycles	G:1 g/m^2^; P:Un	12 w	WHO, Un, FCM	O1,4,5
Li. (2015)	IIIb–IV	≥60	≥3 m	Un	20/20	24/16	45–74	50 ml/10 days/2cycles	G:1 g/m^2^; P:80 mg/m^2^	6 w	WHO, Un	O2,4,5
Ning et al. (2015)	III–IV	Un	Un	RT	31/31	49/13	45–75	50 ml/14 days/3cycles	G:1 g/m^2^; P:75 mg/m^2^	1 year	WHO,WHO	O1,3,4
Zhang. (2015)	III–IV	Un	Un	Un	39/32	37/34	60–83	50 ml/10 days/2cycles	G:1 g/m^2^; P:30 mg/m^2^	8 w	RECIST, CTCAE	O1,4
Zhao et al. (2015)	III–IV	≥70	>3 m	Un	43/43	58/28	43–79	100 ml/10 days/Un	G:1 g/m^2^; P:25 mg/m^2^	Un	WHO,WHO,FCM	O1,2,4,5
Zhu. (2015)	IIIb–IV	≥60	>3 m	Un	21/21	22/20	60–75	100 ml/14 days/Un	G:1 g/m^2^; P:25 mg/m^2^	Un	WHO,WHO	O1,4
Chen. (2016)	III–IV	>60	>3 m	Yes	30/30	36/24	42–76	80 ml/8 days/2cycles	G:1 g/m^2^; P:25 mg/m^2^	6 w	WHO	O1
Fang. (2016)	III–IV	≥70	Un	Un	45/45	Un/Un	40–70	50 ml/10 days/2cycles	G:1 g/m^2^; P:75 mg/m^2^	6 w	WHO,CTCAE	O1,4
Li et al. (2016)	IIIb–IV	≥60	>3 m	Un	47/47	52/42	40–70	50–100 ml/28 days/1cycles	G:1 g/m^2^; P:30 mg/m^2^	12 w	WHO, Un, FCM	O1,2,4,5
Li. (2016)	III–IV	>60	>6 m	Un	35/35	43/27	44–82	100 ml/14 days/1cycles	G:1 g/m^2^; P:80 mg/m^2^	6 w	WHO,WHO	O1,2,4
Ma and Jiang. (2016)	III–IV	>60	>3 m	Un	33/35	39/29	Un	60 ml/14 days/1cycles	G:1 g/m^2^; P:25 mg/m^2^	Un	WHO, Un	O1,4
Zhang. (2016a)	III–IV	Un	Un	Un	19/19	21/17	45–76	50 ml/28 days/1cycles	G:1 g/m^2^; P:25 mg/m^2^	6 w	WHO, Un	O1,2,4
Zhang. (2016b)	IV	Un	>3 m	PT	25/25	Un/Un	32–70	50 ml/10 days/4cycles	G:1 g/m^2^; P:75 mg/m^2^	12 w	RECIST, WHO	O1,2,4
Huang et al. (2017)	IIIb–IV	≥60	≥6 m	Un	39/40	46/33	49–70	60 ml/21 days/3cycles	G:1 g/m^2^; P:25 mg/m^2^	9 w	RECIST,WHO,FCM	O1,4,5
Ma. (2017)	IIIb–IV	≥60	≥6 m	Un	42/42	55/29	44–75	50ml/Un/4cycles	G:1 g/m^2^; P:75 mg/m^2^	16 w	WHO, Un, FCM	O1,4,5
Su. (2017)	IIIb–IV	Un	≥3 m	Un	40/39	45/34	40–70	50 ml/21 days/2cycles	G:1 g/m^2^; P:30 mg/m^2^	6 w	RECIST, Un, FCM	O1,4,5
Wu and Chen. (2017)	III–IV	Un	Un	Un	67/68	83/52	43–71	100 ml/10 days/4cycles	G:1 g/m^2^; P:20 mg/m^2^	12 w	WHO, Un	O1,4
Wu et al. (2017b)	III–IV	Un	Un	Un	109/109	137/79	32–72	50 ml/14 days/3cycles	G:1 g/m^2^; P:80 mg/m^2^	9 w	Un, FCM	O2,4,5
Zhang et al. (2017)	III–IV	Un	Un	Un	54/54	64/40	46–79	60–100 ml/10 days/4cycles	G:1 g/m^2^; P:30 mg/m^2^	12 w	WHO, Un	O1,2,4
Lv et al. (2018)	III–IV	>70	>3 m	Un	30/30	35/25	56–75	50 ml/14 days/Un	G:1 g/m^2^; P:25 mg/m^2^	Un	WHO,FCM	O1,5
Zhang. (2018)	III–IV	Un	≥5m	Un	40/40	43/27	38–72	60 ml/10 days/2cycles	G:1 g/m^2^; P:25 mg/m^2^	6 w	Un	O4
Zhou. (2018)	III–IV	Un	≥3 m	Un	58/58	63/53	41–70	50 ml/20 days/Un	G:1 g/m^2^; P:25 mg/m^2^	Un	RECIST, Un	O1,4
Su and Zhang. (2019)	III–IV	≥60	≥3 m	Un	41/41	54/28	42–78	Un/21 days/4-6cycles	G:1–1.25 g/m^2^; P:50 mg/m^2^	12–18 w	RECIST, Un	O1,2,4
Liu et al. (2019)	IIIb–IV	Un	Un	Un	44/44	54/34	42–76	50ml/Un/2cycles	G:1 g/m^2^; P:20 mg/m^2^	Un	Un	O1,5
Zhao and Li. (2010)	III–IV	Un	≥3 m	PT	43/43	55/31	64.0 ± 2.3/63.5 ± 2.6	50 ml/21dayays/2cycles	G:1.0 g/m^2^; P:80 mg/m^2^	6 w	WHO, Un	O1,3,5
Chen. (2020)	III–IV	Un	Un	Un	49/49	51/47	61–86	50–100 ml/10 days/Un	G:1–1.25 g/m^2^; P:25 mg/m^2^	Un	RECIST, Un	O1,2,4
Geng et al. (2020)	III–IV	≥70	≥3 m	Un	45/45	61/29	44–79	50 ml/14 days/4cycles	G:1.0 g/m^2^; P:30 mg/m^2^	8 w	WHO, Un	O1,4
Tan et al. (2020)	IIIb–IV	>60	≥3 m	Un	60/60	78/42	60–80	50 ml/10 days/2cycles	G:1.0 g/m^2^; P:30 mg/m^2^	6 w	RECIST,WHO,FCM	O1,2,4,5
Guo. (2020)	IIIb–IV	Un	≥3 m	Un	51/51	58/44	43–75	60 ml/14 days/4cycles	G:1.0 g/m^2^; P:25 mg/m^2^	12 w-1 year	WHO,FCM	O3,4,5
Xu and Li. (2020)	IIIb–IV	Un	Un	Un	51/45	53/37	42–82	60 ml/21 days/3cycles	G:1.0 g/m^2^; P:25 mg/m^2^	9 w	WHO	O1,2
Xu. (2020)	III–IV	Un	>6 m	Un	40/40	43/27	49–72	50–100 ml/21 days/2cycles	G:1.0 g/m^2^; P:20 mg/m^2^	6 w	RECIST,Un,FCM	O1,4,5

**Note:** GP: Gemcitabine and cisplatin; E: Experimental group (Aidi plus GP); C: Control group (GP alone); KPS score: Karnofsky Performance Status score; TP: treatment process; PT: primary treatment; RT: retreatment; AST: anticipated survival time; M: male; F: female; WHO: World Health Organization guidelines for solid tumor responses; RECIST: Response Evaluation Criteria in Solid Tumors; FCM: flow cytometry; O1: clinical efficacy included ORR and DCR; O2: quality of life (QOL); O3: patient survival; O4: adverse drug reactions (ADRs); and O5: antitumor immunity.

### Methodological Bias Risk

**FIGURE 2:**
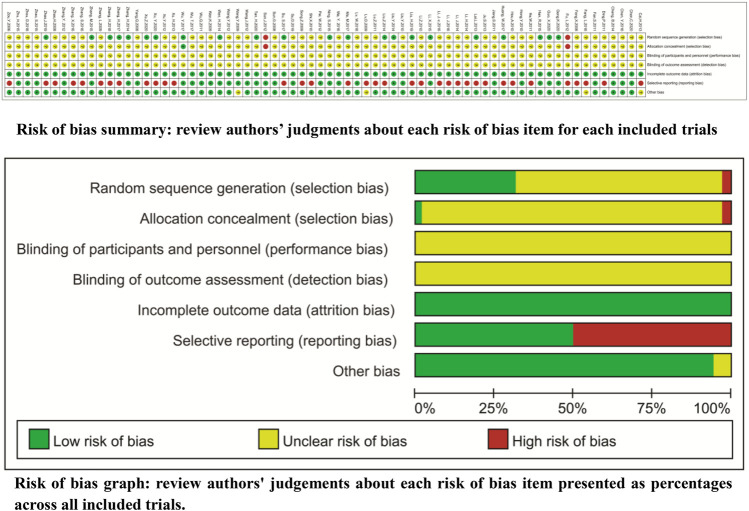
Risk of methodological bias Only 24 trials reported the random number table, draw, computer random, or odd–even random. Two trials reported the exposure of allocation. None of the trials reported the blinding and all trials had complete follow-up.

For the method of generating random sequences, only 24 trials used the random number table (Lai, 2013; Liu and Zhao, 2014; Wen, 2014; Zhang, 2014; Han et al., 2015; Li, 2015; Ning et al., 2015; Zhang, 2015; Huang et al., 2017; Ma, 2017; Su, 2017; Zhang et al., 2017; Zhang, 2018; Liu et al., 2019; Zhao and Li, 2019; Geng et al., 2020; Guo, 2020; Tan et al., 2020; Xu, 2020; Xu and Li, 2020), draw (Liu and Zhang, 2014), computer random (Wu et al., 2017b), or odd–even random (Fu, 2012; Sun et al., 2012). For the allocation, only two trials (Fu, 2012; Sun et al., 2012) reported the exposure of allocation. None of the trials reported blinding, and all trials had complete follow-up. Two trials selectively reported the tumor response (Ding et al., 2011; Xu and Li, 2020) and survival (Li and Yang, 2014; Zhao and Li, 2019). Seven trials selectively reported QOL (Ding et al., 2011; Lu et al., 2011; Xu et al., 2012; Li et al., 2014; Li et al., 2016; Zhang et al., 2017; Xu and Li, 2020), 27 trials selectively reported ADRs, and four trials (Feng et al., 2008; Xu et al., 2013; Su, 2017; Liu et al., 2019) selectively reported the levels of PBLs. In addition, 66 trials had baseline comparability, and four trials (Lv et al., 2009; Wang, 2009; Cai et al., 2013; Fang, 2016) had unclear comparability. We summarize the risk features of methodological bias in Figure 2.

### Tumor Responses

**FIGURE 3:**
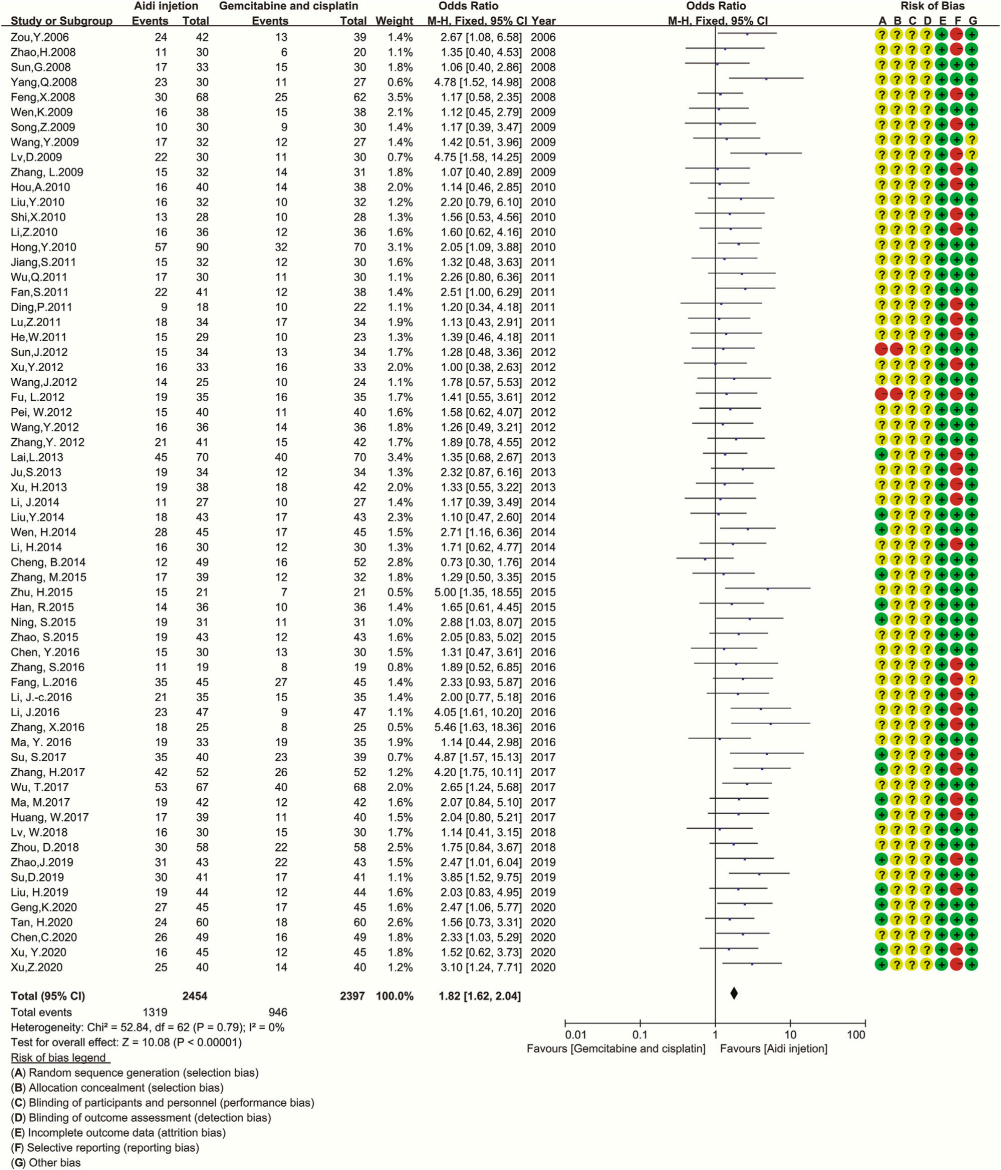
The analysis of tumor response between two groups 3a.The analysis of ORR between two groups. The pooled result showed a significant improvement in ORR in the Aidi and GP combination group compared to that in the control group (OR = 1.82, 95% CI [1.62 to 2.04], *p* < 0.00001). Note: GP, Gemcitabine, and cisplatin; ORR, objective response rate. 3b. The analysis of DCR between two groups. The pooled result showed a significant improvement in DCR in the Aidi and GP combination group compared to that in the control group (OR = 2.29, 95% CI [1.97 to 2.67], *p* < 0.00001). Note: GP, Gemcitabine, and cisplatin; DCR, disease control rate.

Sixty-three trials involving 4,851 patients compared the ORR and DCR (Figures 3A,B). Cochran’s χ^2^ test and *I* ^2^ statistic showed no statistical heterogeneity in ORR and DCR (*I* ^2^ = 0%). Therefore, we pooled the OR of ORR and DCR using a FEM. The pooled result showed a significant improvement in tumor responses (ORR and DCR) in the Aidi and GP combination group compared to that in the control group (OR = 1.82, 95% CI [1.62 to 2.04], *p* < 0.00001; OR = 2.29, 95% CI [1.97 to 2.67], *p* < 0.00001).

### Quality of Life

**FIGURE 4:**
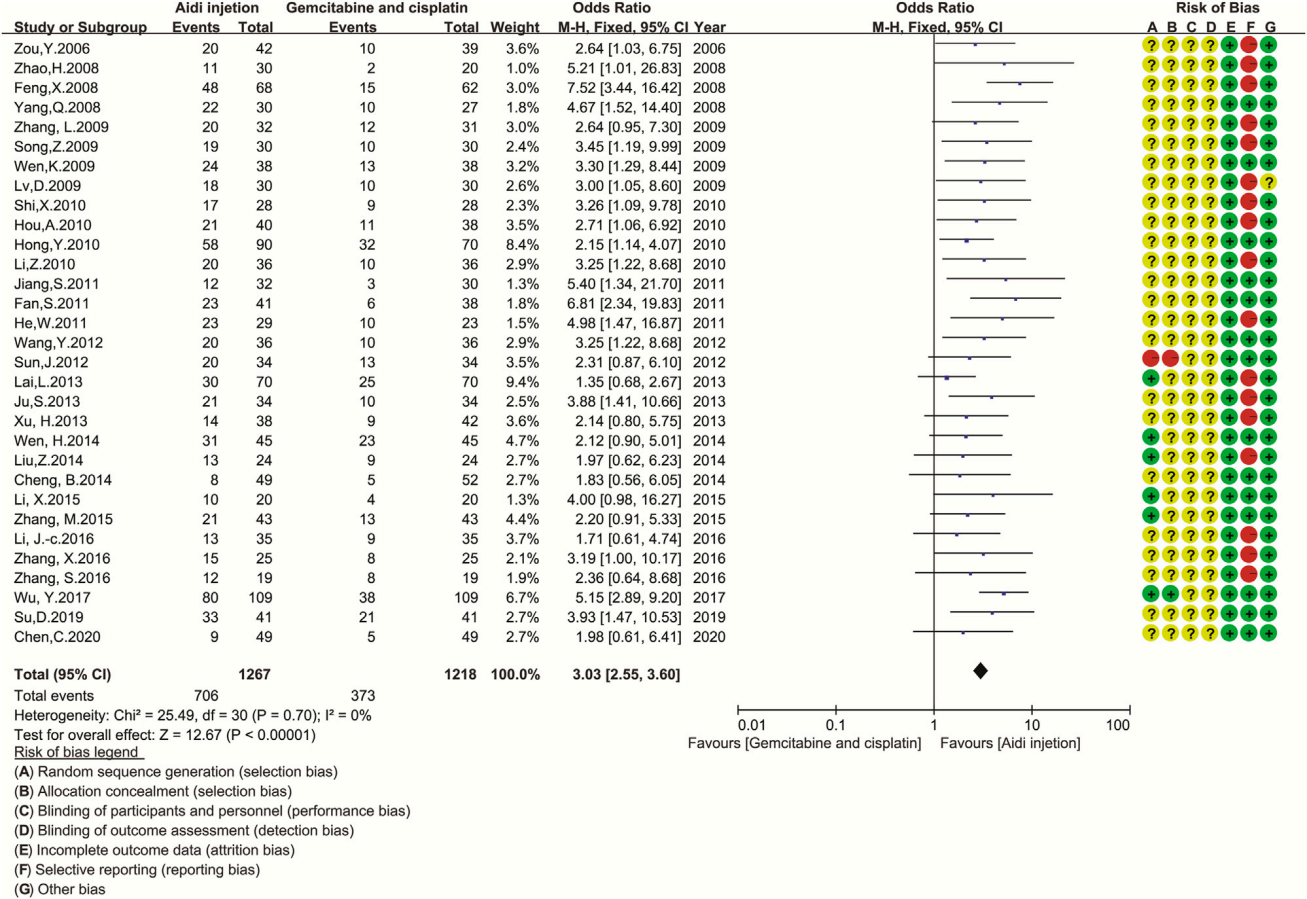
The analysis of QOL between two groups. The pooled result showed a significant improvement in QOL in the Aidi and GP combination compared to that in the control group (OR = 3.03, 95% CI [2.55 to 3.60], *p* < 0.00001). Note: GP, Gemcitabine and cisplatin; QOL, quality of life.

Thirty-one trials involving 2,485 patients completely compared the QOL in the two groups (Figure 4). Cochran’s χ^2^ test and *I* ^2^ statistic showed no significant heterogeneity in QOL (*I* ^2^ = 0%). Therefore, we pooled the OR of QOL using an FEM. The pooled result showed a significant improvement in QOL in the Aidi and GP combination compared to that in the control (OR = 3.03, 95% CI [2.55 to 3.60], *p* < 0.00001).

### Overall Survival

**FIGURE 5:**
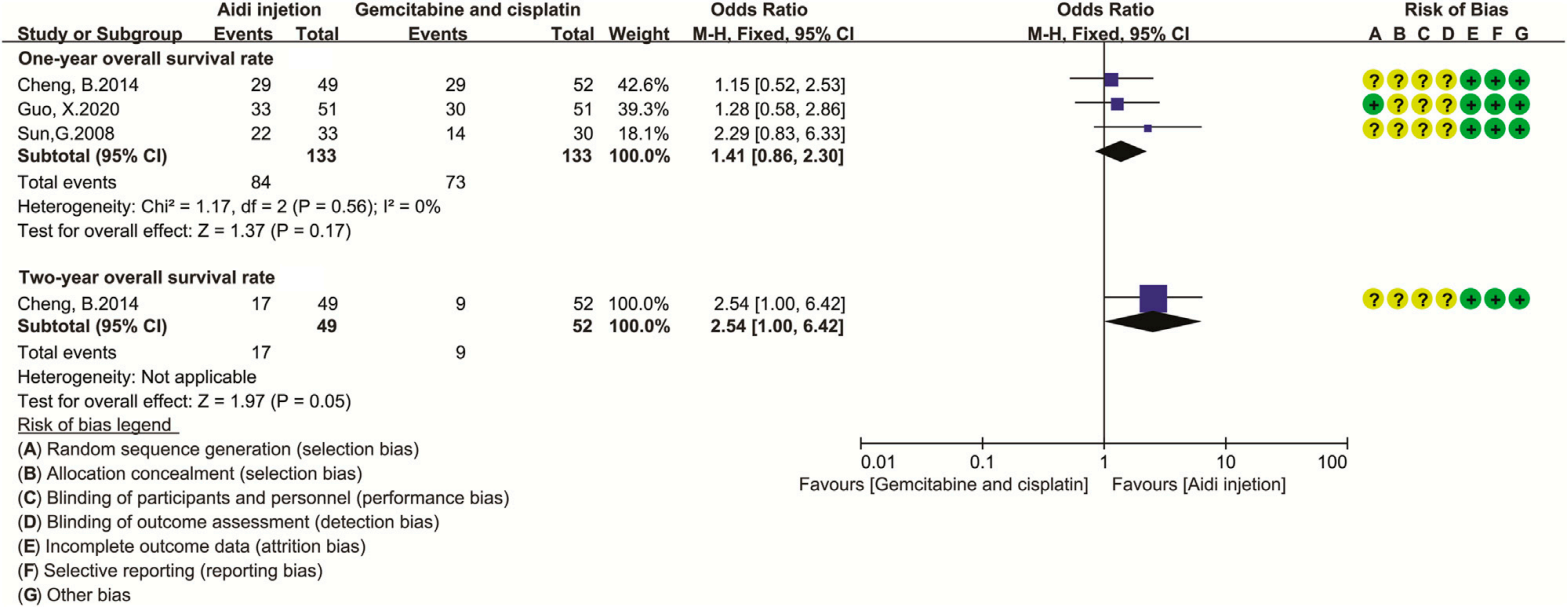
The analysis of overall survival rate. The result showed no difference in one- and two-year OS rate between the two groups (OR = 1.41, 95% CI [0.86 to 2.30], *p* = 0.17; and OR = 2.54, 95% CI [1.00 to 6.42], *p* = 0.05). Note: GP, Gemcitabine and cisplatin.

Only three trials (Sun et al., 2008; Cheng et al., 2014; Guo, 2020) compared the OS rate in the two groups (Figure 5). Cochran’s χ^2^ test and *I* ^2^ statistic showed no significant heterogeneity in the one-year OS rate (*I* ^2^ = 0%). *Therefore,* we pooled the ORs of the one-year OS rate using a FEM. The results showed no significant difference in the one-year OS rate and two-year OS rate between the two groups (OR = 1.41, 95% CI [0.86 to 2.30], *p* = 0.17; and OR = 2.54, 95% CI [1.00 to 6.42], *p* = 0.05).

### Levels of Antitumor Immunity

**FIGURE 6:**
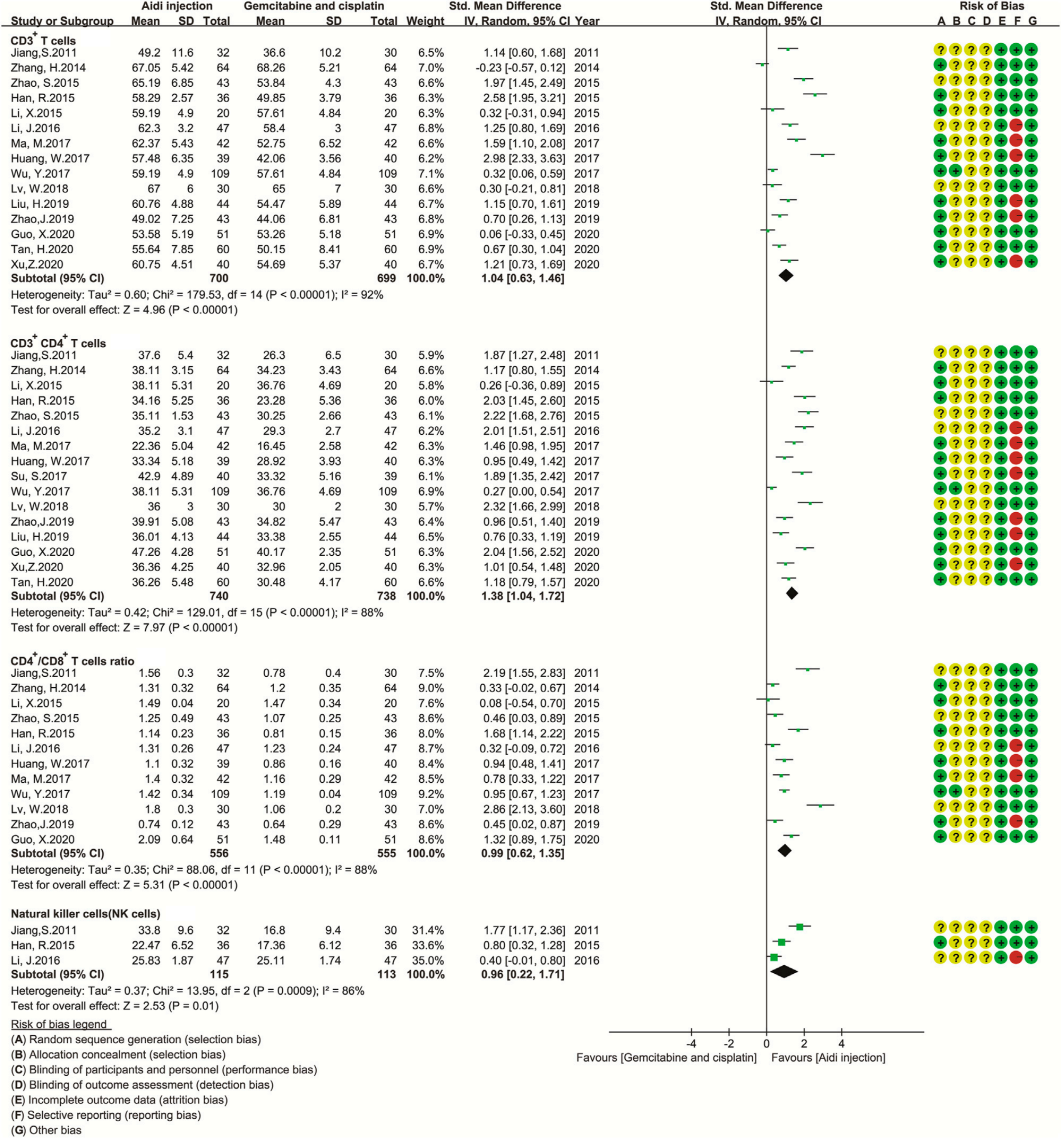
The analysis of antitumor immunity. The pooled result showed a significant up-regulation in CD3^+^ T cells, CD3^+^ CD4^+^ T cells, CD4^+^/CD8^+^ T cell ratios, and NK cell activity in the Aidi and GP combination group (SMD = 1.04, 95% CI [0.63 to 1.46], *p* < 0.00001); SMD = 1.38, 95% CI [1.04 to 1.72], *p* < 0.00001; SMD = 0.99, 95% CI [0.62 to1.35], *p* < 0.00001; SMD = 0.96, 95% CI [0.22 to 1.71], *p* = 0.01).

Eighteen trials involving 1,688 patients (Feng et al., 2008; Jiang et al., 2011; Xu et al., 2013; Zhang, 2014; Han et al., 2015; Li, 2015; Zhao et al., 2015; Li et al., 2016; Wu et al., 2017b; Huang et al., 2017; Ma, 2017; Su, 2017; Lv et al., 2018; Liu et al., 2019; Zhao and Li, 2019; Guo, 2020; Tan et al., 2020; Xu, 2020) compared the antitumor immunity in the two groups (Figure 6). Cochran’s χ^2^ test and I^2^ statistic showed significant heterogeneity in CD3^+^ T cells (*I* ^2^ = 92%), CD3^+^ CD4^+^ T cells (*I* ^2^ = 88%), CD4^+^/CD8^+^ T cell ratios (*I* ^2^ = 88%), and NK cells (*I* ^2^ = 86%) and *significant* clinical heterogeneity in CD3^+^ CD8^+^ T cells. Then, we pooled only the SMD of CD3^+^ T cells, CD3^+^ CD4^+^ T cells, CD4^+^/CD8^+^ T cell ratios, and NK cell activity using REM. The pooled result showed a significant upregulation in CD3^+^ T cells, CD3^+^ CD4^+^ T cells, CD4^+^/CD8^+^ T cell ratios, and NK cell activity (SMD = 1.04, 95% CI [0.63 to 1.46], *p* < 0.00001); SMD = 1.38, 95% CI [1.04 to 1.72], *p* < 0.00001; SMD = 0.99, 95% CI [0.62 to 1.35], *p* < 0.00001; SMD = 0.96, 95% CI [0.22 to 1.71], *p* = 0.01).

In addition, we developed a subgroup analysis model to analyze the causes of heterogeneity in the levels of PBLs (Supplementary Table S6; Supplementary Figures S1–S24). The results of the subgroup analysis showed that the AST, treatment time, and dosage of DDP might be the causes of heterogeneity in CD3^+^ T cells (Supplementary Table S6; Supplementary Figures S4, S16, S22); the KPS score, AST, dosage of Aidi and DDP, and treatment cycles might be the causes in CD3^+^ CD4^+^ T cells (Supplementary Table S6; Supplementary Figures S2, S5, S14, S20, S23). Theage, dosage of Aidi and DDP, treatment time, and treatment cycle might be the causes in CD4^+^/CD8^+^ T cells (Supplementary Table S6; Supplementary Figures S12, S15, S18, S21, S24).

### Adverse Drug Reactions

Fifty-eight trials involving 4,596 patients compared the ADRs in the two groups (Table 2; Supplementary Figures S25–S34). Cochran’s χ^2^ test and *the I* ^2^ statistic showed a significant heterogeneity in alopecia (*I* ^2^ = 70%), minimal heterogeneity in myelosuppression (*I* ^2^ = 31%), and no heterogeneity (*I* ^2^ = 0%) in other ADRs. Therefore, we pooled the ORs of alopecia using an REM, and the ORs of other ADRs using an FEM. The pooled result showed a significant decrease in myelosuppression, neutropenia, thrombocytopenia, anemia, gastrointestinal toxicity, liver toxicity, and renal toxicity (OR = 0.36, 95% CI [0.28 to 0.47], *p* < 0.00001; OR = 0.41, 95% CI [0.35 to 0.49], *p* < 0.00001); OR = 0.48, 95% CI [0.39 to 0.59], *p* < 0.00001; OR = 0.59, 95% CI [0.43 to 0.80], *p* = 0.0009; OR = 0.45, 95% CI [0.39 to 0.51], *p* < 0.00001; OR = 0.58, 95% CI [0.47 to 0.72], *p* < 0.00001; and OR = 0.62, 95% CI [0.48 to 0.79], *p* = 0.0001) and no significant differences in alopecia, neurotoxicity, and oral mucositis.

**TABLE 2:** Meta-analysis results of adverse drug reactions (Supplementary Figures S25–S34).

Indicators	Trials	Aidi injection with GP (Events/Total)	GP alone (Events/Total)	SM	Odds ratio, 95% CI	I^2^	*P*
Myelosuppression (Supplementary Figure S25)	17	218/642	342/632	FEM	0.36 [0.28, 0.47]	31%	*p* < 0.00001
Neutropenia (Supplementary Figure S26)	40	626/1701	862/1670	FEM	0.41 [0.35, 0.49]	0%	*p* < 0.00001
Thrombocytopenia (Supplementary Figure S27)	28	286/1181	409/1156	FEM	0.48 [0.39, 0.59]	0%	*p* < 0.00001
Anemia (Supplementary Figure S28)	11	156/524	200/523	FEM	0.59 [0.43, 0.80]	5%	*p* = 0.0009
Gastrointestinal toxicity (Supplementary Figure S29)	49	756/2043	1059/2002	FEM	0.45 [0.39, 0.51]	0%	*p* < 0.00001
Liver toxicity (Supplementary Figure S30)	29	192/1284	285/1257	FEM	0.58 [0.47, 0.72]	0%	*p* < 0.00001
Renal toxicity (Supplementary Figure S31)	24	132/1070	192/1044	FEM	0.62 [0.48, 0.79]	0%	*p* = 0.0001
Alopecia (Supplementary Figure S32)	3	41/94	57/89	REM	0.27 [0.05, 1.37]	70%	*p* = 0.11
Neurotoxicity (Supplementary Figure S33)	5	26/208	37/208	FEM	0.63 [0.35, 1.12]	0%	*p* = 0.11
Oral mucositis (Supplementary Figure S34)	3	10/106	18/106	FEM	0.50 [0.22, 1.16]	0%	*p* = 0.11

Note: GP: gemcitabine and cisplatin; SM: statistical method; REM: random-effects model; and FEM: fixed-effects model.

### Subgroup and Meta-Regression Analysis

This study included patients with KPS scores (≥60 or ≥70) or AST (≥3 months or ≥5 months). For patients with a KPS score ≥60 or AST ≥3 months, the results of subgroup analyses showed that the Aidi and GP combination achieved a significant improvement in the ORR and DCR and a low incidence rate of neutropenia and gastrointestinal toxicity (Tables 3A,B; ; Supplementary Figures S35–S50). We included patients with treatment *processes* (PT, RT, or PT/RT) and with age (≥60 or others). For patients with PT or age (≥60), the pooled results showed that *the* Aidi and GP combination also achieved the same effects (Tables 3C,D; Supplementary Figures S51–S66). Univariate random effects meta-regression manifested significant differences in the relationship between age and neutropenia (Tables 3D; Supplementary Figures S63–S64).

**TABLE 3:** Subgroup and meta-regression analysis.

Subgroups	Objective response rate (ORR)			Disease control rate (DCR)			Neutropenia			Gastrointestinal toxicity		
	OR (95% CI)	UM	MM	OR (95% CI)	UM	MM	OR (95% CI)	UM	MM	OR (95% CI)	UM	MM
Table 3A.Subgroups analysis according to KPS score (Supplementary Figures S35–S42)												
KPS score (≥60)	1.87 [1.58, 2.22]	0.22	0.27	2.31 [1.84, 2.89]	0.57	0.47	0.42 [0.33, 0.54]	0.82	0.90	0.41 [0.33, 0.50]	0.32	0.64
KPS score (≥70)	1.40 [1.03, 1.90]			2.01 [1.36, 2.98]			0.33 [0.20, 0.54]			0.45 [0.31, 0.66]		
KPS score (others)	1.94 [1.61, 2.33]			2.39 [1.87, 3.06]			0.43 [0.33, 0.55]			0.50 [0.40, 0.62]		
Table 3B.Subgroups analysis according to AST (Supplementary Figures S35–S50)												
AST (≥3m)	1.81 [1.56, 2.09]	0.28	0.99	2.20 [1.82, 2.66]	0.72	0.98	0.42 [0.34, 0.53]	0.78	0.90	0.47 [0.39, 0.56]	0.32	0.42
AST (≥5m)	1.98 [1.31, 3.00]			2.90 [1.69, 4.99]			0.40 [0.26, 0.61]			0.26 [0.15, 0.46]		
AST (unclear)	1.80 [1.45, 2.24]			2.35 [1.74, 3.18]			0.40 [0.29, 0.55]			0.46 [0.36, 0.59]		
Table 3C. Subgroups analysis via treatment process (Supplementary Figures S51–S58)												
Primary treatment (PT)	1.46 [1.10, 1.94]	0.08	0.06	2.04 [1.41, 2.94]	0.27	0.20	0.46 [0.28, 0.76]	0.42	0.52	0.54 [0.36, 0.82]	0.81	0.41
Retreatment (RT)	2.88 [1.03, 8.07]			2.13 [0.62, 7.29]			1.00 [0.37, 2.71]			0.59 [0.22, 1.62]		
PT and RT	1.08 [0.62, 1.89]			1.43 [0.65, 3.14]			0.29 [0.13, 0.63]			0.32 [0.16, 0.65]		
Unclear	1.95 [1.71, 2.23]			2.41 [2.02, 2.87]			0.40 [0.33, 0.48]			0.44 [0.37, 0.51]		
Table 3D. Subgroups analysis via age (Supplementary Figures S59–S66)												
Age (≥60)	1.85 [1.36, 2.51]	0.91	0.88	2.22 [1.49, 3.30]	0.86	0.56	0.25 [0.15, 0.42]	0.04	0.09	0.46 [0.31, 0.69]	0.80	0.99
Age (others)	1.81 [1.60, 2.06]			2.30 [1.95, 2.72]			0.44 [0.37, 0.53]			0.44 [0.38, 0.52]		
Table 3E. Subgroups analysis according to dosage (Supplementary Figures S67–S74)												
50–60 ml	1.72 [1.49, 2.00]	0.75	0.88	2.21 [1.82, 2.68]	0.35	0.21	0.45 [0.37, 0.56]	0.32	0.58	0.47 [0.39, 0.56]	0.09	0.21
70–80 ml	1.85 [1.28, 2.68]			2.80 [1.73, 4.52]			0.24 [0.12, 0.48]			0.39 [0.25, 0.62]		
90–100 ml	1.89 [1.35, 2.64]			2.55 [1.57, 4.14]			0.34 [0.21, 0.53]			0.30 [0.19, 0.48]		
Others	2.16 [1.61, 2.89]			2.18 [1.48, 3.21]			0.41 [0.27, 0.63]			0.48 [0.35, 0.67]		
Table 3F. Subgroups analysis according to time per cycle (Supplementary Figures S75–S82)												
7–10days	1.75 [1.43, 2.13]	0.18	0.18	2.19 [1.70, 2.82]	0.48	0.53	0.35 [0.27, 0.46]	0.32	0.61	0.44 [0.35, 0.57]	0.97	0.41
14–15days	1.62 [1.35, 1.94]			1.87 [1.46, 2.38]			0.40 [0.31, 0.53]			0.45 [0.37, 0.55]		
21–28days	2.48 [1.90, 3.25]			3.59 [2.46, 5.24]			0.62 [0.39, 1.00]			0.42 [0.29, 0.63]		
Others	1.92 [1.13, 3.28]			3.23 [1.64, 6.37]			0.56 [0.28, 1.14]			No		
Table 3G. Subgroups analysis according to treatment cycle (Supplementary Figures S83–S90)												
One cycle	2.22 [1.44, 3.43]	0.70	0.40	2.54 [1.46, 4.41]	1.00	0.74	0.37 [0.21, 0.63]	0.35	0.78	0.41 [0.26, 0.66]	0.21	0.08
Two cycles	1.65 [1.40, 1.94]			2.08 [1.67, 2.58]			0.35 [0.27, 0.45]			0.46 [0.37, 0.57]		
Three cycles	1.75 [1.24, 2.47]			2.48 [1.55, 3.96]			0.61 [0.41, 0.90]			0.45 [0.31, 0.65]		
Four cycles	2.32 [1.68, 3.22]			2.59 [1.69, 3.95]			0.40 [0.27, 0.61]			0.35 [0.24, 0.51]		
Others	1.91 [1.44, 2.52]			2.53 [1.75, 3.64]			0.45 [0.28, 0.70]			0.51 [0.37, 0.72]		
Table 3H. Subgroups analysis according to DDP dosage (Supplementary Figures S91–S98)												
20–30 mg/m^2^	1.83 [1.58, 2.12]	0.77	0.34	2.26 [1.87, 2.72]	0.98	0.75	0.43 [0.35, 0.53]	0.55	0.37	0.49 [0.41, 0.59]	0.28	0.27
40–50 mg/m^2^	1.88 [1.32, 2.69]			2.56 [1.52, 4.31]			0.38 [0.19, 0.76]			0.39 [0.26, 0.59]		
60–80 mg/m^2^	1.83 [1.43, 2.32]			2.25 [1.61, 3.15]			0.39 [0.28, 0.55]			0.39 [0.29, 0.52]		
Others	1.28 [0.64, 2.55]			2.68 [1.02, 7.01]			0.27 [0.11, 0.68]			0.22 [0.08, 0.66]		
Table 3I. Subgroups analysis according to DDP dosage (Supplementary Figures S35–S50)												
WHO	1.81 [1.58, 2.08]	0.85	0.87	2.17 [1.80, 2.62]	0.63	0.43	0.40 [0.32, 0.51]	0.66	0.91	0.45 [0.36, 0.55]	0.69	0.49
RECIST/NCI-CTCAE	1.85 [1.47, 2.31]			2.50 [1.90, 3.30]			0.36 [0.20, 0.66]			0.38 [0.22, 0.66]		
Other	1.70 [0.82, 3.50]			3.64 [1.18, 11.23]			0.43 [0.34, 0.56]			0.46 [0.37, 0.56]		

Note: AST: anticipated survival time; PT: primary treatment; RT: retreatment; Others: unclear or ungroupable; WHO: World Health Organization for solid tumor responses; OR: odds ratio; RECIST: Response Evaluation Criteria in Solid Tumors guideline; NCI-CTCAE: National *Cancer* Institute-Common Terminology Criteria for Adverse Events; UM: univariate meta-regression; and MM: multiple meta-regression.

Aidi was injected intravenously at 30–100 ml/day and 7–28 days/cycle for one to four cycles. In subgroups with Aidi usage (50–100 ml/day, 7–15 days/cycle for one to four cycles), the Aidi and GP combination showed a significant improvement in the tumor response and a low incidence rate of neutropenia and gastrointestinal toxicity (Tables 3E–G; Supplementary Figures S67–S90). Univariate meta-regression analysis also manifested any statistical difference in the relationship between Aidi usage and tumor response/ADRs (Tables 3E–G; Supplementary Figures S67–S90).

GEM (1000 mg/m^2^) is often used in combination with DDP (20–100 mg/m^2^). In subgroups with DDP usage (20–30 mg/m^2^, 40–50 mg/m^2^, or 60–80 mg/m^2^), the Aidi and GP combination also showed a significant improvement in the tumor response and a low incidence rate of neutropenia and gastrointestinal toxicity (Table 3H; Supplementary Figures S91–S98). There was no significance in the relationship between the dosage of DDP and tumor response/ADRs (Table 3H; Supplementary Figures S91–S98). The tumor responses (ORR and DCR) were evaluated using WHO (Miller et al., 1981) or RECIST guidelines (Watanabe et al., 2003), and the ADRs were evaluated using WHO (Miller et al., 1981) or CTCAE criteria (Trotti et al., 2003). Different criteria showed no positive effect on tumor responses and ADRs (Table 3I; Supplementary Figures S99–S106). Post hoc multiple regression analysis manifested no positive relationship between all variables and indicators (Table 3).

### Publication Bias Analysis

**FIGURE 7:**
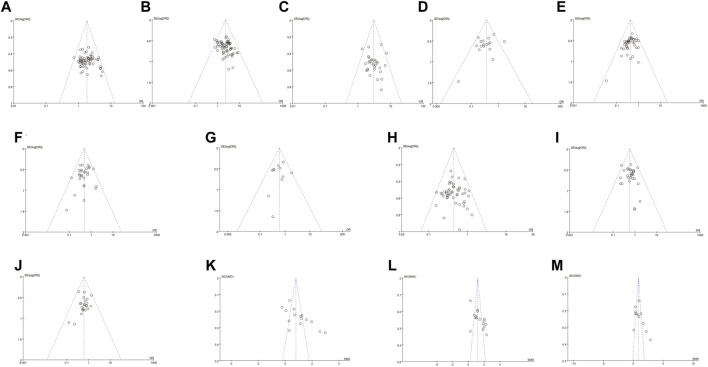
The analysis of publication bias. There was a significant publication bias for DCR, neutropenia, CD3^+^ T cells, and CD3^+^ CD4^+^ T cells (p = 0.02, 95% CI 0.21 to 2.28; p = 0.03, 95% CI –2.24 to –0.13; p = 0.01, 95% CI 3.94 to 16.11; and p = 0.001, 95% CI 4.04–13.58). Note: ORR, objective response rate; DCR, disease control rate.

We analyzed the potential publication bias using a funnel plot and Egger/Begg’s tests (Figure 7). The results showed that no publication bias was found for ORR, QOL, myelosuppression, thrombocytopenia, anemia, gastrointestinal toxicity, liver toxicity, renal toxicity, or CD4^+^/CD8^+^ T cell ratios (*p* = 0.32, 95% CI −0.81–2.41; *p* = 0.68, 95% CI −1.20–1.81; *p* = 0.15, 95% CI −3.84–0.62; *p* = 0.34, 95% CI −1.84–0.64; *p* = 0.49, 95% CI −2.64–1.36; *p* = 0.73, 95% CI −0.96–1.37; *p* = 0.44, 95% CI −0.72–1.60; *p* = 0.52, 95% CI −1.43–0.75; and *p* = 0.11, 95% CI −1.43–12.04, and the trials objectively reported them. However, significant publication bias was found for DCR, neutropenia, CD3^+^ T cells, and CD3^+^ CD4^+^ T cells (*p* = 0.02, 95% CI 0.21 to 2.28; *p* = 0.03, 95% CI −2.24–−0.13; *p* = 0.01, 95% CI 3.94 to 16.11; and *p* = 0.001, 95% CI 4.04–13.58). The trials overestimated DCR, CD3^+^ T cells, and CD3^+^ CD4^+^ T cells and underestimated neutropenia.

### Sensitivity Analysis

Poor trials were found for ORR, DCR, QOL, myelosuppression, neutropenia, thrombocytopenia, anemia, gastrointestinal toxicity, liver toxicity, renal toxicity, neurotoxicity**,** CD3^+^ T cells, CD3^+^ CD4^+^ T cells, CD4^+^/CD8^+^ T cell ratios, and natural killer cells. Before and after rejecting the poor trials, the pooled results were robust. The ORs for anemia and alopecia were poorly robust (Table 4A). Overestimated trials were found for ORR, DCR, QOL, CD3^+^ T cells, CD3^+^ CD4^+^ T cells, CD4^+^/CD8^+^ T cell ratios, and natural killer cells. Underestimated trials were found for myelosuppression, neutropenia, thrombocytopenia, anemia, gastrointestinal toxicity, liver toxicity, renal toxicity, and alopecia. Before and after rejecting the trials with overestimated efficacy or underestimated ADRs, the pooled results were robust (Table 4B).

**TABLE 4:** Sensitivity analysis.

Indicators	Trials	SM	OR (95%CI)	I^2^	Excluded trials (Reference number)	Trials	SM	OR (95%CI)	*I* ^2^
Table 4A. Sensitivity analysis by excluding the poor trials									
Objective response rate	63	FEM	1.82 [1.62, 2.04]	0%	Poor* Zou et al. (2006), Feng et al. (2008), Zhao et al. (2008), Lv et al. (2009), Song et al. (2009), Zhang. (2009), Hou and Zhang. (2010), Li et al. (2010), Shi et al. (2010), Ding et al. (2011), He et al. (2011), Lu et al. (2011), Fu. (2012), Sun et al. (2012), Xu et al. (2012), Ju et al. (2013), Lai. (2013), Xu et al. (2013), Li et al. (2014), Li and Yang. (2014), Zhao et al. (2015), Zhang. (2016b), Fang. (2016), Li. (2016), Li et al. (2016), Huang et al. (2017), Ma. (2017), Su. (2017), Zhang et al. (2017), Liu et al. (2019), Zhao and Li. (2019), Xu. (2020), Xu and Li. (2020)	30	FEM	1.80 [1.53, 2.13]	0%
Disease control rate	61	FEM	2.29 [1.97, 2.67]	0%	Poor* Zou et al. (2006), Feng et al. (2008), Lv et al. (2009), Song et al. (2009), Zhang. (2009), Hou and Zhang. (2010), Li et al. (2010), Shi et al. (2010), He et al. (2011), Lu et al. (2011), Fu. (2012), Sun et al. (2012), Xu et al. (2012), Ju et al. (2013), Lai. (2013), Xu et al. (2013), Li et al. (2014), Li and Yang. (2014), Zhang. (2016a), Zhang. (2016b), Fang. (2016), Li. (2016), Li et al. (2016), Huang et al. (2017), Ma. (2017), Su. (2017), Zhang. (2018), Liu et al. (2019), Zhao and Li. (2019), Xu. (2020), Xu and Li. (2020)	30	FEM	2.20 [1.78, 2.71]	0%
Quality of life	31	FEM	3.03 [2.55, 3.60]	0%	Poor*Zou et al. (2006), Feng et al. (2008), Zhao et al. (2008), Lv et al. (2009), Song et al. (2009), Zhang. (2009), Hou and Zhang. (2010), Li et al. (2010), Shi et al. (2010), He et al. (2011), Sun et al. (2012), Ju et al. (2013), Lai. (2013), Xu et al. (2013), Liu and Zhang. (2014), Zhang. (2016a), Zhang. (2016b), Li. (2016)	13	FEM	3.27 [2.54, 4.21]	0%
Myelosuppression	17	FEM	0.36 [0.28, 0.47]	31%	Poor* Zou et al. (2006), Lv et al. (2009), Song et al. (2009), Li et al. (2010), Shi et al. (2010), He et al. (2011), Xu et al. (2012), Cai et al. (2013), Ju et al. (2013), Lai. (2013), Li et al. (2014), Su. (2017)	5	REM	0.37 [0.16, 0.85]	69%
Neutropenia	40	FEM	0.0.41 [0.35, 0.49]	0%	Poor* Feng et al. (2008), Zhang. (2009), Hou and Zhang. (2010), Lu et al. (2011), Fu. (2012), Sun et al. (2012), Xu et al. (2012), Xu et al. (2013), Li and Yang. (2014), Zhang. (2016a), Fang. (2016), Li. (2016), Li et al. (2016), Huang et al. (2017), Ma. (2017), Su. (2017), Zhang et al. (2017), Zhang. (2018), Xu. (2020)	21	FEM	0.44 [0.35, 0.54]	0%
Thrombocytopenia	28	FEM	0.48 [0.39, 0.59]	0%	Poor* Feng et al. (2008), Zhang. (2009), Hou and Zhang. (2010), Xu et al. (2012), Xu et al. (2013), Li and Yang. (2014), Fang. (2016), Li et al. (2016), Huang et al. (2017), Su. (2017), Xu. (2020)	17	FEM	0.52 [0.40, 0.67]	4%
Anemia	11	FEM	0.59 [0.43, 0.80]	5%	Poor* Li and Yang. (2014), Li. (2016), Ma. (2017), Zhang. (2018)	7	FEM	0.71 [0.48, 1.04]	0%
Gastrointestinal toxicity	49	FEM	0.45 [0.39, 0.51]	0%	Poor* Zou et al. (2006), Feng et al. (2008), Lv et al. (2009), Song et al. (2009), Zhang. (2009), Hou and Zhang. (2010), Li et al. (2010), Liu et al. (2010), Shi et al. (2010), He et al. (2011), Lu et al. (2011), Sun et al. (2012), Xu et al. (2012), Cai et al. (2013), Ju et al. (2013), Lai. (2013), Xu et al. (2013), Li et al. (2014), Zhang. (2016a), Fang. (2016), Li. (2016), Li et al. (2016), Su. (2017), Zhang et al. (2017), Zhang. (2018)	24	FEM	0.44 [0.36, 0.53]	0%
Liver toxicity	29	FEM	0.58 [0.47, 0.72]	0%	Poor* Hou and Zhang. (2010), Sun et al. (2012), Li et al. (2014), Zhang. (2016a), Huang et al. (2017), Ma. (2017), Zhang et al. (2017)	22	FEM	0.57 [0.46, 0.72]	0%
Renal toxicity	24	FEM	0.62 [0.48, 0.79]	0%	Poor* Sun et al. (2012), Li et al. (2014), Huang et al. (2017), Ma. (2017)	20	FEM	0.60 [0.46, 0.79]	0%
Alopecia	3	REM	0.27 [0.05, 1.37]	70%	Poor* Song et al. (2009)	2	FEM	0.12 [0.04, 0.34]	0%
Neurotoxicity	5	FEM	0.63 [0.35, 1.12]	0%	Poor* Song et al. (2009)	4	FEM	0.58 [0.31, 1.09]	No
Table 4B. Sensitivity analysis by excluding the over- or under-estimated trials									
Objective response rate	63	FEM	1.82 [1.62, 2.04]	0%	Over* Zou et al. (2006), Yang et al. (2008), Lv et al. (2009), Hong et al. (2010), Wen. (2014), Ning et al. (2015), Zhu. (2015), Zhang. (2016b), Li et al. (2016), Su. (2017), Wu and Chen. (2017), Zhang et al. (2017), Su and Zhang. (2019), Zhao and Li. (2019), Chen. (. (2020), Geng et al. (2020), Xu. (2020)	46	FEM	1.47 [1.28, 1.69]	0%
Disease control rate	61	FEM	2.29 [1.97, 2.67]	0%	Over* Hong et al. (2010), Pei. (2012), Wang and Peng. (2012), Wen. (2014), Zhao et al. (2015), Zhang. (2016b), Fang. (2016), Li et al. (2016), Huang et al. (2017), Ma. (2017), Su. (2017), Zhou. (2018), Liu et al. (2019), Chen. (2020), Geng et al. (2020), Tan et al. (2020), Xu. (2020), Xu and Li. (2020)	43	FEM	1.78 [1.47, 2.16]	0%
Quality of life	31	FEM	3.03 [2.55, 3.60]	0%	Over* Zou et al. (2006), Feng et al. (2008), Yang et al. (2008), Zhao et al. (2008), Lv et al. (2009), Song et al. (2009), Wen et al. (2009), Hong et al. (2010), Hou and Zhang. (2010), Li et al. (2010), Shi et al. (2010), Fan et al. (2011), He et al. (2011), Jiang et al. (2011), Wang and Peng. (2012), Ju et al. (2013), Wu et al. (2017b), Su and Zhang. (2019)	13	FEM	2.07 [1.56, 2.73]	0%
Myelosuppression	17	FEM	0.36 [0.28, 0.47]	31%	Under*Lv et al. (2009), Li et al. (2010), Liu et al. (2010), Shi et al. (2010), He et al. (2011), Xu et al. (2012), Ju et al. (2013), Zhou. (2018), Su and Zhang. (2019)	8	FEM	0.61 [0.43, 0.86]	0%
Neutropenia	40	FEM	0.0.41 [0.35, 0.49]	0%	Under* Wen et al. (2009), Zhang. (2009), Hong et al. (2010), Hou and Zhang. (2010), Lu et al. (2011), Fu. (2012), Sun et al. (2012), Xu et al. (2012), Cheng et al. (2014), Liu and Zhao. (2014), Li. (2015), Zhang. (2015), Zhu. (2015), Fang. (2016), Li. (2016), Wu and Chen. (2017), Zhang. (2018), Chen. (2020), Guo. (2020)	21	FEM	0.56 [0.44, 0.71]	0%
Thrombocytopenia	28	FEM	0.48 [0.39, 0.59]	0%	Under* Hong et al. (2010), Wang and Peng. (2012), Xu et al. (2012), Zhu. (2015), Guo. (2020), Tan et al. (. (2020)	22	FEM	0.57 [0.45, 0.74]	0%
Anemia	11	FEM	0.59 [0.43, 0.80]	5%	Under* Li. (2016), Wu and Chen. (2017)	9	FEM	0.68 [0.48, 0.96]	0%
Gastrointestinal toxicity	49	FEM	0.45 [0.39, 0.51]	0%	Under* Lv et al. (2009), Wen et al. (2009), Zhang. (2009), Hong et al. (2010), Hou and Zhang. (2010), Shi et al. (2010), Lu et al. (2011), Xu et al. (2012), Wen. (2014), Zhu. (. (2015), Fang. (2016), Li. (2016), Li et al. (2016), Ma and Jiang. (2016), Wu et al. (2017b), Wu and Chen. (2017), Zhang et al. (2017), Zhang. (2018), Su and Zhang. (. (2019), Guo. (2020)	29	FEM	0.61 [0.51, 0.75]	0%
Liver toxicity	29	FEM	0.58 [0.47, 0.72]	0%	Under* Wu et al. (2017b), Wu and Chen. (2017), Su and Zhang. (2019)	26	FEM	0.68 [0.54, 0.86]	0%
Renal toxicity	24	FEM	0.62 [0.48, 0.79]	0%	Under* Wu et al. (2017b)	23	FEM	0.67 [0.51, 0.87]	0%
Alopecia	3	REM	0.27 [0.05, 1.37]	70%	Under* Wang. (2009), Liu et al. (2010)	1	No	1.38 [0.28, 6.80]	No
PBL	Trials	SM	SMD (95% CI)	I^2^	Excluded trials (Reference number)	Trials	SM	SMD (95% CI)	I^2^
Table 4C. Sensitivity analysis by excluding the poor trials									
CD3^+^ T cell	14	REM	1.12 [0.69, 1.55]	92%	Poor* Li et al. (2016), Huang et al. (2017), Ma. (2017), Liu et al. (2019), Zhao and Li. (2019), Xu. (2020)	8	REM	0.86 [0.29, 1.43]	93%
CD3^+^ CD4^+^ T cell	15	REM	1.34 [0.99, 1.68]	88%	Poor* Li et al. (2016), Huang et al. (2017), Ma. (2017), Su. (2017), Liu et al. (2019), Zhao and Li. (2019), Xu. (2020)	8	REM	1.40 [0.81, 1.98]	92%
CD4^+^/CD8^+^ T cell ratios	11	REM	0.96 [0.57, 1.35]	88%	Poor* Li et al. (2016), Huang et al. (2017), Ma. (2017), Liu et al. (2019), Zhao and Li. (2019)	7	REM	0.93 [0.47, 1.38]	86%
Natural killer cell	3	REM	0.96 [0.22, 1.71]	86%	Poor* Li et al. (2016)	2	REM	1.27 [0.32, 2.21]	84%
Table 4D. Sensitivity analysis by excluding the over- or under-estimated trials									
CD3^+^ T cell	14	REM	1.12 [0.69, 1.55]	92%	Over* Jiang et al. (2011), Han et al. (2015), Zhao et al. (2015), Li et al. (2016), Huang et al. (2017), Ma. (2017), Liu et al. (2019), Xu. (2020) Under* Zhang. (2014)	5	FEM	0.46 [0.28, 0.63]	0%
CD3^+^ CD4^+^ T cell	15	REM	1.34 [0.99, 1.68]	88%	Over* Jiang et al. (2011), Han et al. (2015), Zhao et al. (2015), Li et al. (2016), Ma. (2017), Su. (2017), Lv et al. (2018),under* Wu et al. (2017b)	7	FEM	0.97 [0.80, 1.13]	26%
CD4^+^/CD8^+^ T cell ratios	11	REM	0.96 [0.57, 1.35]	88%	Over* Jiang et al. (2011), Han et al. (2015), Wu et al. (2017b), Liu et al. (2019)	7	FEM	0.48 [0.32, 0.65]	0%
Natural killer cell	3	REM	0.96 [0.22, 1.71]	86%	Over* Jiang et al. (2011)	2	FEM	0.56 [0.25, 0.88]	37%

Note: PBL: Peripheral blood lymphocyte; SM: statistical method; FEM: fixed-effects model; OR: odds ratio; SMD: standardized mean difference; CI: confidence interval; Poor trial (Poor*) that had at least one domain being considered as high risk of bias; and Over* or Under*: over or underestimated trial which the result was significant difference and beneficial to Aidi injection use.

### Quality of Evidence

In methodology, this meta-analysis included 36 poor trials. The ORs of anemia and alopecia were poorly robust, and then we downgraded the quality by two levels. The ORs of other indicators were robust, and then we downgraded the quality by only one level. Cochran’s χ^2^ test and the *I* ^2^ statistic found significant heterogeneity for alopecia and levels of PBLs, and all indicators were robust, and not downgraded. The OS rates of patients with alopecia, oral mucositis, and natural killer cells were less than 300. Therefore, we downgraded their quality by one level. There was significant publication bias in DCR, neutropenia, CD3^+^ T cells, and CD3^+^ CD4^+^ T cells, and the pooled results were robust; therefore, their quality was not downgraded. Upgrade was unsuitable for any indicators. Finally, we summarized the quality of ORR, DCR, QOL, myelosuppression, neutropenia, thrombocytopenia, gastrointestinal toxicity, hepatorenal toxicity, neurotoxicity, CD3^+^ T cells, CD3^+^ CD4^+^ T cells, and CD4^+^/CD8^+^ T cell ratios as “moderate” and other indicators as “low to very low” (Table 5).

**TABLE 5:** GRADE evidence profile.

Table 5A. The clinical efficacy and safety										
Indicators (Trials)	Quality assessment					NSCLC		Clinical efficacy and safety		Quality
	Risk of bias	Inconsistency	Indirectness	Imprecision	Publication bias	Aidi injection	GP	Odds ratios (95% CI)	Absolute effects	
Objective response rate (63)	Serious^a^	No	No	No	None	1319/2454 (53.7%)	946/2397 (39.5%)	1.82 (1.62–2.04)	148 more per 1000 (from 119 more to 176 more)	⊕⊕⊕Ο Moderate
Disease control rate (61)	Serious^a^	No	No	No	None^b^	2095/2406 (87.1%)	1766/2355 (75%)	2.29 (1.97–2.67)	123 more per 1000 (from 105 more to 139 more)	⊕⊕⊕Ο Moderate
Quality of life (31)	Serious^a^	No	No	No	None	706/1267 (55.7%)	373/1218 (30.6%)	3.03 (2.55–3.6)	266 more per 1000 (from 223 more to 308 more)	⊕⊕⊕Ο Moderate
1-year OS rate (3)	Serious^c^	No	No	Serious^d^	None	84/133 (63.2%)	73/133 (54.9%)	1.41 (0.86–2.3)	83 more per 1000 (from 38 fewer to 188 more)	⊕⊕ΟΟ Low
2-years OS rate (1)	Serious^c^	No	No	Serious^d^	None	17/49 (34.7%)	9/52 (17.3%)	2.54 (1–6.42)	174 more per 1000 (from 0 more to 400 more)	⊕⊕ΟΟ Low
Myelosuppression (17)	Serious^a^	No	No	No	None	218/642 (34%)	342/632 (54.1%)	0.36 (0.28–0.47)	243 fewer per 1000 (from 185 fewer to 293 fewer)	⊕⊕⊕Ο Moderate
Neutropenia (40)	Serious^a^	No	No	No	None^e^	626/1701 (36.8%)	862/1670 (51.6%)	0.41 (0.35–0.49)	212 fewer per 1000 (from 173 fewer to 244 fewer)	⊕⊕⊕Ο Moderate
Thrombocytopenia (28)	Serious^a^	No	No	No	None	286/1181 (24.2%)	409/1156 (35.4%)	0.48 (0.39–0.59)	146 fewer per 1000 (from 110 fewer to 178 fewer)	⊕⊕⊕Ο Moderate
Anemia (11)	Very serious^f^	No	No	No	None	156/524 (29.8%)	200/523 (38.2%)	0.59 (0.43–0.8)	115 fewer per 1000 (from 51 fewer to 172 fewer)	⊕⊕ΟΟ Low
Gastrointestinal toxicity (49)	Serious^a^	No	No	No	None	756/2043 (37%)	1059/2002 (52.9%)	0.45 (0.39–0.51)	193 fewer per 1000 (from 165 fewer to 224 fewer)	⊕⊕⊕Ο Moderate
Liver toxicity (29)	Serious^a^	No	No	No	None^b^	192/1284 (15%)	285/1257 (22.7%)	0.58 (0.47–0.72)	81 fewer per 1000 (from 52 fewer to 106 fewer)	⊕⊕⊕Ο Moderate
Renal toxicity (24)	Serious^a^	No	No	No	None	132/1070 (12.3%)	192/1044 (18.4%)	0.62 (0.48–0.79)	61 fewer per 1000 (from 33 fewer to 86 fewer)	⊕⊕⊕Ο Moderate
Alopecia (3)	Very serious^f^	No^g^	No	Serious^d^	None	41/94 (43.6%)	57/89 (64%)	0.27 (0.05–1.37)	316 fewer per 1000 (from 559 fewer to 69 more)	⊕ΟΟΟ Very low
Neurotoxicity (5)	Serious^a^	No	No	Serious^d^	None	26/208 (12.5%)	37/208 (17.8%)	0.63 (0.35–1.12)	58 fewer per 1000 (from 107 fewer to 17 more)	⊕⊕⊕Ο Moderate
Oral mucositis (3)	Serious^a^	No	No	Serious^d^	None	10/106 (9.4%)	18/106 (17%)	0.5 (0.22–1.16)	77 fewer per 1000 (from 127 fewer to 22 more)	⊕⊕ΟΟ Low
Table 5B. The levels of peripheral blood lymphocytes										
Indicators (Trials)	Risk of bias	Inconsistency	Indirectness	Imprecision	Publication bias	Aidi injection	GP	Odds ratios (95% CI)	SMD (95% CI)	Quality
CD3^+^ T cell (15)	Serious^a^	No^g^	No	No	None^b^	700	699	No	1.04 higher (0.63–1.46 higher)	⊕⊕⊕Ο Moderate
CD3^+^ CD4^+^ T cell (16)	Serious^a^	No^g^	No	No	None^b^	740	738	No	1.38 higher (1.04–1.72 higher)	⊕⊕⊕Ο Moderate
CD4^+^/CD8^+^ T cell ratios (12)	Serious^a^	No^g^	No	No	None	556	555	No	0.99 higher (0.62–1.35 higher)	⊕⊕⊕Ο Moderate
Natural killer cell (3)	Serious^a^	No^g^	No	Serious^d^	None	115	113	No	0.96 higher (0.22–1.71 higher)	⊕⊕ΟΟ Low

Note: NSCLC: non-small cell lung cancer; GP: gemcitabine and cisplatin; OS: overall survival; SMD: standardized mean difference; and CI: confidence interval.

a Most trials had unclear risk and with high risk, the result of sensitivity analysis was robust, and the evidence was downgraded by only one level

b with publication bias, the result was overestimated and robust, and not downgraded

c most trials had unclear risk and without high risk, the evidence was downgraded by only one level

d the sample size for result <300 cases, and the evidence was downgraded by one level

e with publication bias, the result was underestimated and robust, and not downgraded

f most trials had unclear risk and with high risk, the result of sensitivity analysis was poorly robust, and the evidence was downgraded by two levels

g with heterogeneity, the results was robust, and not downgraded

## Discussion

Based on three previous SRs/meta-analyses (Yang and Ding, 2012; Han et al., 2016; Xiao et al., 2017) and six related SRs/meta-analyses (Ma et al., 2009; Tian et al., 2014; Xiao et al., 2016; Wu et al., 2017a; Xiao et al., 2018b; Wang et al., 2018), we included 70 trials for this meta-analysis, which involved 5,509 NSCLC patients including 3,278 males and 1,995 females with ages ranging from 21–86 years old. The experimental group with 2,783 cases received the Aidi and GP combination, and the control group with 2,726 cases received the GP alone. Aidi was intravenously injected at 30–100 ml/day, 7–28 days per cycle for one to four cycles. GEM (1000 mg/m^2^) was mainly used in combination with DDP (20–100 mg/m^2^). After six weeks to two years of follow-up, the trials evaluated tumor response, survival, QOL, antitumor immunity, and ADRs.

**FIGURE 8:**
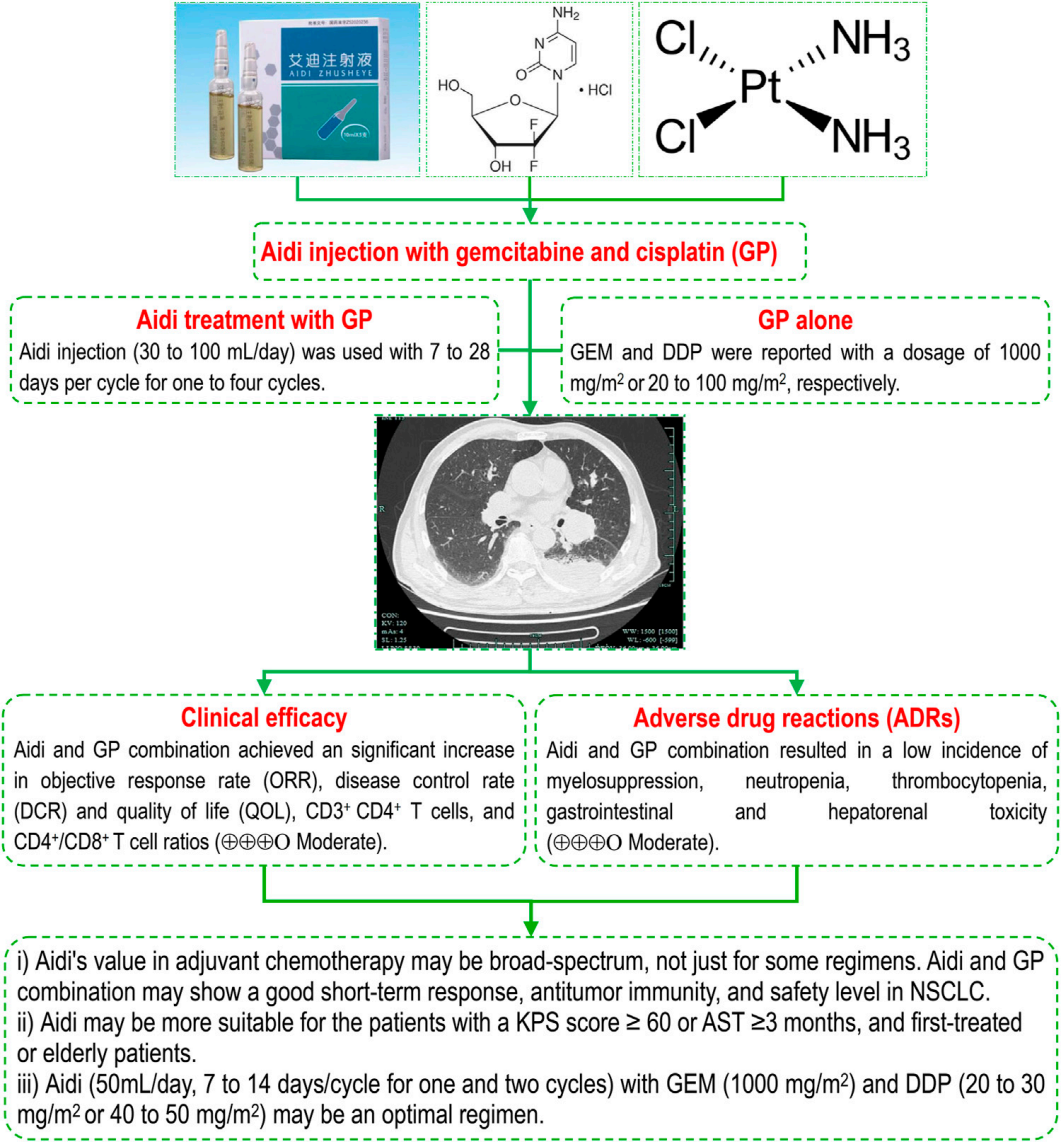
Aidi injection and GP combination in NSCLC, Aidi's value in adjuvant chemotherapy may be broad-spectrum, not just for some regimens. The Aidi and GP combination may show a good short-term response, antitumor immunity, and safety level in NSCLC. Aidi may be more suitable for patients with a KPS score ≥60 or AST ≥3 months, and first-treated or elderly patients. Aidi (50 ml/day, 7–14 days/cycle for one and two cycles) with GEM (1000 mg/m^2^) and DDP (20–30 mg/m^2^ or 40–50 mg/m^2^) may be an optimal regimen. Note: AST, anticipated survival time; DCR, disease control rate; DDP, cisplatin; GEM, Gemcitabine; KPS: KPS, Karnofsky Performance Status; NSCLC, non-small cell lung cancer; ORR, objective response rate.

Gemcitabine and cisplatin is one of the standard regimens in the treatment of advanced NSCLC. As a cantharidin-based CHI and important adjuvant drug, Aidi is often used in combination with GP to treat NSCLC (Yang and Ding, 2012; Han et al., 2016; Xiao et al., 2017). Three years ago, we reported that the Aidi and GP combination might improve the tumor response and QOL with a low risk of hematotoxicity and gastrointestinal toxicity in patients (Xiao et al., 2017). However, the methodology had many shortcomings, and new trials have been published (Chen, 2020; Guo, 2020; Tan et al., 2020; Xu, 2020). Therefore, we further improved the methodology, integrated all previous three SRs/meta-analyses (Yang and Ding, 2012; Han et al., 2016; Xiao et al., 2017), and supplemented 34 trials with 2,927 cases for this meta-analysis. The pooled results demonstrated that the Aidi and GP combination significantly improved the ORR, DCR, and QOL, reduced the incidences of hematotoxicity and gastrointestinal and hepatorenal toxicity, and upregulated the levels of CD3^+^ T cells and CD3^+^ CD4^+^ T cells, CD4^+^/CD8^+^ T cell ratios, and NK cell activity. However, the pooled results of PBLs showed significant heterogeneity, and further subgroup analysis revealed the patient features, DDP, and Aidi usage might be important causes of heterogeneity. Moreover, four trials with 320 patients reported survival (Sun et al., 2008; Cheng et al., 2014; Li and Yang, 2014; Guo, 2020), and no trials reported PFS. However, current evidence does not support whether the Aidi and GP combination improves survival. Most trials had unclear risk, some had high risk, and some indicators were overestimated or underestimated. Nevertheless, most results were robust, and their quality was “moderate” (Figure 8).

Aidi injection is composed of cantharidin, astragaloside, ginsenoside, elentheroside E, and syringin (Zhang et al., 2012; Zeng et al., 2016). The results from clinical and basic studies revealed that Aidi has an important antitumor, immunoregulatory, anti-inflammatory and antioxidative stress function (Duan et al., 2018b; Li et al., 2018; Zhou et al., 2018; Xiao et al., 2019a; Chen et al., 2019; Farag et al., 2019; Huang et al., 2019; Li et al., 2019; Qu et al., 2019; Zhang et al., 2019). The results of previous SRs/meta-analyses (Ma et al., 2009; Xiao et al., 2016; Wu et al., 2017a; Xiao et al., 2018a; Xiao et al., 2018b; Wang et al., 2018; Xiao et al., 2019b) demonstrated that Aidi in combination with chemotherapy showed significant improvements in tumor response, QOL, and OS rate, and decreases in ADRs. Recently, we found that Aidi in combination with NP might improve the tumor response and QOL, upregulate antitumor immunity, and reduce the incidence of hematotoxicity and gastrointestinal and liver toxicity in patients with NSCLC (Xiao et al., 2020b). After this study, we further found that Aidi in combination with chemotherapy resulted in a low incidence of hepatorenal toxicity in patients with lung cancer (Xiao et al., 2020a). In addition, another meta-analysis (Xiao et al., 2016) revealed that Aidi might significantly upregulate the antitumor immunity in NSCLC patients undergoing platinum-based chemotherapy. This meta-analysis further demonstrated that the Aidi and GP combination also significantly improved the tumor response and QOL, upregulated the antitumor immunity, and decreased the incidences of ADRs, especially GP-induced hepatorenal toxicity (Figure 8). More interestingly, the results further demonstrated that Aidi may show an important protective function for the liver and kidney in NSCLC patients undergoing chemotherapy. In addition, current evidence does not support that the Aidi and GP combination improves survival. All these findings demonstrated that Aidi had an important clinical value in improving short-term responses and antitumor immunity and reducing ADRs. Aidi's value in adjuvant chemotherapy may be broad-spectrum, not just for some regimens. Therefore, the strategy of Aidi use should focus on reducing toxicity and improving tumor responses and QOL.

In previous studies (Xiao et al., 2020a; Xiao et al., 2020b), we found that patient features and Aidi and DDP usage might be important influencing factors in obtaining an ideal tumor response and safety level for NSCLC. For patients with a KPS score ≥60, the Aidi and NP combination may produce an ideal tumor response and achieve a good safety level (Xiao et al., 2020b). Aidi decreased the risk of hepatorenal toxicity for patients with KPS scores ≥60, PT and who are elderly (Xiao et al., 2020a). The results indicate that patients who are first-treated, elderly, patients with a KPS score ≥60, or AST ≥3 months may be appropriate populations for Aidi. In this meta-analysis, series subgroup analyses further revealed that the Aidi and GP combination also achieved an ideal response and safety level in patients with KPS scores ≥60, AST ≥3 months, PT or age ≥60. The patients with a KPS score ≥ 60 or AST ≥3 months and patients who were first-treated or elderly might be appropriate populations for Aidi and GP combinations. These populations may have a treatment threshold for Aidi and GP combinations, which is of important clinical significance for standardizing the Aidi treatment (Figure 8). Unfortunately, no evidence supports that Aidi achieves the same effects in patients with retreatment or drug resistance, which needs to be confirmed by new trials.

In the GP regimen, GEM and DDP are recommended at dosages of 1000 mg/m^2^ or 75 mg/m^2^, respectively, according to the guidelines (Association et al., 2018). Wang Z. et al. (Wang and Zhu, 2015) reported that Aidi treatment with a 60% dose of GP or NP might achieve the same tumor response as the conventional dose. Ruan F. et al. (Ruan and Lu, 2012) reported that Aidi treatment with low-dose capecitabine might improve tumor responses and survival in advanced gastric cancer. Our previous studies (Xiao et al., 2020a; Xiao et al., 2020b) also reported that Aidi in combination with vinorelbine and cisplatin (low- or high-dose) might achieve the same tumor response and good safety level (Xiao et al., 2020b). In this meta-analysis, we further confirmed a similar relationship between Aidi and GP. The results of subgroup analyses further revealed that Aidi (50 ml/day, 7–14 days/cycle for one and two cycles) in combination with GEM (1000 mg/m^2^) and DDP (20–30 mg/m^2^, 40–50 mg/m^2^) might obtain the above effects (Figure 8). The results indicate that Aidi has a similar dosage, treatment time, and cycle in combination with different regimens, which is beneficial to further standardized/rational drug use. In addition, Aidi treatment with low- or high-dose DDP might both obtain satisfactory effects, and Aidi might decrease the dosage of DDP and show a synergistic effect with different chemotherapy regimens, which will be of important clinical significance in improving the prognosis of patients by innovating chemotherapy strategies based on synergistic effects. However, a post hoc univariate and multiple regression analysis found only one positive correlation. These conclusions were drawn from the subgroup analysis and need to be further confirmed by new studies. Based on the optimization of efficacy and safety, Aidi treatment (50 ml/day, 7–14 days/cycle for one and two cycles) with GEM (1000 mg/m^2^) and DDP (20–30 mg/m^2^ or 40–50 mg/m^2^) may be an optimal therapy for realizing an ideal goal. If confirmed, these findings will be of importance for developing a standardized and rational drug use strategy against advanced NSCLC.

Some limitations exist in this meta-analysis. First, we collected the trials only from Chinese and English-language databases, which might have resulted in retrieval bias. Second, methodologically, the bias risk of most trials was unclear, and 36 trials selectively reported the clinical efficacy, ADRs, and PBLs; the quality was “moderate” to “very low.” Third, only some trials provided the baseline information such as age, KPS score, AST, retreatment, and drug resistance. Limited trials and patients were available to analyze the OS, phlebitis, and antitumor immunity, and none of the trials reported the PFS. Fourth, this meta-analysis did not support that the Aidi and GP combination is suitable for patients with retreatment or drug resistance or improves survival. We have not further explored the optimal conditions to achieve ideal antitumor immunity. Fifth, the univariate and multiple meta-regression analyses found only one positive correlation, and these conclusions from the subgroup analysis belonged to indirect evidence. All of these questions need further high-quality study to determine.

## Conclusion

Current evidence indicates that Aidi’s value in adjuvant chemotherapy may be broad-spectrum, not just for some regimens. The Aidi and GP combination may show a good short-term response, antitumor immunity, and a safety level in patients with NSCLC. Aidi may be more suitable for patients with a KPS score ≥60 or AST ≥3 months, patients first-treated, and elderly patients. Aidi treatment (50 ml/day, 7–14 days/cycle for one and two cycles) with GEM (1000 mg/m^2^) and DDP (20–30 mg/m^2^ or 40–50 mg/m^2^) may be an optimal therapy for realizing an ideal goal. Moreover, Aidi may decrease the dosage of DDP use and show a synergistic effect with different regimens. Finally, we hope that this meta-analysis will provide valuable evidence by which to develop an optimal CHI treatment strategy against advanced NSCLC.

## Glossary

ADRs: Adverse drug reactions
Aidi: Aidi injection
AST: anticipated survival time
CBM: China Biological Medicine database
CENTRAL: Cochrane Central Register of Controlled Trials database;
CHIs: Chinese herbs injections
CIs: Confidence intervals
CNKI: China National Knowledge Infrastructure (CNKI) database
CR: Complete response
CTCAE: Common terminology criteria for adverse events
DCR: disease control rate
DDP: cisplatin
FCM: flow cytometry
FEM: Fixed-effects model
GP: Gemcitabine and cisplatin
GRADE: Grading of Recommendations Assessment Development and Evaluation approach
GEM: Gemcitabine
HR: Hazard ratio
KPS: Karnofsky Performance Status score
MM: Multiple meta-regression
NC: No change
NK cell: Natural killer cell
NSCLC: Non-small cell lung cancer
OR: Odds ratio
ORR: Objective response rate
OS: overall survival
PBLs: Peripheral blood lymphocytes
PD: Progressive disease
PFS: Progression-free survival
PR: Partial response
PRISMA: Preferred Reporting Items for Systematic Reviews and Meta-Analyses guidelines
PT: Primary treatment
QOL: Quality of life
RCT: Randomized controlled trial
RECIST: Response Evaluation Criteria in Solid Tumors
REM: Random-effects model
RT: Retreatment
SM: Statistical method
SMD: Standardized mean difference
SRs: Systematic reviews
TCM: Traditional Chinese medicine
TNM staging system: Tumor node metastasis staging system
TP: Treatment process
UM: Univariate meta-regression
VIP: Chinese Scientific Journals Full-Text database
WHO: World Health Organization
