# Supplementary material for: The Optimal Adjuvant Strategy of Aidi Injection With Gemcitabine and Cisplatin in Advanced Non–small Cell Lung Cancer: A Meta-analysis of 70 Randomized Controlled Trials
Source: Front Pharmacol. 2021 May 28;12:582447. doi: 10.3389/fphar.2021.582447 (PMC8194277; doi:10.3389/fphar.2021.582447)
Supplement: Supplementary file 1 [file DataSheet2.pdf]

# Supplementary material 5. Meta-analysis results of adverse drug reactions (Figures S25-34)

| Study or Subgroup | Aidi injection Events | Aidi injection Total | Gemcitabine and cisplatin Events | Gemcitabine and cisplatin Total | Weight | Odds Ratio M-H, Fixed, 95% CI |
|---|---|---|---|---|---|---|
| Cai,H.2013 | 5 | 19 | 8 | 19 | 2.9% | 0.49 [0.13, 1.93] |
| Geng,K.2020 | 12 | 45 | 8 | 45 | 2.9% | 1.68 [0.61, 4.62] |
| He,W.2011 | 9 | 29 | 17 | 23 | 6.4% | 0.16 [0.05, 0.54] |
| Ju,S.2013 | 11 | 34 | 23 | 34 | 7.6% | 0.23 [0.08, 0.63] |
| Lai,L.2013 | 25 | 70 | 31 | 70 | 9.8% | 0.70 [0.35, 1.38] |
| Li, H.2014 | 16 | 30 | 21 | 30 | 4.8% | 0.49 [0.17, 1.41] |
| Li,Z.2010 | 10 | 36 | 21 | 36 | 7.4% | 0.27 [0.10, 0.74] |
| Liu,Y.2010 | 3 | 32 | 11 | 32 | 4.9% | 0.20 [0.05, 0.80] |
| Lv,D.2009 | 16 | 30 | 24 | 30 | 5.5% | 0.29 [0.09, 0.90] |
| Shi,X.2010 | 16 | 28 | 23 | 28 | 4.8% | 0.29 [0.09, 0.98] |
| Song,Z.2009 | 7 | 30 | 12 | 30 | 4.5% | 0.46 [0.15, 1.40] |
| Su,D.2019 | 4 | 41 | 13 | 41 | 5.8% | 0.23 [0.07, 0.79] |
| Su, S.2017 | 2 | 40 | 3 | 39 | 1.4% | 0.63 [0.10, 4.00] |
| Wen, H.2014 | 17 | 45 | 26 | 45 | 7.9% | 0.44 [0.19, 1.03] |
| Xu,Y.2012 | 23 | 33 | 33 | 33 | 5.1% | 0.03 [0.00, 0.60] |
| Zhou, D.2018 | 12 | 58 | 34 | 58 | 13.2% | 0.18 [0.08, 0.42] |
| Zou,Y.2006 | 30 | 42 | 34 | 39 | 4.9% | 0.37 [0.12, 1.16] |
| **Total (95% CI)** | | **642** | | **632** | **100.0%** | **0.36 [0.28, 0.47]** |
| Total events | 218 | | 342 | | | |

Heterogeneity: Chi² = 23.27, df = 16 (P = 0.11); I² = 31%
Test for overall effect: Z = 7.83 (P < 0.00001)

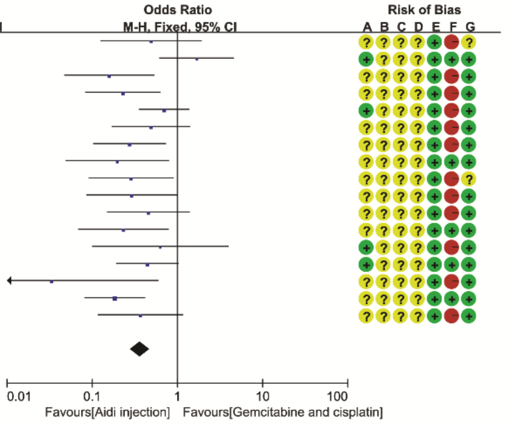


Risk of bias legend
(A) Random sequence generation (selection bias)
(B) Allocation concealment (selection bias)
(C) Blinding of participants and personnel (performance bias)
(D) Blinding of outcome assessment (detection bias)
(E) Incomplete outcome data (attrition bias)
(F) Selective reporting (reporting bias)
(G) Other bias

## Figure S25 The analysis of myelosuppression between the two groups

| Study or Subgroup | Aidi injection Events | Aidi injection Total | Gemcitabine and cisplatin Events | Gemcitabine and cisplatin Total | Weight | Odds Ratio M-H, Fixed, 95% CI | Year |
|---|---|---|---|---|---|---|---|
| Feng,X.2008 | 64 | 68 | 60 | 62 | 0.9% | 0.53 [0.09, 3.02] | 2008 |
| Wang,Y.2009 | 26 | 32 | 22 | 27 | 1.0% | 0.98 [0.26, 3.67] | 2009 |
| Wen,K.2009 | 23 | 38 | 31 | 38 | 2.8% | 0.35 [0.12, 0.99] | 2009 |
| Zhang, L.2009 | 5 | 32 | 14 | 31 | 2.8% | 0.22 [0.07, 0.74] | 2009 |
| Hong,Y.2010 | 26 | 90 | 31 | 70 | 5.7% | 0.51 [0.27, 0.98] | 2010 |
| Hou,A.2010 | 14 | 40 | 22 | 38 | 3.4% | 0.39 [0.16, 0.98] | 2010 |
| Lu,Z.2011 | 11 | 34 | 20 | 34 | 3.1% | 0.33 [0.12, 0.90] | 2011 |
| Zhang,Y. 2012 | 12 | 41 | 17 | 42 | 2.7% | 0.61 [0.24, 1.52] | 2012 |
| Xu,Y.2012 | 23 | 33 | 33 | 33 | 2.4% | 0.03 [0.00, 0.60] | 2012 |
| Fu, L.2012 | 24 | 35 | 32 | 35 | 2.3% | 0.20 [0.05, 0.81] | 2012 |
| Wang,J.2012 | 7 | 25 | 9 | 24 | 1.5% | 0.65 [0.19, 2.16] | 2012 |
| Wang,Y.2012 | 19 | 36 | 26 | 36 | 2.8% | 0.43 [0.16, 1.15] | 2012 |
| Sun,J.2012 | 23 | 34 | 30 | 34 | 2.2% | 0.28 [0.08, 0.99] | 2012 |
| Xu, H.2013 | 13 | 38 | 22 | 42 | 3.2% | 0.47 [0.19, 1.17] | 2013 |
| Cheng, B.2014 | 34 | 49 | 47 | 52 | 3.2% | 0.24 [0.08, 0.73] | 2014 |
| Liu,Y.2014 | 27 | 43 | 36 | 43 | 3.1% | 0.33 [0.12, 0.91] | 2014 |
| Li, J.2014 | 5 | 27 | 6 | 27 | 1.1% | 0.80 [0.21, 3.00] | 2014 |
| Li, X.2015 | 2 | 20 | 8 | 20 | 1.7% | 0.17 [0.03, 0.92] | 2015 |
| Zhao, S.2015 | 7 | 43 | 10 | 43 | 1.9% | 0.64 [0.22, 1.88] | 2015 |
| Zhang, M.2015 | 26 | 39 | 28 | 32 | 2.4% | 0.29 [0.08, 0.99] | 2015 |
| Ning, S.2015 | 16 | 31 | 16 | 31 | 1.8% | 1.00 [0.37, 2.71] | 2015 |
| Zhu, H.2015 | 6 | 21 | 15 | 21 | 2.5% | 0.16 [0.04, 0.61] | 2015 |
| Han, R.2015 | 7 | 36 | 11 | 36 | 2.0% | 0.55 [0.18, 1.63] | 2015 |
| Li, J.-c.2016 | 14 | 35 | 26 | 35 | 3.6% | 0.23 [0.08, 0.64] | 2016 |
| Ma, Y. 2016 | 7 | 33 | 15 | 35 | 2.7% | 0.36 [0.12, 1.05] | 2016 |
| Fang, L.2016 | 5 | 45 | 13 | 45 | 2.7% | 0.31 [0.10, 0.95] | 2016 |
| Li, J.2016 | 12 | 47 | 19 | 47 | 3.3% | 0.51 [0.21, 1.21] | 2016 |
| Zhang, S.2016 | 2 | 19 | 4 | 19 | 0.8% | 0.44 [0.07, 2.76] | 2016 |
| Zhang, H.2017 | 13 | 52 | 22 | 52 | 3.8% | 0.45 [0.20, 1.05] | 2017 |
| Ma, M.2017 | 11 | 42 | 16 | 42 | 2.7% | 0.58 [0.23, 1.46] | 2017 |
| Huang, W.2017 | 10 | 39 | 12 | 40 | 2.0% | 0.80 [0.30, 2.16] | 2017 |
| Su, S.2017 | 2 | 40 | 2 | 39 | 0.4% | 0.97 [0.13, 7.28] | 2017 |
| Wu, Y.2017 | 3 | 109 | 9 | 109 | 2.0% | 0.31 [0.08, 1.19] | 2017 |
| Wu, T.2017 | 10 | 67 | 21 | 68 | 4.1% | 0.39 [0.17, 0.92] | 2017 |
| Zhang, H.2018 | 13 | 30 | 22 | 30 | 2.9% | 0.28 [0.09, 0.82] | 2018 |
| Zhou, D.2018 | 11 | 58 | 13 | 58 | 2.4% | 0.81 [0.33, 1.99] | 2018 |
| Guo, X.2020 | 7 | 51 | 16 | 51 | 3.2% | 0.35 [0.13, 0.94] | 2020 |
| Tan, H.2020 | 51 | 60 | 57 | 60 | 2.0% | 0.30 [0.08, 1.16] | 2020 |
| Chen,C.2020 | 33 | 49 | 43 | 49 | 3.2% | 0.29 [0.10, 0.82] | 2020 |
| Xu,Z.2020 | 2 | 40 | 6 | 40 | 1.3% | 0.30 [0.06, 1.58] | 2020 |
| **Total (95% CI)** | | **1701** | | **1670** | **100.0%** | **0.41 [0.35, 0.49]** | |
| Total events | 626 | | 862 | | | | |

Heterogeneity: Chi² = 26.97, df = 39 (P = 0.93); I² = 0%
Test for overall effect: Z = 10.32 (P < 0.00001)

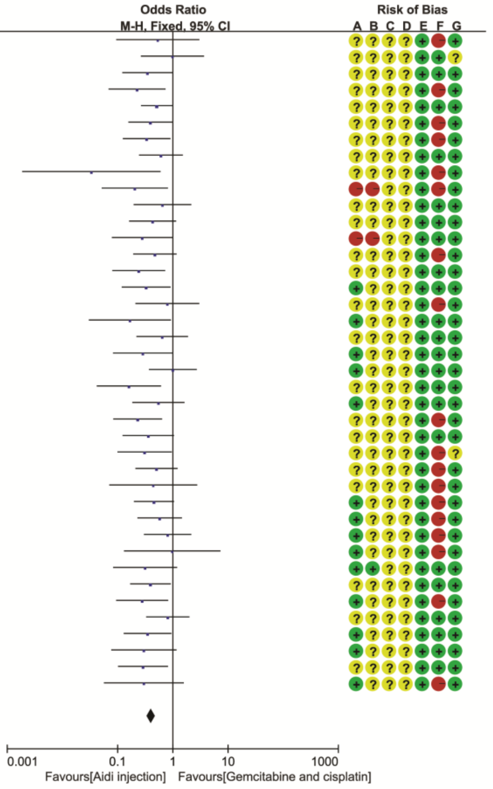


Risk of bias legend
(A) Random sequence generation (selection bias)
(B) Allocation concealment (selection bias)
(C) Blinding of participants and personnel (performance bias)
(D) Blinding of outcome assessment (detection bias)
(E) Incomplete outcome data (attrition bias)
(F) Selective reporting (reporting bias)
(G) Other bias

## Figure S26 The analysis of neutropenia between the two groups

| Study or Subgroup | Aidi injection Events | Aidi injection Total | Gemcitabine and cisplatin Events | Gemcitabine and cisplatin Total | Weight | Odds Ratio M-H, Fixed, 95% CI | Year |
|---|---|---|---|---|---|---|---|
| Feng,X.2008 | 62 | 68 | 62 | 62 | 2.4% | 0.08 [0.00, 1.39] | 2008 |
| Zhang, L.2009 | 4 | 32 | 9 | 31 | 3.2% | 0.35 [0.09, 1.29] | 2009 |
| Wang,Y.2009 | 4 | 32 | 2 | 27 | 0.8% | 1.79 [0.30, 10.60] | 2009 |
| Wen,K.2009 | 22 | 38 | 24 | 38 | 4.0% | 0.80 [0.32, 2.02] | 2009 |
| Hou,A.2010 | 3 | 40 | 8 | 38 | 3.0% | 0.30 [0.07, 1.25] | 2010 |
| Hong,Y.2010 | 12 | 90 | 23 | 70 | 8.9% | 0.31 [0.14, 0.69] | 2010 |
| Wang,Y.2012 | 7 | 36 | 15 | 36 | 4.8% | 0.34 [0.12, 0.97] | 2012 |
| Wang,J.2012 | 4 | 25 | 7 | 24 | 2.4% | 0.46 [0.12, 1.85] | 2012 |
| Xu,Y.2012 | 14 | 33 | 24 | 33 | 5.5% | 0.28 [0.10, 0.77] | 2012 |
| Zhang,Y. 2012 | 6 | 41 | 11 | 42 | 3.7% | 0.48 [0.16, 1.46] | 2012 |
| Xu, H.2013 | 4 | 38 | 10 | 42 | 3.4% | 0.38 [0.11, 1.32] | 2013 |
| Cheng, B.2014 | 9 | 49 | 14 | 52 | 4.4% | 0.61 [0.24, 1.58] | 2014 |
| Li, J.2014 | 3 | 27 | 2 | 27 | 0.7% | 1.56 [0.24, 10.19] | 2014 |
| Liu,Y.2014 | 26 | 43 | 28 | 43 | 4.4% | 0.82 [0.34, 1.97] | 2014 |
| Han, R.2015 | 2 | 36 | 4 | 36 | 1.5% | 0.47 [0.08, 2.75] | 2015 |
| Zhu, H.2015 | 5 | 21 | 15 | 21 | 4.5% | 0.13 [0.03, 0.50] | 2015 |
| Zhao, S.2015 | 2 | 43 | 4 | 43 | 1.5% | 0.48 [0.08, 2.75] | 2015 |
| Ning, S.2015 | 7 | 31 | 9 | 31 | 2.8% | 0.71 [0.23, 2.24] | 2015 |
| Fang, L.2016 | 7 | 45 | 15 | 45 | 5.0% | 0.37 [0.13, 1.02] | 2016 |
| Ma, Y. 2016 | 4 | 33 | 11 | 35 | 3.7% | 0.30 [0.08, 1.07] | 2016 |
| Li, J.2016 | 6 | 47 | 10 | 47 | 3.5% | 0.54 [0.18, 1.64] | 2016 |
| Huang, W.2017 | 5 | 39 | 7 | 40 | 2.4% | 0.69 [0.20, 2.40] | 2017 |
| Su, S.2017 | 1 | 40 | 2 | 39 | 0.8% | 0.47 [0.04, 5.45] | 2017 |
| Zhou, D.2018 | 16 | 58 | 20 | 58 | 5.7% | 0.72 [0.33, 1.60] | 2018 |
| Geng,K.2020 | 12 | 45 | 10 | 45 | 2.9% | 1.27 [0.49, 3.34] | 2020 |
| Guo, X.2020 | 4 | 51 | 12 | 51 | 4.4% | 0.28 [0.08, 0.93] | 2020 |
| Tan, H.2020 | 34 | 60 | 46 | 60 | 7.9% | 0.40 [0.18, 0.87] | 2020 |
| Xu,Z.2020 | 1 | 40 | 5 | 40 | 1.9% | 0.18 [0.02, 1.61] | 2020 |
| **Total (95% CI)** | | **1181** | | **1156** | **100.0%** | **0.48 [0.39, 0.59]** | |
| Total events | 286 | | 409 | | | | |

Heterogeneity: Chi² = 23.48, df = 27 (P = 0.66); I² = 0%
Test for overall effect: Z = 6.85 (P < 0.00001)

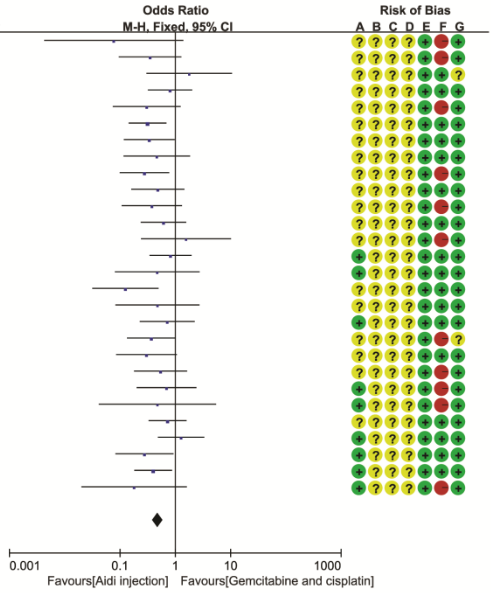


Risk of bias legend
(**A**) Random sequence generation (selection bias)
(**B**) Allocation concealment (selection bias)
(**C**) Blinding of participants and personnel (performance bias)
(**D**) Blinding of outcome assessment (detection bias)
(**E**) Incomplete outcome data (attrition bias)
(**F**) Selective reporting (reporting bias)
(**G**) Other bias

**Figure S27 The analysis of thrombocytopenia between the two groups**

| Study or Subgroup | Aidi injection Events | Aidi injection Total | Gemcitabine and cisplatin Events | Gemcitabine and cisplatin Total | Weight | Odds Ratio M-H, Fixed, 95% CI | Year |
|---|---|---|---|---|---|---|---|
| Wang,Y.2009 | 5 | 32 | 5 | 27 | 4.5% | 0.81 [0.21, 3.18] | 2009 |
| Cheng, B.2014 | 44 | 49 | 43 | 52 | 4.2% | 1.84 [0.57, 5.94] | 2014 |
| Li, J.2014 | 1 | 27 | 4 | 27 | 3.8% | 0.22 [0.02, 2.12] | 2014 |
| Li, X.2015 | 15 | 20 | 16 | 20 | 3.9% | 0.75 [0.17, 3.33] | 2015 |
| Zhao, S.2015 | 0 | 43 | 1 | 43 | 1.5% | 0.33 [0.01, 8.22] | 2015 |
| Li, J.-c.2016 | 7 | 35 | 16 | 35 | 12.6% | 0.30 [0.10, 0.86] | 2016 |
| Ma, M.2017 | 10 | 42 | 14 | 42 | 10.5% | 0.63 [0.24, 1.63] | 2017 |
| Wu, T.2017 | 6 | 67 | 15 | 68 | 13.3% | 0.35 [0.13, 0.96] | 2017 |
| Wu, Y.2017 | 20 | 109 | 23 | 109 | 18.5% | 0.84 [0.43, 1.64] | 2017 |
| Zhang, H.2018 | 7 | 40 | 15 | 40 | 12.2% | 0.35 [0.13, 1.00] | 2018 |
| Tan, H.2020 | 41 | 60 | 48 | 60 | 15.0% | 0.54 [0.23, 1.24] | 2020 |
| **Total (95% CI)** | | **524** | | **523** | **100.0%** | **0.59 [0.43, 0.80]** | |
| Total events | 156 | | 200 | | | | |

Heterogeneity: Chi² = 9.52, df = 10 (P = 0.48); I² = 0%
Test for overall effect: Z = 3.32 (P = 0.0009)

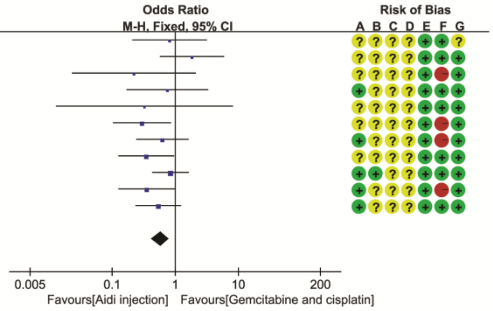


Risk of bias legend
(**A**) Random sequence generation (selection bias)
(**B**) Allocation concealment (selection bias)
(**C**) Blinding of participants and personnel (performance bias)
(**D**) Blinding of outcome assessment (detection bias)
(**E**) Incomplete outcome data (attrition bias)
(**F**) Selective reporting (reporting bias)
(**G**) Other bias

**Figure S28 The analysis of anemia between the two groups**

| Study or Subgroup | Aidi injection Events | Aidi injection Total | Gemcitabine and cisplatin Events | Gemcitabine and cisplatin Total | Weight | Odds Ratio M-H, Fixed, 95% CI | Year |
|---|---|---|---|---|---|---|---|
| Zou,Y.2006 | 32 | 42 | 29 | 39 | 1.2% | 1.10 [0.40, 3.03] | 2006 |
| Feng,X.2008 | 55 | 68 | 54 | 62 | 1.9% | 0.63 [0.24, 1.63] | 2008 |
| Song,Z.2009 | 10 | 30 | 11 | 30 | 1.3% | 0.86 [0.30, 2.50] | 2009 |
| Wang,Y.2009 | 5 | 32 | 5 | 27 | 0.8% | 0.81 [0.21, 3.18] | 2009 |
| Lv,D.2009 | 10 | 30 | 19 | 30 | 2.2% | 0.29 [0.10, 0.84] | 2009 |
| Wen,K.2009 | 20 | 38 | 29 | 38 | 2.4% | 0.34 [0.13, 0.92] | 2009 |
| Zhang, L.2009 | 11 | 32 | 20 | 31 | 2.3% | 0.29 [0.10, 0.81] | 2009 |
| Hou,A.2010 | 5 | 40 | 12 | 38 | 1.8% | 0.31 [0.10, 0.99] | 2010 |
| Li,Z.2010 | 16 | 36 | 20 | 36 | 1.9% | 0.64 [0.25, 1.62] | 2010 |
| Liu,Y.2010 | 7 | 32 | 14 | 32 | 1.9% | 0.36 [0.12, 1.07] | 2010 |
| Hong,Y.2010 | 20 | 90 | 29 | 70 | 4.4% | 0.40 [0.20, 0.80] | 2010 |
| Shi,X.2010 | 12 | 28 | 20 | 28 | 2.0% | 0.30 [0.10, 0.91] | 2010 |
| Lu,Z.2011 | 10 | 34 | 19 | 34 | 2.3% | 0.33 [0.12, 0.90] | 2011 |
| He,W.2011 | 12 | 29 | 13 | 23 | 1.5% | 0.54 [0.18, 1.64] | 2011 |
| Sun,J.2012 | 28 | 34 | 29 | 34 | 0.9% | 0.80 [0.22, 2.94] | 2012 |
| Wang,Y.2012 | 21 | 36 | 28 | 36 | 2.0% | 0.40 [0.14, 1.12] | 2012 |
| Xu,Y.2012 | 7 | 33 | 18 | 33 | 2.4% | 0.22 [0.08, 0.66] | 2012 |
| Wang,J.2012 | 5 | 25 | 8 | 24 | 1.1% | 0.50 [0.14, 1.83] | 2012 |
| Zhang,Y. 2012 | 9 | 41 | 14 | 42 | 1.9% | 0.56 [0.21, 1.50] | 2012 |
| Cai,H.2013 | 6 | 19 | 8 | 19 | 0.9% | 0.63 [0.17, 2.40] | 2013 |
| Xu, H.2013 | 20 | 38 | 30 | 42 | 2.3% | 0.44 [0.18, 1.12] | 2013 |
| Ju,S.2013 | 13 | 34 | 14 | 34 | 1.5% | 0.88 [0.33, 2.34] | 2013 |
| Lai,L.2013 | 20 | 70 | 28 | 70 | 3.4% | 0.60 [0.30, 1.21] | 2013 |
| Li, H.2014 | 18 | 30 | 22 | 30 | 1.5% | 0.55 [0.18, 1.62] | 2014 |
| Cheng, B.2014 | 7 | 49 | 7 | 52 | 1.0% | 1.07 [0.35, 3.31] | 2014 |
| Wen, H.2014 | 23 | 45 | 39 | 45 | 3.3% | 0.16 [0.06, 0.45] | 2014 |
| Liu,Y.2014 | 24 | 43 | 32 | 43 | 2.4% | 0.43 [0.17, 1.08] | 2014 |
| Ning, S.2015 | 12 | 31 | 16 | 31 | 1.7% | 0.59 [0.22, 1.62] | 2015 |
| Li, X.2015 | 15 | 20 | 15 | 20 | 0.6% | 1.00 [0.24, 4.18] | 2015 |
| Zhu, H.2015 | 7 | 21 | 16 | 21 | 1.8% | 0.16 [0.04, 0.60] | 2015 |
| Zhao, S.2015 | 5 | 43 | 10 | 43 | 1.5% | 0.43 [0.13, 1.40] | 2015 |
| Zhang, M.2015 | 32 | 39 | 27 | 32 | 0.9% | 0.85 [0.24, 2.97] | 2015 |
| Zhang, S.2016 | 2 | 19 | 3 | 19 | 0.5% | 0.63 [0.09, 4.26] | 2016 |
| Fang, L.2016 | 6 | 45 | 14 | 45 | 2.1% | 0.34 [0.12, 0.99] | 2016 |
| Li, J.2016 | 15 | 47 | 27 | 47 | 3.2% | 0.35 [0.15, 0.81] | 2016 |
| Ma, Y. 2016 | 5 | 33 | 13 | 35 | 1.8% | 0.30 [0.09, 0.98] | 2016 |
| Li, J.-c.2016 | 7 | 35 | 19 | 35 | 2.6% | 0.21 [0.07, 0.61] | 2016 |
| Su, S.2017 | 4 | 40 | 3 | 39 | 0.5% | 1.33 [0.28, 6.39] | 2017 |
| Zhang, H.2017 | 16 | 52 | 29 | 52 | 3.4% | 0.35 [0.16, 0.79] | 2017 |
| Wu, Y.2017 | 37 | 109 | 63 | 109 | 7.1% | 0.38 [0.22, 0.65] | 2017 |
| Wu, T.2017 | 11 | 67 | 23 | 68 | 3.3% | 0.38 [0.17, 0.87] | 2017 |
| Zhang, H.2018 | 7 | 40 | 18 | 40 | 2.5% | 0.26 [0.09, 0.72] | 2018 |
| Zhou, D.2018 | 29 | 58 | 31 | 58 | 2.7% | 0.87 [0.42, 1.81] | 2018 |
| Su,D.2019 | 5 | 41 | 15 | 41 | 2.3% | 0.24 [0.08, 0.75] | 2019 |
| Geng,K.2020 | 21 | 45 | 30 | 45 | 2.7% | 0.44 [0.19, 1.03] | 2020 |
| Guo, X.2020 | 5 | 51 | 13 | 51 | 2.0% | 0.32 [0.10, 0.97] | 2020 |
| Tan, H.2020 | 47 | 60 | 54 | 60 | 2.0% | 0.40 [0.14, 1.14] | 2020 |
| Chen,C.2020 | 40 | 49 | 42 | 49 | 1.3% | 0.74 [0.25, 2.18] | 2020 |
| Xu,Z.2020 | 2 | 40 | 7 | 40 | 1.1% | 0.25 [0.05, 1.28] | 2020 |
| **Total (95% CI)** | | **2043** | | **2002** | **100.0%** | **0.45 [0.39, 0.51]** | |
| Total events | 756 | | 1059 | | | | |

Heterogeneity: $Chi^2 = 38.98$, df = 48 (P = 0.82); $I^2 = 0\%$
Test for overall effect: Z = 11.21 (P < 0.00001)

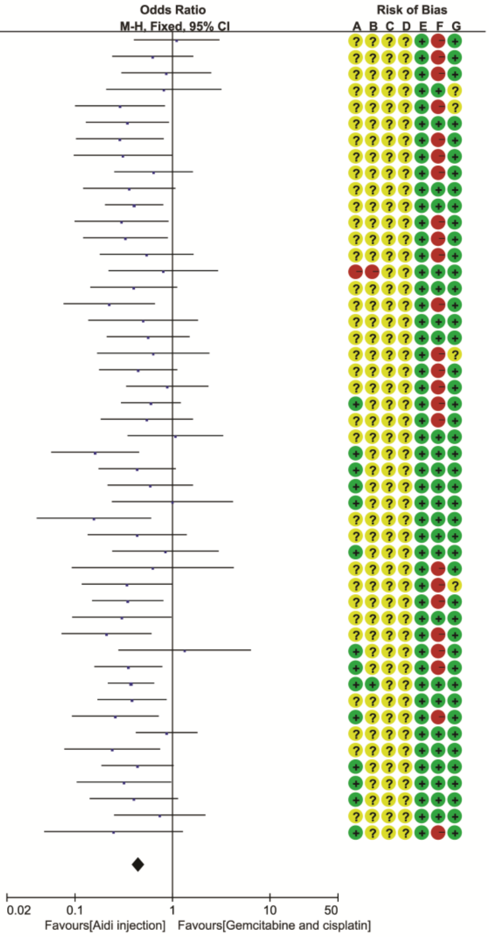


Risk of bias legend
(**A**) Random sequence generation (selection bias)
(**B**) Allocation concealment (selection bias)
(**C**) Blinding of participants and personnel (performance bias)
(**D**) Blinding of outcome assessment (detection bias)
(**E**) Incomplete outcome data (attrition bias)
(**F**) Selective reporting (reporting bias)
(**G**) Other bias

**Figure S29 The analysis of gastrointestinal toxicity between the two groups**

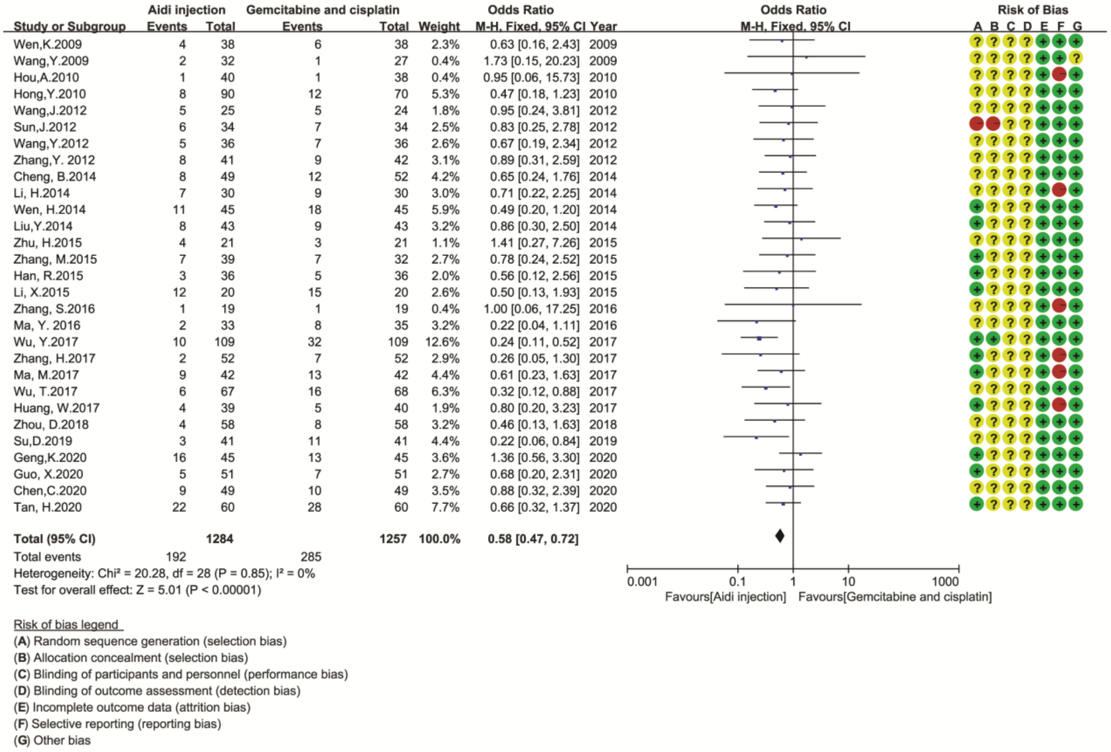


**Figure S30 The analysis of liver toxicity between the two groups**

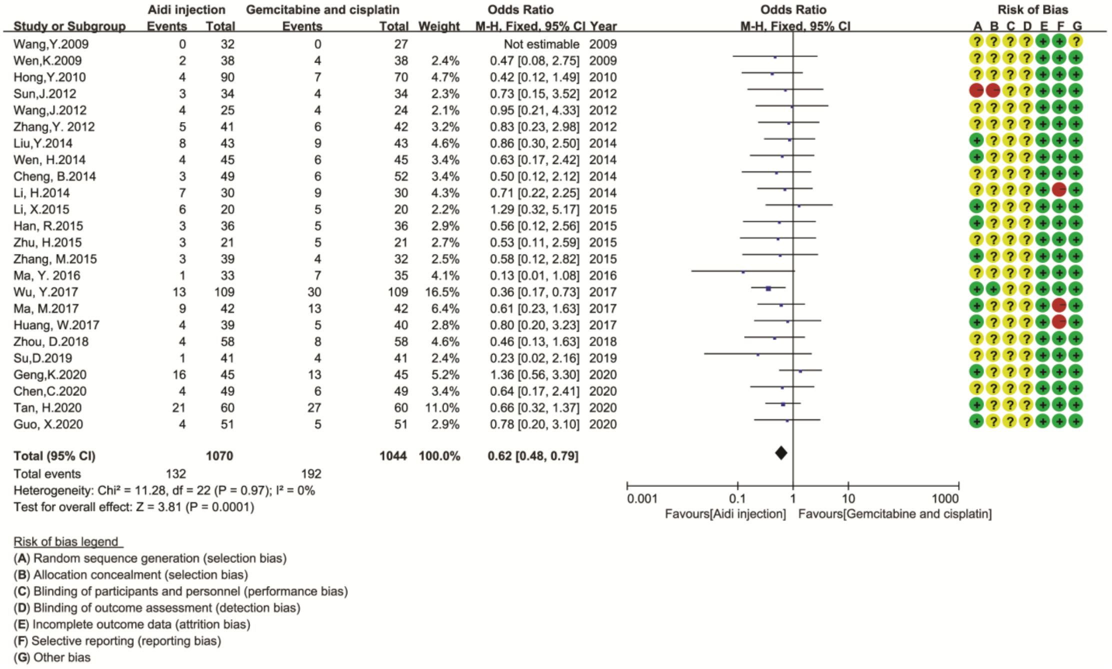


**Figure S31 The analysis of renal toxicity between the two groups**

| Study or Subgroup | Aidi injection Events | Aidi injection Total | Gemcitabine and cisplatin Events | Gemcitabine and cisplatin Total | Weight | Odds Ratio M-H, Random, 95% CI |
|---|---|---|---|---|---|---|
| Liu,Y.2010 | 15 | 32 | 29 | 32 | 35.3% | 0.09 [0.02, 0.36] |
| Song,Z.2009 | 4 | 30 | 3 | 30 | 32.5% | 1.38 [0.28, 6.80] |
| Wang,Y.2009 | 22 | 32 | 25 | 27 | 32.1% | 0.18 [0.03, 0.89] |
| **Total (95% CI)** | | **94** | | **89** | **100.0%** | **0.27 [0.05, 1.37]** |
| Total events | 41 | | 57 | | | |

Heterogeneity: Tau² = 1.42; Chi² = 6.69, df = 2 (P = 0.04); I² = 70%
Test for overall effect: Z = 1.58 (P = 0.11)

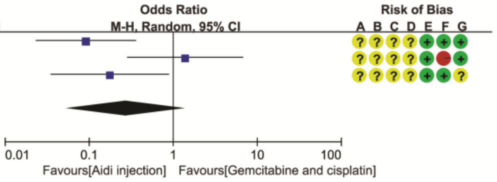


Risk of bias legend
(A) Random sequence generation (selection bias)
(B) Allocation concealment (selection bias)
(C) Blinding of participants and personnel (performance bias)
(D) Blinding of outcome assessment (detection bias)
(E) Incomplete outcome data (attrition bias)
(F) Selective reporting (reporting bias)
(G) Other bias

**Figure S32 The analysis of alopecia between the two groups**

| Study or Subgroup | Aidi injection Events | Aidi injection Total | Gemcitabine and cisplatin Events | Gemcitabine and cisplatin Total | Weight | Odds Ratio M-H, Fixed, 95% CI | Year |
|---|---|---|---|---|---|---|---|
| Song,Z.2009 | 4 | 30 | 4 | 30 | 11.7% | 1.00 [0.23, 4.43] | 2009 |
| Liu,Y.2010 | 1 | 32 | 5 | 32 | 16.3% | 0.17 [0.02, 1.58] | 2010 |
| Wen, H.2014 | 2 | 45 | 4 | 45 | 12.9% | 0.48 [0.08, 2.74] | 2014 |
| Su,D.2019 | 0 | 41 | 2 | 41 | 8.3% | 0.19 [0.01, 4.09] | 2019 |
| Tan, H.2020 | 19 | 60 | 22 | 60 | 50.7% | 0.80 [0.38, 1.70] | 2020 |
| **Total (95% CI)** | | **208** | | **208** | **100.0%** | **0.63 [0.35, 1.12]** | |
| Total events | 26 | | 37 | | | | |

Heterogeneity: Chi² = 2.74, df = 4 (P = 0.60); I² = 0%
Test for overall effect: Z = 1.58 (P = 0.11)

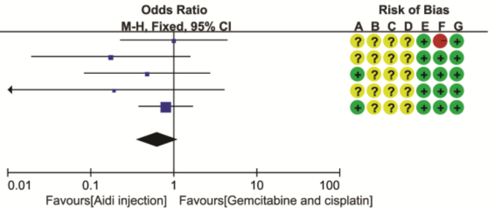


Risk of bias legend
(A) Random sequence generation (selection bias)
(B) Allocation concealment (selection bias)
(C) Blinding of participants and personnel (performance bias)
(D) Blinding of outcome assessment (detection bias)
(E) Incomplete outcome data (attrition bias)
(F) Selective reporting (reporting bias)
(G) Other bias

**Figure S33 The analysis of neurotoxicity between the two groups**

| Study or Subgroup | Aidi injection Events | Aidi injection Total | Gemcitabine and cisplatin Events | Gemcitabine and cisplatin Total | Weight | Odds Ratio M-H, Fixed, 95% CI |
|---|---|---|---|---|---|---|
| Liu,Y.2010 | 0 | 32 | 4 | 32 | 27.6% | 0.10 [0.01, 1.89] |
| Ning, S.2015 | 7 | 31 | 9 | 31 | 43.4% | 0.71 [0.23, 2.24] |
| Zhao, S.2015 | 3 | 43 | 5 | 43 | 29.0% | 0.57 [0.13, 2.55] |
| **Total (95% CI)** | | **106** | | **106** | **100.0%** | **0.50 [0.22, 1.16]** |
| Total events | 10 | | 18 | | | |

Heterogeneity: Chi² = 1.56, df = 2 (P = 0.46); I² = 0%
Test for overall effect: Z = 1.62 (P = 0.11)

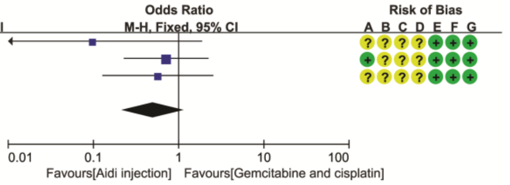


Risk of bias legend
(A) Random sequence generation (selection bias)
(B) Allocation concealment (selection bias)
(C) Blinding of participants and personnel (performance bias)
(D) Blinding of outcome assessment (detection bias)
(E) Incomplete outcome data (attrition bias)
(F) Selective reporting (reporting bias)
(G) Other bias

**Figure S34 The analysis of oral mucositis between the two groups**
