# Supplementary material for: The Optimal Adjuvant Strategy of Aidi Injection With Gemcitabine and Cisplatin in Advanced Non–small Cell Lung Cancer: A Meta-analysis of 70 Randomized Controlled Trials
Source: Front Pharmacol. 2021 May 28;12:582447. doi: 10.3389/fphar.2021.582447 (PMC8194277; doi:10.3389/fphar.2021.582447)
Supplement: Supplementary file 2 [file DataSheet3.pdf]

Supplementary material 6. Subgroup and meta-regression analysis results (Figures S35-106)

Study or Subgroup	Aidi injetion Events	Aidi injetion Total	Gemcitabine and cisplatin Events	Gemcitabine and cisplatin Total	Weight	Odds Ratio M-H, Fixed, 95% CI	Year
**KPS score(≥60)**							
Zou,Y.2006	24	42	13	39	1.4%	2.67 [1.08, 6.58]	2006
Sun,G.2008	17	33	15	30	1.8%	1.06 [0.40, 2.86]	2008
Yang,Q.2008	23	30	11	27	0.6%	4.78 [1.52, 14.98]	2008
Wang,Y.2009	17	32	12	27	1.4%	1.42 [0.51, 3.96]	2009
Song,Z.2009	10	30	9	30	1.4%	1.17 [0.39, 3.47]	2009
Zhang, L.2009	15	32	14	31	1.8%	1.07 [0.40, 2.89]	2009
Lv,D.2009	22	30	11	30	0.7%	4.75 [1.58, 14.25]	2009
Shi,X.2010	13	28	10	28	1.3%	1.56 [0.53, 4.56]	2010
Hou,A.2010	16	40	14	38	2.0%	1.14 [0.46, 2.85]	2010
Hong,Y.2010	57	90	32	70	3.1%	2.05 [1.09, 3.88]	2010
Li,Z.2010	16	36	12	36	1.6%	1.60 [0.62, 4.16]	2010
Liu,Y.2010	16	32	10	32	1.2%	2.20 [0.79, 6.10]	2010
Lu,Z.2011	18	34	17	34	1.9%	1.13 [0.43, 2.91]	2011
Jiang,S.2011	15	32	12	30	1.6%	1.32 [0.48, 3.63]	2011
Fan,S.2011	22	41	12	38	1.4%	2.51 [1.00, 6.29]	2011
He,W.2011	15	29	10	23	1.3%	1.39 [0.46, 4.18]	2011
Wang,J.2012	14	25	10	24	1.1%	1.78 [0.57, 5.53]	2012
Zhang,Y. 2012	21	41	15	42	1.7%	1.89 [0.78, 4.55]	2012
Li, H.2014	16	30	12	30	1.3%	1.71 [0.62, 4.77]	2014
Wen, H.2014	28	45	17	45	1.5%	2.71 [1.16, 6.36]	2014
Liu,Y.2014	18	43	17	43	2.3%	1.10 [0.47, 2.60]	2014
Zhu, H.2015	15	21	7	21	0.5%	5.00 [1.35, 18.55]	2015
Ma, Y. 2016	19	33	19	35	1.9%	1.14 [0.44, 2.98]	2016
Li, J.-c.2016	21	35	15	35	1.4%	2.00 [0.77, 5.18]	2016
Chen, Y.2016	15	30	13	30	1.5%	1.31 [0.47, 3.61]	2016
Li, J.2016	23	47	9	47	1.1%	4.05 [1.61, 10.20]	2016
Ma, M.2017	19	42	12	42	1.6%	2.07 [0.84, 5.10]	2017
Huang, W.2017	17	39	11	40	1.5%	2.04 [0.80, 5.21]	2017
Su,D.2019	30	41	17	41	1.1%	3.85 [1.52, 9.75]	2019
Tan, H.2020	24	60	18	60	2.6%	1.56 [0.73, 3.31]	2020
**Subtotal (95% CI)**		**1123**		**1078**	**45.4%**	**1.87 [1.58, 2.22]**	
Total events	596		406				
Heterogeneity: Chi² = 24.34, df = 29 (P = 0.71); I² = 0%							
Test for overall effect: Z = 7.16 (P < 0.00001)							
**KPS score(≥70)**							
Feng,X.2008	30	68	25	62	3.5%	1.17 [0.58, 2.35]	2008
Wang,Y.2012	16	36	14	36	1.8%	1.26 [0.49, 3.21]	2012
Xu,Y.2012	16	33	16	33	2.0%	1.00 [0.38, 2.63]	2012
Cheng, B.2014	12	49	16	52	2.8%	0.73 [0.30, 1.76]	2014
Zhao, S.2015	19	43	12	43	1.6%	2.05 [0.83, 5.02]	2015
Fang, L.2016	35	45	27	45	1.4%	2.33 [0.93, 5.87]	2016
Lv, W.2018	16	30	15	30	1.7%	1.14 [0.41, 3.15]	2018
Geng,K.2020	27	45	17	45	1.6%	2.47 [1.06, 5.77]	2020
**Subtotal (95% CI)**		**349**		**346**	**16.3%**	**1.40 [1.03, 1.90]**	
Total events	171		142				
Heterogeneity: Chi² = 6.63, df = 7 (P = 0.47); I² = 0%							
Test for overall effect: Z = 2.13 (P = 0.03)							
**KPS score(others)**							
Zhao,H.2008	11	30	6	20	1.1%	1.35 [0.40, 4.53]	2008
Wen,K.2009	16	38	15	38	2.1%	1.12 [0.45, 2.79]	2009
Wu,Q.2011	17	30	11	30	1.1%	2.26 [0.80, 6.36]	2011
Ding,P.2011	9	18	10	22	1.1%	1.20 [0.34, 4.18]	2011
Pei, W.2012	15	40	11	40	1.6%	1.58 [0.62, 4.07]	2012
Sun,J.2012	15	34	13	34	1.7%	1.28 [0.48, 3.36]	2012
Fu, L.2012	19	35	16	35	1.7%	1.41 [0.55, 3.61]	2012
Lai,L.2013	45	70	40	70	3.4%	1.35 [0.68, 2.67]	2013
Ju,S.2013	19	34	12	34	1.3%	2.32 [0.87, 6.16]	2013
Xu, H.2013	19	38	18	42	2.0%	1.33 [0.55, 3.22]	2013
Li, J.2014	11	27	10	27	1.4%	1.17 [0.39, 3.49]	2014
Zhang, M.2015	17	39	12	32	1.8%	1.29 [0.50, 3.35]	2015
Ning, S.2015	19	31	11	31	1.0%	2.88 [1.03, 8.07]	2015
Han, R.2015	14	36	10	36	1.4%	1.65 [0.61, 4.45]	2015
Zhang, X.2016	18	25	8	25	0.5%	5.46 [1.63, 18.36]	2016
Zhang, S.2016	11	19	8	19	0.8%	1.89 [0.52, 6.85]	2016
Su, S.2017	35	40	23	39	0.7%	4.87 [1.57, 15.13]	2017
Zhang, H.2017	42	52	26	52	1.2%	4.20 [1.75, 10.11]	2017
Wu, T.2017	53	67	40	68	2.0%	2.65 [1.24, 5.68]	2017
Zhou, D.2018	30	58	22	58	2.5%	1.75 [0.84, 3.67]	2018
Zhao,J.2019	31	43	22	43	1.5%	2.47 [1.01, 6.04]	2019
Liu, H.2019	19	44	12	44	1.6%	2.03 [0.83, 4.95]	2019
Chen,C.2020	26	49	16	49	1.8%	2.33 [1.03, 5.29]	2020
Xu, Y.2020	16	45	12	45	1.8%	1.52 [0.62, 3.73]	2020
Xu,Z.2020	25	40	14	40	1.2%	3.10 [1.24, 7.71]	2020
**Subtotal (95% CI)**		**982**		**973**	**38.3%**	**1.94 [1.61, 2.33]**	
Total events	552		398				
Heterogeneity: Chi² = 18.65, df = 24 (P = 0.77); I² = 0%							
Test for overall effect: Z = 6.99 (P < 0.00001)							
**Total (95% CI)**		**2454**		**2397**	**100.0%**	**1.82 [1.62, 2.04]**	
Total events	1319		946				
Heterogeneity: Chi² = 52.84, df = 62 (P = 0.79); I² = 0%							
Test for overall effect: Z = 10.08 (P < 0.00001)							
Test for subgroup differences: Chi² = 3.38, df = 2 (P = 0.18), I² = 40.8%							

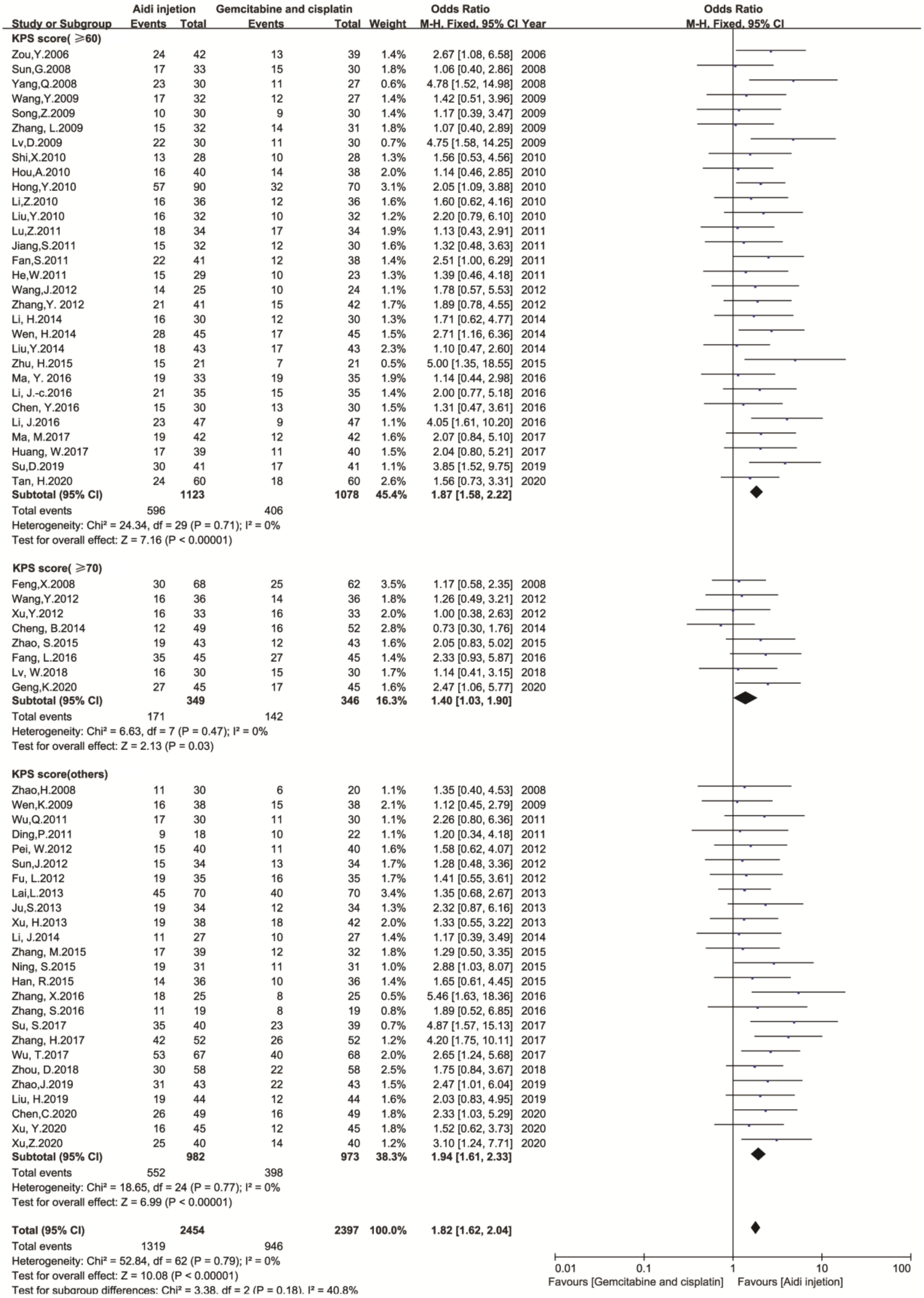

Figure S35 Subgroups analysis of objective response rate by KPS score

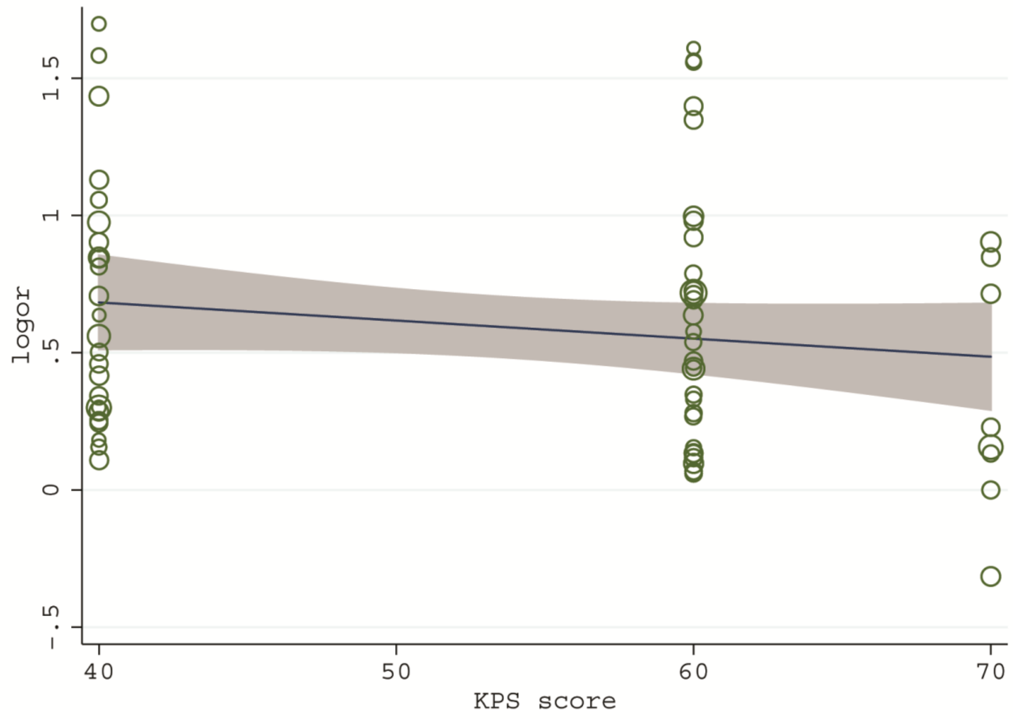

Figure S36 Meta-regression of objective response rate by KPS score

Study or Subgroup	Aidi injetion Events	Aidi injetion Total	Gemcitabine and cisplatin Events	Gemcitabine and cisplatin Total	Weight	Odds Ratio M-H, Fixed, 95% CI	Year
**KPS score(≥60)**							
Zou,Y.2006	35	42	25	39	1.9%	2.80 [0.99, 7.94]	2006
Yang,Q.2008	29	30	23	27	0.4%	5.04 [0.53, 48.26]	2008
Sun,G.2008	31	33	27	30	0.8%	1.72 [0.27, 11.09]	2008
Song,Z.2009	27	30	25	30	1.1%	1.80 [0.39, 8.32]	2009
Lv,D.2009	28	30	23	30	0.7%	4.26 [0.81, 22.53]	2009
Zhang, L.2009	27	32	23	31	1.6%	1.88 [0.54, 6.54]	2009
Wang,Y.2009	29	32	21	27	1.0%	2.76 [0.62, 12.32]	2009
Liu,Y.2010	25	32	22	32	2.2%	1.62 [0.53, 4.99]	2010
Shi,X.2010	27	28	25	28	0.4%	3.24 [0.32, 33.22]	2010
Li,Z.2010	18	36	21	36	4.7%	0.71 [0.28, 1.81]	2010
Hong,Y.2010	78	90	50	70	3.4%	2.60 [1.17, 5.78]	2010
Hou,A.2010	37	40	29	38	1.0%	3.83 [0.95, 15.43]	2010
He,W.2011	26	29	19	23	1.0%	1.82 [0.36, 9.12]	2011
Lu,Z.2011	30	34	28	34	1.5%	1.61 [0.41, 6.30]	2011
Fan,S.2011	35	41	29	38	2.0%	1.81 [0.58, 5.68]	2011
Jiang,S.2011	29	32	25	30	1.1%	1.93 [0.42, 8.91]	2011
Zhang,Y. 2012	34	41	32	42	2.4%	1.52 [0.52, 4.47]	2012
Wang,J.2012	20	25	16	24	1.5%	2.00 [0.55, 7.31]	2012
Wen, H.2014	43	45	36	45	0.7%	5.38 [1.09, 26.49]	2014
Liu,Y.2014	35	43	33	43	2.8%	1.33 [0.47, 3.77]	2014
Li, H.2014	26	30	23	30	1.4%	1.98 [0.51, 7.63]	2014
Zhu, H.2015	19	21	14	21	0.6%	4.75 [0.85, 26.43]	2015
Li, J.2016	45	47	34	47	0.7%	8.60 [1.82, 40.69]	2016
Ma, Y. 2016	28	33	28	35	1.9%	1.40 [0.40, 4.94]	2016
Li, J.-c.2016	31	35	26	35	1.3%	2.68 [0.74, 9.73]	2016
Chen, Y.2016	24	30	18	30	1.6%	2.67 [0.84, 8.46]	2016
Ma, M.2017	37	42	29	42	1.6%	3.32 [1.06, 10.37]	2017
Huang, W.2017	36	39	30	40	1.0%	4.00 [1.01, 15.87]	2017
Su,D.2019	39	41	33	41	0.7%	4.73 [0.94, 23.82]	2019
Tan, H.2020	49	60	39	60	3.2%	2.40 [1.03, 5.57]	2020
**Subtotal (95% CI)**		**1123**		**1078**	**46.0%**	**2.31 [1.84, 2.89]**	
Total events	977		806				
Heterogeneity: Chi² = 17.95, df = 29 (P = 0.95); I² = 0%							
Test for overall effect: Z = 7.25 (P < 0.00001)							
**KPS score(≥70)**							
Feng,X.2008	62	68	56	62	2.3%	1.11 [0.34, 3.63]	2008
Wang,Y.2012	28	36	20	36	2.0%	2.80 [1.01, 7.80]	2012
Xu,Y.2012	30	33	26	33	1.1%	2.69 [0.63, 11.49]	2012
Cheng, B.2014	39	49	42	52	3.7%	0.93 [0.35, 2.47]	2014
Zhao, S.2015	37	43	28	43	1.8%	3.30 [1.14, 9.60]	2015
Fang, L.2016	42	45	34	45	1.0%	4.53 [1.17, 17.55]	2016
Lv, W.2018	24	30	25	30	2.2%	0.80 [0.22, 2.97]	2018
Geng,K.2020	39	45	31	45	1.9%	2.94 [1.01, 8.53]	2020
**Subtotal (95% CI)**		**349**		**346**	**16.0%**	**2.01 [1.36, 2.98]**	
Total events	301		262				
Heterogeneity: Chi² = 8.51, df = 7 (P = 0.29); I² = 18%							
Test for overall effect: Z = 3.47 (P = 0.0005)							
**KPS score(others)**							
Wen,K.2009	32	38	32	38	2.3%	1.00 [0.29, 3.43]	2009
Wu,Q.2011	28	30	26	30	0.8%	2.15 [0.36, 12.76]	2011
Fu, L.2012	32	35	28	35	1.1%	2.67 [0.63, 11.31]	2012
Pei, W.2012	35	40	27	40	1.5%	3.37 [1.07, 10.61]	2012
Sun,J.2012	25	34	25	34	3.0%	1.00 [0.34, 2.94]	2012
Ju,S.2013	32	34	29	34	0.8%	2.76 [0.50, 15.33]	2013
Lai,L.2013	63	70	60	70	2.7%	1.50 [0.54, 4.20]	2013
Xu, H.2013	33	38	34	42	1.9%	1.55 [0.46, 5.24]	2013
Li, J.2014	19	27	16	27	2.1%	1.63 [0.53, 5.04]	2014
Ning, S.2015	26	31	22	31	1.6%	2.13 [0.62, 7.29]	2015
Zhang, M.2015	28	39	23	32	3.2%	1.00 [0.35, 2.82]	2015
Han, R.2015	32	36	27	36	1.3%	2.67 [0.74, 9.63]	2015
Zhang, X.2016	21	25	14	25	1.0%	4.13 [1.09, 15.59]	2016
Zhang, S.2016	14	19	14	19	1.7%	1.00 [0.24, 4.24]	2016
Su, S.2017	37	40	26	39	0.9%	6.17 [1.60, 23.83]	2017
Wu, T.2017	62	67	56	68	1.9%	2.66 [0.88, 8.02]	2017
Zhang, H.2017	50	52	46	52	0.8%	3.26 [0.63, 16.97]	2017
Zhou, D.2018	50	58	38	58	2.4%	3.29 [1.31, 8.27]	2018
Zhao,J.2019	39	43	34	43	1.4%	2.58 [0.73, 9.14]	2019
Liu, H.2019	39	44	30	44	1.5%	3.64 [1.18, 11.23]	2019
Chen,C.2020	44	49	34	49	1.6%	3.88 [1.28, 11.74]	2020
Xu, Y.2020	43	45	33	45	0.7%	7.82 [1.64, 37.36]	2020
Xu,Z.2020	33	40	24	40	1.9%	3.14 [1.12, 8.82]	2020
**Subtotal (95% CI)**		**934**		**931**	**37.9%**	**2.39 [1.87, 3.06]**	
Total events	817		698				
Heterogeneity: Chi² = 17.67, df = 22 (P = 0.73); I² = 0%							
Test for overall effect: Z = 6.91 (P < 0.00001)							
**Total (95% CI)**		**2406**		**2355**	**100.0%**	**2.29 [1.97, 2.67]**	
Total events	2095		1766				
Heterogeneity: Chi² = 44.59, df = 60 (P = 0.93); I² = 0%							
Test for overall effect: Z = 10.58 (P < 0.00001)							
Test for subgroup differences: Chi² = 0.54, df = 2 (P = 0.76), I² = 0%							

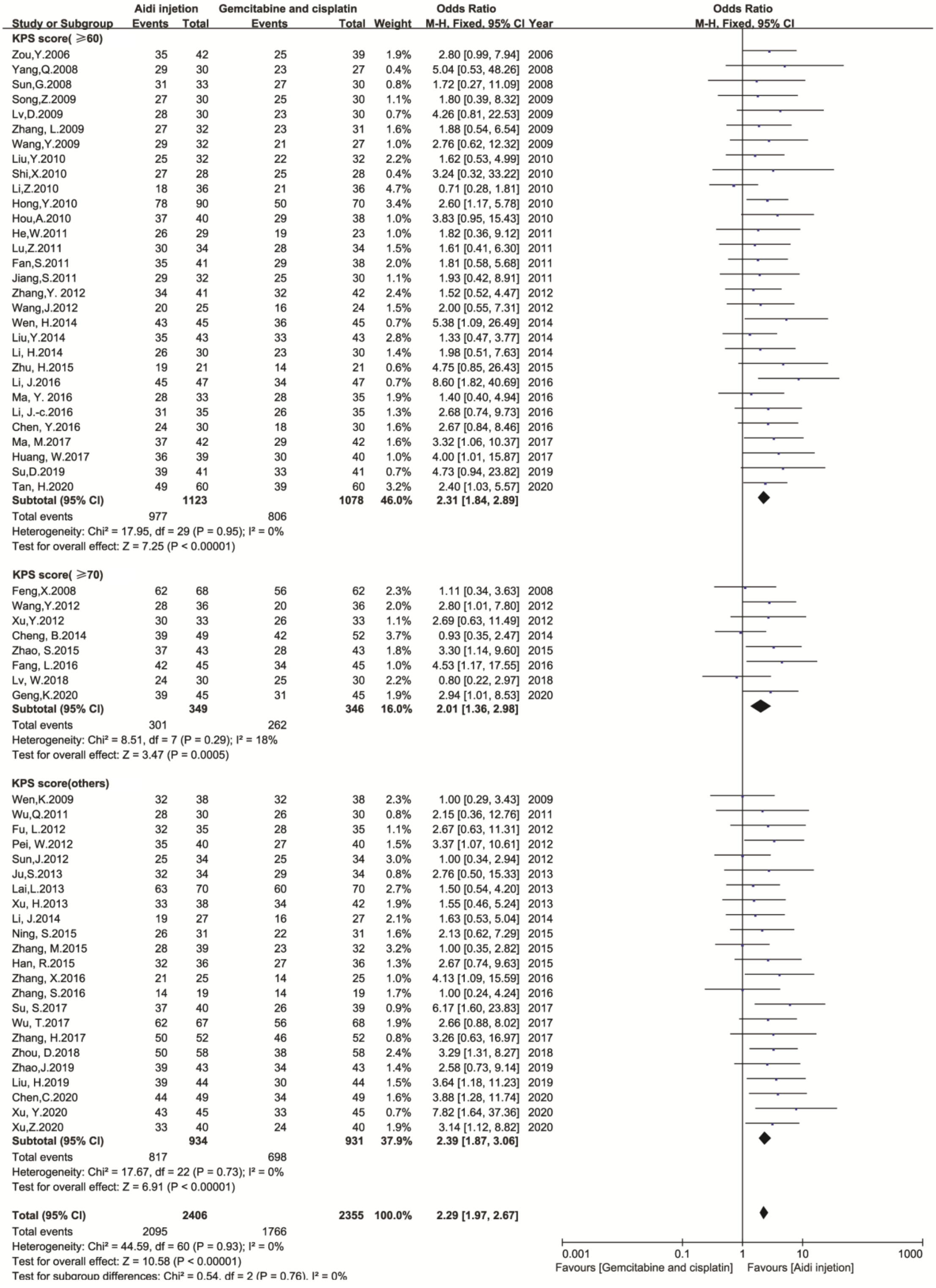

Figure S37 Subgroups analysis of disease control rate by KPS score

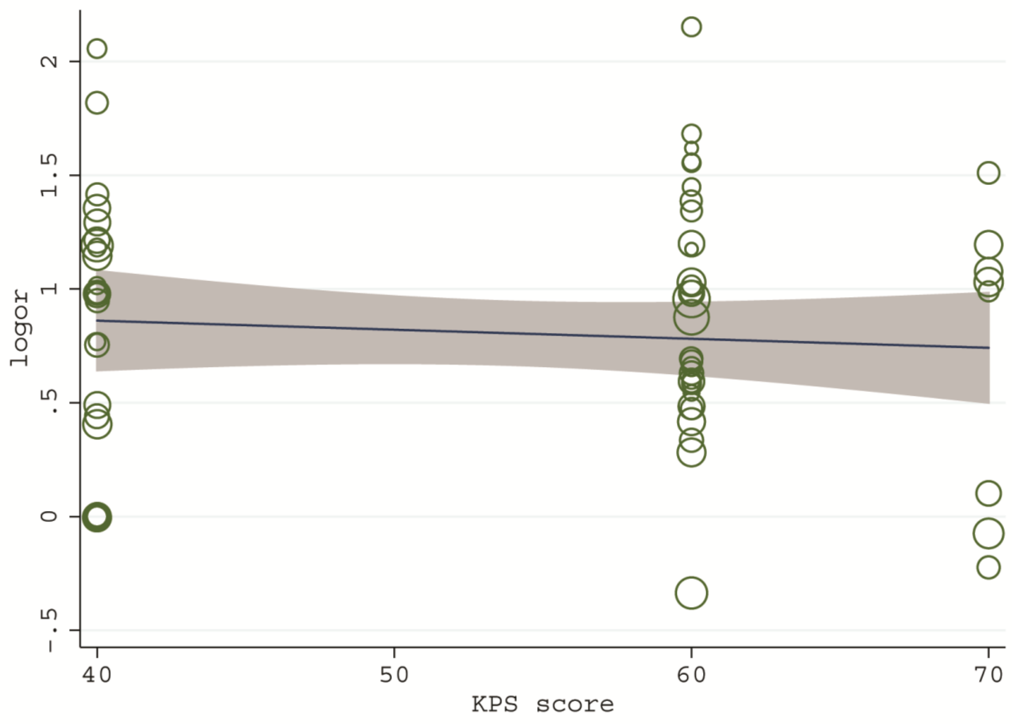

Figure S38 Meta-regression of disease control rate by KPS score

Study or Subgroup	Aidi injection Events	Aidi injection Total	Gemcitabine and cisplatin Events	Gemcitabine and cisplatin Total	Weight	Odds Ratio M-H, Fixed, 95% CI	Year
**KPS score(≥60)**							
Wang,Y.2009	26	32	22	27	1.0%	0.98 [0.26, 3.67]	2009
Zhang, L.2009	5	32	14	31	2.8%	0.22 [0.07, 0.74]	2009
Hong,Y.2010	26	90	31	70	5.7%	0.51 [0.27, 0.98]	2010
Hou,A.2010	14	40	22	38	3.4%	0.39 [0.16, 0.98]	2010
Lu,Z.2011	11	34	20	34	3.1%	0.33 [0.12, 0.90]	2011
Zhang,Y. 2012	12	41	17	42	2.7%	0.61 [0.24, 1.52]	2012
Wang,J.2012	7	25	9	24	1.5%	0.65 [0.19, 2.16]	2012
Liu,Y.2014	27	43	36	43	3.1%	0.33 [0.12, 0.91]	2014
Zhu, H.2015	6	21	15	21	2.5%	0.16 [0.04, 0.61]	2015
Li, X.2015	2	20	8	20	1.7%	0.17 [0.03, 0.92]	2015
Li, J.-c.2016	14	35	26	35	3.6%	0.23 [0.08, 0.64]	2016
Ma, Y. 2016	7	33	15	35	2.7%	0.36 [0.12, 1.05]	2016
Li, J.2016	12	47	19	47	3.3%	0.51 [0.21, 1.21]	2016
Huang, W.2017	10	39	12	40	2.0%	0.80 [0.30, 2.16]	2017
Ma, M.2017	11	42	16	42	2.7%	0.58 [0.23, 1.46]	2017
Tan, H.2020	51	60	57	60	2.0%	0.30 [0.08, 1.16]	2020
**Subtotal (95% CI)**		**634**		**609**	**43.9%**	**0.42 [0.33, 0.54]**	
Total events	241		339				
Heterogeneity: Chi² = 11.65, df = 15 (P = 0.70); I² = 0%							
Test for overall effect: Z = 6.72 (P < 0.00001)							
**KPS score(≥70)**							
Feng,X.2008	64	68	60	62	0.9%	0.53 [0.09, 3.02]	2008
Wang,Y.2012	19	36	26	36	2.8%	0.43 [0.16, 1.15]	2012
Xu,Y.2012	23	33	33	33	2.4%	0.03 [0.00, 0.60]	2012
Cheng, B.2014	34	49	47	52	3.2%	0.24 [0.08, 0.73]	2014
Zhao, S.2015	7	43	10	43	1.9%	0.64 [0.22, 1.88]	2015
Fang, L.2016	5	45	13	45	2.7%	0.31 [0.10, 0.95]	2016
**Subtotal (95% CI)**		**274**		**271**	**13.9%**	**0.33 [0.20, 0.54]**	
Total events	152		189				
Heterogeneity: Chi² = 4.79, df = 5 (P = 0.44); I² = 0%							
Test for overall effect: Z = 4.47 (P < 0.00001)							
**KPS score(others)**							
Wen,K.2009	23	38	31	38	2.8%	0.35 [0.12, 0.99]	2009
Sun,J.2012	23	34	30	34	2.2%	0.28 [0.08, 0.99]	2012
Fu, L.2012	24	35	32	35	2.3%	0.20 [0.05, 0.81]	2012
Xu, H.2013	13	38	22	42	3.2%	0.47 [0.19, 1.17]	2013
Li, J.2014	5	27	6	27	1.1%	0.80 [0.21, 3.00]	2014
Zhang, M.2015	26	39	28	32	2.4%	0.29 [0.08, 0.99]	2015
Ning, S.2015	16	31	16	31	1.8%	1.00 [0.37, 2.71]	2015
Han, R.2015	7	36	11	36	2.0%	0.55 [0.18, 1.63]	2015
Zhang, S.2016	2	19	4	19	0.8%	0.44 [0.07, 2.76]	2016
Su, S.2017	2	40	2	39	0.4%	0.97 [0.13, 7.28]	2017
Wu, Y.2017	3	109	9	109	2.0%	0.31 [0.08, 1.19]	2017
Zhang, H.2017	13	52	22	52	3.8%	0.45 [0.20, 1.05]	2017
Wu, T.2017	10	67	21	68	4.1%	0.39 [0.17, 0.92]	2017
Zhang, H.2018	13	30	22	30	2.9%	0.28 [0.09, 0.82]	2018
Zhou, D.2018	11	58	13	58	2.4%	0.81 [0.33, 1.99]	2018
Chen,C.2020	33	49	43	49	3.2%	0.29 [0.10, 0.82]	2020
Guo, X.2020	7	51	16	51	3.2%	0.35 [0.13, 0.94]	2020
Xu,Z.2020	2	40	6	40	1.3%	0.30 [0.06, 1.58]	2020
**Subtotal (95% CI)**		**793**		**790**	**42.2%**	**0.43 [0.33, 0.55]**	
Total events	233		334				
Heterogeneity: Chi² = 10.32, df = 17 (P = 0.89); I² = 0%							
Test for overall effect: Z = 6.46 (P < 0.00001)							
**Total (95% CI)**		**1701**		**1670**	**100.0%**	**0.41 [0.35, 0.49]**	
Total events	626		862				
Heterogeneity: Chi² = 26.97, df = 39 (P = 0.93); I² = 0%							
Test for overall effect: Z = 10.32 (P < 0.00001)							
Test for subgroup differences: Chi² = 0.92, df = 2 (P = 0.63), I² = 0%							

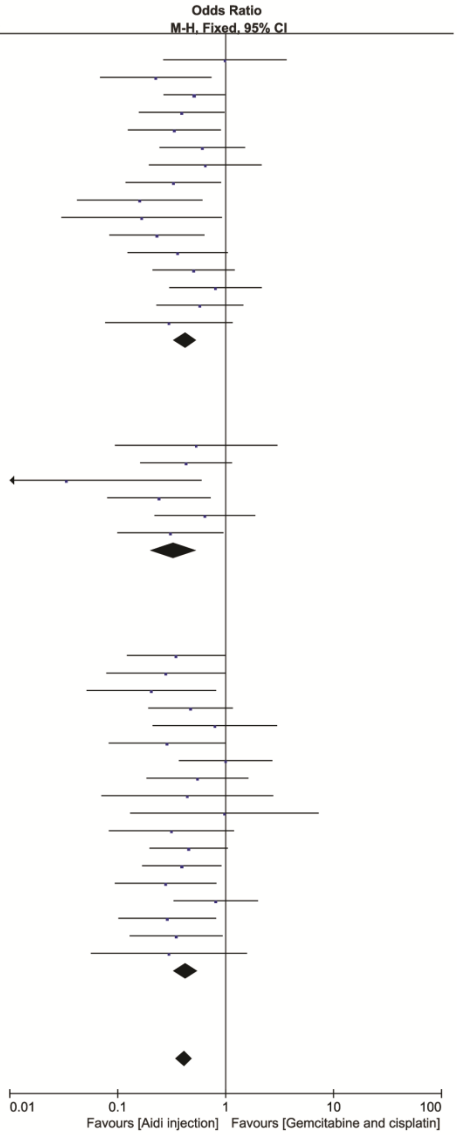

Figure S39 Subgroups analysis of neutropenia by KPS score

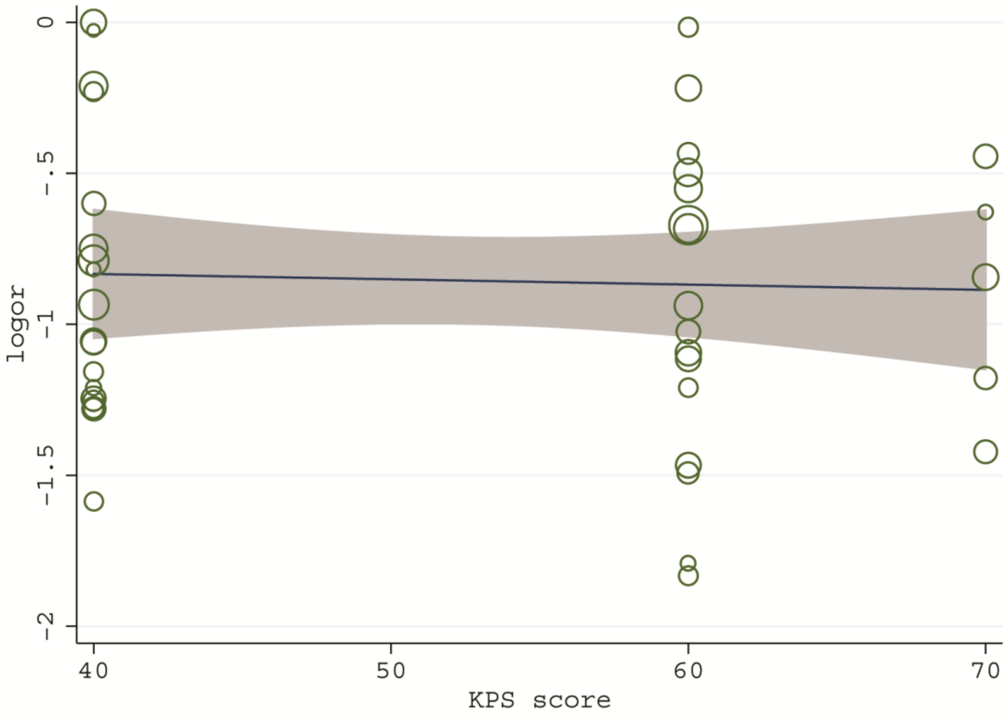

Figure S40 Meta-regression of neutropenia by KPS score

Study or Subgroup	Aidi injetion Events	Aidi injetion Total	Gemcitabine and cisplatin Events	Gemcitabine and cisplatin Total	Weight	Odds Ratio M-H, Fixed, 95% CI	Year
**KPS score(≥60)**							
Zou,Y.2006	32	42	29	39	1.2%	1.10 [0.40, 3.03]	2006
Song,Z.2009	10	30	11	30	1.3%	0.86 [0.30, 2.50]	2009
Zhang, L.2009	11	32	20	31	2.3%	0.29 [0.10, 0.81]	2009
Wang,Y.2009	5	32	5	27	0.8%	0.81 [0.21, 3.18]	2009
Lv,D.2009	10	30	19	30	2.2%	0.29 [0.10, 0.84]	2009
Shi,X.2010	12	28	20	28	2.0%	0.30 [0.10, 0.91]	2010
Hong,Y.2010	20	90	29	70	4.4%	0.40 [0.20, 0.80]	2010
Liu,Y.2010	7	32	14	32	1.9%	0.36 [0.12, 1.07]	2010
Li,Z.2010	16	36	20	36	1.9%	0.64 [0.25, 1.62]	2010
Hou,A.2010	5	40	12	38	1.8%	0.31 [0.10, 0.99]	2010
Lu,Z.2011	10	34	19	34	2.3%	0.33 [0.12, 0.90]	2011
He,W.2011	12	29	13	23	1.5%	0.54 [0.18, 1.64]	2011
Wang,J.2012	5	25	8	24	1.1%	0.50 [0.14, 1.83]	2012
Zhang,Y. 2012	9	41	14	42	1.9%	0.56 [0.21, 1.50]	2012
Cai,H.2013	6	19	8	19	0.9%	0.63 [0.17, 2.40]	2013
Wen, H.2014	23	45	39	45	3.3%	0.16 [0.06, 0.45]	2014
Liu,Y.2014	24	43	32	43	2.4%	0.43 [0.17, 1.08]	2014
Li, H.2014	18	30	22	30	1.5%	0.55 [0.18, 1.62]	2014
Li, X.2015	15	20	15	20	0.6%	1.00 [0.24, 4.18]	2015
Zhu, H.2015	7	21	16	21	1.8%	0.16 [0.04, 0.60]	2015
Ma, Y. 2016	5	33	13	35	1.8%	0.30 [0.09, 0.98]	2016
Li, J.2016	15	47	27	47	3.2%	0.35 [0.15, 0.81]	2016
Li, J.-c.2016	7	35	19	35	2.6%	0.21 [0.07, 0.61]	2016
Su,D.2019	5	41	15	41	2.3%	0.24 [0.08, 0.75]	2019
Tan, H.2020	47	60	54	60	2.0%	0.40 [0.14, 1.14]	2020
**Subtotal (95% CI)**		**915**		**880**	**48.9%**	**0.41 [0.33, 0.50]**	
Total events	336		493				
Heterogeneity: Chi² = 19.83, df = 24 (P = 0.71); I² = 0%							
Test for overall effect: Z = 8.55 (P < 0.00001)							
**KPS score(≥70)**							
Feng,X.2008	55	68	54	62	1.9%	0.63 [0.24, 1.63]	2008
Wang,Y.2012	21	36	28	36	2.0%	0.40 [0.14, 1.12]	2012
Xu,Y.2012	7	33	18	33	2.4%	0.22 [0.08, 0.66]	2012
Cheng, B.2014	7	49	7	52	1.0%	1.07 [0.35, 3.31]	2014
Zhao, S.2015	5	43	10	43	1.5%	0.43 [0.13, 1.40]	2015
Fang, L.2016	6	45	14	45	2.1%	0.34 [0.12, 0.99]	2016
Geng,K.2020	21	45	30	45	2.7%	0.44 [0.19, 1.03]	2020
**Subtotal (95% CI)**		**319**		**316**	**13.6%**	**0.45 [0.31, 0.66]**	
Total events	122		161				
Heterogeneity: Chi² = 4.64, df = 6 (P = 0.59); I² = 0%							
Test for overall effect: Z = 4.07 (P < 0.0001)							
**KPS score(others)**							
Wen,K.2009	20	38	29	38	2.4%	0.34 [0.13, 0.92]	2009
Sun,J.2012	28	34	29	34	0.9%	0.80 [0.22, 2.94]	2012
Ju,S.2013	13	34	14	34	1.5%	0.88 [0.33, 2.34]	2013
Xu, H.2013	20	38	30	42	2.3%	0.44 [0.18, 1.12]	2013
Lai,L.2013	20	70	28	70	3.4%	0.60 [0.30, 1.21]	2013
Zhang, M.2015	32	39	27	32	0.9%	0.85 [0.24, 2.97]	2015
Ning, S.2015	12	31	16	31	1.7%	0.59 [0.22, 1.62]	2015
Zhang, S.2016	2	19	3	19	0.5%	0.63 [0.09, 4.26]	2016
Zhang, H.2017	16	52	29	52	3.4%	0.35 [0.16, 0.79]	2017
Wu, T.2017	11	67	23	68	3.3%	0.38 [0.17, 0.87]	2017
Wu, Y.2017	37	109	63	109	7.1%	0.38 [0.22, 0.65]	2017
Su, S.2017	4	40	3	39	0.5%	1.33 [0.28, 6.39]	2017
Zhang, H.2018	29	58	31	58	2.7%	0.87 [0.42, 1.81]	2018
Zhou, D.2018	7	40	18	40	2.5%	0.26 [0.09, 0.72]	2018
Chen,C.2020	40	49	42	49	1.3%	0.74 [0.25, 2.18]	2020
Guo, X.2020	5	51	13	51	2.0%	0.32 [0.10, 0.97]	2020
Xu,Z.2020	2	40	7	40	1.1%	0.25 [0.05, 1.28]	2020
**Subtotal (95% CI)**		**809**		**806**	**37.5%**	**0.50 [0.40, 0.62]**	
Total events	298		405				
Heterogeneity: Chi² = 12.87, df = 16 (P = 0.68); I² = 0%							
Test for overall effect: Z = 6.12 (P < 0.00001)							
**Total (95% CI)**		**2043**		**2002**	**100.0%**	**0.45 [0.39, 0.51]**	
Total events	756		1059				
Heterogeneity: Chi² = 38.98, df = 48 (P = 0.82); I² = 0%							
Test for overall effect: Z = 11.21 (P < 0.00001)							
Test for subgroup differences: Chi² = 1.70, df = 2 (P = 0.43), I² = 0%							

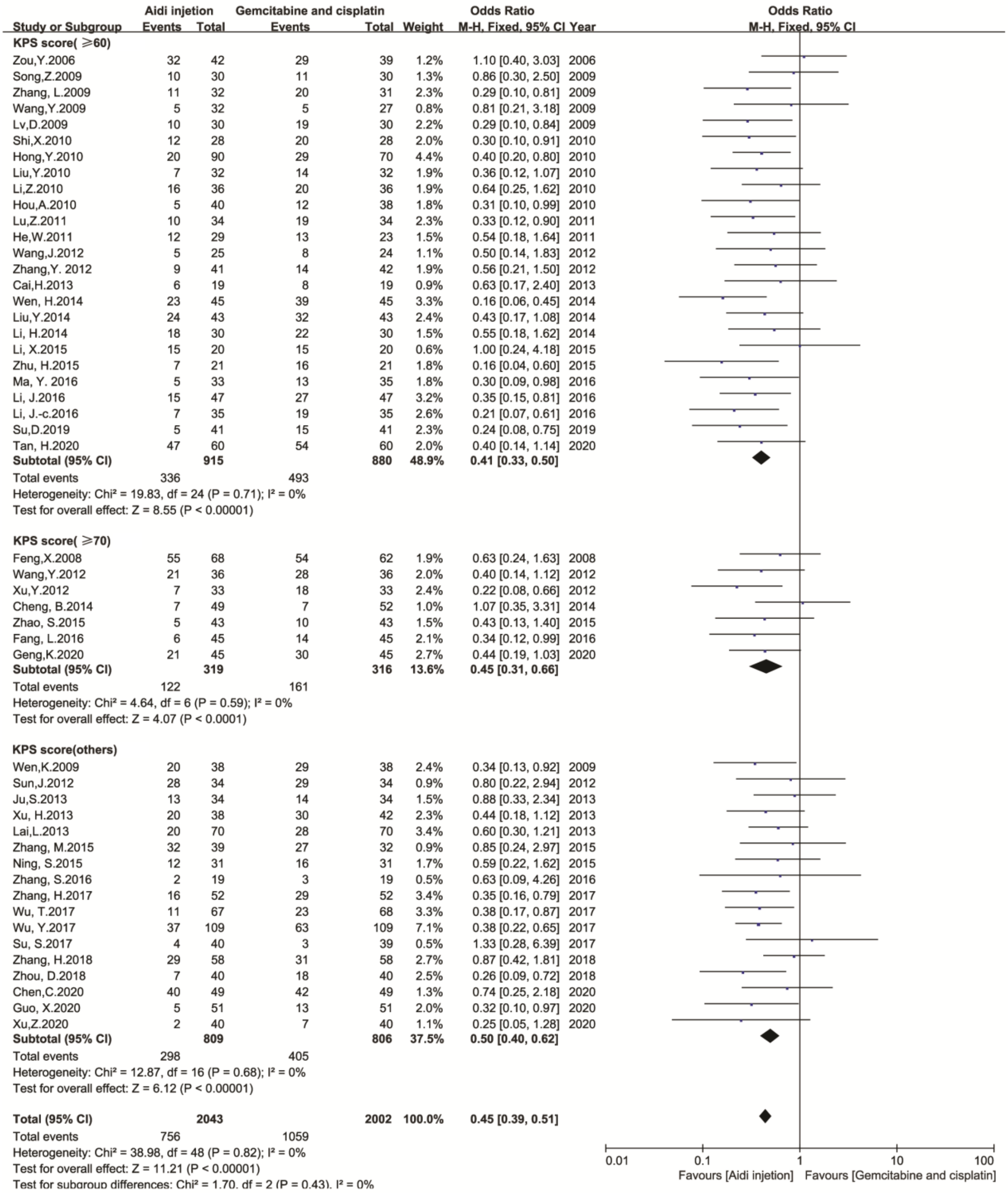

Figure S41 Subgroups analysis of gastrointestinal reaction by KPS score

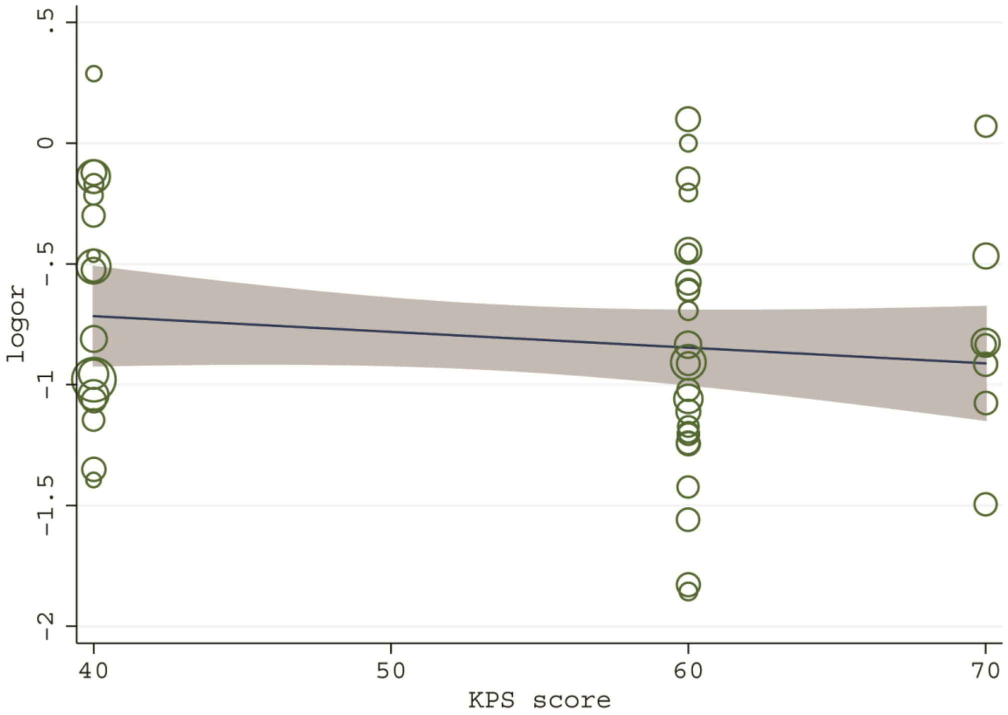

Figure S42 Meta-regression of gastrointestinal reaction by KPS score

Study or Subgroup	Aidi injetion Events	Aidi injetion Total	Gemcitabine and cisplatin Events	Gemcitabine and cisplatin Total	Weight	Odds Ratio M-H, Fixed, 95% CI	Year
**Anticipated survival time(≥3month)**							
Zou,Y.2006	24	42	13	39	1.4%	2.67 [1.08, 6.58]	2006
Zhao,H.2008	11	30	6	20	1.1%	1.35 [0.40, 4.53]	2008
Yang,Q.2008	23	30	11	27	0.6%	4.78 [1.52, 14.98]	2008
Sun,G.2008	17	33	15	30	1.8%	1.06 [0.40, 2.86]	2008
Zhang, L.2009	15	32	14	31	1.8%	1.07 [0.40, 2.89]	2009
Wen,K.2009	16	38	15	38	2.1%	1.12 [0.45, 2.79]	2009
Lv,D.2009	22	30	11	30	0.7%	4.75 [1.58, 14.25]	2009
Song,Z.2009	10	30	9	30	1.4%	1.17 [0.39, 3.47]	2009
Wang,Y.2009	17	32	12	27	1.4%	1.42 [0.51, 3.96]	2009
Hou,A.2010	16	40	14	38	2.0%	1.14 [0.46, 2.85]	2010
Li,Z.2010	16	36	12	36	1.6%	1.60 [0.62, 4.16]	2010
Liu,Y.2010	16	32	10	32	1.2%	2.20 [0.79, 6.10]	2010
Shi,X.2010	13	28	10	28	1.3%	1.56 [0.53, 4.56]	2010
Hong,Y.2010	57	90	32	70	3.1%	2.05 [1.09, 3.88]	2010
He,W.2011	15	29	10	23	1.3%	1.39 [0.46, 4.18]	2011
Fan,S.2011	22	41	12	38	1.4%	2.51 [1.00, 6.29]	2011
Jiang,S.2011	15	32	12	30	1.6%	1.32 [0.48, 3.63]	2011
Ding,P.2011	9	18	10	22	1.1%	1.20 [0.34, 4.18]	2011
Zhang,Y. 2012	21	41	15	42	1.7%	1.89 [0.78, 4.55]	2012
Sun,J.2012	15	34	13	34	1.7%	1.28 [0.48, 3.36]	2012
Fu, L.2012	19	35	16	35	1.7%	1.41 [0.55, 3.61]	2012
Wang,Y.2012	16	36	14	36	1.8%	1.26 [0.49, 3.21]	2012
Wang,J.2012	14	25	10	24	1.1%	1.78 [0.57, 5.53]	2012
Ju,S.2013	19	34	12	34	1.3%	2.32 [0.87, 6.16]	2013
Li, H.2014	16	30	12	30	1.3%	1.71 [0.62, 4.77]	2014
Cheng, B.2014	12	49	16	52	2.8%	0.73 [0.30, 1.76]	2014
Wen, H.2014	28	45	17	45	1.5%	2.71 [1.16, 6.36]	2014
Li, J.2014	11	27	10	27	1.4%	1.17 [0.39, 3.49]	2014
Liu,Y.2014	18	43	17	43	2.3%	1.10 [0.47, 2.60]	2014
Zhao, S.2015	19	43	12	43	1.6%	2.05 [0.83, 5.02]	2015
Zhu, H.2015	15	21	7	21	0.5%	5.00 [1.35, 18.55]	2015
Li, J.2016	23	47	9	47	1.1%	4.05 [1.61, 10.20]	2016
Zhang, X.2016	18	25	8	25	0.5%	5.46 [1.63, 18.36]	2016
Ma, Y. 2016	19	33	19	35	1.9%	1.14 [0.44, 2.98]	2016
Chen, Y.2016	15	30	13	30	1.5%	1.31 [0.47, 3.61]	2016
Su, S.2017	35	40	23	39	0.7%	4.87 [1.57, 15.13]	2017
Lv, W.2018	16	30	15	30	1.7%	1.14 [0.41, 3.15]	2018
Zhou, D.2018	30	58	22	58	2.5%	1.75 [0.84, 3.67]	2018
Su,D.2019	30	41	17	41	1.1%	3.85 [1.52, 9.75]	2019
Zhao,J.2019	31	43	22	43	1.5%	2.47 [1.01, 6.04]	2019
Geng,K.2020	27	45	17	45	1.6%	2.47 [1.06, 5.77]	2020
Tan, H.2020	24	60	18	60	2.6%	1.56 [0.73, 3.31]	2020
**Subtotal (95% CI)**		**1558**		**1508**	**64.1%**	**1.81 [1.56, 2.09]**	
Total events	825		582				
Heterogeneity: Chi² = 39.31, df = 41 (P = 0.55); I² = 0%							
Test for overall effect: Z = 7.96 (P < 0.00001)							
**Anticipated survival time(≥5month)**							
Lu,Z.2011	18	34	17	34	1.9%	1.13 [0.43, 2.91]	2011
Li, J.-c.2016	21	35	15	35	1.4%	2.00 [0.77, 5.18]	2016
Huang, W.2017	17	39	11	40	1.5%	2.04 [0.80, 5.21]	2017
Ma, M.2017	19	42	12	42	1.6%	2.07 [0.84, 5.10]	2017
Xu,Z.2020	25	40	14	40	1.2%	3.10 [1.24, 7.71]	2020
**Subtotal (95% CI)**		**190**		**191**	**7.6%**	**1.98 [1.31, 3.00]**	
Total events	100		69				
Heterogeneity: Chi² = 2.29, df = 4 (P = 0.68); I² = 0%							
Test for overall effect: Z = 3.24 (P = 0.001)							
**Anticipated survival time(Unclear)**							
Feng,X.2008	30	68	25	62	3.5%	1.17 [0.58, 2.35]	2008
Wu,Q.2011	17	30	11	30	1.1%	2.26 [0.80, 6.36]	2011
Xu,Y.2012	16	33	16	33	2.0%	1.00 [0.38, 2.63]	2012
Pei, W.2012	15	40	11	40	1.6%	1.58 [0.62, 4.07]	2012
Xu, H.2013	19	38	18	42	2.0%	1.33 [0.55, 3.22]	2013
Lai,L.2013	45	70	40	70	3.4%	1.35 [0.68, 2.67]	2013
Han, R.2015	14	36	10	36	1.4%	1.65 [0.61, 4.45]	2015
Zhang, M.2015	17	39	12	32	1.8%	1.29 [0.50, 3.35]	2015
Ning, S.2015	19	31	11	31	1.0%	2.88 [1.03, 8.07]	2015
Zhang, S.2016	11	19	8	19	0.8%	1.89 [0.52, 6.85]	2016
Fang, L.2016	35	45	27	45	1.4%	2.33 [0.93, 5.87]	2016
Zhang, H.2017	42	52	26	52	1.2%	4.20 [1.75, 10.11]	2017
Wu, T.2017	53	67	40	68	2.0%	2.65 [1.24, 5.68]	2017
Liu, H.2019	19	44	12	44	1.6%	2.03 [0.83, 4.95]	2019
Chen,C.2020	26	49	16	49	1.8%	2.33 [1.03, 5.29]	2020
Xu, Y.2020	16	45	12	45	1.8%	1.52 [0.62, 3.73]	2020
**Subtotal (95% CI)**		**706**		**698**	**28.4%**	**1.80 [1.45, 2.24]**	
Total events	394		295				
Heterogeneity: Chi² = 11.05, df = 15 (P = 0.75); I² = 0%							
Test for overall effect: Z = 5.27 (P < 0.00001)							
**Total (95% CI)**		**2454**		**2397**	**100.0%**	**1.82 [1.62, 2.04]**	
Total events	1319		946				
Heterogeneity: Chi² = 52.84, df = 62 (P = 0.79); I² = 0%							
Test for overall effect: Z = 10.08 (P < 0.00001)							
Test for subgroup differences: Chi² = 0.18, df = 2 (P = 0.91), I² = 0%							

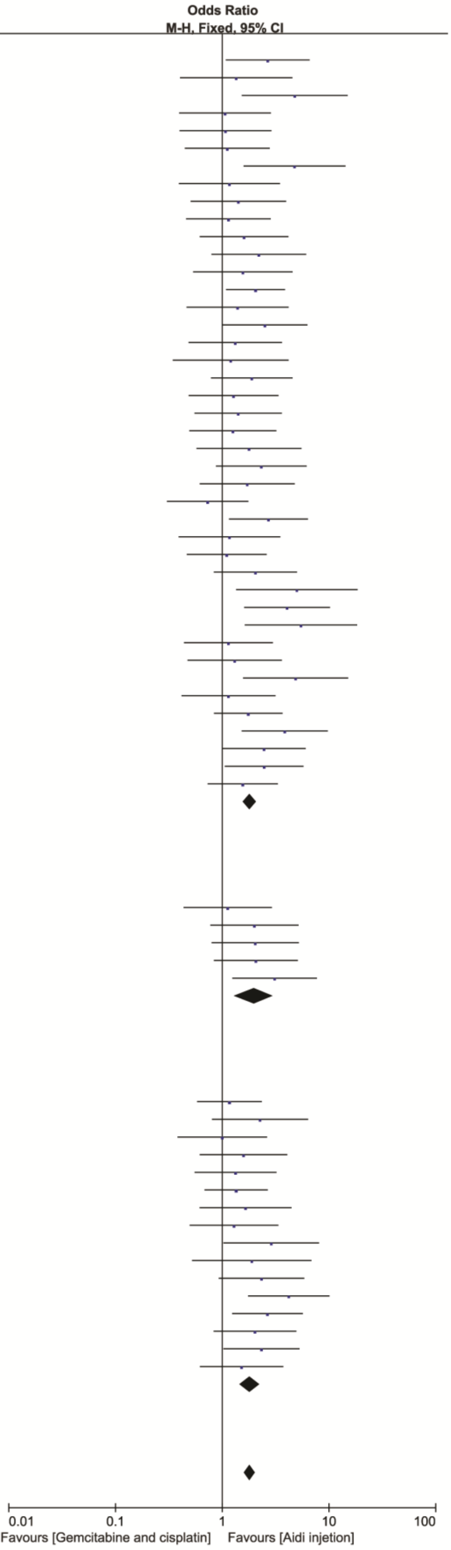

Figure S43 Subgroups analysis of objective response rate by anticipated survival time

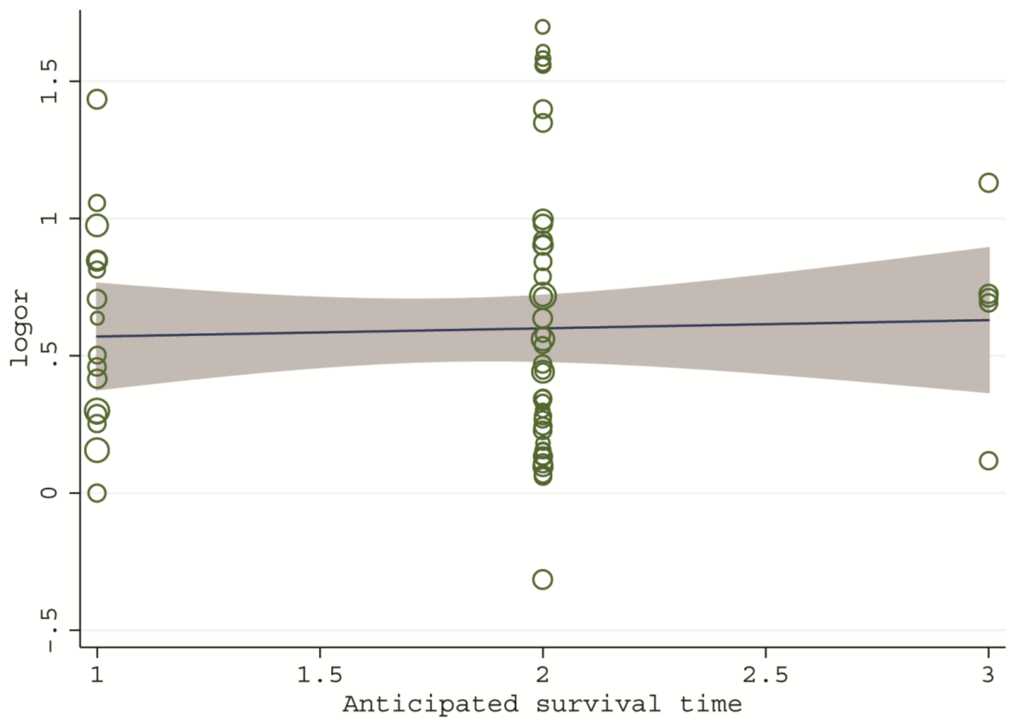

Figure S44 Meta-regression of objective response rate by anticipated survival time

Study or Subgroup	Aidi injetion Events	Aidi injetion Total	Gemcitabine and cisplatin Events	Gemcitabine and cisplatin Total	Weight	Odds Ratio M-H, Fixed, 95% CI	Year
**Anticipated survival time(≥3month)**							
Zou,Y.2006	35	42	25	39	1.9%	2.80 [0.99, 7.94]	2006
Sun,G.2008	31	33	27	30	0.8%	1.72 [0.27, 11.09]	2008
Yang,Q.2008	29	30	23	27	0.4%	5.04 [0.53, 48.26]	2008
Wang,Y.2009	29	32	21	27	1.0%	2.76 [0.62, 12.32]	2009
Song,Z.2009	27	30	25	30	1.1%	1.80 [0.39, 8.32]	2009
Wen,K.2009	32	38	32	38	2.3%	1.00 [0.29, 3.43]	2009
Lv,D.2009	28	30	23	30	0.7%	4.26 [0.81, 22.53]	2009
Zhang, L.2009	27	32	23	31	1.6%	1.88 [0.54, 6.54]	2009
Li,Z.2010	18	36	21	36	4.7%	0.71 [0.28, 1.81]	2010
Shi,X.2010	27	28	25	28	0.4%	3.24 [0.32, 33.22]	2010
Hong,Y.2010	78	90	50	70	3.4%	2.60 [1.17, 5.78]	2010
Liu,Y.2010	25	32	22	32	2.2%	1.62 [0.53, 4.99]	2010
Hou,A.2010	37	40	29	38	1.0%	3.83 [0.95, 15.43]	2010
Jiang,S.2011	29	32	25	30	1.1%	1.93 [0.42, 8.91]	2011
He,W.2011	26	29	19	23	1.0%	1.82 [0.36, 9.12]	2011
Fan,S.2011	35	41	29	38	2.0%	1.81 [0.58, 5.68]	2011
Wang,J.2012	20	25	16	24	1.5%	2.00 [0.55, 7.31]	2012
Fu, L.2012	32	35	28	35	1.1%	2.67 [0.63, 11.31]	2012
Zhang,Y. 2012	34	41	32	42	2.4%	1.52 [0.52, 4.47]	2012
Wang,Y.2012	28	36	20	36	2.0%	2.80 [1.01, 7.80]	2012
Sun,J.2012	25	34	25	34	3.0%	1.00 [0.34, 2.94]	2012
Ju,S.2013	32	34	29	34	0.8%	2.76 [0.50, 15.33]	2013
Li, J.2014	19	27	16	27	2.1%	1.63 [0.53, 5.04]	2014
Wen, H.2014	43	45	36	45	0.7%	5.38 [1.09, 26.49]	2014
Li, H.2014	26	30	23	30	1.4%	1.98 [0.51, 7.63]	2014
Cheng, B.2014	39	49	42	52	3.7%	0.93 [0.35, 2.47]	2014
Liu,Y.2014	35	43	33	43	2.8%	1.33 [0.47, 3.77]	2014
Zhao, S.2015	37	43	28	43	1.8%	3.30 [1.14, 9.60]	2015
Zhu, H.2015	19	21	14	21	0.6%	4.75 [0.85, 26.43]	2015
Li, J.2016	45	47	34	47	0.7%	8.60 [1.82, 40.69]	2016
Chen, Y.2016	24	30	18	30	1.6%	2.67 [0.84, 8.46]	2016
Zhang, X.2016	21	25	14	25	1.0%	4.13 [1.09, 15.59]	2016
Ma, Y. 2016	28	33	28	35	1.9%	1.40 [0.40, 4.94]	2016
Su, S.2017	37	40	26	39	0.9%	6.17 [1.60, 23.83]	2017
Lv, W.2018	24	30	25	30	2.2%	0.80 [0.22, 2.97]	2018
Zhou, D.2018	50	58	38	58	2.4%	3.29 [1.31, 8.27]	2018
Su,D.2019	39	41	33	41	0.7%	4.73 [0.94, 23.82]	2019
Zhao,J.2019	39	43	34	43	1.4%	2.58 [0.73, 9.14]	2019
Tan, H.2020	49	60	39	60	3.2%	2.40 [1.03, 5.57]	2020
Geng,K.2020	39	45	31	45	1.9%	2.94 [1.01, 8.53]	2020
**Subtotal (95% CI)**		**1510**		**1466**	**67.2%**	**2.20 [1.82, 2.66]**	
Total events	1297		1081				
Heterogeneity: Chi² = 30.65, df = 39 (P = 0.83); I² = 0%							
Test for overall effect: Z = 8.20 (P < 0.00001)							
**Anticipated survival time(≥5month)**							
Lu,Z.2011	30	34	28	34	1.5%	1.61 [0.41, 6.30]	2011
Li, J.-c.2016	31	35	26	35	1.3%	2.68 [0.74, 9.73]	2016
Ma, M.2017	37	42	29	42	1.6%	3.32 [1.06, 10.37]	2017
Huang, W.2017	36	39	30	40	1.0%	4.00 [1.01, 15.87]	2017
Xu,Z.2020	33	40	24	40	1.9%	3.14 [1.12, 8.82]	2020
**Subtotal (95% CI)**		**190**		**191**	**7.3%**	**2.90 [1.69, 4.99]**	
Total events	167		137				
Heterogeneity: Chi² = 1.02, df = 4 (P = 0.91); I² = 0%							
Test for overall effect: Z = 3.86 (P = 0.0001)							
**Anticipated survival time(Unclear)**							
Feng,X.2008	62	68	56	62	2.3%	1.11 [0.34, 3.63]	2008
Wu,Q.2011	28	30	26	30	0.8%	2.15 [0.36, 12.76]	2011
Pei, W.2012	35	40	27	40	1.5%	3.37 [1.07, 10.61]	2012
Xu,Y.2012	30	33	26	33	1.1%	2.69 [0.63, 11.49]	2012
Lai,L.2013	63	70	60	70	2.7%	1.50 [0.54, 4.20]	2013
Xu, H.2013	33	38	34	42	1.9%	1.55 [0.46, 5.24]	2013
Zhang, M.2015	28	39	23	32	3.2%	1.00 [0.35, 2.82]	2015
Han, R.2015	32	36	27	36	1.3%	2.67 [0.74, 9.63]	2015
Ning, S.2015	26	31	22	31	1.6%	2.13 [0.62, 7.29]	2015
Fang, L.2016	42	45	34	45	1.0%	4.53 [1.17, 17.55]	2016
Zhang, S.2016	14	19	14	19	1.7%	1.00 [0.24, 4.24]	2016
Zhang, H.2017	50	52	46	52	0.8%	3.26 [0.63, 16.97]	2017
Wu, T.2017	62	67	56	68	1.9%	2.66 [0.88, 8.02]	2017
Liu, H.2019	39	44	30	44	1.5%	3.64 [1.18, 11.23]	2019
Chen,C.2020	44	49	34	49	1.6%	3.88 [1.28, 11.74]	2020
Xu, Y.2020	43	45	33	45	0.7%	7.82 [1.64, 37.36]	2020
**Subtotal (95% CI)**		**706**		**698**	**25.5%**	**2.35 [1.74, 3.18]**	
Total events	631		548				
Heterogeneity: Chi² = 11.91, df = 15 (P = 0.69); I² = 0%							
Test for overall effect: Z = 5.53 (P < 0.00001)							
**Total (95% CI)**		**2406**		**2355**	**100.0%**	**2.29 [1.97, 2.67]**	
Total events	2095		1766				
Heterogeneity: Chi² = 44.59, df = 60 (P = 0.93); I² = 0%							
Test for overall effect: Z = 10.58 (P < 0.00001)							
Test for subgroup differences: Chi² = 0.93, df = 2 (P = 0.63), I² = 0%							

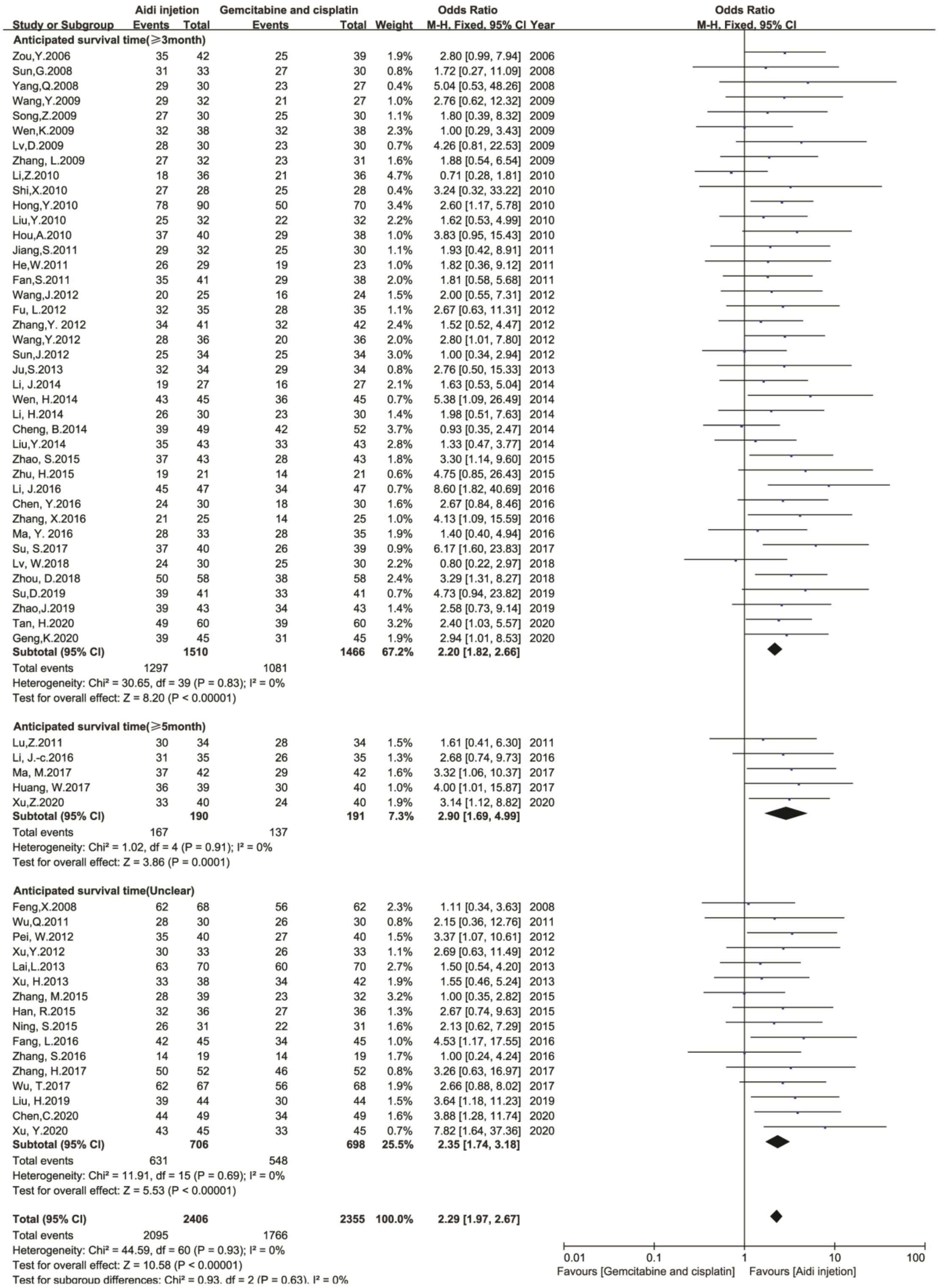

Figure S45 Subgroups analysis of disease control rate by anticipated survival time

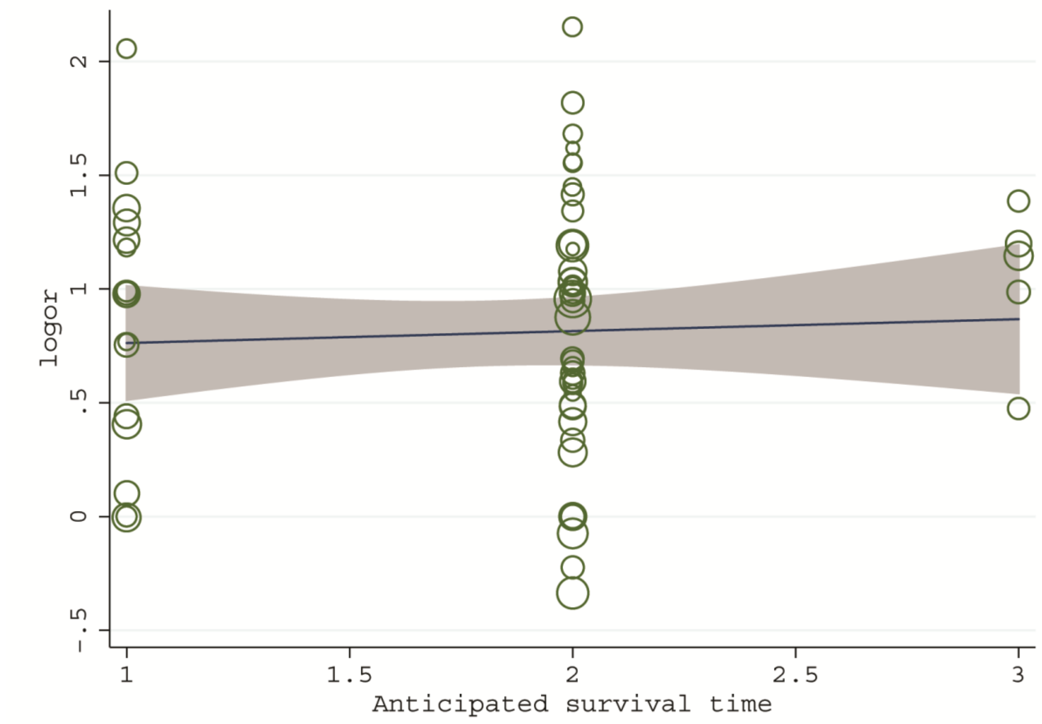

Figure S46 Meta-regression of disease control rate by anticipated survival time

Study or Subgroup	Aidi injection Events	Aidi injection Total	Gemcitabine and cisplatin Events	Gemcitabine and cisplatin Total	Weight	Odds Ratio M-H, Fixed, 95% CI	Year
**Anticipated survival time(≥3month)**							
Wang,Y.2009	26	32	22	27	1.0%	0.98 [0.26, 3.67]	2009
Wen,K.2009	23	38	31	38	2.8%	0.35 [0.12, 0.99]	2009
Zhang, L.2009	5	32	14	31	2.8%	0.22 [0.07, 0.74]	2009
Hong,Y.2010	26	90	31	70	5.7%	0.51 [0.27, 0.98]	2010
Hou,A.2010	14	40	22	38	3.4%	0.39 [0.16, 0.98]	2010
Wang,J.2012	7	25	9	24	1.5%	0.65 [0.19, 2.16]	2012
Zhang,Y. 2012	12	41	17	42	2.7%	0.61 [0.24, 1.52]	2012
Wang,Y.2012	19	36	26	36	2.8%	0.43 [0.16, 1.15]	2012
Fu, L.2012	24	35	32	35	2.3%	0.20 [0.05, 0.81]	2012
Sun,J.2012	23	34	30	34	2.2%	0.28 [0.08, 0.99]	2012
Liu,Y.2014	27	43	36	43	3.1%	0.33 [0.12, 0.91]	2014
Cheng, B.2014	34	49	47	52	3.2%	0.24 [0.08, 0.73]	2014
Li, J.2014	5	27	6	27	1.1%	0.80 [0.21, 3.00]	2014
Li, X.2015	2	20	8	20	1.7%	0.17 [0.03, 0.92]	2015
Zhao, S.2015	7	43	10	43	1.9%	0.64 [0.22, 1.88]	2015
Zhu, H.2015	6	21	15	21	2.5%	0.16 [0.04, 0.61]	2015
Ma, Y. 2016	7	33	15	35	2.7%	0.36 [0.12, 1.05]	2016
Li, J.2016	12	47	19	47	3.3%	0.51 [0.21, 1.21]	2016
Su, S.2017	2	40	2	39	0.4%	0.97 [0.13, 7.28]	2017
Zhou, D.2018	11	58	13	58	2.4%	0.81 [0.33, 1.99]	2018
Guo, X.2020	7	51	16	51	3.2%	0.35 [0.13, 0.94]	2020
Tan, H.2020	51	60	57	60	2.0%	0.30 [0.08, 1.16]	2020
**Subtotal (95% CI)**		**895**		**871**	**55.0%**	**0.42 [0.34, 0.53]**	
Total events	350		478				
Heterogeneity: Chi² = 14.88, df = 21 (P = 0.83); I² = 0%							
Test for overall effect: Z = 7.51 (P < 0.00001)							
**Anticipated survival time(≥5month)**							
Lu,Z.2011	11	34	20	34	3.1%	0.33 [0.12, 0.90]	2011
Li, J.-c.2016	14	35	26	35	3.6%	0.23 [0.08, 0.64]	2016
Huang, W.2017	10	39	12	40	2.0%	0.80 [0.30, 2.16]	2017
Ma, M.2017	11	42	16	42	2.7%	0.58 [0.23, 1.46]	2017
Zhang, H.2018	13	30	22	30	2.9%	0.28 [0.09, 0.82]	2018
Xu,Z.2020	2	40	6	40	1.3%	0.30 [0.06, 1.58]	2020
**Subtotal (95% CI)**		**220**		**221**	**15.7%**	**0.40 [0.26, 0.61]**	
Total events	61		102				
Heterogeneity: Chi² = 4.32, df = 5 (P = 0.50); I² = 0%							
Test for overall effect: Z = 4.22 (P < 0.0001)							
**Anticipated survival time(Unclear)**							
Feng,X.2008	64	68	60	62	0.9%	0.53 [0.09, 3.02]	2008
Xu,Y.2012	23	33	33	33	2.4%	0.03 [0.00, 0.60]	2012
Xu, H.2013	13	38	22	42	3.2%	0.47 [0.19, 1.17]	2013
Han, R.2015	7	36	11	36	2.0%	0.55 [0.18, 1.63]	2015
Ning, S.2015	16	31	16	31	1.8%	1.00 [0.37, 2.71]	2015
Zhang, M.2015	26	39	28	32	2.4%	0.29 [0.08, 0.99]	2015
Zhang, S.2016	2	19	4	19	0.8%	0.44 [0.07, 2.76]	2016
Fang, L.2016	5	45	13	45	2.7%	0.31 [0.10, 0.95]	2016
Zhang, H.2017	13	52	22	52	3.8%	0.45 [0.20, 1.05]	2017
Wu, T.2017	10	67	21	68	4.1%	0.39 [0.17, 0.92]	2017
Wu, Y.2017	3	109	9	109	2.0%	0.31 [0.08, 1.19]	2017
Chen,C.2020	33	49	43	49	3.2%	0.29 [0.10, 0.82]	2020
**Subtotal (95% CI)**		**586**		**578**	**29.3%**	**0.40 [0.29, 0.55]**	
Total events	215		282				
Heterogeneity: Chi² = 7.75, df = 11 (P = 0.74); I² = 0%							
Test for overall effect: Z = 5.69 (P < 0.00001)							
**Total (95% CI)**		**1701**		**1670**	**100.0%**	**0.41 [0.35, 0.49]**	
Total events	626		862				
Heterogeneity: Chi² = 26.97, df = 39 (P = 0.93); I² = 0%							
Test for overall effect: Z = 10.32 (P < 0.00001)							
Test for subgroup differences: Chi² = 0.09, df = 2 (P = 0.95), I² = 0%							

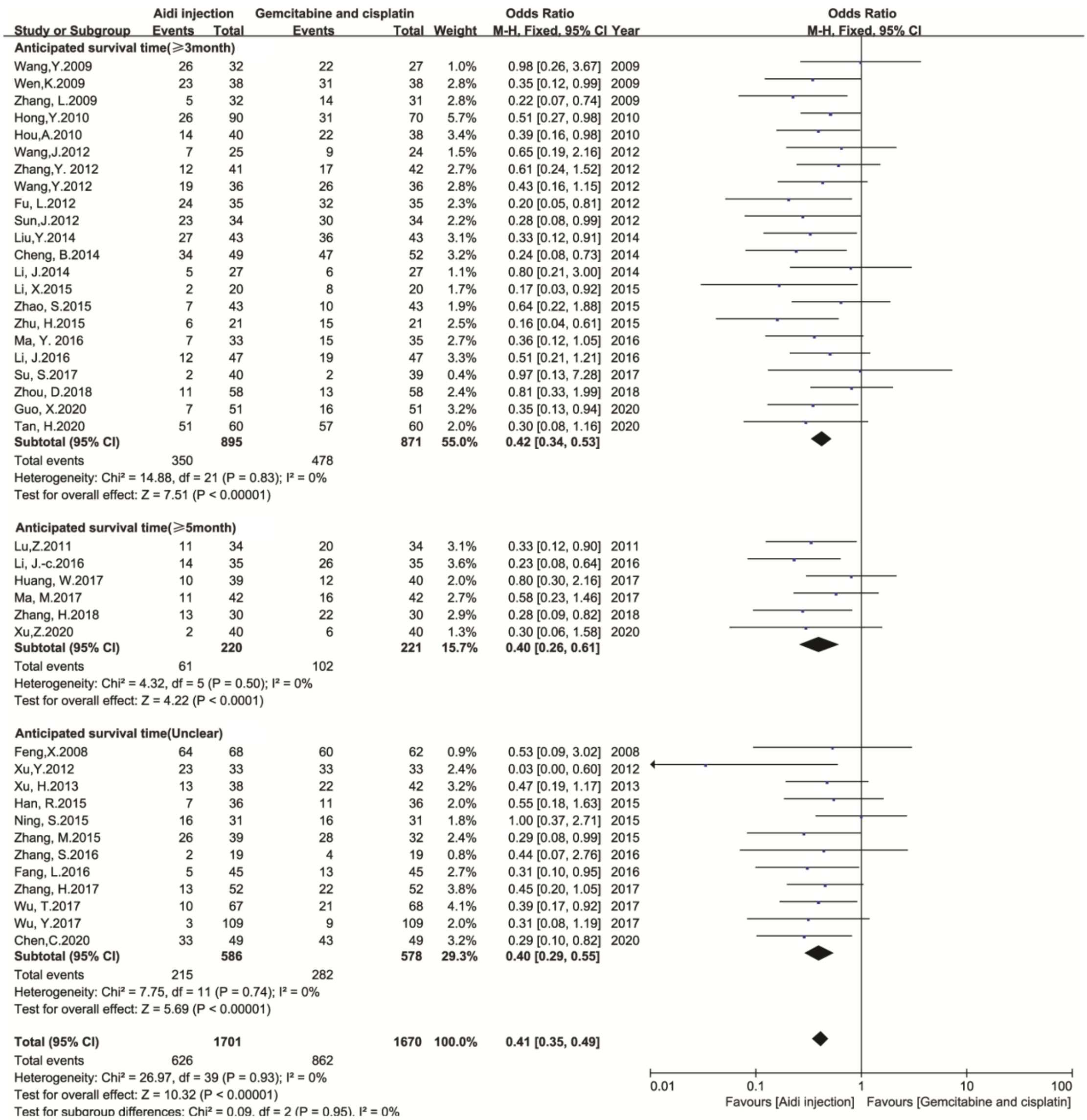

Figure S47 Subgroups analysis of neutropenia by anticipated survival time

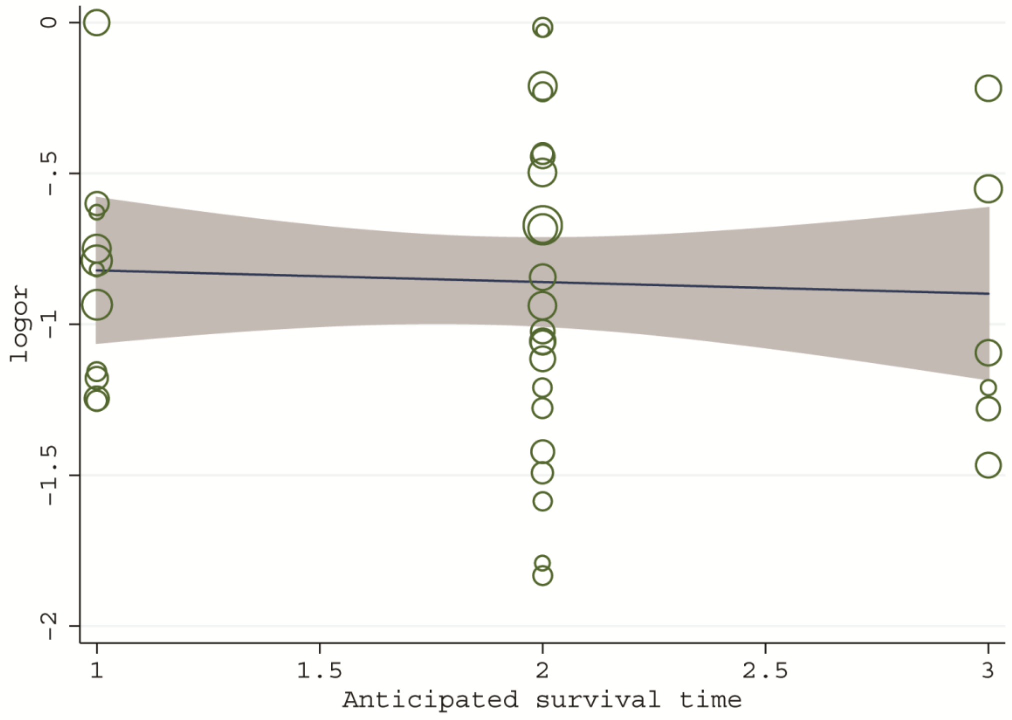

Figure S48 Meta-regression of neutropenia by anticipated survival time

Study or Subgroup	Aidi injetion Events	Aidi injetion Total	Gemcitabine and cisplatin Events	Gemcitabine and cisplatin Total	Weight	Odds Ratio M-H, Fixed, 95% CI	Year
**Anticipated survival time(≥3month)**							
Zou,Y.2006	32	42	29	39	1.2%	1.10 [0.40, 3.03]	2006
Lv,D.2009	10	30	19	30	2.2%	0.29 [0.10, 0.84]	2009
Song,Z.2009	10	30	11	30	1.3%	0.86 [0.30, 2.50]	2009
Zhang, L.2009	11	32	20	31	2.3%	0.29 [0.10, 0.81]	2009
Wang,Y.2009	5	32	5	27	0.8%	0.81 [0.21, 3.18]	2009
Wen,K.2009	20	38	29	38	2.4%	0.34 [0.13, 0.92]	2009
Shi,X.2010	12	28	20	28	2.0%	0.30 [0.10, 0.91]	2010
Hong,Y.2010	20	90	29	70	4.4%	0.40 [0.20, 0.80]	2010
Li,Z.2010	16	36	20	36	1.9%	0.64 [0.25, 1.62]	2010
Liu,Y.2010	7	32	14	32	1.9%	0.36 [0.12, 1.07]	2010
Hou,A.2010	5	40	12	38	1.8%	0.31 [0.10, 0.99]	2010
He,W.2011	12	29	13	23	1.5%	0.54 [0.18, 1.64]	2011
Wang,J.2012	5	25	8	24	1.1%	0.50 [0.14, 1.83]	2012
Sun,J.2012	28	34	29	34	0.9%	0.80 [0.22, 2.94]	2012
Zhang,Y. 2012	9	41	14	42	1.9%	0.56 [0.21, 1.50]	2012
Wang,Y.2012	21	36	28	36	2.0%	0.40 [0.14, 1.12]	2012
Ju,S.2013	13	34	14	34	1.5%	0.88 [0.33, 2.34]	2013
Cheng, B.2014	7	49	7	52	1.0%	1.07 [0.35, 3.31]	2014
Li, H.2014	18	30	22	30	1.5%	0.55 [0.18, 1.62]	2014
Wen, H.2014	23	45	39	45	3.3%	0.16 [0.06, 0.45]	2014
Liu,Y.2014	24	43	32	43	2.4%	0.43 [0.17, 1.08]	2014
Zhao, S.2015	5	43	10	43	1.5%	0.43 [0.13, 1.40]	2015
Li, X.2015	15	20	15	20	0.6%	1.00 [0.24, 4.18]	2015
Zhu, H.2015	7	21	16	21	1.8%	0.16 [0.04, 0.60]	2015
Ma, Y. 2016	5	33	13	35	1.8%	0.30 [0.09, 0.98]	2016
Li, J.2016	15	47	27	47	3.2%	0.35 [0.15, 0.81]	2016
Su, S.2017	4	40	3	39	0.5%	1.33 [0.28, 6.39]	2017
Zhou, D.2018	29	58	31	58	2.7%	0.87 [0.42, 1.81]	2018
Su,D.2019	5	41	15	41	2.3%	0.24 [0.08, 0.75]	2019
Geng,K.2020	21	45	30	45	2.7%	0.44 [0.19, 1.03]	2020
Guo, X.2020	5	51	13	51	2.0%	0.32 [0.10, 0.97]	2020
Tan, H.2020	47	60	54	60	2.0%	0.40 [0.14, 1.14]	2020
**Subtotal (95% CI)**		**1255**		**1222**	**60.1%**	**0.47 [0.39, 0.56]**	
Total events	466		641				
Heterogeneity: Chi² = 28.51, df = 31 (P = 0.59); I² = 0%							
Test for overall effect: Z = 8.31 (P < 0.00001)							
**Anticipated survival time(≥5month)**							
Lu,Z.2011	10	34	19	34	2.3%	0.33 [0.12, 0.90]	2011
Li, J.-c.2016	7	35	19	35	2.6%	0.21 [0.07, 0.61]	2016
Zhang, H.2018	7	40	18	40	2.5%	0.26 [0.09, 0.72]	2018
Xu,Z.2020	2	40	7	40	1.1%	0.25 [0.05, 1.28]	2020
**Subtotal (95% CI)**		**149**		**149**	**8.6%**	**0.26 [0.15, 0.46]**	
Total events	26		63				
Heterogeneity: Chi² = 0.37, df = 3 (P = 0.95); I² = 0%							
Test for overall effect: Z = 4.71 (P < 0.00001)							
**Anticipated survival time(Unclear)**							
Feng,X.2008	55	68	54	62	1.9%	0.63 [0.24, 1.63]	2008
Xu,Y.2012	7	33	18	33	2.4%	0.22 [0.08, 0.66]	2012
Lai,L.2013	20	70	28	70	3.4%	0.60 [0.30, 1.21]	2013
Xu, H.2013	20	38	30	42	2.3%	0.44 [0.18, 1.12]	2013
Cai,H.2013	6	19	8	19	0.9%	0.63 [0.17, 2.40]	2013
Ning, S.2015	12	31	16	31	1.7%	0.59 [0.22, 1.62]	2015
Zhang, M.2015	32	39	27	32	0.9%	0.85 [0.24, 2.97]	2015
Zhang, S.2016	2	19	3	19	0.5%	0.63 [0.09, 4.26]	2016
Fang, L.2016	6	45	14	45	2.1%	0.34 [0.12, 0.99]	2016
Zhang, H.2017	16	52	29	52	3.4%	0.35 [0.16, 0.79]	2017
Wu, Y.2017	37	109	63	109	7.1%	0.38 [0.22, 0.65]	2017
Wu, T.2017	11	67	23	68	3.3%	0.38 [0.17, 0.87]	2017
Chen,C.2020	40	49	42	49	1.3%	0.74 [0.25, 2.18]	2020
**Subtotal (95% CI)**		**639**		**631**	**31.3%**	**0.46 [0.36, 0.59]**	
Total events	264		355				
Heterogeneity: Chi² = 6.31, df = 12 (P = 0.90); I² = 0%							
Test for overall effect: Z = 6.12 (P < 0.00001)							
**Total (95% CI)**		**2043**		**2002**	**100.0%**	**0.45 [0.39, 0.51]**	
Total events	756		1059				
Heterogeneity: Chi² = 38.98, df = 48 (P = 0.82); I² = 0%							
Test for overall effect: Z = 11.21 (P < 0.00001)							
Test for subgroup differences: Chi² = 3.78, df = 2 (P = 0.15), I² = 47.1%							

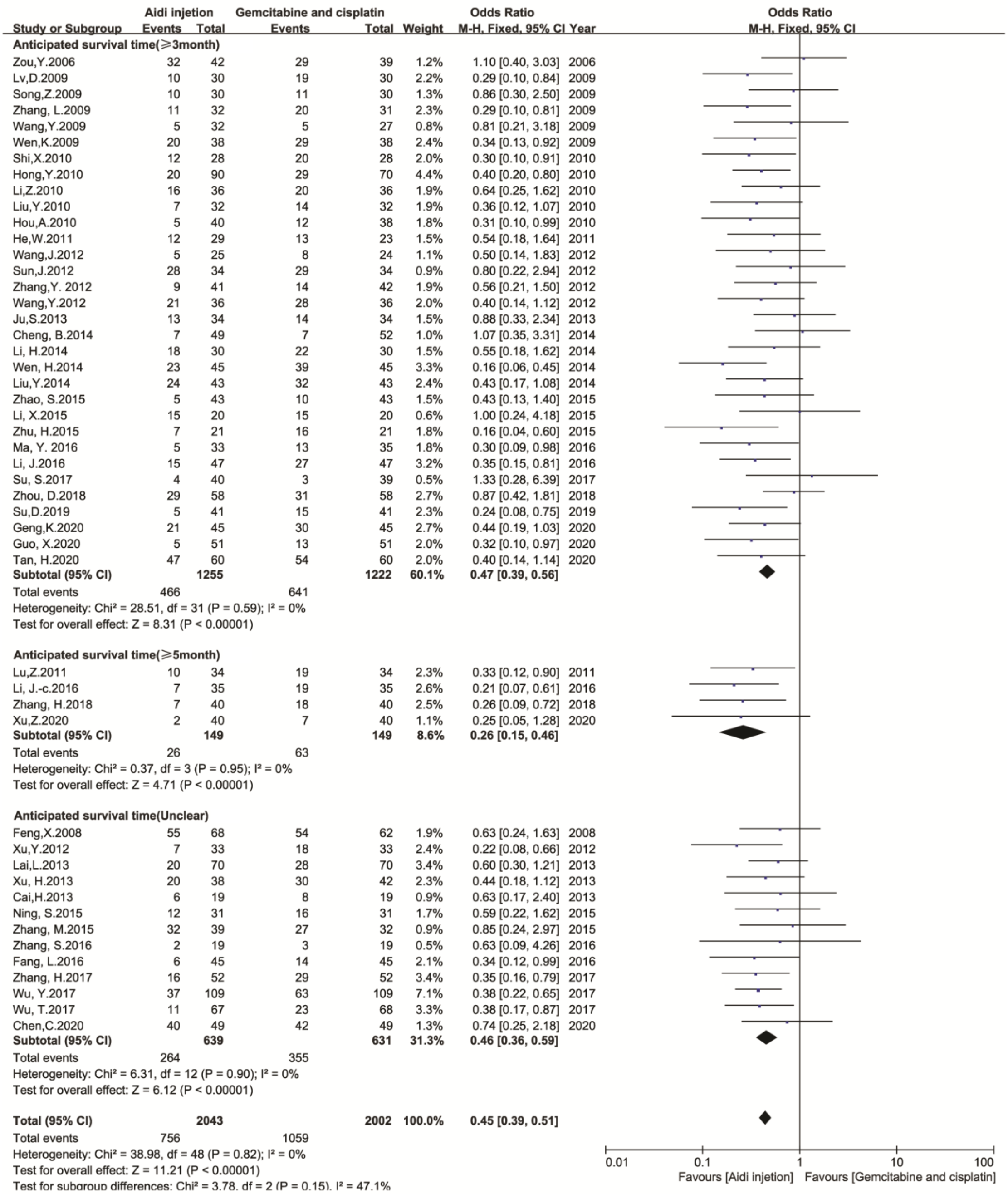

Figure S49 Subgroups analysis of gastrointestinal reaction by anticipated survival time

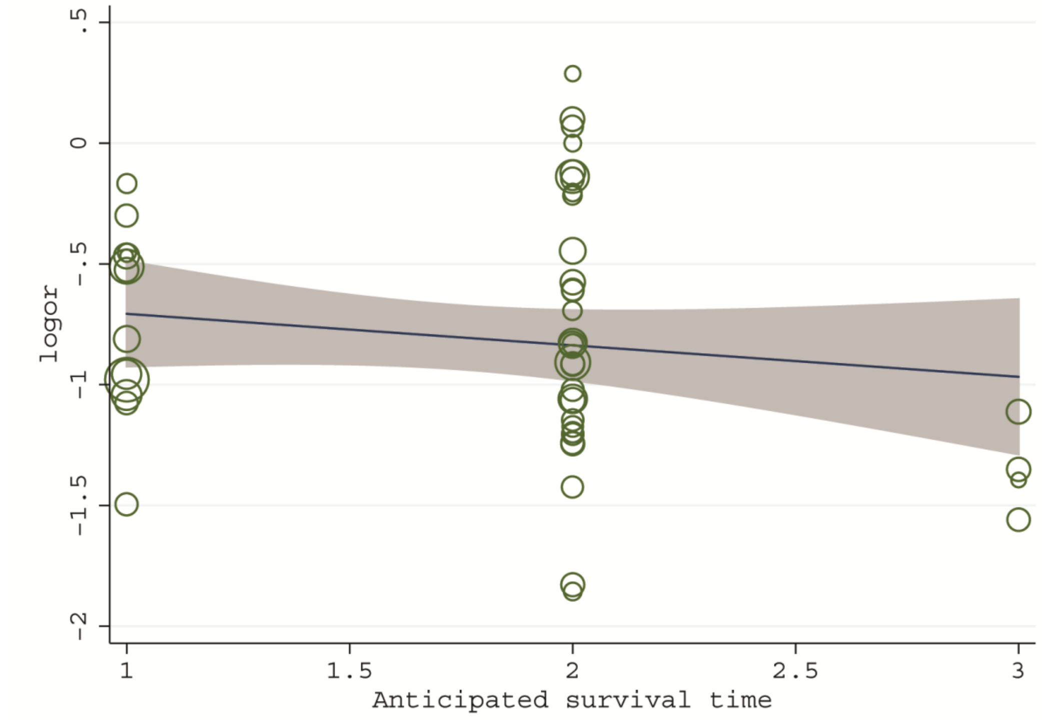

Figure S50 Meta-regression of gastrointestinal reaction by anticipated survival time

Study or Subgroup	Aidi injetion Events	Aidi injetion Total	Gemcitabine and cisplatin Events	Gemcitabine and cisplatin Total	Weight	Odds Ratio M-H, Fixed, 95% CI	Year	Odds Ratio M-H, Fixed, 95% CI
**Primary treatment(PT)**								
Feng,X.2008	30	68	25	62	3.5%	1.17 [0.58, 2.35]	2008	
Song,Z.2009	10	30	9	30	1.4%	1.17 [0.39, 3.47]	2009	
Hong,Y.2010	57	90	32	70	3.1%	2.05 [1.09, 3.88]	2010	
Shi,X.2010	13	28	10	28	1.3%	1.56 [0.53, 4.56]	2010	
Jiang,S.2011	15	32	12	30	1.6%	1.32 [0.48, 3.63]	2011	
Pei, W.2012	15	40	11	40	1.6%	1.58 [0.62, 4.07]	2012	
Li, J.2014	11	27	10	27	1.4%	1.17 [0.39, 3.49]	2014	
Cheng, B.2014	12	49	16	52	2.8%	0.73 [0.30, 1.76]	2014	
Zhang, X.2016	18	25	8	25	0.5%	5.46 [1.63, 18.36]	2016	
Chen, Y.2016	15	30	13	30	1.5%	1.31 [0.47, 3.61]	2016	
**Subtotal (95% CI)**		**419**		**394**	**18.7%**	**1.46 [1.10, 1.94]**		
Total events	196		146					
Heterogeneity: Chi² = 8.89, df = 9 (P = 0.45); I² = 0%								
Test for overall effect: Z = 2.61 (P = 0.009)								
**Retreatment(RT)**								
Ning, S.2015	19	31	11	31	1.0%	2.88 [1.03, 8.07]	2015	
**Subtotal (95% CI)**		**31**		**31**	**1.0%**	**2.88 [1.03, 8.07]**		
Total events	19		11					
Heterogeneity: Not applicable								
Test for overall effect: Z = 2.01 (P = 0.04)								
**PT/RT**								
Sun,G.2008	17	33	15	30	1.8%	1.06 [0.40, 2.86]	2008	
Wen,K.2009	16	38	15	38	2.1%	1.12 [0.45, 2.79]	2009	
Zhang, L.2009	15	32	14	31	1.8%	1.07 [0.40, 2.89]	2009	
**Subtotal (95% CI)**		**103**		**99**	**5.6%**	**1.08 [0.62, 1.89]**		
Total events	48		44					
Heterogeneity: Chi² = 0.01, df = 2 (P = 1.00); I² = 0%								
Test for overall effect: Z = 0.29 (P = 0.77)								
**Treatment process(unclear)**								
Zou,Y.2006	24	42	13	39	1.4%	2.67 [1.08, 6.58]	2006	
Zhao,H.2008	11	30	6	20	1.1%	1.35 [0.40, 4.53]	2008	
Yang,Q.2008	23	30	11	27	0.6%	4.78 [1.52, 14.98]	2008	
Wang,Y.2009	17	32	12	27	1.4%	1.42 [0.51, 3.96]	2009	
Lv,D.2009	22	30	11	30	0.7%	4.75 [1.58, 14.25]	2009	
Liu,Y.2010	16	32	10	32	1.2%	2.20 [0.79, 6.10]	2010	
Hou,A.2010	16	40	14	38	2.0%	1.14 [0.46, 2.85]	2010	
Li,Z.2010	16	36	12	36	1.6%	1.60 [0.62, 4.16]	2010	
He,W.2011	15	29	10	23	1.3%	1.39 [0.46, 4.18]	2011	
Fan,S.2011	22	41	12	38	1.4%	2.51 [1.00, 6.29]	2011	
Ding,P.2011	9	18	10	22	1.1%	1.20 [0.34, 4.18]	2011	
Wu,Q.2011	17	30	11	30	1.1%	2.26 [0.80, 6.36]	2011	
Lu,Z.2011	18	34	17	34	1.9%	1.13 [0.43, 2.91]	2011	
Zhang,Y. 2012	21	41	15	42	1.7%	1.89 [0.78, 4.55]	2012	
Sun,J.2012	15	34	13	34	1.7%	1.28 [0.48, 3.36]	2012	
Wang,Y.2012	16	36	14	36	1.8%	1.26 [0.49, 3.21]	2012	
Fu, L.2012	19	35	16	35	1.7%	1.41 [0.55, 3.61]	2012	
Xu,Y.2012	16	33	16	33	2.0%	1.00 [0.38, 2.63]	2012	
Wang,J.2012	14	25	10	24	1.1%	1.78 [0.57, 5.53]	2012	
Ju,S.2013	19	34	12	34	1.3%	2.32 [0.87, 6.16]	2013	
Xu, H.2013	19	38	18	42	2.0%	1.33 [0.55, 3.22]	2013	
Lai,L.2013	45	70	40	70	3.4%	1.35 [0.68, 2.67]	2013	
Liu,Y.2014	18	43	17	43	2.3%	1.10 [0.47, 2.60]	2014	
Li, H.2014	16	30	12	30	1.3%	1.71 [0.62, 4.77]	2014	
Wen, H.2014	28	45	17	45	1.5%	2.71 [1.16, 6.36]	2014	
Zhang, M.2015	17	39	12	32	1.8%	1.29 [0.50, 3.35]	2015	
Han, R.2015	14	36	10	36	1.4%	1.65 [0.61, 4.45]	2015	
Zhu, H.2015	15	21	7	21	0.5%	5.00 [1.35, 18.55]	2015	
Zhao, S.2015	19	43	12	43	1.6%	2.05 [0.83, 5.02]	2015	
Fang, L.2016	35	45	27	45	1.4%	2.33 [0.93, 5.87]	2016	
Li, J.2016	23	47	9	47	1.1%	4.05 [1.61, 10.20]	2016	
Ma, Y. 2016	19	33	19	35	1.9%	1.14 [0.44, 2.98]	2016	
Li, J.-c.2016	21	35	15	35	1.4%	2.00 [0.77, 5.18]	2016	
Zhang, S.2016	11	19	8	19	0.8%	1.89 [0.52, 6.85]	2016	
Huang, W.2017	17	39	11	40	1.5%	2.04 [0.80, 5.21]	2017	
Zhang, H.2017	42	52	26	52	1.2%	4.20 [1.75, 10.11]	2017	
Ma, M.2017	19	42	12	42	1.6%	2.07 [0.84, 5.10]	2017	
Wu, T.2017	53	67	40	68	2.0%	2.65 [1.24, 5.68]	2017	
Su, S.2017	35	40	23	39	0.7%	4.87 [1.57, 15.13]	2017	
Lv, W.2018	16	30	15	30	1.7%	1.14 [0.41, 3.15]	2018	
Zhou, D.2018	30	58	22	58	2.5%	1.75 [0.84, 3.67]	2018	
Su,D.2019	30	41	17	41	1.1%	3.85 [1.52, 9.75]	2019	
Liu, H.2019	19	44	12	44	1.6%	2.03 [0.83, 4.95]	2019	
Zhao,J.2019	31	43	22	43	1.5%	2.47 [1.01, 6.04]	2019	
Chen,C.2020	26	49	16	49	1.8%	2.33 [1.03, 5.29]	2020	
Geng,K.2020	27	45	17	45	1.6%	2.47 [1.06, 5.77]	2020	
Tan, H.2020	24	60	18	60	2.6%	1.56 [0.73, 3.31]	2020	
Xu, Y.2020	16	45	12	45	1.8%	1.52 [0.62, 3.73]	2020	
Xu,Z.2020	25	40	14	40	1.2%	3.10 [1.24, 7.71]	2020	
**Subtotal (95% CI)**		**1901**		**1873**	**74.6%**	**1.95 [1.71, 2.23]**		
Total events	1056		745					
Heterogeneity: Chi² = 36.62, df = 48 (P = 0.88); I² = 0%								
Test for overall effect: Z = 9.88 (P < 0.00001)								
**Total (95% CI)**		**2454**		**2397**	**100.0%**	**1.82 [1.62, 2.04]**		
Total events	1319		946					
Heterogeneity: Chi² = 52.84, df = 62 (P = 0.79); I² = 0%								
Test for overall effect: Z = 10.08 (P < 0.00001)								
Test for subgroup differences: Chi² = 7.45, df = 3 (P = 0.06), I² = 59.7%								

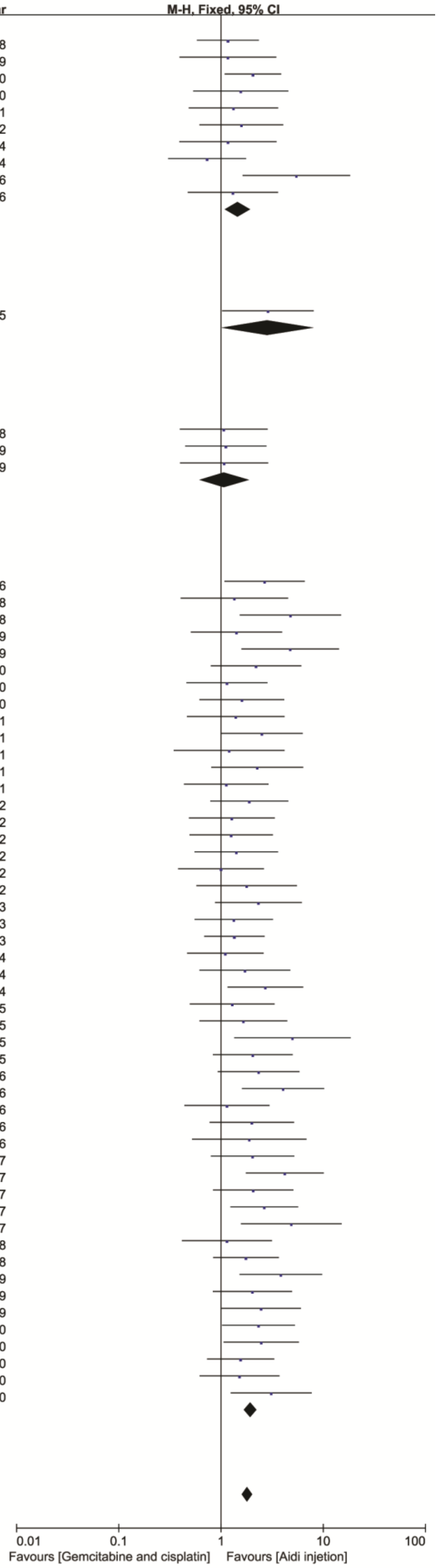

Figure S51 Subgroups analysis of objective response rate by treatment process

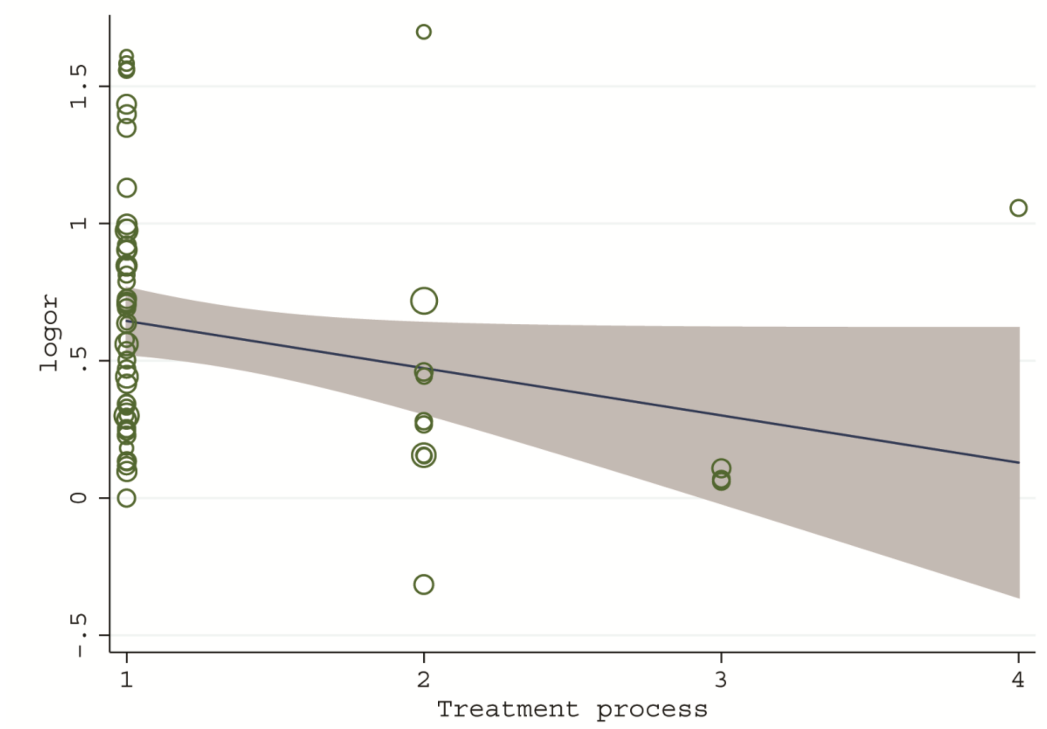

Figure S52 Meta-regression of objective response rate by treatment process

Study or Subgroup	Aidi injetion Events	Aidi injetion Total	Gemcitabine and cisplatin Events	Gemcitabine and cisplatin Total	Weight	Odds Ratio M-H, Fixed, 95% CI	Year
**Primary treatment(PT)**							
Feng,X.2008	62	68	56	62	2.3%	1.11 [0.34, 3.63]	2008
Song,Z.2009	27	30	25	30	1.1%	1.80 [0.39, 8.32]	2009
Shi,X.2010	27	28	25	28	0.4%	3.24 [0.32, 33.22]	2010
Hong,Y.2010	78	90	50	70	3.4%	2.60 [1.17, 5.78]	2010
Jiang,S.2011	29	32	25	30	1.1%	1.93 [0.42, 8.91]	2011
Pei, W.2012	35	40	27	40	1.5%	3.37 [1.07, 10.61]	2012
Li, J.2014	19	27	16	27	2.1%	1.63 [0.53, 5.04]	2014
Cheng, B.2014	39	49	42	52	3.7%	0.93 [0.35, 2.47]	2014
Zhang, X.2016	21	25	14	25	1.0%	4.13 [1.09, 15.59]	2016
Chen, Y.2016	24	30	18	30	1.6%	2.67 [0.84, 8.46]	2016
**Subtotal (95% CI)**		**419**		**394**	**18.3%**	**2.04 [1.41, 2.94]**	
Total events	361		298				
Heterogeneity: Chi² = 6.21, df = 9 (P = 0.72); I² = 0%							
Test for overall effect: Z = 3.81 (P = 0.0001)							
**Retreatment(RT)**							
Ning, S.2015	26	31	22	31	1.6%	2.13 [0.62, 7.29]	2015
**Subtotal (95% CI)**		**31**		**31**	**1.6%**	**2.13 [0.62, 7.29]**	
Total events	26		22				
Heterogeneity: Not applicable							
Test for overall effect: Z = 1.20 (P = 0.23)							
**PT/RT**							
Sun,G.2008	31	33	27	30	0.8%	1.72 [0.27, 11.09]	2008
Zhang, L.2009	27	32	23	31	1.6%	1.88 [0.54, 6.54]	2009
Wen,K.2009	32	38	32	38	2.3%	1.00 [0.29, 3.43]	2009
**Subtotal (95% CI)**		**103**		**99**	**4.7%**	**1.43 [0.65, 3.14]**	
Total events	90		82				
Heterogeneity: Chi² = 0.54, df = 2 (P = 0.76); I² = 0%							
Test for overall effect: Z = 0.88 (P = 0.38)							
**Treatment process(unclear)**							
Zou,Y.2006	35	42	25	39	1.9%	2.80 [0.99, 7.94]	2006
Yang,Q.2008	29	30	23	27	0.4%	5.04 [0.53, 48.26]	2008
Lv,D.2009	28	30	23	30	0.7%	4.26 [0.81, 22.53]	2009
Wang,Y.2009	29	32	21	27	1.0%	2.76 [0.62, 12.32]	2009
Liu,Y.2010	25	32	22	32	2.2%	1.62 [0.53, 4.99]	2010
Hou,A.2010	37	40	29	38	1.0%	3.83 [0.95, 15.43]	2010
Li,Z.2010	18	36	21	36	4.7%	0.71 [0.28, 1.81]	2010
Wu,Q.2011	28	30	26	30	0.8%	2.15 [0.36, 12.76]	2011
He,W.2011	26	29	19	23	1.0%	1.82 [0.36, 9.12]	2011
Lu,Z.2011	30	34	28	34	1.5%	1.61 [0.41, 6.30]	2011
Fan,S.2011	35	41	29	38	2.0%	1.81 [0.58, 5.68]	2011
Wang,Y.2012	28	36	20	36	2.0%	2.80 [1.01, 7.80]	2012
Sun,J.2012	25	34	25	34	3.0%	1.00 [0.34, 2.94]	2012
Xu,Y.2012	30	33	26	33	1.1%	2.69 [0.63, 11.49]	2012
Zhang,Y. 2012	34	41	32	42	2.4%	1.52 [0.52, 4.47]	2012
Fu, L.2012	32	35	28	35	1.1%	2.67 [0.63, 11.31]	2012
Wang,J.2012	20	25	16	24	1.5%	2.00 [0.55, 7.31]	2012
Lai,L.2013	63	70	60	70	2.7%	1.50 [0.54, 4.20]	2013
Ju,S.2013	32	34	29	34	0.8%	2.76 [0.50, 15.33]	2013
Xu, H.2013	33	38	34	42	1.9%	1.55 [0.46, 5.24]	2013
Wen, H.2014	43	45	36	45	0.7%	5.38 [1.09, 26.49]	2014
Liu,Y.2014	35	43	33	43	2.8%	1.33 [0.47, 3.77]	2014
Li, H.2014	26	30	23	30	1.4%	1.98 [0.51, 7.63]	2014
Zhu, H.2015	19	21	14	21	0.6%	4.75 [0.85, 26.43]	2015
Zhang, M.2015	28	39	23	32	3.2%	1.00 [0.35, 2.82]	2015
Han, R.2015	32	36	27	36	1.3%	2.67 [0.74, 9.63]	2015
Zhao, S.2015	37	43	28	43	1.8%	3.30 [1.14, 9.60]	2015
Zhang, S.2016	14	19	14	19	1.7%	1.00 [0.24, 4.24]	2016
Fang, L.2016	42	45	34	45	1.0%	4.53 [1.17, 17.55]	2016
Li, J.-c.2016	31	35	26	35	1.3%	2.68 [0.74, 9.73]	2016
Ma, Y. 2016	28	33	28	35	1.9%	1.40 [0.40, 4.94]	2016
Li, J.2016	45	47	34	47	0.7%	8.60 [1.82, 40.69]	2016
Wu, T.2017	62	67	56	68	1.9%	2.66 [0.88, 8.02]	2017
Ma, M.2017	37	42	29	42	1.6%	3.32 [1.06, 10.37]	2017
Huang, W.2017	36	39	30	40	1.0%	4.00 [1.01, 15.87]	2017
Zhang, H.2017	50	52	46	52	0.8%	3.26 [0.63, 16.97]	2017
Su, S.2017	37	40	26	39	0.9%	6.17 [1.60, 23.83]	2017
Zhou, D.2018	50	58	38	58	2.4%	3.29 [1.31, 8.27]	2018
Lv, W.2018	24	30	25	30	2.2%	0.80 [0.22, 2.97]	2018
Zhao,J.2019	39	43	34	43	1.4%	2.58 [0.73, 9.14]	2019
Su,D.2019	39	41	33	41	0.7%	4.73 [0.94, 23.82]	2019
Liu, H.2019	39	44	30	44	1.5%	3.64 [1.18, 11.23]	2019
Xu,Z.2020	33	40	24	40	1.9%	3.14 [1.12, 8.82]	2020
Chen,C.2020	44	49	34	49	1.6%	3.88 [1.28, 11.74]	2020
Geng,K.2020	39	45	31	45	1.9%	2.94 [1.01, 8.53]	2020
Tan, H.2020	49	60	39	60	3.2%	2.40 [1.03, 5.57]	2020
Xu, Y.2020	43	45	33	45	0.7%	7.82 [1.64, 37.36]	2020
**Subtotal (95% CI)**		**1853**		**1831**	**75.4%**	**2.41 [2.02, 2.87]**	
Total events	1618		1364				
Heterogeneity: Chi² = 36.03, df = 46 (P = 0.85); I² = 0%							
Test for overall effect: Z = 9.84 (P < 0.00001)							
**Total (95% CI)**		**2406**		**2355**	**100.0%**	**2.29 [1.97, 2.67]**	
Total events	2095		1766				
Heterogeneity: Chi² = 44.59, df = 60 (P = 0.93); I² = 0%							
Test for overall effect: Z = 10.58 (P < 0.00001)							
Test for subgroup differences: Chi² = 2.13, df = 3 (P = 0.55), I² = 0%							

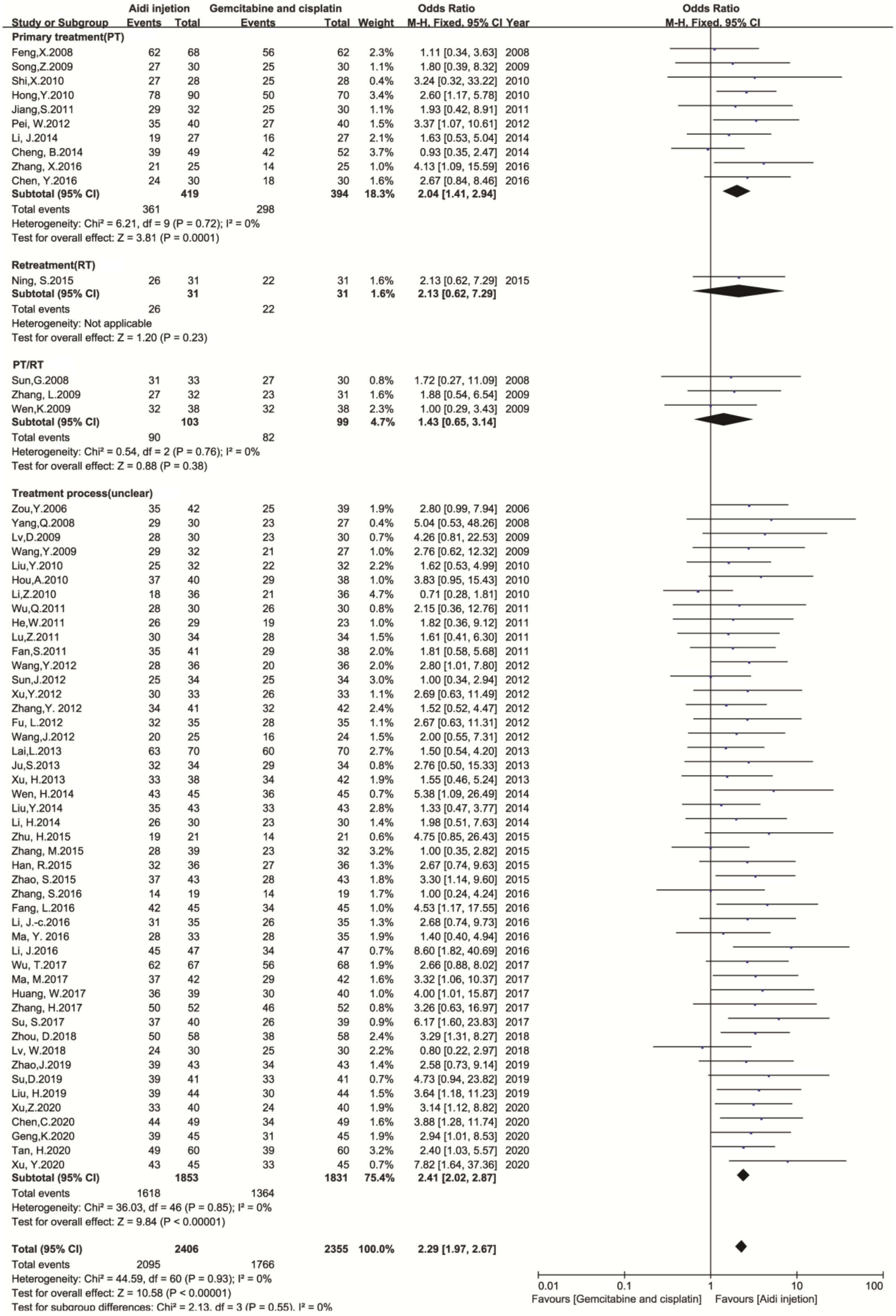

Figure S53 Subgroups analysis of disease control rate by treatment process

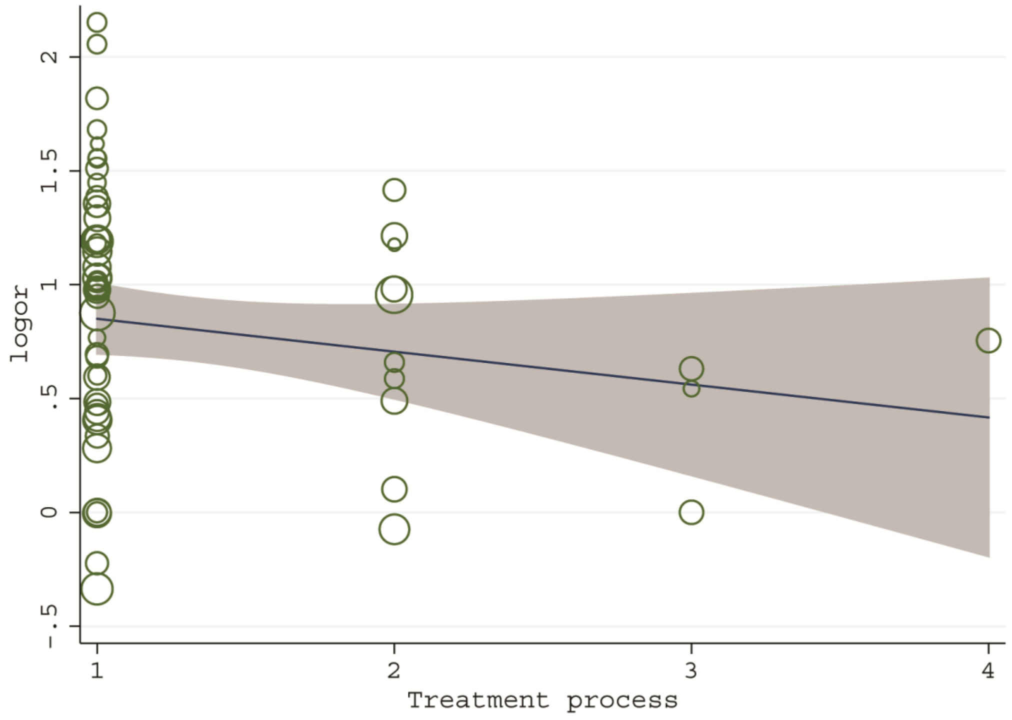

Figure S54 Meta-regression of disease control rate by treatment process

Study or Subgroup	Aidi injection Events	Aidi injection Total	Gemcitabine and cisplatin Events	Gemcitabine and cisplatin Total	Weight	Odds Ratio M-H, Fixed, 95% CI	Year
**Primary treatment(PT)**							
Feng,X.2008	64	68	60	62	0.9%	0.53 [0.09, 3.02]	2008
Hong,Y.2010	26	90	31	70	5.7%	0.51 [0.27, 0.98]	2010
Cheng, B.2014	34	49	47	52	3.2%	0.24 [0.08, 0.73]	2014
Li, J.2014	5	27	6	27	1.1%	0.80 [0.21, 3.00]	2014
**Subtotal (95% CI)**		**234**		**211**	**10.9%**	**0.46 [0.28, 0.76]**	
Total events	129		144				
Heterogeneity: Chi² = 2.09, df = 3 (P = 0.55); I² = 0%							
Test for overall effect: Z = 3.07 (P = 0.002)							
**Retreatment(RT)**							
Ning, S.2015	16	31	16	31	1.8%	1.00 [0.37, 2.71]	2015
**Subtotal (95% CI)**		**31**		**31**	**1.8%**	**1.00 [0.37, 2.71]**	
Total events	16		16				
Heterogeneity: Not applicable							
Test for overall effect: Z = 0.00 (P = 1.00)							
**PT/RT**							
Wen,K.2009	23	38	31	38	2.8%	0.35 [0.12, 0.99]	2009
Zhang, L.2009	5	32	14	31	2.8%	0.22 [0.07, 0.74]	2009
**Subtotal (95% CI)**		**70**		**69**	**5.6%**	**0.29 [0.13, 0.63]**	
Total events	28		45				
Heterogeneity: Chi² = 0.29, df = 1 (P = 0.59); I² = 0%							
Test for overall effect: Z = 3.13 (P = 0.002)							
**Treatment process(unclear)**							
Wang,Y.2009	26	32	22	27	1.0%	0.98 [0.26, 3.67]	2009
Hou,A.2010	14	40	22	38	3.4%	0.39 [0.16, 0.98]	2010
Lu,Z.2011	11	34	20	34	3.1%	0.33 [0.12, 0.90]	2011
Wang,J.2012	7	25	9	24	1.5%	0.65 [0.19, 2.16]	2012
Wang,Y.2012	19	36	26	36	2.8%	0.43 [0.16, 1.15]	2012
Fu, L.2012	24	35	32	35	2.3%	0.20 [0.05, 0.81]	2012
Zhang,Y. 2012	12	41	17	42	2.7%	0.61 [0.24, 1.52]	2012
Sun,J.2012	23	34	30	34	2.2%	0.28 [0.08, 0.99]	2012
Xu,Y.2012	23	33	33	33	2.4%	0.03 [0.00, 0.60]	2012
Xu, H.2013	13	38	22	42	3.2%	0.47 [0.19, 1.17]	2013
Liu,Y.2014	27	43	36	43	3.1%	0.33 [0.12, 0.91]	2014
Zhao, S.2015	7	43	10	43	1.9%	0.64 [0.22, 1.88]	2015
Li, X.2015	2	20	8	20	1.7%	0.17 [0.03, 0.92]	2015
Han, R.2015	7	36	11	36	2.0%	0.55 [0.18, 1.63]	2015
Zhang, M.2015	26	39	28	32	2.4%	0.29 [0.08, 0.99]	2015
Zhu, H.2015	6	21	15	21	2.5%	0.16 [0.04, 0.61]	2015
Zhang, S.2016	2	19	4	19	0.8%	0.44 [0.07, 2.76]	2016
Fang, L.2016	5	45	13	45	2.7%	0.31 [0.10, 0.95]	2016
Li, J.2016	12	47	19	47	3.3%	0.51 [0.21, 1.21]	2016
Li, J.-c.2016	14	35	26	35	3.6%	0.23 [0.08, 0.64]	2016
Ma, Y. 2016	7	33	15	35	2.7%	0.36 [0.12, 1.05]	2016
Wu, Y.2017	3	109	9	109	2.0%	0.31 [0.08, 1.19]	2017
Su, S.2017	2	40	2	39	0.4%	0.97 [0.13, 7.28]	2017
Zhang, H.2017	13	52	22	52	3.8%	0.45 [0.20, 1.05]	2017
Ma, M.2017	11	42	16	42	2.7%	0.58 [0.23, 1.46]	2017
Huang, W.2017	10	39	12	40	2.0%	0.80 [0.30, 2.16]	2017
Wu, T.2017	10	67	21	68	4.1%	0.39 [0.17, 0.92]	2017
Zhou, D.2018	11	58	13	58	2.4%	0.81 [0.33, 1.99]	2018
Zhang, H.2018	13	30	22	30	2.9%	0.28 [0.09, 0.82]	2018
Chen,C.2020	33	49	43	49	3.2%	0.29 [0.10, 0.82]	2020
Guo, X.2020	7	51	16	51	3.2%	0.35 [0.13, 0.94]	2020
Tan, H.2020	51	60	57	60	2.0%	0.30 [0.08, 1.16]	2020
Xu,Z.2020	2	40	6	40	1.3%	0.30 [0.06, 1.58]	2020
**Subtotal (95% CI)**		**1366**		**1359**	**81.7%**	**0.40 [0.33, 0.48]**	
Total events	453		657				
Heterogeneity: Chi² = 20.54, df = 32 (P = 0.94); I² = 0%							
Test for overall effect: Z = 9.53 (P < 0.00001)							
**Total (95% CI)**		**1701**		**1670**	**100.0%**	**0.41 [0.35, 0.49]**	
Total events	626		862				
Heterogeneity: Chi² = 26.97, df = 39 (P = 0.93); I² = 0%							
Test for overall effect: Z = 10.32 (P < 0.00001)							
Test for subgroup differences: Chi² = 4.17, df = 3 (P = 0.24), I² = 28.0%							

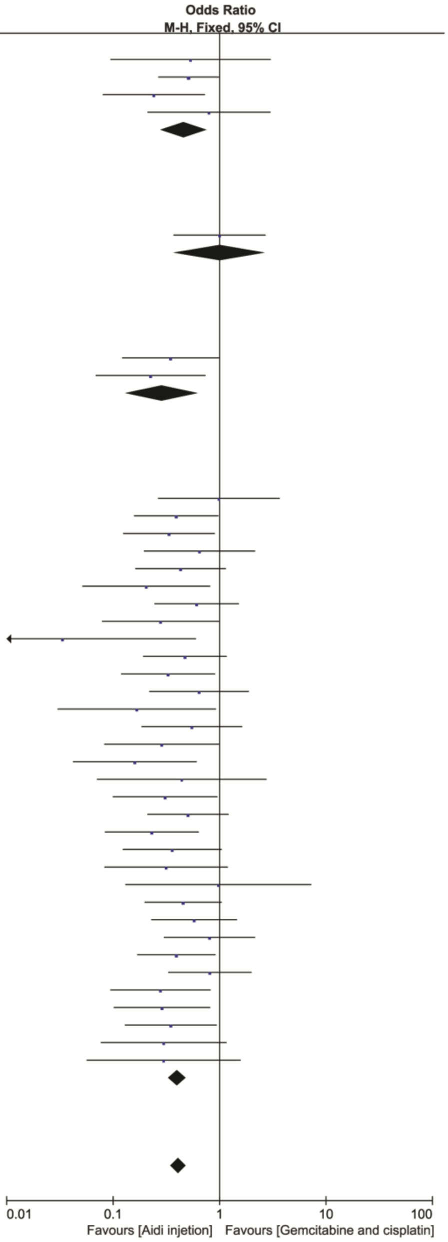

Figure S55 Subgroups analysis of neutropenia by treatment process

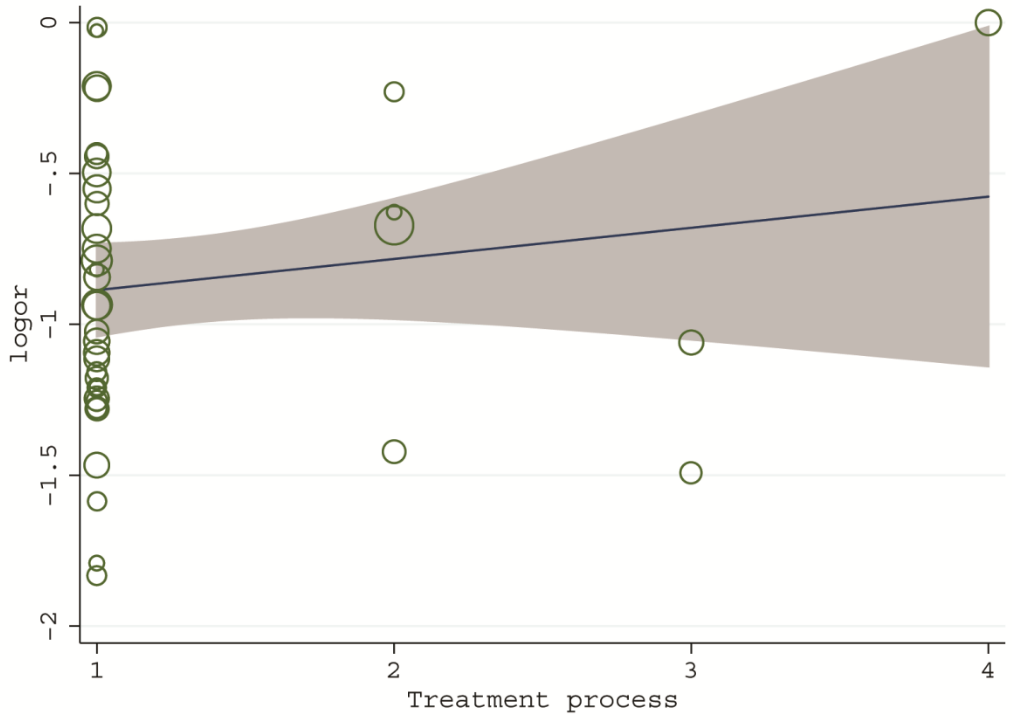

Figure S56 Meta-regression of neutropenia by treatment process

Study or Subgroup	Aidi injetion Events	Aidi injetion Total	Gemcitabine and cisplatin Events	Gemcitabine and cisplatin Total	Weight	Odds Ratio M-H, Fixed, 95% CI	Year
**Primary treatment(PT)**							
Feng,X.2008	55	68	54	62	1.9%	0.63 [0.24, 1.63]	2008
Song,Z.2009	10	30	11	30	1.3%	0.86 [0.30, 2.50]	2009
Hong,Y.2010	20	90	29	70	4.4%	0.40 [0.20, 0.80]	2010
Shi,X.2010	12	28	20	28	2.0%	0.30 [0.10, 0.91]	2010
Cheng, B.2014	7	49	7	52	1.0%	1.07 [0.35, 3.31]	2014
**Subtotal (95% CI)**		**265**		**242**	**10.4%**	**0.54 [0.36, 0.82]**	
Total events	104		121				
Heterogeneity: Chi² = 4.02, df = 4 (P = 0.40); I² = 1%							
Test for overall effect: Z = 2.88 (P = 0.004)							
**Retreatment(RT)**							
Ning, S.2015	12	31	16	31	1.7%	0.59 [0.22, 1.62]	2015
**Subtotal (95% CI)**		**31**		**31**	**1.7%**	**0.59 [0.22, 1.62]**	
Total events	12		16				
Heterogeneity: Not applicable							
Test for overall effect: Z = 1.02 (P = 0.31)							
**PT/RT**							
Zhang, L.2009	11	32	20	31	2.3%	0.29 [0.10, 0.81]	2009
Wen,K.2009	20	38	29	38	2.4%	0.34 [0.13, 0.92]	2009
**Subtotal (95% CI)**		**70**		**69**	**4.6%**	**0.32 [0.16, 0.65]**	
Total events	31		49				
Heterogeneity: Chi² = 0.06, df = 1 (P = 0.81); I² = 0%							
Test for overall effect: Z = 3.16 (P = 0.002)							
**Treatment process(unclear)**							
Zou,Y.2006	32	42	29	39	1.2%	1.10 [0.40, 3.03]	2006
Wang,Y.2009	5	32	5	27	0.8%	0.81 [0.21, 3.18]	2009
Lv,D.2009	10	30	19	30	2.2%	0.29 [0.10, 0.84]	2009
Hou,A.2010	5	40	12	38	1.8%	0.31 [0.10, 0.99]	2010
Li,Z.2010	16	36	20	36	1.9%	0.64 [0.25, 1.62]	2010
Liu,Y.2010	7	32	14	32	1.9%	0.36 [0.12, 1.07]	2010
He,W.2011	12	29	13	23	1.5%	0.54 [0.18, 1.64]	2011
Lu,Z.2011	10	34	19	34	2.3%	0.33 [0.12, 0.90]	2011
Wang,J.2012	5	25	8	24	1.1%	0.50 [0.14, 1.83]	2012
Zhang,Y. 2012	9	41	14	42	1.9%	0.56 [0.21, 1.50]	2012
Xu,Y.2012	7	33	18	33	2.4%	0.22 [0.08, 0.66]	2012
Sun,J.2012	28	34	29	34	0.9%	0.80 [0.22, 2.94]	2012
Wang,Y.2012	21	36	28	36	2.0%	0.40 [0.14, 1.12]	2012
Ju,S.2013	13	34	14	34	1.5%	0.88 [0.33, 2.34]	2013
Xu, H.2013	20	38	30	42	2.3%	0.44 [0.18, 1.12]	2013
Cai,H.2013	6	19	8	19	0.9%	0.63 [0.17, 2.40]	2013
Lai,L.2013	20	70	28	70	3.4%	0.60 [0.30, 1.21]	2013
Wen, H.2014	23	45	39	45	3.3%	0.16 [0.06, 0.45]	2014
Liu,Y.2014	24	43	32	43	2.4%	0.43 [0.17, 1.08]	2014
Li, H.2014	18	30	22	30	1.5%	0.55 [0.18, 1.62]	2014
Zhao, S.2015	5	43	10	43	1.5%	0.43 [0.13, 1.40]	2015
Li, X.2015	15	20	15	20	0.6%	1.00 [0.24, 4.18]	2015
Zhu, H.2015	7	21	16	21	1.8%	0.16 [0.04, 0.60]	2015
Zhang, M.2015	32	39	27	32	0.9%	0.85 [0.24, 2.97]	2015
Li, J.-c.2016	7	35	19	35	2.6%	0.21 [0.07, 0.61]	2016
Ma, Y. 2016	5	33	13	35	1.8%	0.30 [0.09, 0.98]	2016
Zhang, S.2016	2	19	3	19	0.5%	0.63 [0.09, 4.26]	2016
Li, J.2016	15	47	27	47	3.2%	0.35 [0.15, 0.81]	2016
Fang, L.2016	6	45	14	45	2.1%	0.34 [0.12, 0.99]	2016
Wu, Y.2017	37	109	63	109	7.1%	0.38 [0.22, 0.65]	2017
Zhang, H.2017	16	52	29	52	3.4%	0.35 [0.16, 0.79]	2017
Su, S.2017	4	40	3	39	0.5%	1.33 [0.28, 6.39]	2017
Wu, T.2017	11	67	23	68	3.3%	0.38 [0.17, 0.87]	2017
Zhou, D.2018	29	58	31	58	2.7%	0.87 [0.42, 1.81]	2018
Zhang, H.2018	7	40	18	40	2.5%	0.26 [0.09, 0.72]	2018
Su,D.2019	5	41	15	41	2.3%	0.24 [0.08, 0.75]	2019
Chen,C.2020	40	49	42	49	1.3%	0.74 [0.25, 2.18]	2020
Geng,K.2020	21	45	30	45	2.7%	0.44 [0.19, 1.03]	2020
Guo, X.2020	5	51	13	51	2.0%	0.32 [0.10, 0.97]	2020
Tan, H.2020	47	60	54	60	2.0%	0.40 [0.14, 1.14]	2020
Xu,Z.2020	2	40	7	40	1.1%	0.25 [0.05, 1.28]	2020
**Subtotal (95% CI)**		**1677**		**1660**	**83.3%**	**0.44 [0.37, 0.51]**	
Total events	609		873				
Heterogeneity: Chi² = 32.85, df = 40 (P = 0.78); I² = 0%							
Test for overall effect: Z = 10.40 (P < 0.00001)							
**Total (95% CI)**		**2043**		**2002**	**100.0%**	**0.45 [0.39, 0.51]**	
Total events	756		1059				
Heterogeneity: Chi² = 38.98, df = 48 (P = 0.82); I² = 0%							
Test for overall effect: Z = 11.21 (P < 0.00001)							
Test for subgroup differences: Chi² = 2.11, df = 3 (P = 0.55), I² = 0%							

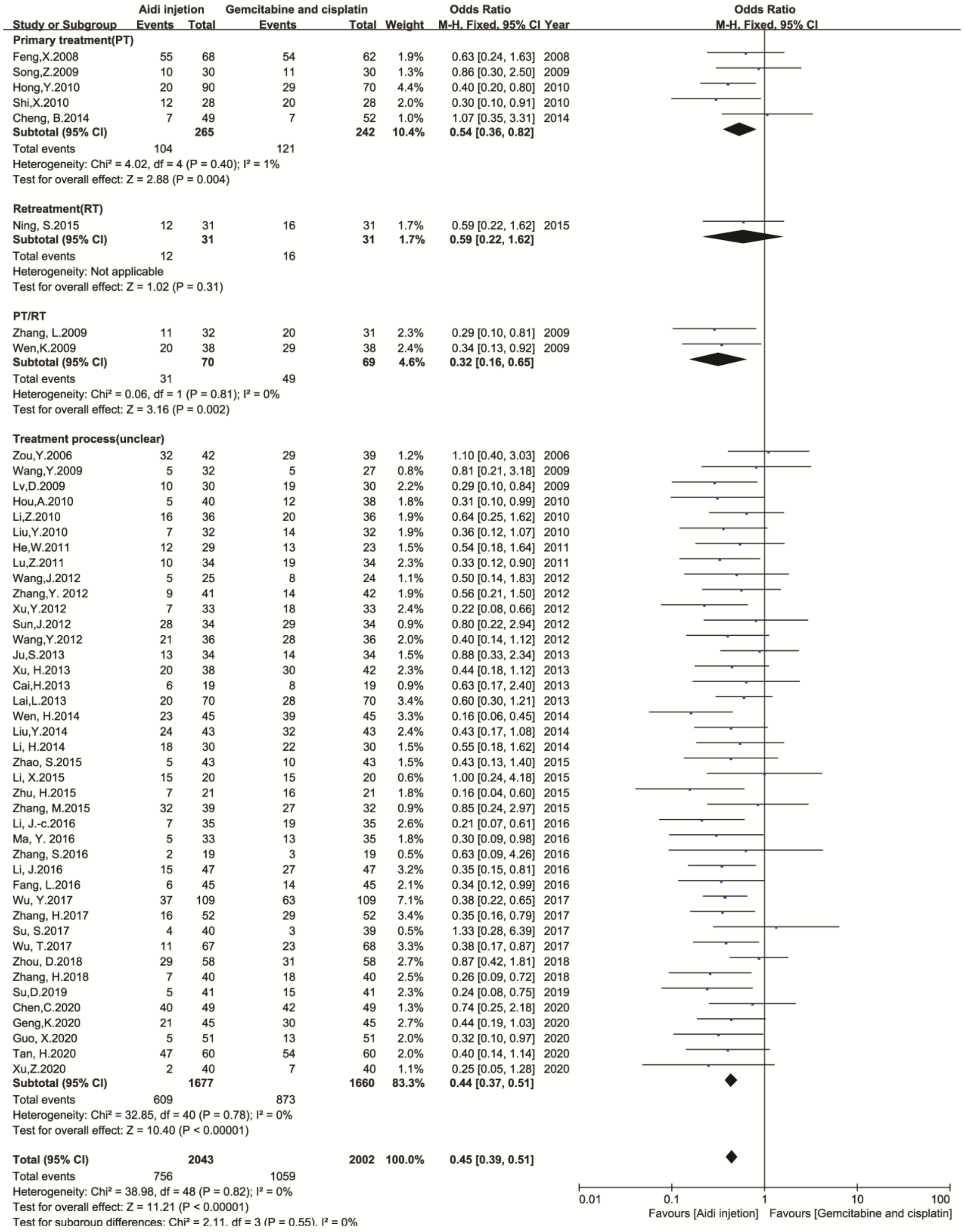

Figure S57 Subgroups analysis of gastrointestinal reaction by treatment process

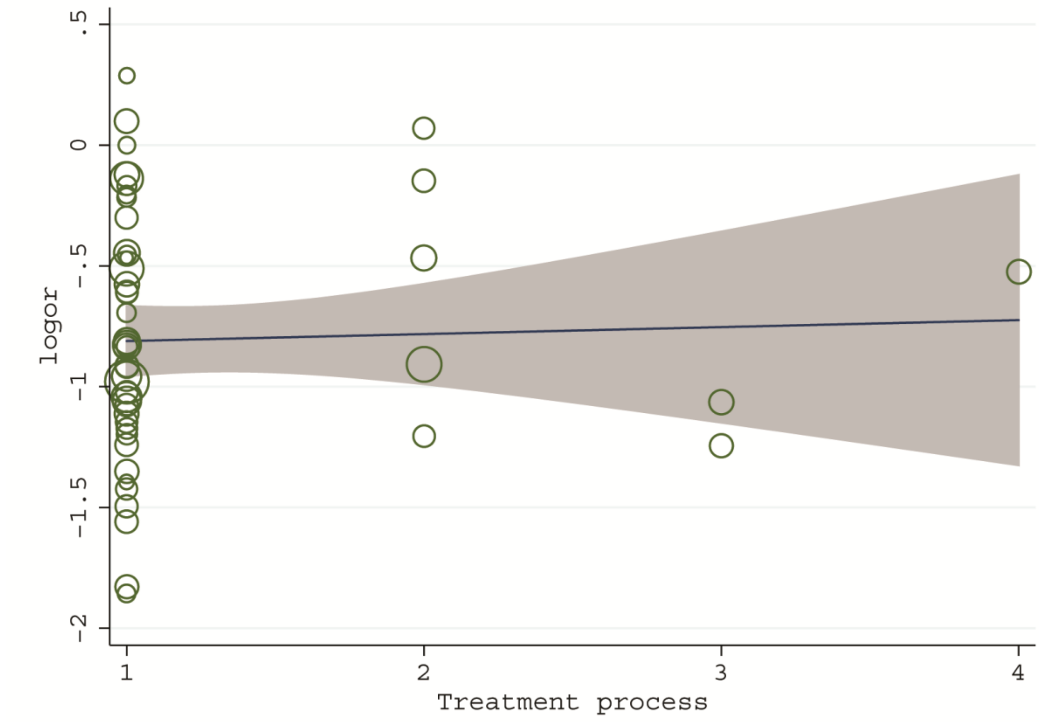

Figure S58 Meta-regression of gastrointestinal reaction by treatment process

Study or Subgroup	Aidi injetion Events	Aidi injetion Total	Gemcitabine and cisplatin Events	Gemcitabine and cisplatin Total	Weight	Odds Ratio M-H, Fixed, 95% CI	Year
**Age(≥60)**							
Jiang,S.2011	15	32	12	30	1.6%	1.32 [0.48, 3.63]	2011
Sun,J.2012	15	34	13	34	1.7%	1.28 [0.48, 3.36]	2012
Fu, L.2012	19	35	16	35	1.7%	1.41 [0.55, 3.61]	2012
Ju,S.2013	19	34	12	34	1.3%	2.32 [0.87, 6.16]	2013
Wen, H.2014	28	45	17	45	1.5%	2.71 [1.16, 6.36]	2014
Zhang, M.2015	17	39	12	32	1.8%	1.29 [0.50, 3.35]	2015
Zhu, H.2015	15	21	7	21	0.5%	5.00 [1.35, 18.55]	2015
Chen,C.2020	26	49	16	49	1.8%	2.33 [1.03, 5.29]	2020
Tan, H.2020	24	60	18	60	2.6%	1.56 [0.73, 3.31]	2020
**Subtotal (95% CI)**		**349**		**340**	**14.4%**	**1.85 [1.36, 2.51]**	
Total events	178		123				
Heterogeneity: Chi² = 5.56, df = 8 (P = 0.70); I² = 0%							
Test for overall effect: Z = 3.93 (P < 0.0001)							
**Age(others)**							
Zou,Y.2006	24	42	13	39	1.4%	2.67 [1.08, 6.58]	2006
Sun,G.2008	17	33	15	30	1.8%	1.06 [0.40, 2.86]	2008
Feng,X.2008	30	68	25	62	3.5%	1.17 [0.58, 2.35]	2008
Zhao,H.2008	11	30	6	20	1.1%	1.35 [0.40, 4.53]	2008
Yang,Q.2008	23	30	11	27	0.6%	4.78 [1.52, 14.98]	2008
Wang,Y.2009	17	32	12	27	1.4%	1.42 [0.51, 3.96]	2009
Lv,D.2009	22	30	11	30	0.7%	4.75 [1.58, 14.25]	2009
Zhang, L.2009	15	32	14	31	1.8%	1.07 [0.40, 2.89]	2009
Wen,K.2009	16	38	15	38	2.1%	1.12 [0.45, 2.79]	2009
Song,Z.2009	10	30	9	30	1.4%	1.17 [0.39, 3.47]	2009
Liu,Y.2010	16	32	10	32	1.2%	2.20 [0.79, 6.10]	2010
Li,Z.2010	16	36	12	36	1.6%	1.60 [0.62, 4.16]	2010
Hong,Y.2010	57	90	32	70	3.1%	2.05 [1.09, 3.88]	2010
Hou,A.2010	16	40	14	38	2.0%	1.14 [0.46, 2.85]	2010
Shi,X.2010	13	28	10	28	1.3%	1.56 [0.53, 4.56]	2010
Ding,P.2011	9	18	10	22	1.1%	1.20 [0.34, 4.18]	2011
He,W.2011	15	29	10	23	1.3%	1.39 [0.46, 4.18]	2011
Fan,S.2011	22	41	12	38	1.4%	2.51 [1.00, 6.29]	2011
Wu,Q.2011	17	30	11	30	1.1%	2.26 [0.80, 6.36]	2011
Lu,Z.2011	18	34	17	34	1.9%	1.13 [0.43, 2.91]	2011
Wang,J.2012	14	25	10	24	1.1%	1.78 [0.57, 5.53]	2012
Wang,Y.2012	16	36	14	36	1.8%	1.26 [0.49, 3.21]	2012
Xu,Y.2012	16	33	16	33	2.0%	1.00 [0.38, 2.63]	2012
Zhang,Y. 2012	21	41	15	42	1.7%	1.89 [0.78, 4.55]	2012
Pei, W.2012	15	40	11	40	1.6%	1.58 [0.62, 4.07]	2012
Lai,L.2013	45	70	40	70	3.4%	1.35 [0.68, 2.67]	2013
Xu, H.2013	19	38	18	42	2.0%	1.33 [0.55, 3.22]	2013
Cheng, B.2014	12	49	16	52	2.8%	0.73 [0.30, 1.76]	2014
Liu,Y.2014	18	43	17	43	2.3%	1.10 [0.47, 2.60]	2014
Li, H.2014	16	30	12	30	1.3%	1.71 [0.62, 4.77]	2014
Li, J.2014	11	27	10	27	1.4%	1.17 [0.39, 3.49]	2014
Han, R.2015	14	36	10	36	1.4%	1.65 [0.61, 4.45]	2015
Zhao, S.2015	19	43	12	43	1.6%	2.05 [0.83, 5.02]	2015
Ning, S.2015	19	31	11	31	1.0%	2.88 [1.03, 8.07]	2015
Chen, Y.2016	15	30	13	30	1.5%	1.31 [0.47, 3.61]	2016
Zhang, S.2016	11	19	8	19	0.8%	1.89 [0.52, 6.85]	2016
Li, J.-c.2016	21	35	15	35	1.4%	2.00 [0.77, 5.18]	2016
Ma, Y. 2016	19	33	19	35	1.9%	1.14 [0.44, 2.98]	2016
Zhang, X.2016	18	25	8	25	0.5%	5.46 [1.63, 18.36]	2016
Fang, L.2016	35	45	27	45	1.4%	2.33 [0.93, 5.87]	2016
Li, J.2016	23	47	9	47	1.1%	4.05 [1.61, 10.20]	2016
Huang, W.2017	17	39	11	40	1.5%	2.04 [0.80, 5.21]	2017
Wu, T.2017	53	67	40	68	2.0%	2.65 [1.24, 5.68]	2017
Su, S.2017	35	40	23	39	0.7%	4.87 [1.57, 15.13]	2017
Ma, M.2017	19	42	12	42	1.6%	2.07 [0.84, 5.10]	2017
Zhang, H.2017	42	52	26	52	1.2%	4.20 [1.75, 10.11]	2017
Lv, W.2018	16	30	15	30	1.7%	1.14 [0.41, 3.15]	2018
Zhou, D.2018	30	58	22	58	2.5%	1.75 [0.84, 3.67]	2018
Liu, H.2019	19	44	12	44	1.6%	2.03 [0.83, 4.95]	2019
Su,D.2019	30	41	17	41	1.1%	3.85 [1.52, 9.75]	2019
Zhao,J.2019	31	43	22	43	1.5%	2.47 [1.01, 6.04]	2019
Geng,K.2020	27	45	17	45	1.6%	2.47 [1.06, 5.77]	2020
Xu, Y.2020	16	45	12	45	1.8%	1.52 [0.62, 3.73]	2020
Xu,Z.2020	25	40	14	40	1.2%	3.10 [1.24, 7.71]	2020
**Subtotal (95% CI)**		**2105**		**2057**	**85.6%**	**1.81 [1.60, 2.06]**	
Total events	1141		823				
Heterogeneity: Chi² = 47.26, df = 53 (P = 0.70); I² = 0%							
Test for overall effect: Z = 9.28 (P < 0.00001)							
**Total (95% CI)**		**2454**		**2397**	**100.0%**	**1.82 [1.62, 2.04]**	
Total events	1319		946				
Heterogeneity: Chi² = 52.84, df = 62 (P = 0.79); I² = 0%							
Test for overall effect: Z = 10.08 (P < 0.00001)							
Test for subgroup differences: Chi² = 0.01, df = 1 (P = 0.92), I² = 0%							

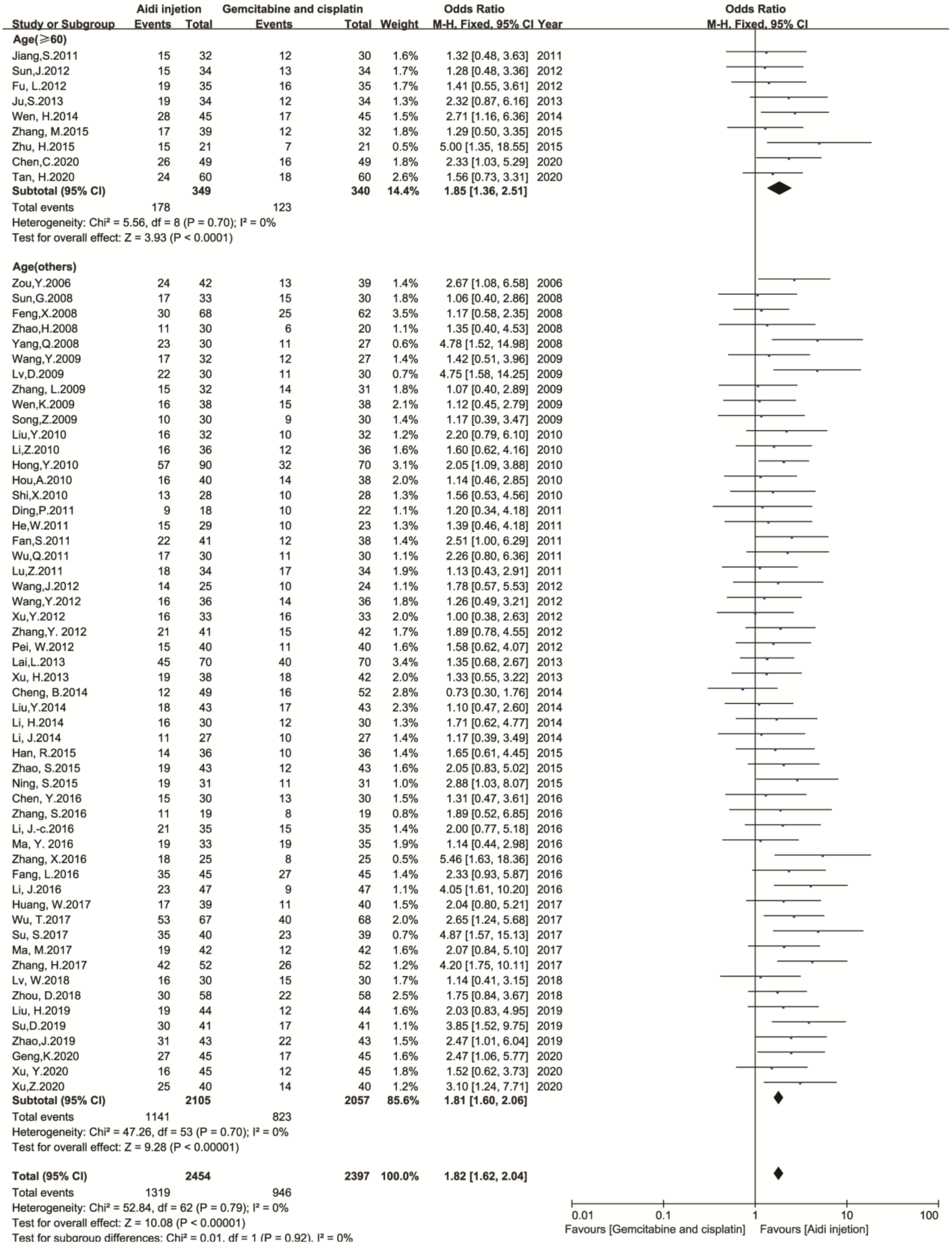

Figure S59 Subgroups analysis of objective response rate by age

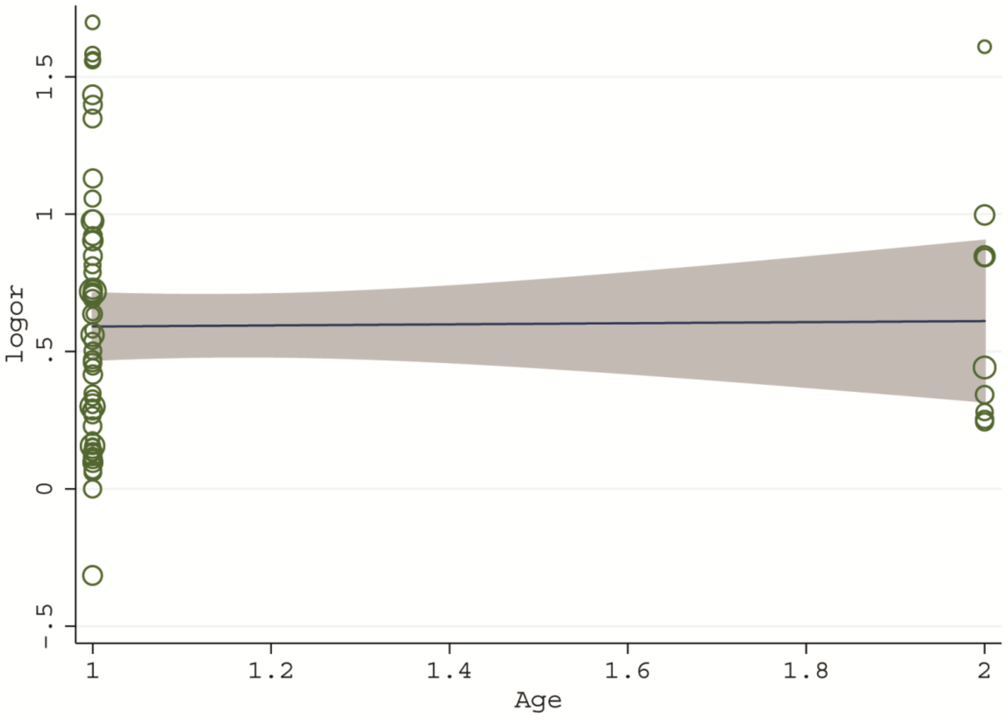

Figure S60 Meta-regression of objective response rate by age

Study or Subgroup	Aidi injetion Events	Aidi injetion Total	Gemcitabine and cisplatin Events	Gemcitabine and cisplatin Total	Weight	Odds Ratio M-H, Fixed, 95% CI	Year
**Age(≥60)**							
Jiang,S.2011	29	32	25	30	1.1%	1.93 [0.42, 8.91]	2011
Fu, L.2012	32	35	28	35	1.1%	2.67 [0.63, 11.31]	2012
Sun,J.2012	25	34	25	34	3.0%	1.00 [0.34, 2.94]	2012
Ju,S.2013	32	34	29	34	0.8%	2.76 [0.50, 15.33]	2013
Wen, H.2014	43	45	36	45	0.7%	5.38 [1.09, 26.49]	2014
Zhang, M.2015	28	39	23	32	3.2%	1.00 [0.35, 2.82]	2015
Zhu, H.2015	19	21	14	21	0.6%	4.75 [0.85, 26.43]	2015
Tan, H.2020	49	60	39	60	3.2%	2.40 [1.03, 5.57]	2020
Chen,C.2020	44	49	34	49	1.6%	3.88 [1.28, 11.74]	2020
**Subtotal (95% CI)**		**349**		**340**	**15.2%**	**2.22 [1.49, 3.30]**	
Total events	301		253				
Heterogeneity: Chi² = 7.49, df = 8 (P = 0.48); I² = 0%							
Test for overall effect: Z = 3.94 (P < 0.0001)							
**Age(others)**							
Zou,Y.2006	35	42	25	39	1.9%	2.80 [0.99, 7.94]	2006
Feng,X.2008	62	68	56	62	2.3%	1.11 [0.34, 3.63]	2008
Yang,Q.2008	29	30	23	27	0.4%	5.04 [0.53, 48.26]	2008
Sun,G.2008	31	33	27	30	0.8%	1.72 [0.27, 11.09]	2008
Wen,K.2009	32	38	32	38	2.3%	1.00 [0.29, 3.43]	2009
Zhang, L.2009	27	32	23	31	1.6%	1.88 [0.54, 6.54]	2009
Song,Z.2009	27	30	25	30	1.1%	1.80 [0.39, 8.32]	2009
Lv,D.2009	28	30	23	30	0.7%	4.26 [0.81, 22.53]	2009
Wang,Y.2009	29	32	21	27	1.0%	2.76 [0.62, 12.32]	2009
Shi,X.2010	27	28	25	28	0.4%	3.24 [0.32, 33.22]	2010
Liu,Y.2010	25	32	22	32	2.2%	1.62 [0.53, 4.99]	2010
Hong,Y.2010	78	90	50	70	3.4%	2.60 [1.17, 5.78]	2010
Li,Z.2010	18	36	21	36	4.7%	0.71 [0.28, 1.81]	2010
Hou,A.2010	37	40	29	38	1.0%	3.83 [0.95, 15.43]	2010
Fan,S.2011	35	41	29	38	2.0%	1.81 [0.58, 5.68]	2011
He,W.2011	26	29	19	23	1.0%	1.82 [0.36, 9.12]	2011
Wu,Q.2011	28	30	26	30	0.8%	2.15 [0.36, 12.76]	2011
Lu,Z.2011	30	34	28	34	1.5%	1.61 [0.41, 6.30]	2011
Wang,J.2012	20	25	16	24	1.5%	2.00 [0.55, 7.31]	2012
Wang,Y.2012	28	36	20	36	2.0%	2.80 [1.01, 7.80]	2012
Pei, W.2012	35	40	27	40	1.5%	3.37 [1.07, 10.61]	2012
Zhang,Y. 2012	34	41	32	42	2.4%	1.52 [0.52, 4.47]	2012
Xu,Y.2012	30	33	26	33	1.1%	2.69 [0.63, 11.49]	2012
Lai,L.2013	63	70	60	70	2.7%	1.50 [0.54, 4.20]	2013
Xu, H.2013	33	38	34	42	1.9%	1.55 [0.46, 5.24]	2013
Li, H.2014	26	30	23	30	1.4%	1.98 [0.51, 7.63]	2014
Liu,Y.2014	35	43	33	43	2.8%	1.33 [0.47, 3.77]	2014
Li, J.2014	19	27	16	27	2.1%	1.63 [0.53, 5.04]	2014
Cheng, B.2014	39	49	42	52	3.7%	0.93 [0.35, 2.47]	2014
Han, R.2015	32	36	27	36	1.3%	2.67 [0.74, 9.63]	2015
Ning, S.2015	26	31	22	31	1.6%	2.13 [0.62, 7.29]	2015
Zhao, S.2015	37	43	28	43	1.8%	3.30 [1.14, 9.60]	2015
Chen, Y.2016	24	30	18	30	1.6%	2.67 [0.84, 8.46]	2016
Zhang, S.2016	14	19	14	19	1.7%	1.00 [0.24, 4.24]	2016
Zhang, X.2016	21	25	14	25	1.0%	4.13 [1.09, 15.59]	2016
Ma, Y. 2016	28	33	28	35	1.9%	1.40 [0.40, 4.94]	2016
Li, J.-c.2016	31	35	26	35	1.3%	2.68 [0.74, 9.73]	2016
Fang, L.2016	42	45	34	45	1.0%	4.53 [1.17, 17.55]	2016
Li, J.2016	45	47	34	47	0.7%	8.60 [1.82, 40.69]	2016
Wu, T.2017	62	67	56	68	1.9%	2.66 [0.88, 8.02]	2017
Su, S.2017	37	40	26	39	0.9%	6.17 [1.60, 23.83]	2017
Ma, M.2017	37	42	29	42	1.6%	3.32 [1.06, 10.37]	2017
Huang, W.2017	36	39	30	40	1.0%	4.00 [1.01, 15.87]	2017
Zhang, H.2017	50	52	46	52	0.8%	3.26 [0.63, 16.97]	2017
Lv, W.2018	24	30	25	30	2.2%	0.80 [0.22, 2.97]	2018
Zhou, D.2018	50	58	38	58	2.4%	3.29 [1.31, 8.27]	2018
Su,D.2019	39	41	33	41	0.7%	4.73 [0.94, 23.82]	2019
Zhao,J.2019	39	43	34	43	1.4%	2.58 [0.73, 9.14]	2019
Liu, H.2019	39	44	30	44	1.5%	3.64 [1.18, 11.23]	2019
Geng,K.2020	39	45	31	45	1.9%	2.94 [1.01, 8.53]	2020
Xu, Y.2020	43	45	33	45	0.7%	7.82 [1.64, 37.36]	2020
Xu,Z.2020	33	40	24	40	1.9%	3.14 [1.12, 8.82]	2020
**Subtotal (95% CI)**		**2057**		**2015**	**84.8%**	**2.30 [1.95, 2.72]**	
Total events	1794		1513				
Heterogeneity: Chi² = 37.07, df = 51 (P = 0.93); I² = 0%							
Test for overall effect: Z = 9.82 (P < 0.00001)							
**Total (95% CI)**		**2406**		**2355**	**100.0%**	**2.29 [1.97, 2.67]**	
Total events	2095		1766				
Heterogeneity: Chi² = 44.59, df = 60 (P = 0.93); I² = 0%							
Test for overall effect: Z = 10.58 (P < 0.00001)							
Test for subgroup differences: Chi² = 0.03, df = 1 (P = 0.86), I² = 0%							

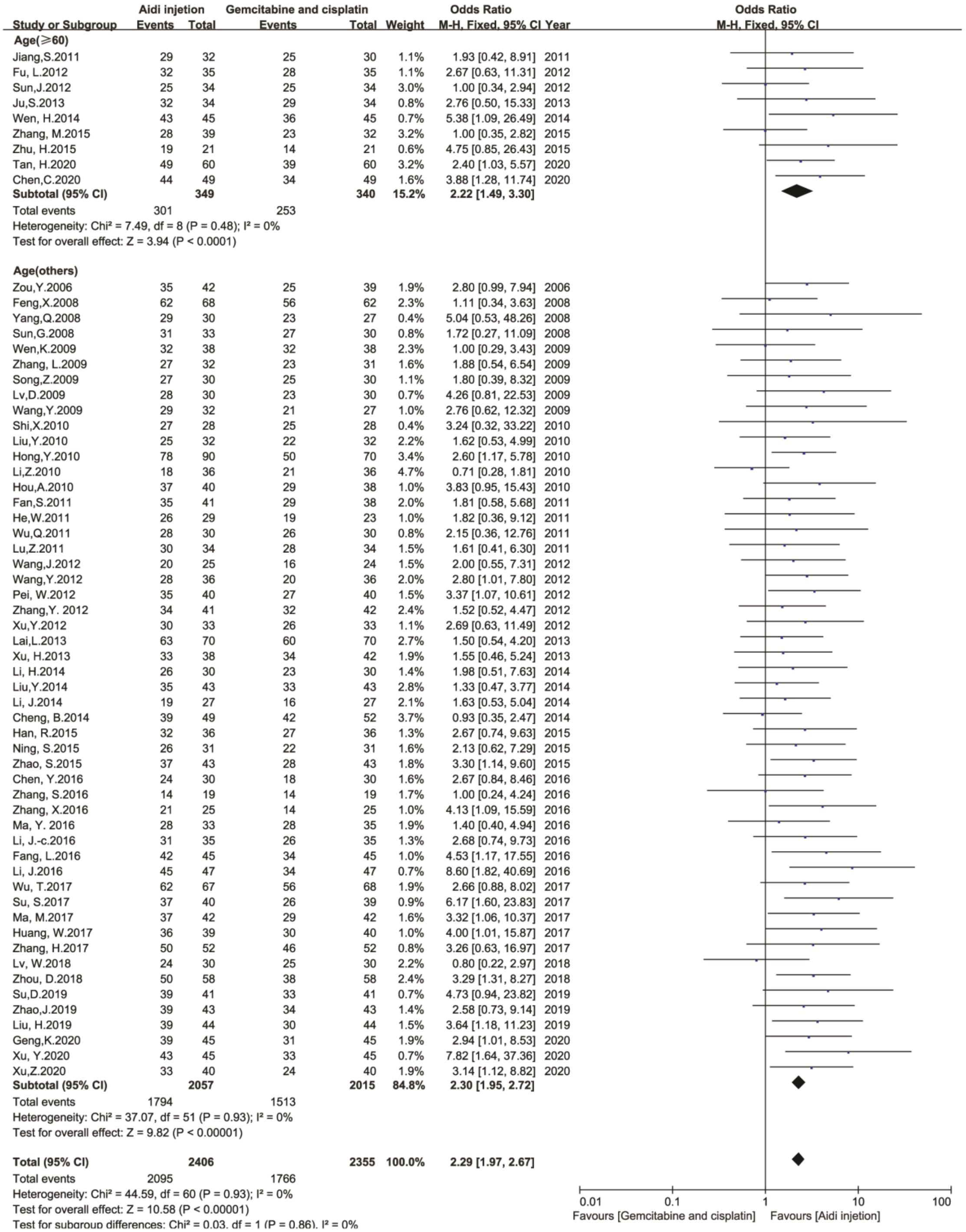

Figure S61 Subgroups analysis of disease control rate by age

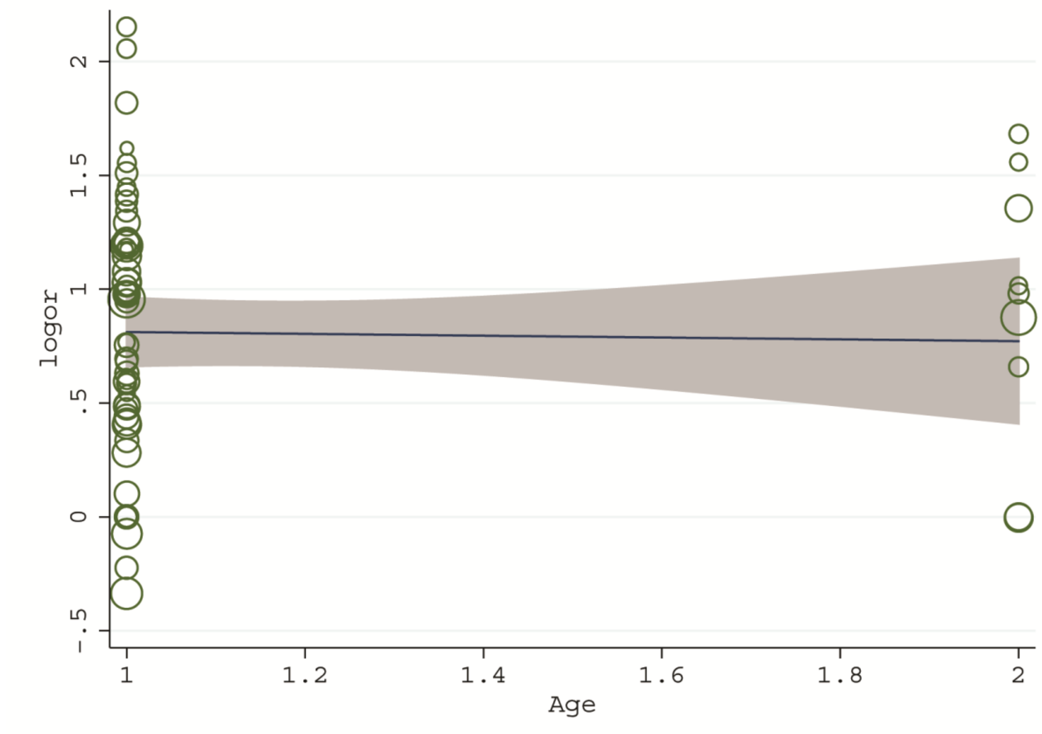

Figure S62 Meta-regression of disease control rate by age

Study or Subgroup	Aidi injection Events	Aidi injection Total	Gemcitabine and cisplatin Events	Gemcitabine and cisplatin Total	Weight	Odds Ratio M-H, Fixed, 95% CI	Year
**Age(≥60)**							
Sun,J.2012	23	34	30	34	2.2%	0.28 [0.08, 0.99]	2012
Fu, L.2012	24	35	32	35	2.3%	0.20 [0.05, 0.81]	2012
Zhang, M.2015	26	39	28	32	2.4%	0.29 [0.08, 0.99]	2015
Zhu, H.2015	6	21	15	21	2.5%	0.16 [0.04, 0.61]	2015
Chen,C.2020	33	49	43	49	3.2%	0.29 [0.10, 0.82]	2020
Tan, H.2020	51	60	57	60	2.0%	0.30 [0.08, 1.16]	2020
**Subtotal (95% CI)**		**238**		**231**	**14.6%**	**0.25 [0.15, 0.42]**	
Total events	163		205				
Heterogeneity: Chi² = 0.71, df = 5 (P = 0.98); I² = 0%							
Test for overall effect: Z = 5.27 (P < 0.00001)							
**Age(others)**							
Feng,X.2008	64	68	60	62	0.9%	0.53 [0.09, 3.02]	2008
Wang,Y.2009	26	32	22	27	1.0%	0.98 [0.26, 3.67]	2009
Zhang, L.2009	5	32	14	31	2.8%	0.22 [0.07, 0.74]	2009
Wen,K.2009	23	38	31	38	2.8%	0.35 [0.12, 0.99]	2009
Hou,A.2010	14	40	22	38	3.4%	0.39 [0.16, 0.98]	2010
Hong,Y.2010	26	90	31	70	5.7%	0.51 [0.27, 0.98]	2010
Lu,Z.2011	11	34	20	34	3.1%	0.33 [0.12, 0.90]	2011
Xu,Y.2012	23	33	33	33	2.4%	0.03 [0.00, 0.60]	2012
Wang,Y.2012	19	36	26	36	2.8%	0.43 [0.16, 1.15]	2012
Zhang,Y. 2012	12	41	17	42	2.7%	0.61 [0.24, 1.52]	2012
Wang,J.2012	7	25	9	24	1.5%	0.65 [0.19, 2.16]	2012
Xu, H.2013	13	38	22	42	3.2%	0.47 [0.19, 1.17]	2013
Liu,Y.2014	27	43	36	43	3.1%	0.33 [0.12, 0.91]	2014
Cheng, B.2014	34	49	47	52	3.2%	0.24 [0.08, 0.73]	2014
Li, J.2014	5	27	6	27	1.1%	0.80 [0.21, 3.00]	2014
Ning, S.2015	16	31	16	31	1.8%	1.00 [0.37, 2.71]	2015
Han, R.2015	7	36	11	36	2.0%	0.55 [0.18, 1.63]	2015
Li, X.2015	2	20	8	20	1.7%	0.17 [0.03, 0.92]	2015
Zhao, S.2015	7	43	10	43	1.9%	0.64 [0.22, 1.88]	2015
Ma, Y. 2016	7	33	15	35	2.7%	0.36 [0.12, 1.05]	2016
Zhang, S.2016	2	19	4	19	0.8%	0.44 [0.07, 2.76]	2016
Li, J.2016	12	47	19	47	3.3%	0.51 [0.21, 1.21]	2016
Li, J.-c.2016	14	35	26	35	3.6%	0.23 [0.08, 0.64]	2016
Fang, L.2016	5	45	13	45	2.7%	0.31 [0.10, 0.95]	2016
Ma, M.2017	11	42	16	42	2.7%	0.58 [0.23, 1.46]	2017
Huang, W.2017	10	39	12	40	2.0%	0.80 [0.30, 2.16]	2017
Su, S.2017	2	40	2	39	0.4%	0.97 [0.13, 7.28]	2017
Zhang, H.2017	13	52	22	52	3.8%	0.45 [0.20, 1.05]	2017
Wu, T.2017	10	67	21	68	4.1%	0.39 [0.17, 0.92]	2017
Wu, Y.2017	3	109	9	109	2.0%	0.31 [0.08, 1.19]	2017
Zhou, D.2018	11	58	13	58	2.4%	0.81 [0.33, 1.99]	2018
Zhang, H.2018	13	30	22	30	2.9%	0.28 [0.09, 0.82]	2018
Guo, X.2020	7	51	16	51	3.2%	0.35 [0.13, 0.94]	2020
Xu,Z.2020	2	40	6	40	1.3%	0.30 [0.06, 1.58]	2020
**Subtotal (95% CI)**		**1463**		**1439**	**85.4%**	**0.44 [0.37, 0.53]**	
Total events	463		657				
Heterogeneity: Chi² = 21.87, df = 33 (P = 0.93); I² = 0%							
Test for overall effect: Z = 9.01 (P < 0.00001)							
**Total (95% CI)**		**1701**		**1670**	**100.0%**	**0.41 [0.35, 0.49]**	
Total events	626		862				
Heterogeneity: Chi² = 26.97, df = 39 (P = 0.93); I² = 0%							
Test for overall effect: Z = 10.32 (P < 0.00001)							
Test for subgroup differences: Chi² = 4.00, df = 1 (P = 0.05), I² = 75.0%							

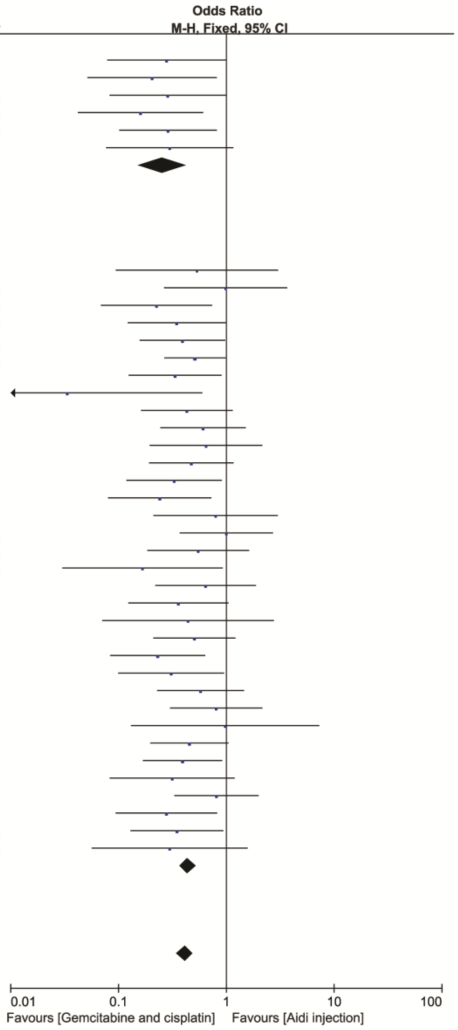

Figure S63 Subgroups analysis of neutropenia by age

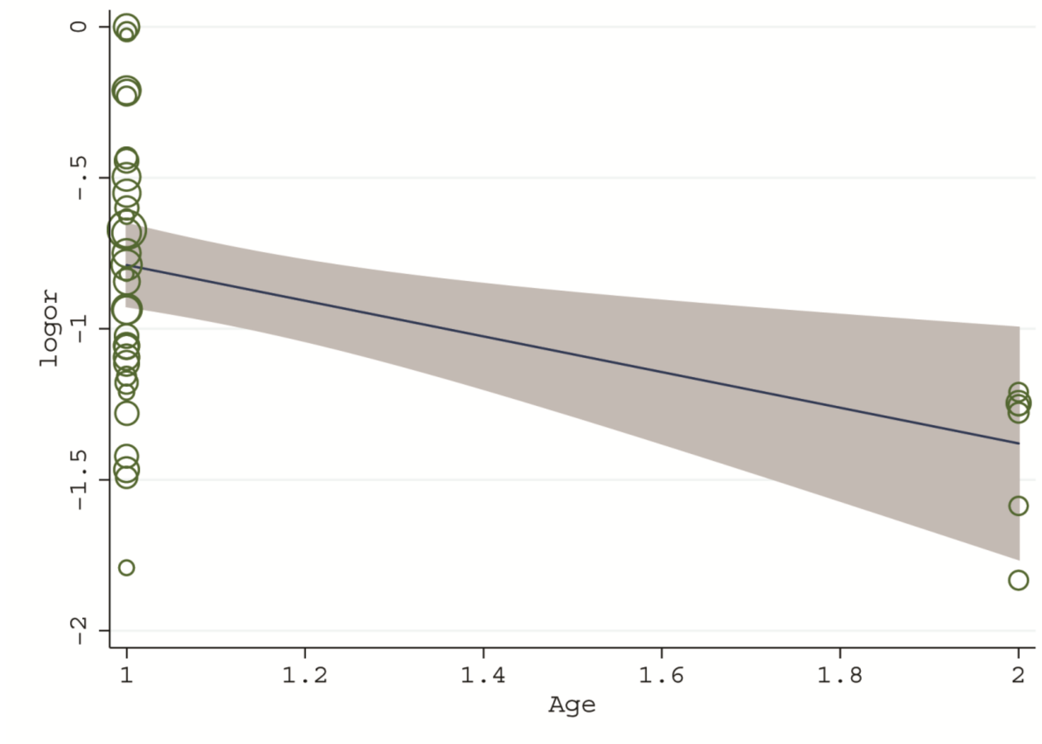

Figure S64 Meta-regression of neutropenia by age

Study or Subgroup	Aidi injetion Events	Aidi injetion Total	Gemcitabine and cisplatin Events	Gemcitabine and cisplatin Total	Weight	Odds Ratio M-H, Fixed, 95% CI	Year
**Age(≥60)**							
Sun,J.2012	28	34	29	34	0.9%	0.80 [0.22, 2.94]	2012
Ju,S.2013	13	34	14	34	1.5%	0.88 [0.33, 2.34]	2013
Wen, H.2014	23	45	39	45	3.3%	0.16 [0.06, 0.45]	2014
Zhang, M.2015	32	39	27	32	0.9%	0.85 [0.24, 2.97]	2015
Zhu, H.2015	7	21	16	21	1.8%	0.16 [0.04, 0.60]	2015
Chen,C.2020	40	49	42	49	1.3%	0.74 [0.25, 2.18]	2020
Tan, H.2020	47	60	54	60	2.0%	0.40 [0.14, 1.14]	2020
**Subtotal (95% CI)**		**282**		**275**	**11.7%**	**0.46 [0.31, 0.69]**	
Total events	190		221				
Heterogeneity: Chi² = 10.54, df = 6 (P = 0.10); I² = 43%							
Test for overall effect: Z = 3.69 (P = 0.0002)							
**Age(others)**							
Zou,Y.2006	32	42	29	39	1.2%	1.10 [0.40, 3.03]	2006
Feng,X.2008	55	68	54	62	1.9%	0.63 [0.24, 1.63]	2008
Wang,Y.2009	5	32	5	27	0.8%	0.81 [0.21, 3.18]	2009
Wen,K.2009	20	38	29	38	2.4%	0.34 [0.13, 0.92]	2009
Lv,D.2009	10	30	19	30	2.2%	0.29 [0.10, 0.84]	2009
Zhang, L.2009	11	32	20	31	2.3%	0.29 [0.10, 0.81]	2009
Song,Z.2009	10	30	11	30	1.3%	0.86 [0.30, 2.50]	2009
Hou,A.2010	5	40	12	38	1.8%	0.31 [0.10, 0.99]	2010
Li,Z.2010	16	36	20	36	1.9%	0.64 [0.25, 1.62]	2010
Shi,X.2010	12	28	20	28	2.0%	0.30 [0.10, 0.91]	2010
Liu,Y.2010	7	32	14	32	1.9%	0.36 [0.12, 1.07]	2010
Hong,Y.2010	20	90	29	70	4.4%	0.40 [0.20, 0.80]	2010
Lu,Z.2011	10	34	19	34	2.3%	0.33 [0.12, 0.90]	2011
He,W.2011	12	29	13	23	1.5%	0.54 [0.18, 1.64]	2011
Xu,Y.2012	7	33	18	33	2.4%	0.22 [0.08, 0.66]	2012
Wang,Y.2012	21	36	28	36	2.0%	0.40 [0.14, 1.12]	2012
Zhang,Y. 2012	9	41	14	42	1.9%	0.56 [0.21, 1.50]	2012
Wang,J.2012	5	25	8	24	1.1%	0.50 [0.14, 1.83]	2012
Xu, H.2013	20	38	30	42	2.3%	0.44 [0.18, 1.12]	2013
Lai,L.2013	20	70	28	70	3.4%	0.60 [0.30, 1.21]	2013
Cai,H.2013	6	19	8	19	0.9%	0.63 [0.17, 2.40]	2013
Li, H.2014	18	30	22	30	1.5%	0.55 [0.18, 1.62]	2014
Cheng, B.2014	7	49	7	52	1.0%	1.07 [0.35, 3.31]	2014
Liu,Y.2014	24	43	32	43	2.4%	0.43 [0.17, 1.08]	2014
Zhao, S.2015	5	43	10	43	1.5%	0.43 [0.13, 1.40]	2015
Li, X.2015	15	20	15	20	0.6%	1.00 [0.24, 4.18]	2015
Ning, S.2015	12	31	16	31	1.7%	0.59 [0.22, 1.62]	2015
Li, J.-c.2016	7	35	19	35	2.6%	0.21 [0.07, 0.61]	2016
Zhang, S.2016	2	19	3	19	0.5%	0.63 [0.09, 4.26]	2016
Ma, Y. 2016	5	33	13	35	1.8%	0.30 [0.09, 0.98]	2016
Fang, L.2016	6	45	14	45	2.1%	0.34 [0.12, 0.99]	2016
Li, J.2016	15	47	27	47	3.2%	0.35 [0.15, 0.81]	2016
Wu, T.2017	11	67	23	68	3.3%	0.38 [0.17, 0.87]	2017
Zhang, H.2017	16	52	29	52	3.4%	0.35 [0.16, 0.79]	2017
Su, S.2017	4	40	3	39	0.5%	1.33 [0.28, 6.39]	2017
Wu, Y.2017	37	109	63	109	7.1%	0.38 [0.22, 0.65]	2017
Zhang, H.2018	7	40	18	40	2.5%	0.26 [0.09, 0.72]	2018
Zhou, D.2018	29	58	31	58	2.7%	0.87 [0.42, 1.81]	2018
Su,D.2019	5	41	15	41	2.3%	0.24 [0.08, 0.75]	2019
Geng,K.2020	21	45	30	45	2.7%	0.44 [0.19, 1.03]	2020
Guo, X.2020	5	51	13	51	2.0%	0.32 [0.10, 0.97]	2020
Xu,Z.2020	2	40	7	40	1.1%	0.25 [0.05, 1.28]	2020
**Subtotal (95% CI)**		**1761**		**1727**	**88.3%**	**0.44 [0.38, 0.52]**	
Total events	566		838				
Heterogeneity: Chi² = 28.39, df = 41 (P = 0.93); I² = 0%							
Test for overall effect: Z = 10.58 (P < 0.00001)							
**Total (95% CI)**		**2043**		**2002**	**100.0%**	**0.45 [0.39, 0.51]**	
Total events	756		1059				
Heterogeneity: Chi² = 38.98, df = 48 (P = 0.82); I² = 0%							
Test for overall effect: Z = 11.21 (P < 0.00001)							
Test for subgroup differences: Chi² = 0.03, df = 1 (P = 0.87), I² = 0%							

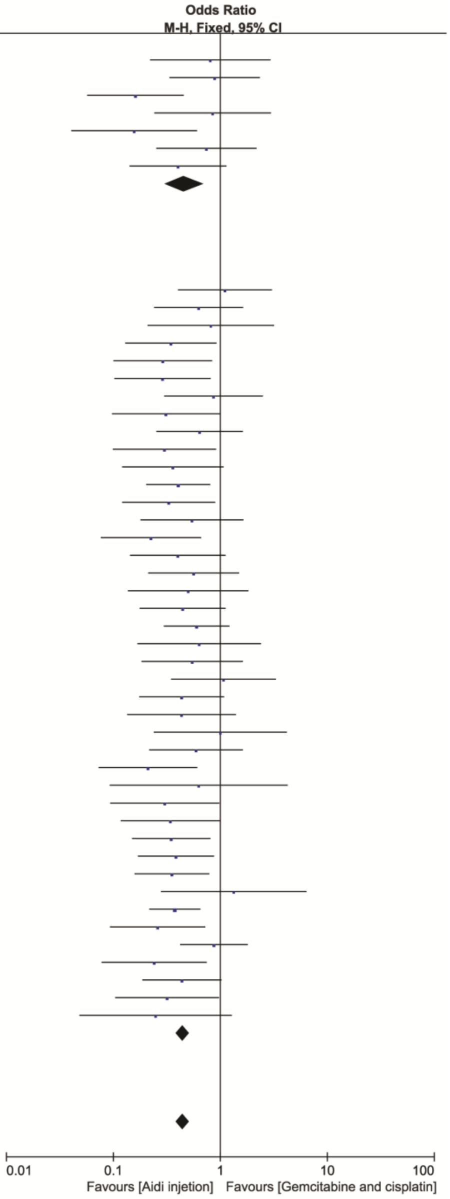

Figure S65 Subgroups analysis of gastrointestinal reaction by age

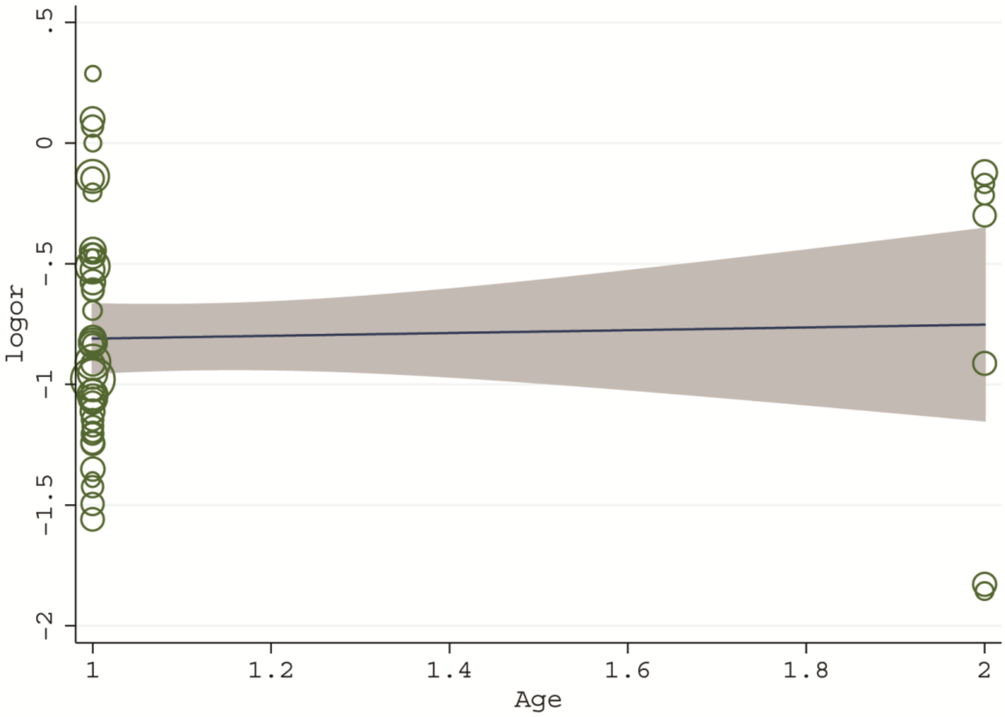

Figure S66 Meta-regression of gastrointestinal reaction by age

Study or Subgroup	Aidi injection Events	Aidi injection Total	Gemcitabine and cisplatin Events	Gemcitabine and cisplatin Total	Weight	Odds Ratio M-H, Fixed, 95% CI	Year
**Aidi injection(50-60ml)**							
Feng,X.2008	30	68	25	62	3.5%	1.17 [0.58, 2.35]	2008
Song,Z.2009	10	30	9	30	1.4%	1.17 [0.39, 3.47]	2009
Wen,K.2009	16	38	15	38	2.1%	1.12 [0.45, 2.79]	2009
Liu,Y.2010	16	32	10	32	1.2%	2.20 [0.79, 6.10]	2010
Hou,A.2010	16	40	14	38	2.0%	1.14 [0.46, 2.85]	2010
Hong,Y.2010	57	90	32	70	3.1%	2.05 [1.09, 3.88]	2010
Shi,X.2010	13	28	10	28	1.3%	1.56 [0.53, 4.56]	2010
Fan,S.2011	22	41	12	38	1.4%	2.51 [1.00, 6.29]	2011
Ding,P.2011	9	18	10	22	1.1%	1.20 [0.34, 4.18]	2011
Pei, W.2012	15	40	11	40	1.6%	1.58 [0.62, 4.07]	2012
Fu, L.2012	19	35	16	35	1.7%	1.41 [0.55, 3.61]	2012
Sun,J.2012	15	34	13	34	1.7%	1.28 [0.48, 3.36]	2012
Wang,J.2012	14	25	10	24	1.1%	1.78 [0.57, 5.53]	2012
Zhang,Y. 2012	21	41	15	42	1.7%	1.89 [0.78, 4.55]	2012
Xu, H.2013	19	38	18	42	2.0%	1.33 [0.55, 3.22]	2013
Lai,L.2013	45	70	40	70	3.4%	1.35 [0.68, 2.67]	2013
Ju,S.2013	19	34	12	34	1.3%	2.32 [0.87, 6.16]	2013
Liu,Y.2014	18	43	17	43	2.3%	1.10 [0.47, 2.60]	2014
Li, J.2014	11	27	10	27	1.4%	1.17 [0.39, 3.49]	2014
Li, H.2014	16	30	12	30	1.3%	1.71 [0.62, 4.77]	2014
Wen, H.2014	28	45	17	45	1.5%	2.71 [1.16, 6.36]	2014
Zhang, M.2015	17	39	12	32	1.8%	1.29 [0.50, 3.35]	2015
Ning, S.2015	19	31	11	31	1.0%	2.88 [1.03, 8.07]	2015
Han, R.2015	14	36	10	36	1.4%	1.65 [0.61, 4.45]	2015
Zhang, S.2016	11	19	8	19	0.8%	1.89 [0.52, 6.85]	2016
Ma, Y. 2016	19	33	19	35	1.9%	1.14 [0.44, 2.98]	2016
Fang, L.2016	35	45	27	45	1.4%	2.33 [0.93, 5.87]	2016
Zhang, X.2016	18	25	8	25	0.5%	5.46 [1.63, 18.36]	2016
Su, S.2017	35	40	23	39	0.7%	4.87 [1.57, 15.13]	2017
Ma, M.2017	19	42	12	42	1.6%	2.07 [0.84, 5.10]	2017
Huang, W.2017	17	39	11	40	1.5%	2.04 [0.80, 5.21]	2017
Lv, W.2018	16	30	15	30	1.7%	1.14 [0.41, 3.15]	2018
Zhou, D.2018	30	58	22	58	2.5%	1.75 [0.84, 3.67]	2018
Liu, H.2019	19	44	12	44	1.6%	2.03 [0.83, 4.95]	2019
Zhao,J.2019	31	43	22	43	1.5%	2.47 [1.01, 6.04]	2019
Geng,K.2020	27	45	17	45	1.6%	2.47 [1.06, 5.77]	2020
Xu, Y.2020	16	45	12	45	1.8%	1.52 [0.62, 3.73]	2020
Tan, H.2020	24	60	18	60	2.6%	1.56 [0.73, 3.31]	2020
**Subtotal (95% CI)**		**1521**		**1493**	**63.8%**	**1.72 [1.49, 2.00]**	
Total events	796		587				
Heterogeneity: Chi² = 20.91, df = 37 (P = 0.98); I² = 0%							
Test for overall effect: Z = 7.23 (P < 0.00001)							
**Aidi injection(70-80ml)**							
Zou,Y.2006	24	42	13	39	1.4%	2.67 [1.08, 6.58]	2006
Yang,Q.2008	23	30	11	27	0.6%	4.78 [1.52, 14.98]	2008
Zhang, L.2009	15	32	14	31	1.8%	1.07 [0.40, 2.89]	2009
Lv,D.2009	22	30	11	30	0.7%	4.75 [1.58, 14.25]	2009
Xu,Y.2012	16	33	16	33	2.0%	1.00 [0.38, 2.63]	2012
Wang,Y.2012	16	36	14	36	1.8%	1.26 [0.49, 3.21]	2012
Chen, Y.2016	15	30	13	30	1.5%	1.31 [0.47, 3.61]	2016
**Subtotal (95% CI)**		**233**		**226**	**9.8%**	**1.85 [1.28, 2.68]**	
Total events	131		92				
Heterogeneity: Chi² = 9.94, df = 6 (P = 0.13); I² = 40%							
Test for overall effect: Z = 3.27 (P = 0.001)							
**Aidi injection(90-100ml)**							
Sun,G.2008	17	33	15	30	1.8%	1.06 [0.40, 2.86]	2008
Lu,Z.2011	18	34	17	34	1.9%	1.13 [0.43, 2.91]	2011
Wu,Q.2011	17	30	11	30	1.1%	2.26 [0.80, 6.36]	2011
Jiang,S.2011	15	32	12	30	1.6%	1.32 [0.48, 3.63]	2011
Zhao, S.2015	19	43	12	43	1.6%	2.05 [0.83, 5.02]	2015
Zhu, H.2015	15	21	7	21	0.5%	5.00 [1.35, 18.55]	2015
Li, J.-c.2016	21	35	15	35	1.4%	2.00 [0.77, 5.18]	2016
Wu, T.2017	53	67	40	68	2.0%	2.65 [1.24, 5.68]	2017
**Subtotal (95% CI)**		**295**		**291**	**11.8%**	**1.89 [1.35, 2.64]**	
Total events	175		129				
Heterogeneity: Chi² = 5.95, df = 7 (P = 0.55); I² = 0%							
Test for overall effect: Z = 3.71 (P = 0.0002)							
**Aidi injection(others)**							
Zhao,H.2008	11	30	6	20	1.1%	1.35 [0.40, 4.53]	2008
Wang,Y.2009	17	32	12	27	1.4%	1.42 [0.51, 3.96]	2009
Li,Z.2010	16	36	12	36	1.6%	1.60 [0.62, 4.16]	2010
He,W.2011	15	29	10	23	1.3%	1.39 [0.46, 4.18]	2011
Cheng, B.2014	12	49	16	52	2.8%	0.73 [0.30, 1.76]	2014
Li, J.2016	23	47	9	47	1.1%	4.05 [1.61, 10.20]	2016
Zhang, H.2017	42	52	26	52	1.2%	4.20 [1.75, 10.11]	2017
Su,D.2019	30	41	17	41	1.1%	3.85 [1.52, 9.75]	2019
Chen,C.2020	26	49	16	49	1.8%	2.33 [1.03, 5.29]	2020
Xu,Z.2020	25	40	14	40	1.2%	3.10 [1.24, 7.71]	2020
**Subtotal (95% CI)**		**405**		**387**	**14.5%**	**2.16 [1.61, 2.89]**	
Total events	217		138				
Heterogeneity: Chi² = 14.17, df = 9 (P = 0.12); I² = 37%							
Test for overall effect: Z = 5.15 (P < 0.00001)							
**Total (95% CI)**		**2454**		**2397**	**100.0%**	**1.82 [1.62, 2.04]**	
Total events	1319		946				
Heterogeneity: Chi² = 52.84, df = 62 (P = 0.79); I² = 0%							
Test for overall effect: Z = 10.08 (P < 0.00001)							
Test for subgroup differences: Chi² = 1.89, df = 3 (P = 0.60), I² = 0%							

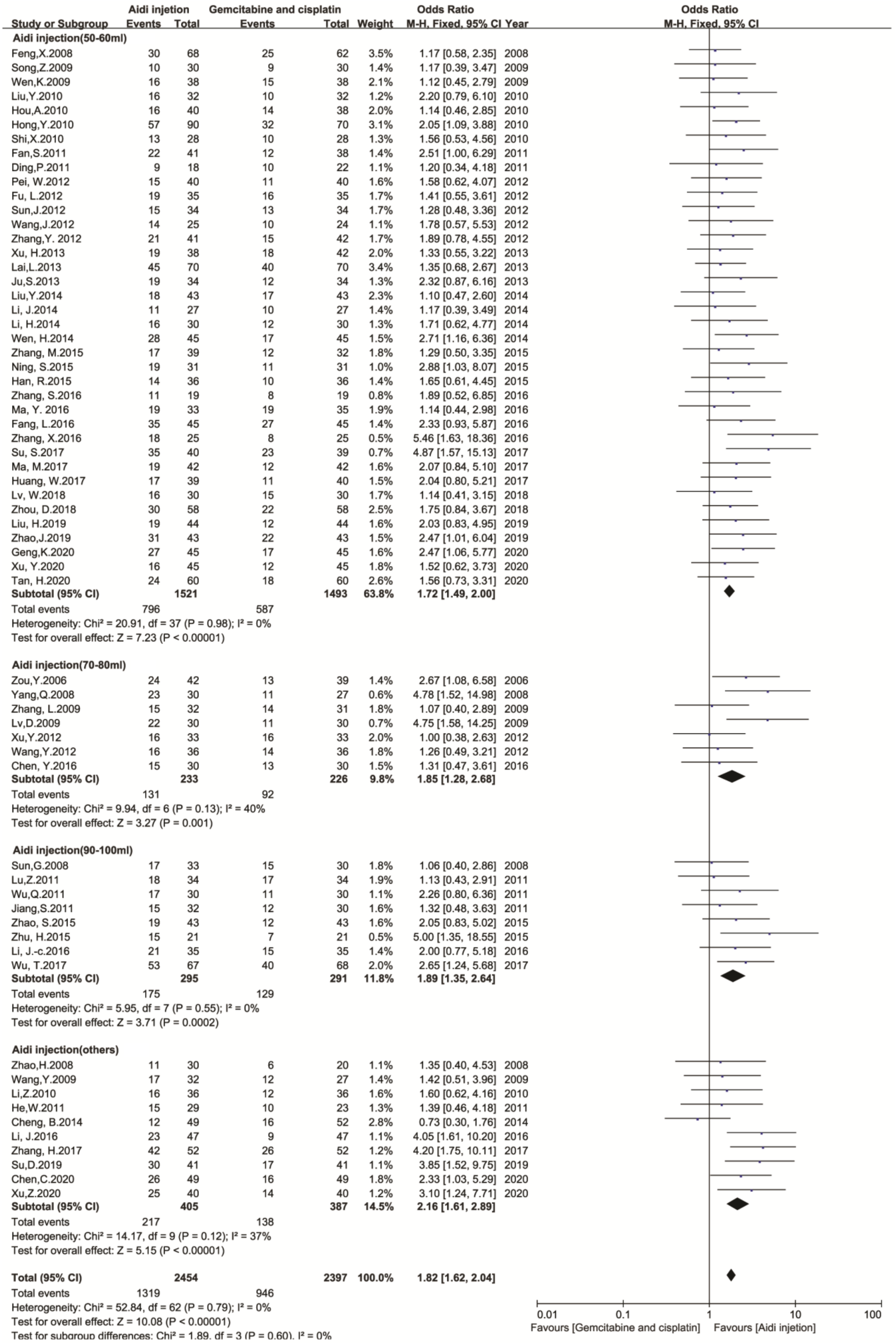

Figure S67 Subgroups analysis of objective response rate by dosage of Aidi injection

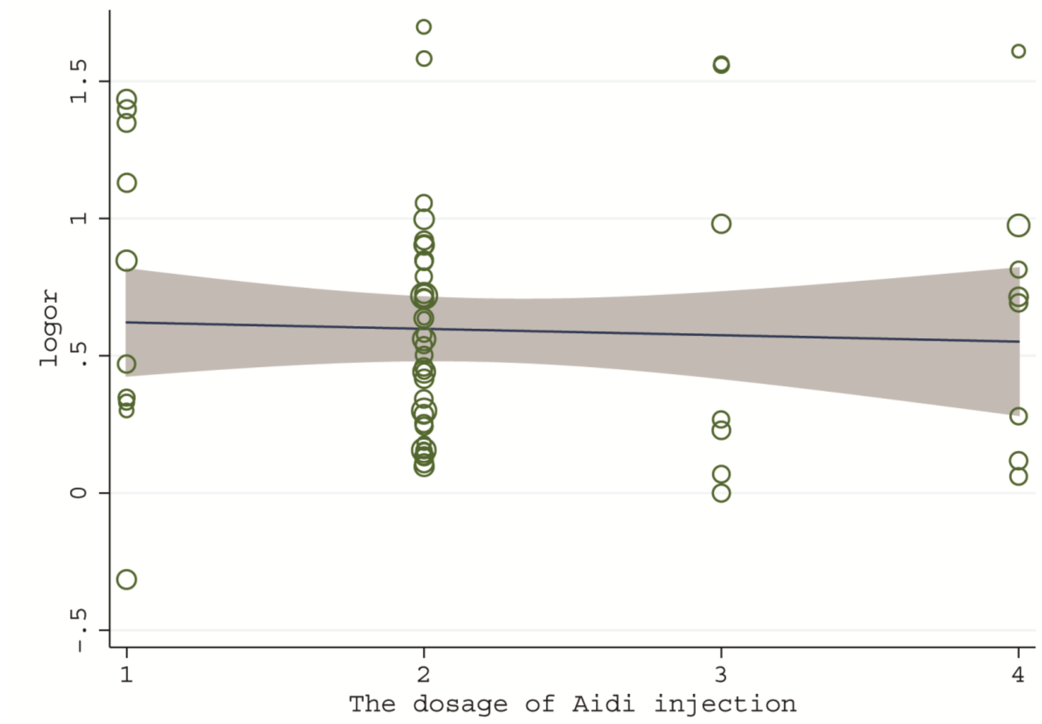

Figure S68 Meta-regression of objective response rate by dosage of Aidi injection

Study or Subgroup	Aidi injetion Events	Aidi injetion Total	Gemcitabine and cisplatin Events	Gemcitabine and cisplatin Total	Weight	Odds Ratio M-H, Fixed, 95% CI	Year
**Aidi injection(50-60ml)**							
Feng,X.2008	62	68	56	62	2.3%	1.11 [0.34, 3.63]	2008
Song,Z.2009	27	30	25	30	1.1%	1.80 [0.39, 8.32]	2009
Wen,K.2009	32	38	32	38	2.3%	1.00 [0.29, 3.43]	2009
Hong,Y.2010	78	90	50	70	3.4%	2.60 [1.17, 5.78]	2010
Hou,A.2010	37	40	29	38	1.0%	3.83 [0.95, 15.43]	2010
Liu,Y.2010	25	32	22	32	2.2%	1.62 [0.53, 4.99]	2010
Shi,X.2010	27	28	25	28	0.4%	3.24 [0.32, 33.22]	2010
Fan,S.2011	35	41	29	38	2.0%	1.81 [0.58, 5.68]	2011
Pei, W.2012	35	40	27	40	1.5%	3.37 [1.07, 10.61]	2012
Sun,J.2012	25	34	25	34	3.0%	1.00 [0.34, 2.94]	2012
Wang,J.2012	20	25	16	24	1.5%	2.00 [0.55, 7.31]	2012
Zhang,Y. 2012	34	41	32	42	2.4%	1.52 [0.52, 4.47]	2012
Fu, L.2012	32	35	28	35	1.1%	2.67 [0.63, 11.31]	2012
Xu, H.2013	33	38	34	42	1.9%	1.55 [0.46, 5.24]	2013
Lai,L.2013	63	70	60	70	2.7%	1.50 [0.54, 4.20]	2013
Ju,S.2013	32	34	29	34	0.8%	2.76 [0.50, 15.33]	2013
Liu,Y.2014	35	43	33	43	2.8%	1.33 [0.47, 3.77]	2014
Li, J.2014	19	27	16	27	2.1%	1.63 [0.53, 5.04]	2014
Wen, H.2014	43	45	36	45	0.7%	5.38 [1.09, 26.49]	2014
Li, H.2014	26	30	23	30	1.4%	1.98 [0.51, 7.63]	2014
Zhang, M.2015	28	39	23	32	3.2%	1.00 [0.35, 2.82]	2015
Ning, S.2015	26	31	22	31	1.6%	2.13 [0.62, 7.29]	2015
Han, R.2015	32	36	27	36	1.3%	2.67 [0.74, 9.63]	2015
Ma, Y. 2016	28	33	28	35	1.9%	1.40 [0.40, 4.94]	2016
Zhang, S.2016	14	19	14	19	1.7%	1.00 [0.24, 4.24]	2016
Fang, L.2016	42	45	34	45	1.0%	4.53 [1.17, 17.55]	2016
Zhang, X.2016	21	25	14	25	1.0%	4.13 [1.09, 15.59]	2016
Ma, M.2017	37	42	29	42	1.6%	3.32 [1.06, 10.37]	2017
Su, S.2017	37	40	26	39	0.9%	6.17 [1.60, 23.83]	2017
Huang, W.2017	36	39	30	40	1.0%	4.00 [1.01, 15.87]	2017
Lv, W.2018	24	30	25	30	2.2%	0.80 [0.22, 2.97]	2018
Zhou, D.2018	50	58	38	58	2.4%	3.29 [1.31, 8.27]	2018
Zhao,J.2019	39	43	34	43	1.4%	2.58 [0.73, 9.14]	2019
Liu, H.2019	39	44	30	44	1.5%	3.64 [1.18, 11.23]	2019
Tan, H.2020	49	60	39	60	3.2%	2.40 [1.03, 5.57]	2020
Xu, Y.2020	43	45	33	45	0.7%	7.82 [1.64, 37.36]	2020
Geng,K.2020	39	45	31	45	1.9%	2.94 [1.01, 8.53]	2020
**Subtotal (95% CI)**		**1503**		**1471**	**65.0%**	**2.21 [1.82, 2.68]**	
Total events	1304		1104				
Heterogeneity: Chi² = 26.71, df = 36 (P = 0.87); I² = 0%							
Test for overall effect: Z = 8.09 (P < 0.00001)							
**Aidi injection(70-80ml)**							
Zou,Y.2006	35	42	25	39	1.9%	2.80 [0.99, 7.94]	2006
Yang,Q.2008	29	30	23	27	0.4%	5.04 [0.53, 48.26]	2008
Lv,D.2009	28	30	23	30	0.7%	4.26 [0.81, 22.53]	2009
Zhang, L.2009	27	32	23	31	1.6%	1.88 [0.54, 6.54]	2009
Wang,Y.2012	28	36	20	36	2.0%	2.80 [1.01, 7.80]	2012
Xu,Y.2012	30	33	26	33	1.1%	2.69 [0.63, 11.49]	2012
Chen, Y.2016	24	30	18	30	1.6%	2.67 [0.84, 8.46]	2016
**Subtotal (95% CI)**		**233**		**226**	**9.3%**	**2.80 [1.73, 4.52]**	
Total events	201		158				
Heterogeneity: Chi² = 0.91, df = 6 (P = 0.99); I² = 0%							
Test for overall effect: Z = 4.20 (P < 0.0001)							
**Aidi injection(90-100ml)**							
Sun,G.2008	31	33	27	30	0.8%	1.72 [0.27, 11.09]	2008
Lu,Z.2011	30	34	28	34	1.5%	1.61 [0.41, 6.30]	2011
Wu,Q.2011	28	30	26	30	0.8%	2.15 [0.36, 12.76]	2011
Jiang,S.2011	29	32	25	30	1.1%	1.93 [0.42, 8.91]	2011
Zhao, S.2015	37	43	28	43	1.8%	3.30 [1.14, 9.60]	2015
Zhu, H.2015	19	21	14	21	0.6%	4.75 [0.85, 26.43]	2015
Li, J.-c.2016	31	35	26	35	1.3%	2.68 [0.74, 9.73]	2016
Wu, T.2017	62	67	56	68	1.9%	2.66 [0.88, 8.02]	2017
**Subtotal (95% CI)**		**295**		**291**	**9.7%**	**2.55 [1.57, 4.14]**	
Total events	267		230				
Heterogeneity: Chi² = 1.51, df = 7 (P = 0.98); I² = 0%							
Test for overall effect: Z = 3.79 (P = 0.0001)							
**Aidi injection(others)**							
Wang,Y.2009	29	32	21	27	1.0%	2.76 [0.62, 12.32]	2009
Li,Z.2010	18	36	21	36	4.7%	0.71 [0.28, 1.81]	2010
He,W.2011	26	29	19	23	1.0%	1.82 [0.36, 9.12]	2011
Cheng, B.2014	39	49	42	52	3.7%	0.93 [0.35, 2.47]	2014
Li, J.2016	45	47	34	47	0.7%	8.60 [1.82, 40.69]	2016
Zhang, H.2017	50	52	46	52	0.8%	3.26 [0.63, 16.97]	2017
Su,D.2019	39	41	33	41	0.7%	4.73 [0.94, 23.82]	2019
Chen,C.2020	44	49	34	49	1.6%	3.88 [1.28, 11.74]	2020
Xu,Z.2020	33	40	24	40	1.9%	3.14 [1.12, 8.82]	2020
**Subtotal (95% CI)**		**375**		**367**	**16.0%**	**2.18 [1.48, 3.21]**	
Total events	323		274				
Heterogeneity: Chi² = 14.22, df = 8 (P = 0.08); I² = 44%							
Test for overall effect: Z = 3.92 (P < 0.0001)							
**Total (95% CI)**		**2406**		**2355**	**100.0%**	**2.29 [1.97, 2.67]**	
Total events	2095		1766				
Heterogeneity: Chi² = 44.59, df = 60 (P = 0.93); I² = 0%							
Test for overall effect: Z = 10.58 (P < 0.00001)							
Test for subgroup differences: Chi² = 1.06, df = 3 (P = 0.79), I² = 0%							

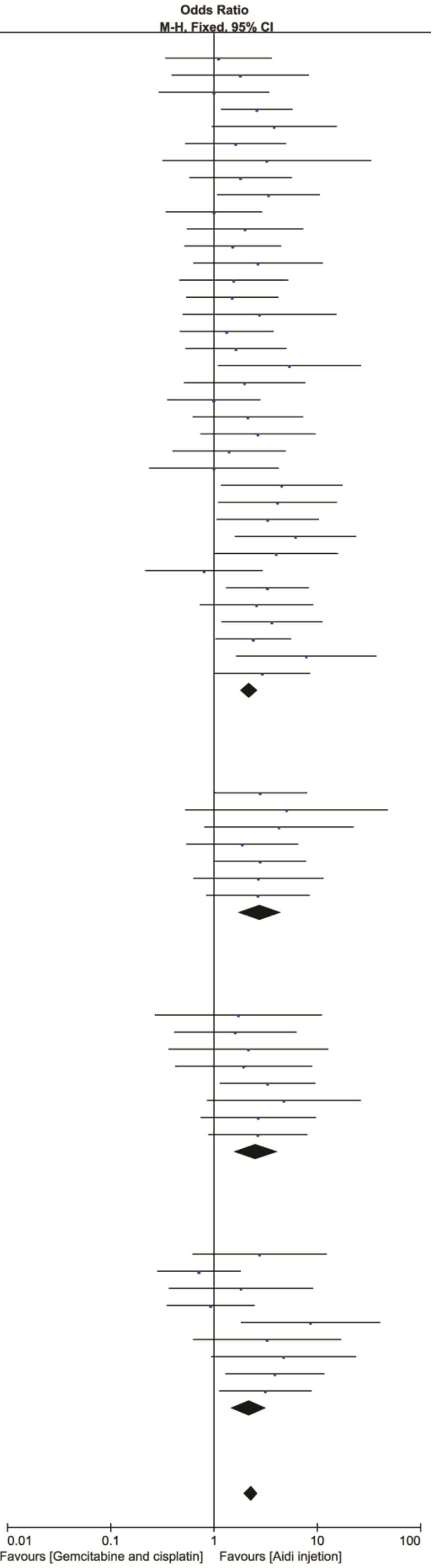

Figure S69 Subgroups analysis of disease control rate by dosage of Aidi injection

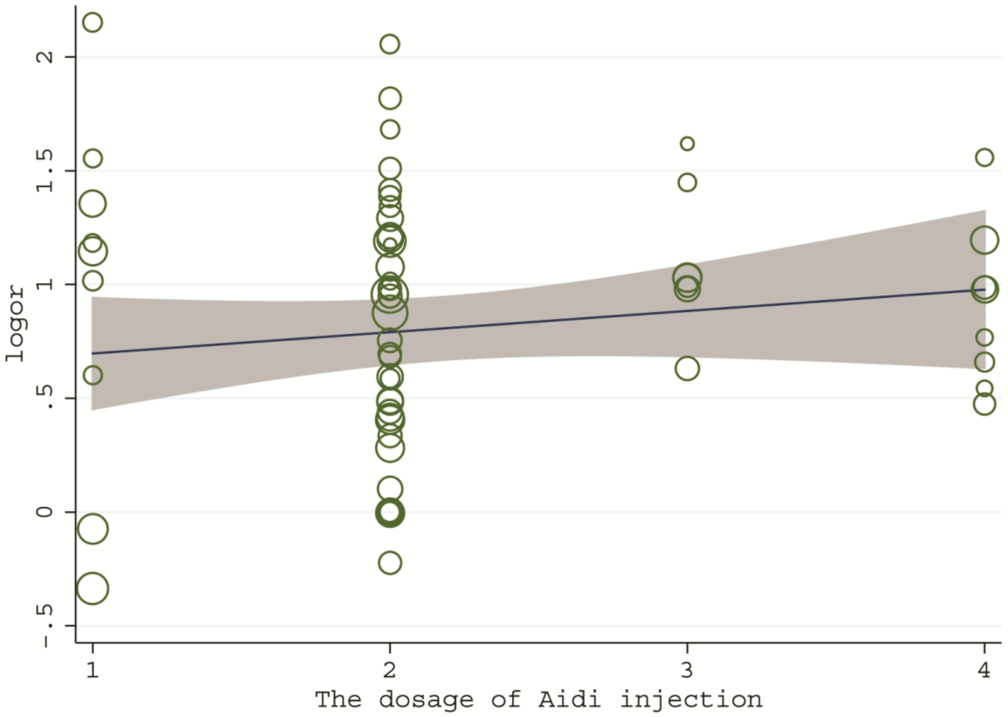

Figure S70 Meta-regression of disease control rate by dosage of Aidi injection

Study or Subgroup	Aidi injection Events	Aidi injection Total	Gemcitabine and cisplatin Events	Gemcitabine and cisplatin Total	Weight	Odds Ratio M-H, Fixed, 95% CI	Year
**Aidi injection(50-60ml)**							
Feng,X.2008	64	68	60	62	0.9%	0.53 [0.09, 3.02]	2008
Wen,K.2009	23	38	31	38	2.8%	0.35 [0.12, 0.99]	2009
Hong,Y.2010	26	90	31	70	5.7%	0.51 [0.27, 0.98]	2010
Hou,A.2010	14	40	22	38	3.4%	0.39 [0.16, 0.98]	2010
Wang,J.2012	7	25	9	24	1.5%	0.65 [0.19, 2.16]	2012
Zhang,Y. 2012	12	41	17	42	2.7%	0.61 [0.24, 1.52]	2012
Fu, L.2012	24	35	32	35	2.3%	0.20 [0.05, 0.81]	2012
Sun,J.2012	23	34	30	34	2.2%	0.28 [0.08, 0.99]	2012
Xu, H.2013	13	38	22	42	3.2%	0.47 [0.19, 1.17]	2013
Liu,Y.2014	27	43	36	43	3.1%	0.33 [0.12, 0.91]	2014
Li, J.2014	5	27	6	27	1.1%	0.80 [0.21, 3.00]	2014
Han, R.2015	7	36	11	36	2.0%	0.55 [0.18, 1.63]	2015
Li, X.2015	2	20	8	20	1.7%	0.17 [0.03, 0.92]	2015
Zhang, M.2015	26	39	28	32	2.4%	0.29 [0.08, 0.99]	2015
Ning, S.2015	16	31	16	31	1.8%	1.00 [0.37, 2.71]	2015
Fang, L.2016	5	45	13	45	2.7%	0.31 [0.10, 0.95]	2016
Zhang, S.2016	2	19	4	19	0.8%	0.44 [0.07, 2.76]	2016
Ma, Y. 2016	7	33	15	35	2.7%	0.36 [0.12, 1.05]	2016
Su, S.2017	2	40	2	39	0.4%	0.97 [0.13, 7.28]	2017
Wu, Y.2017	3	109	9	109	2.0%	0.31 [0.08, 1.19]	2017
Ma, M.2017	11	42	16	42	2.7%	0.58 [0.23, 1.46]	2017
Huang, W.2017	10	39	12	40	2.0%	0.80 [0.30, 2.16]	2017
Zhou, D.2018	11	58	13	58	2.4%	0.81 [0.33, 1.99]	2018
Zhang, H.2018	13	30	22	30	2.9%	0.28 [0.09, 0.82]	2018
Tan, H.2020	51	60	57	60	2.0%	0.30 [0.08, 1.16]	2020
Guo, X.2020	7	51	16	51	3.2%	0.35 [0.13, 0.94]	2020
**Subtotal (95% CI)**		**1131**		**1102**	**60.8%**	**0.45 [0.37, 0.56]**	
Total events	411		538				
Heterogeneity: Chi² = 14.60, df = 25 (P = 0.95); I² = 0%							
Test for overall effect: Z = 7.35 (P < 0.00001)							
**Aidi injection(70-80ml)**							
Zhang, L.2009	5	32	14	31	2.8%	0.22 [0.07, 0.74]	2009
Wang,Y.2012	19	36	26	36	2.8%	0.43 [0.16, 1.15]	2012
Xu,Y.2012	23	33	33	33	2.4%	0.03 [0.00, 0.60]	2012
**Subtotal (95% CI)**		**101**		**100**	**8.0%**	**0.24 [0.12, 0.48]**	
Total events	47		73				
Heterogeneity: Chi² = 3.16, df = 2 (P = 0.21); I² = 37%							
Test for overall effect: Z = 4.01 (P < 0.0001)							
**Aidi injection(90-100ml)**							
Lu,Z.2011	11	34	20	34	3.1%	0.33 [0.12, 0.90]	2011
Zhu, H.2015	6	21	15	21	2.5%	0.16 [0.04, 0.61]	2015
Zhao, S.2015	7	43	10	43	1.9%	0.64 [0.22, 1.88]	2015
Li, J.-c.2016	14	35	26	35	3.6%	0.23 [0.08, 0.64]	2016
Wu, T.2017	10	67	21	68	4.1%	0.39 [0.17, 0.92]	2017
**Subtotal (95% CI)**		**200**		**201**	**15.3%**	**0.34 [0.21, 0.53]**	
Total events	48		92				
Heterogeneity: Chi² = 3.23, df = 4 (P = 0.52); I² = 0%							
Test for overall effect: Z = 4.72 (P < 0.00001)							
**Aidi injection(others)**							
Wang,Y.2009	26	32	22	27	1.0%	0.98 [0.26, 3.67]	2009
Cheng, B.2014	34	49	47	52	3.2%	0.24 [0.08, 0.73]	2014
Li, J.2016	12	47	19	47	3.3%	0.51 [0.21, 1.21]	2016
Zhang, H.2017	13	52	22	52	3.8%	0.45 [0.20, 1.05]	2017
Chen,C.2020	33	49	43	49	3.2%	0.29 [0.10, 0.82]	2020
Xu,Z.2020	2	40	6	40	1.3%	0.30 [0.06, 1.58]	2020
**Subtotal (95% CI)**		**269**		**267**	**15.9%**	**0.41 [0.27, 0.63]**	
Total events	120		159				
Heterogeneity: Chi² = 3.45, df = 5 (P = 0.63); I² = 0%							
Test for overall effect: Z = 4.13 (P < 0.0001)							
**Total (95% CI)**		**1701**		**1670**	**100.0%**	**0.41 [0.35, 0.49]**	
Total events	626		862				
Heterogeneity: Chi² = 26.97, df = 39 (P = 0.93); I² = 0%							
Test for overall effect: Z = 10.32 (P < 0.00001)							
Test for subgroup differences: Chi² = 3.91, df = 3 (P = 0.27), I² = 23.3%							

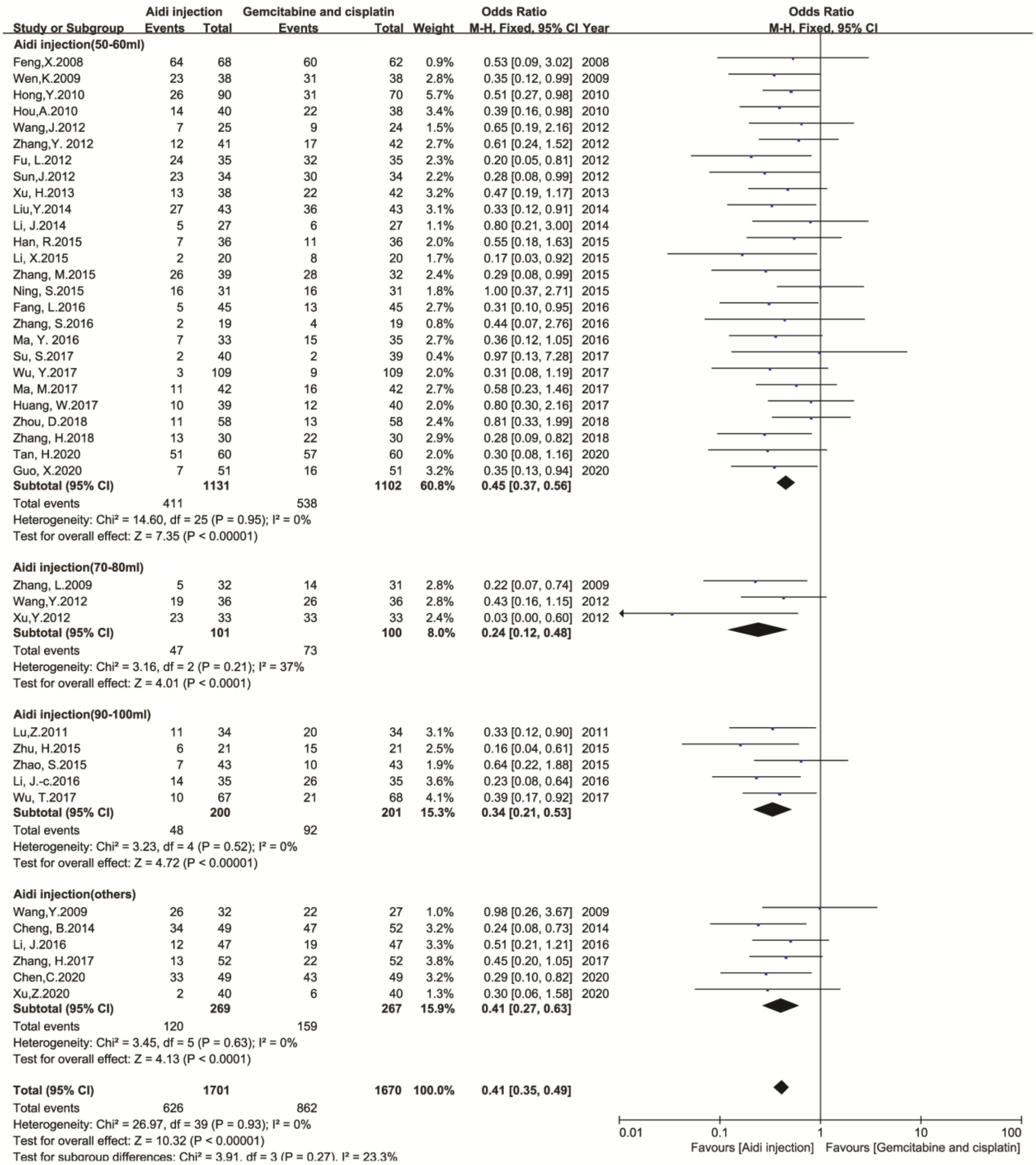

Figure S71 Subgroups analysis of neutropenia by dosage of Aidi injection

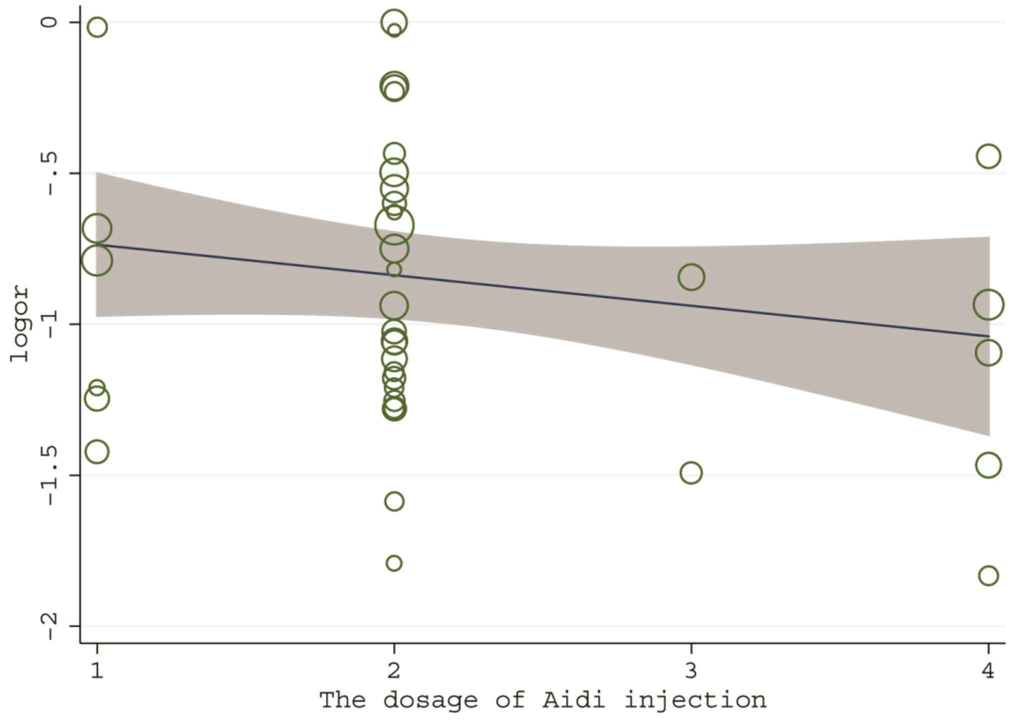

Figure S72 Meta-regression of neutropenia by dosage of Aidi injection

Study or Subgroup	Aidi injetion Events	Aidi injetion Total	Gemcitabine and cisplatin Events	Gemcitabine and cisplatin Total	Weight	Odds Ratio M-H, Fixed, 95% CI	Year
**Aidi injection(50-60ml)**							
Feng,X.2008	55	68	54	62	1.9%	0.63 [0.24, 1.63]	2008
Wen,K.2009	20	38	29	38	2.4%	0.34 [0.13, 0.92]	2009
Song,Z.2009	10	30	11	30	1.3%	0.86 [0.30, 2.50]	2009
Hou,A.2010	5	40	12	38	1.8%	0.31 [0.10, 0.99]	2010
Hong,Y.2010	20	90	29	70	4.4%	0.40 [0.20, 0.80]	2010
Liu,Y.2010	7	32	14	32	1.9%	0.36 [0.12, 1.07]	2010
Shi,X.2010	12	28	20	28	2.0%	0.30 [0.10, 0.91]	2010
Zhang,Y. 2012	9	41	14	42	1.9%	0.56 [0.21, 1.50]	2012
Sun,J.2012	28	34	29	34	0.9%	0.80 [0.22, 2.94]	2012
Wang,J.2012	5	25	8	24	1.1%	0.50 [0.14, 1.83]	2012
Ju,S.2013	13	34	14	34	1.5%	0.88 [0.33, 2.34]	2013
Xu, H.2013	20	38	30	42	2.3%	0.44 [0.18, 1.12]	2013
Lai,L.2013	20	70	28	70	3.4%	0.60 [0.30, 1.21]	2013
Li, H.2014	18	30	22	30	1.5%	0.55 [0.18, 1.62]	2014
Wen, H.2014	23	45	39	45	3.3%	0.16 [0.06, 0.45]	2014
Liu,Y.2014	24	43	32	43	2.4%	0.43 [0.17, 1.08]	2014
Ning, S.2015	12	31	16	31	1.7%	0.59 [0.22, 1.62]	2015
Li, X.2015	15	20	15	20	0.6%	1.00 [0.24, 4.18]	2015
Zhang, M.2015	32	39	27	32	0.9%	0.85 [0.24, 2.97]	2015
Fang, L.2016	6	45	14	45	2.1%	0.34 [0.12, 0.99]	2016
Ma, Y. 2016	5	33	13	35	1.8%	0.30 [0.09, 0.98]	2016
Zhang, S.2016	2	19	3	19	0.5%	0.63 [0.09, 4.26]	2016
Su, S.2017	4	40	3	39	0.5%	1.33 [0.28, 6.39]	2017
Wu, Y.2017	37	109	63	109	7.1%	0.38 [0.22, 0.65]	2017
Zhou, D.2018	29	58	31	58	2.7%	0.87 [0.42, 1.81]	2018
Zhang, H.2018	7	40	18	40	2.5%	0.26 [0.09, 0.72]	2018
Geng,K.2020	21	45	30	45	2.7%	0.44 [0.19, 1.03]	2020
Guo, X.2020	5	51	13	51	2.0%	0.32 [0.10, 0.97]	2020
Tan, H.2020	47	60	54	60	2.0%	0.40 [0.14, 1.14]	2020
**Subtotal (95% CI)**		**1276**		**1246**	**61.0%**	**0.47 [0.39, 0.56]**	
Total events	511		685				
Heterogeneity: Chi² = 20.69, df = 28 (P = 0.84); I² = 0%							
Test for overall effect: Z = 8.29 (P < 0.00001)							
**Aidi injection(70-80ml)**							
Zou,Y.2006	32	42	29	39	1.2%	1.10 [0.40, 3.03]	2006
Zhang, L.2009	11	32	20	31	2.3%	0.29 [0.10, 0.81]	2009
Lv,D.2009	10	30	19	30	2.2%	0.29 [0.10, 0.84]	2009
Wang,Y.2012	21	36	28	36	2.0%	0.40 [0.14, 1.12]	2012
Xu,Y.2012	7	33	18	33	2.4%	0.22 [0.08, 0.66]	2012
**Subtotal (95% CI)**		**173**		**169**	**10.1%**	**0.39 [0.25, 0.62]**	
Total events	81		114				
Heterogeneity: Chi² = 5.71, df = 4 (P = 0.22); I² = 30%							
Test for overall effect: Z = 3.99 (P < 0.0001)							
**Aidi injection(90-100ml)**							
Lu,Z.2011	10	34	19	34	2.3%	0.33 [0.12, 0.90]	2011
Zhao, S.2015	5	43	10	43	1.5%	0.43 [0.13, 1.40]	2015
Zhu, H.2015	7	21	16	21	1.8%	0.16 [0.04, 0.60]	2015
Li, J.-c.2016	7	35	19	35	2.6%	0.21 [0.07, 0.61]	2016
Wu, T.2017	11	67	23	68	3.3%	0.38 [0.17, 0.87]	2017
**Subtotal (95% CI)**		**200**		**201**	**11.5%**	**0.30 [0.19, 0.48]**	
Total events	40		87				
Heterogeneity: Chi² = 2.08, df = 4 (P = 0.72); I² = 0%							
Test for overall effect: Z = 5.05 (P < 0.00001)							
**Aidi injection(others)**							
Wang,Y.2009	5	32	5	27	0.8%	0.81 [0.21, 3.18]	2009
Li,Z.2010	16	36	20	36	1.9%	0.64 [0.25, 1.62]	2010
He,W.2011	12	29	13	23	1.5%	0.54 [0.18, 1.64]	2011
Cai,H.2013	6	19	8	19	0.9%	0.63 [0.17, 2.40]	2013
Cheng, B.2014	7	49	7	52	1.0%	1.07 [0.35, 3.31]	2014
Li, J.2016	15	47	27	47	3.2%	0.35 [0.15, 0.81]	2016
Zhang, H.2017	16	52	29	52	3.4%	0.35 [0.16, 0.79]	2017
Su,D.2019	5	41	15	41	2.3%	0.24 [0.08, 0.75]	2019
Xu,Z.2020	2	40	7	40	1.1%	0.25 [0.05, 1.28]	2020
Chen,C.2020	40	49	42	49	1.3%	0.74 [0.25, 2.18]	2020
**Subtotal (95% CI)**		**394**		**386**	**17.4%**	**0.48 [0.35, 0.67]**	
Total events	124		173				
Heterogeneity: Chi² = 6.91, df = 9 (P = 0.65); I² = 0%							
Test for overall effect: Z = 4.29 (P < 0.0001)							
**Total (95% CI)**		**2043**		**2002**	**100.0%**	**0.45 [0.39, 0.51]**	
Total events	756		1059				
Heterogeneity: Chi² = 38.98, df = 48 (P = 0.82); I² = 0%							
Test for overall effect: Z = 11.21 (P < 0.00001)							
Test for subgroup differences: Chi² = 3.48, df = 3 (P = 0.32), I² = 13.9%							

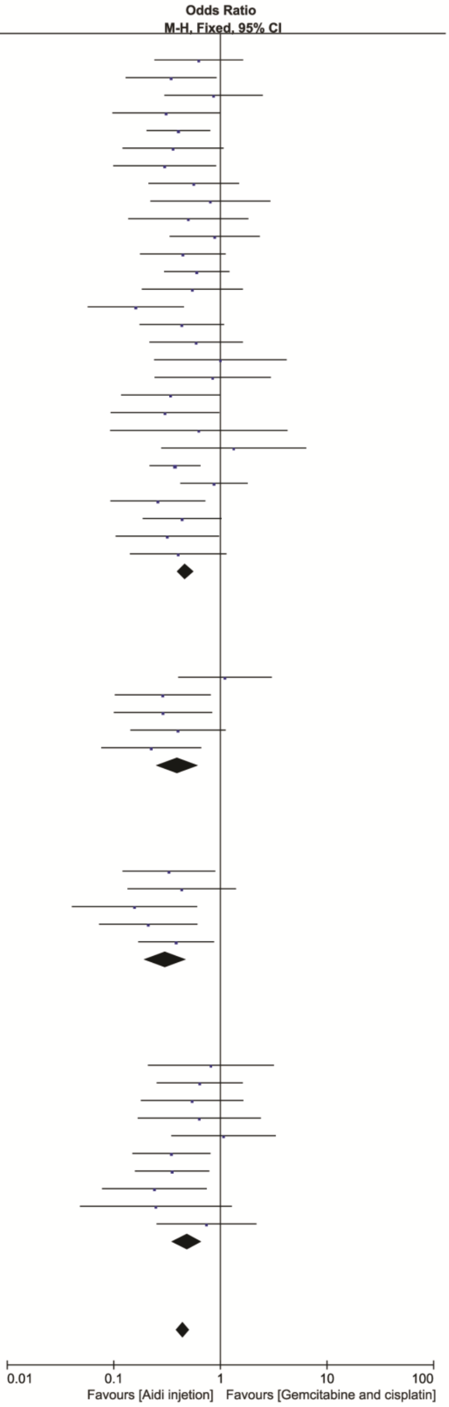

Figure S73 Subgroups analysis of gastrointestinal reaction by dosage of Aidi injection

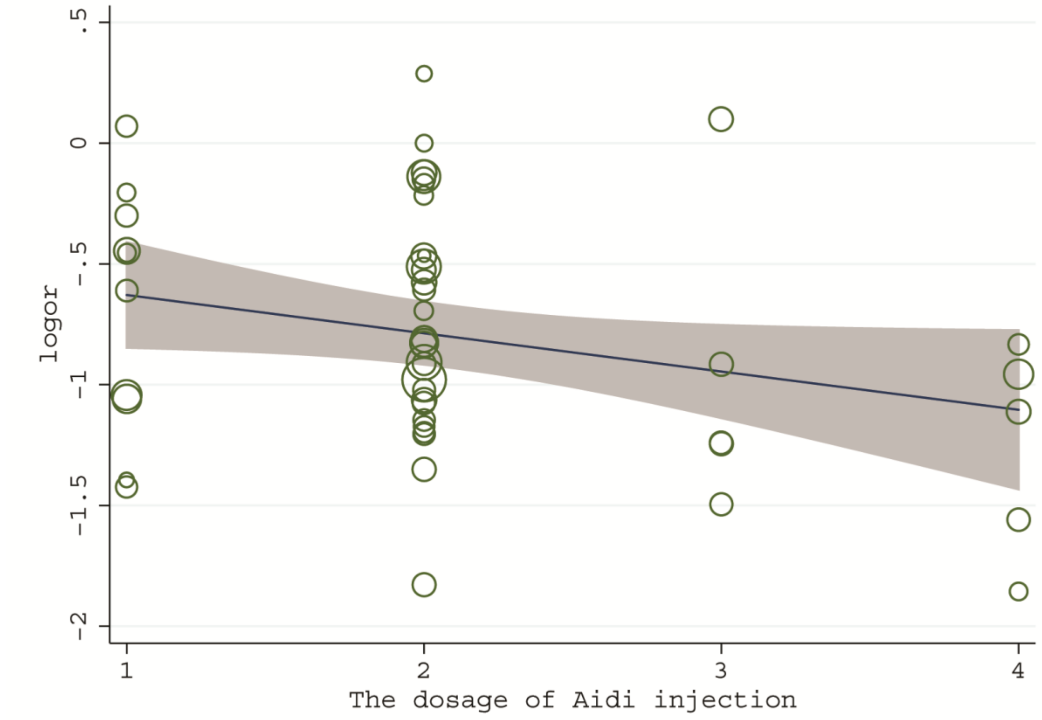

Figure S74 Meta-regression of gastrointestinal reaction by dosage of Aidi injection

Study or Subgroup	Aidi injetion Events	Aidi injetion Total	Gemcitabine and cisplatin Events	Gemcitabine and cisplatin Total	Weight	Odds Ratio M-H, Fixed, 95% CI	Year
**Treatment time(7 to 10days/cycle)**							
Yang,Q.2008	23	30	11	27	0.6%	4.78 [1.52, 14.98]	2008
Lv,D.2009	22	30	11	30	0.7%	4.75 [1.58, 14.25]	2009
Wen,K.2009	16	38	15	38	2.1%	1.12 [0.45, 2.79]	2009
Wang,Y.2009	17	32	12	27	1.4%	1.42 [0.51, 3.96]	2009
Ding,P.2011	9	18	10	22	1.1%	1.20 [0.34, 4.18]	2011
Wang,Y.2012	16	36	14	36	1.8%	1.26 [0.49, 3.21]	2012
Xu,Y.2012	16	33	16	33	2.0%	1.00 [0.38, 2.63]	2012
Pei, W.2012	15	40	11	40	1.6%	1.58 [0.62, 4.07]	2012
Sun,J.2012	15	34	13	34	1.7%	1.28 [0.48, 3.36]	2012
Li, H.2014	16	30	12	30	1.3%	1.71 [0.62, 4.77]	2014
Li, J.2014	11	27	10	27	1.4%	1.17 [0.39, 3.49]	2014
Liu,Y.2014	18	43	17	43	2.3%	1.10 [0.47, 2.60]	2014
Cheng, B.2014	12	49	16	52	2.8%	0.73 [0.30, 1.76]	2014
Zhao, S.2015	19	43	12	43	1.6%	2.05 [0.83, 5.02]	2015
Zhang, M.2015	17	39	12	32	1.8%	1.29 [0.50, 3.35]	2015
Chen, Y.2016	15	30	13	30	1.5%	1.31 [0.47, 3.61]	2016
Fang, L.2016	35	45	27	45	1.4%	2.33 [0.93, 5.87]	2016
Zhang, X.2016	18	25	8	25	0.5%	5.46 [1.63, 18.36]	2016
Wu, T.2017	53	67	40	68	2.0%	2.65 [1.24, 5.68]	2017
Zhang, H.2017	42	52	26	52	1.2%	4.20 [1.75, 10.11]	2017
Chen,C.2020	26	49	16	49	1.8%	2.33 [1.03, 5.29]	2020
Tan, H.2020	24	60	18	60	2.6%	1.56 [0.73, 3.31]	2020
**Subtotal (95% CI)**		**850**		**843**	**35.2%**	**1.75 [1.43, 2.13]**	
Total events	455		340				
Heterogeneity: Chi² = 25.38, df = 21 (P = 0.23); I² = 17%							
Test for overall effect: Z = 5.53 (P < 0.00001)							
**Treatment time(14 to 15days/cycle)**							
Zou,Y.2006	24	42	13	39	1.4%	2.67 [1.08, 6.58]	2006
Feng,X.2008	30	68	25	62	3.5%	1.17 [0.58, 2.35]	2008
Sun,G.2008	17	33	15	30	1.8%	1.06 [0.40, 2.86]	2008
Song,Z.2009	10	30	9	30	1.4%	1.17 [0.39, 3.47]	2009
Zhang, L.2009	15	32	14	31	1.8%	1.07 [0.40, 2.89]	2009
Hou,A.2010	16	40	14	38	2.0%	1.14 [0.46, 2.85]	2010
Liu,Y.2010	16	32	10	32	1.2%	2.20 [0.79, 6.10]	2010
Shi,X.2010	13	28	10	28	1.3%	1.56 [0.53, 4.56]	2010
Hong,Y.2010	57	90	32	70	3.1%	2.05 [1.09, 3.88]	2010
Li,Z.2010	16	36	12	36	1.6%	1.60 [0.62, 4.16]	2010
Wu,Q.2011	17	30	11	30	1.1%	2.26 [0.80, 6.36]	2011
Jiang,S.2011	15	32	12	30	1.6%	1.32 [0.48, 3.63]	2011
Lu,Z.2011	18	34	17	34	1.9%	1.13 [0.43, 2.91]	2011
He,W.2011	15	29	10	23	1.3%	1.39 [0.46, 4.18]	2011
Fu, L.2012	19	35	16	35	1.7%	1.41 [0.55, 3.61]	2012
Wang,J.2012	14	25	10	24	1.1%	1.78 [0.57, 5.53]	2012
Zhang,Y. 2012	21	41	15	42	1.7%	1.89 [0.78, 4.55]	2012
Lai,L.2013	45	70	40	70	3.4%	1.35 [0.68, 2.67]	2013
Ju,S.2013	19	34	12	34	1.3%	2.32 [0.87, 6.16]	2013
Xu, H.2013	19	38	18	42	2.0%	1.33 [0.55, 3.22]	2013
Ning, S.2015	19	31	11	31	1.0%	2.88 [1.03, 8.07]	2015
Zhu, H.2015	15	21	7	21	0.5%	5.00 [1.35, 18.55]	2015
Li, J.-c.2016	21	35	15	35	1.4%	2.00 [0.77, 5.18]	2016
Ma, Y. 2016	19	33	19	35	1.9%	1.14 [0.44, 2.98]	2016
Lv, W.2018	16	30	15	30	1.7%	1.14 [0.41, 3.15]	2018
Geng,K.2020	27	45	17	45	1.6%	2.47 [1.06, 5.77]	2020
**Subtotal (95% CI)**		**994**		**957**	**44.1%**	**1.62 [1.35, 1.94]**	
Total events	533		399				
Heterogeneity: Chi² = 13.70, df = 25 (P = 0.97); I² = 0%							
Test for overall effect: Z = 5.25 (P < 0.00001)							
**Treatment time(21 to 28 days/cycle)**							
Zhao,H.2008	11	30	6	20	1.1%	1.35 [0.40, 4.53]	2008
Fan,S.2011	22	41	12	38	1.4%	2.51 [1.00, 6.29]	2011
Wen, H.2014	28	45	17	45	1.5%	2.71 [1.16, 6.36]	2014
Zhang, S.2016	11	19	8	19	0.8%	1.89 [0.52, 6.85]	2016
Li, J.2016	23	47	9	47	1.1%	4.05 [1.61, 10.20]	2016
Huang, W.2017	17	39	11	40	1.5%	2.04 [0.80, 5.21]	2017
Su, S.2017	35	40	23	39	0.7%	4.87 [1.57, 15.13]	2017
Zhou, D.2018	30	58	22	58	2.5%	1.75 [0.84, 3.67]	2018
Zhao,J.2019	31	43	22	43	1.5%	2.47 [1.01, 6.04]	2019
Su,D.2019	30	41	17	41	1.1%	3.85 [1.52, 9.75]	2019
Xu,Z.2020	25	40	14	40	1.2%	3.10 [1.24, 7.71]	2020
Xu, Y.2020	16	45	12	45	1.8%	1.52 [0.62, 3.73]	2020
**Subtotal (95% CI)**		**488**		**475**	**16.1%**	**2.48 [1.90, 3.25]**	
Total events	279		173				
Heterogeneity: Chi² = 6.87, df = 11 (P = 0.81); I² = 0%							
Test for overall effect: Z = 6.63 (P < 0.00001)							
**Treatment time(others)**							
Han, R.2015	14	36	10	36	1.4%	1.65 [0.61, 4.45]	2015
Ma, M.2017	19	42	12	42	1.6%	2.07 [0.84, 5.10]	2017
Liu, H.2019	19	44	12	44	1.6%	2.03 [0.83, 4.95]	2019
**Subtotal (95% CI)**		**122**		**122**	**4.6%**	**1.92 [1.13, 3.28]**	
Total events	52		34				
Heterogeneity: Chi² = 0.13, df = 2 (P = 0.94); I² = 0%							
Test for overall effect: Z = 2.40 (P = 0.02)							
**Total (95% CI)**		**2454**		**2397**	**100.0%**	**1.82 [1.62, 2.04]**	
Total events	1319		946				
Heterogeneity: Chi² = 52.84, df = 62 (P = 0.79); I² = 0%							
Test for overall effect: Z = 10.08 (P < 0.00001)							
Test for subgroup differences: Chi² = 6.93, df = 3 (P = 0.07), I² = 56.7%							

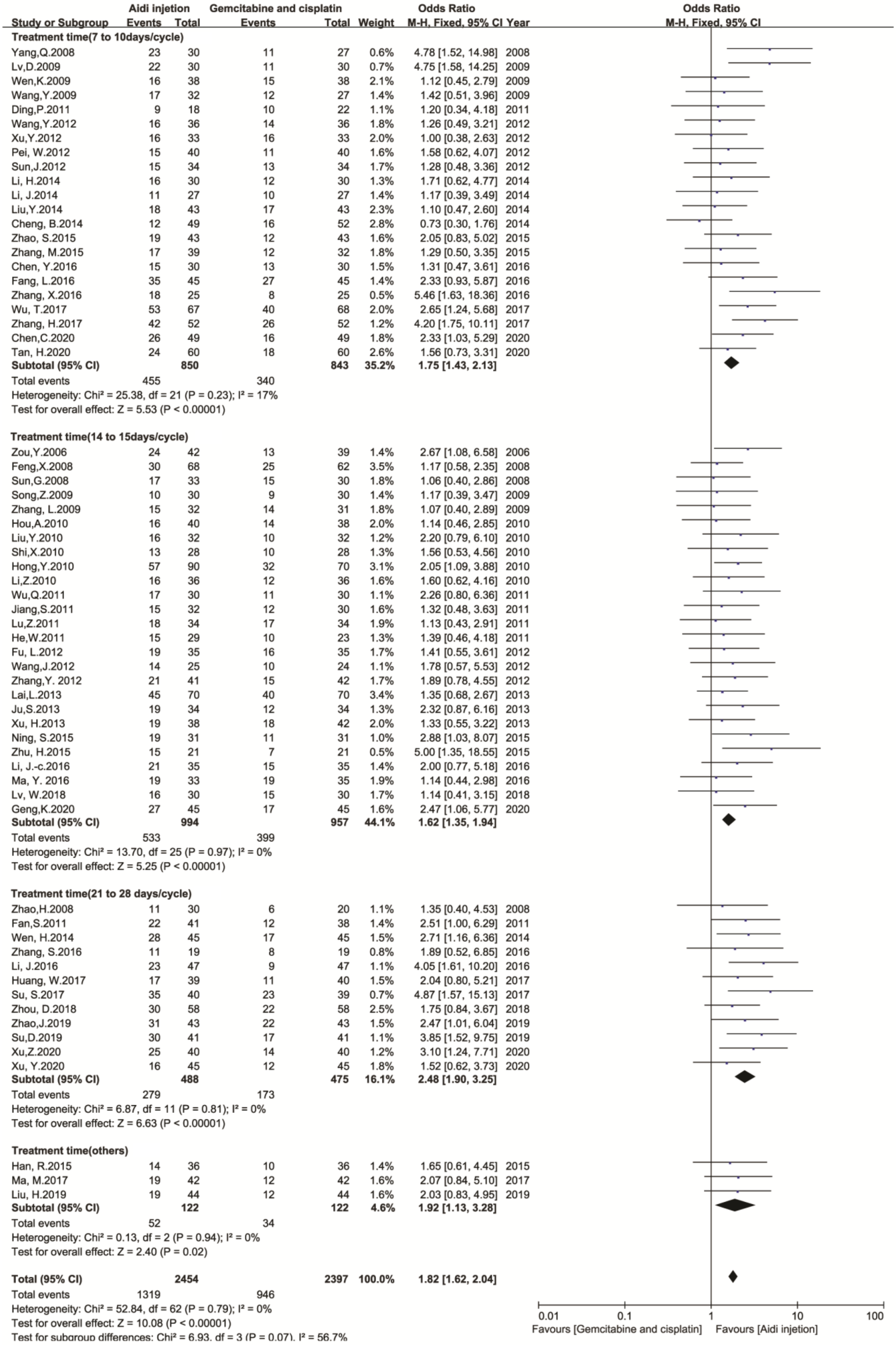

Figure S75 Subgroups analysis of objective response rate by treatment time

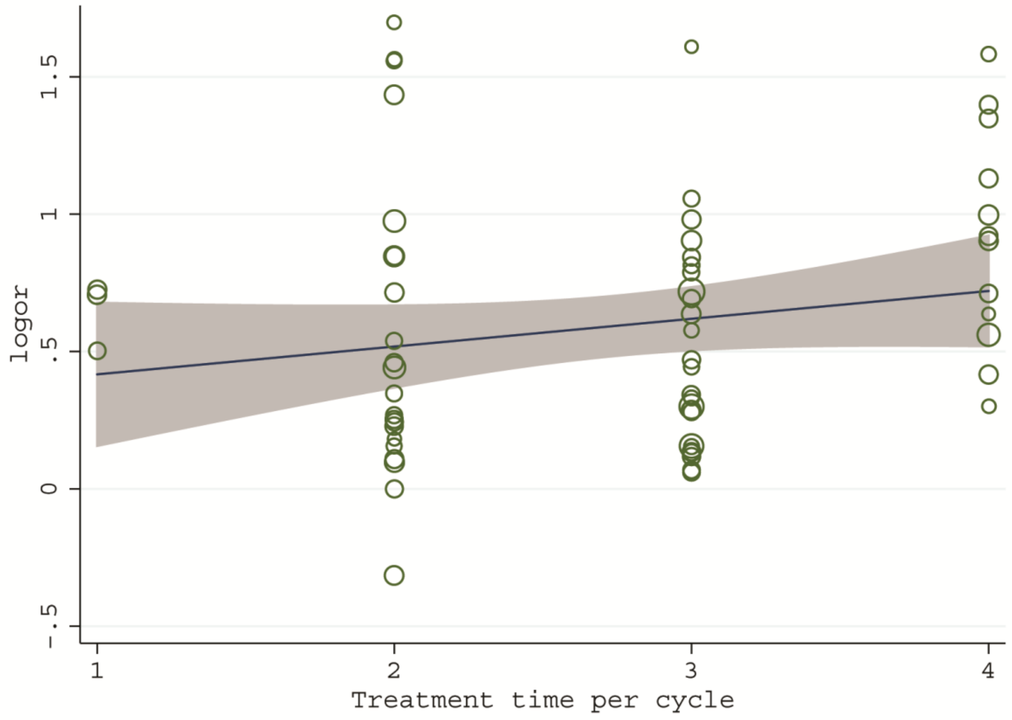

Figure S76 Meta-regression of objective response rate by treatment time

Study or Subgroup	Aidi injetion Events	Aidi injetion Total	Gemcitabine and cisplatin Events	Gemcitabine and cisplatin Total	Weight	Odds Ratio M-H, Fixed, 95% CI	Year
**Treatment time(7 to 10days/cycle)**							
Yang,Q.2008	29	30	23	27	0.4%	5.04 [0.53, 48.26]	2008
Wang,Y.2009	29	32	21	27	1.0%	2.76 [0.62, 12.32]	2009
Wen,K.2009	32	38	32	38	2.3%	1.00 [0.29, 3.43]	2009
Lv,D.2009	28	30	23	30	0.7%	4.26 [0.81, 22.53]	2009
Pei, W.2012	35	40	27	40	1.5%	3.37 [1.07, 10.61]	2012
Sun,J.2012	25	34	25	34	3.0%	1.00 [0.34, 2.94]	2012
Wang,Y.2012	28	36	20	36	2.0%	2.80 [1.01, 7.80]	2012
Xu,Y.2012	30	33	26	33	1.1%	2.69 [0.63, 11.49]	2012
Li, H.2014	26	30	23	30	1.4%	1.98 [0.51, 7.63]	2014
Cheng, B.2014	39	49	42	52	3.7%	0.93 [0.35, 2.47]	2014
Li, J.2014	19	27	16	27	2.1%	1.63 [0.53, 5.04]	2014
Liu,Y.2014	35	43	33	43	2.8%	1.33 [0.47, 3.77]	2014
Zhang, M.2015	28	39	23	32	3.2%	1.00 [0.35, 2.82]	2015
Zhao, S.2015	37	43	28	43	1.8%	3.30 [1.14, 9.60]	2015
Fang, L.2016	42	45	34	45	1.0%	4.53 [1.17, 17.55]	2016
Chen, Y.2016	24	30	18	30	1.6%	2.67 [0.84, 8.46]	2016
Zhang, X.2016	21	25	14	25	1.0%	4.13 [1.09, 15.59]	2016
Zhang, H.2017	50	52	46	52	0.8%	3.26 [0.63, 16.97]	2017
Wu, T.2017	62	67	56	68	1.9%	2.66 [0.88, 8.02]	2017
Tan, H.2020	49	60	39	60	3.2%	2.40 [1.03, 5.57]	2020
Chen,C.2020	44	49	34	49	1.6%	3.88 [1.28, 11.74]	2020
**Subtotal (95% CI)**		**832**		**821**	**37.9%**	**2.19 [1.70, 2.82]**	
Total events	712		603				
Heterogeneity: Chi² = 16.06, df = 20 (P = 0.71); I² = 0%							
Test for overall effect: Z = 6.11 (P < 0.00001)							
**Treatment time(14 to 15days/cycle)**							
Zou,Y.2006	35	42	25	39	1.9%	2.80 [0.99, 7.94]	2006
Sun,G.2008	31	33	27	30	0.8%	1.72 [0.27, 11.09]	2008
Feng,X.2008	62	68	56	62	2.3%	1.11 [0.34, 3.63]	2008
Zhang, L.2009	27	32	23	31	1.6%	1.88 [0.54, 6.54]	2009
Song,Z.2009	27	30	25	30	1.1%	1.80 [0.39, 8.32]	2009
Hong,Y.2010	78	90	50	70	3.4%	2.60 [1.17, 5.78]	2010
Shi,X.2010	27	28	25	28	0.4%	3.24 [0.32, 33.22]	2010
Liu,Y.2010	25	32	22	32	2.2%	1.62 [0.53, 4.99]	2010
Hou,A.2010	37	40	29	38	1.0%	3.83 [0.95, 15.43]	2010
Li,Z.2010	18	36	21	36	4.7%	0.71 [0.28, 1.81]	2010
Lu,Z.2011	30	34	28	34	1.5%	1.61 [0.41, 6.30]	2011
Wu,Q.2011	28	30	26	30	0.8%	2.15 [0.36, 12.76]	2011
Jiang,S.2011	29	32	25	30	1.1%	1.93 [0.42, 8.91]	2011
He,W.2011	26	29	19	23	1.0%	1.82 [0.36, 9.12]	2011
Wang,J.2012	20	25	16	24	1.5%	2.00 [0.55, 7.31]	2012
Zhang,Y. 2012	34	41	32	42	2.4%	1.52 [0.52, 4.47]	2012
Fu, L.2012	32	35	28	35	1.1%	2.67 [0.63, 11.31]	2012
Lai,L.2013	63	70	60	70	2.7%	1.50 [0.54, 4.20]	2013
Xu, H.2013	33	38	34	42	1.9%	1.55 [0.46, 5.24]	2013
Ju,S.2013	32	34	29	34	0.8%	2.76 [0.50, 15.33]	2013
Zhu, H.2015	19	21	14	21	0.6%	4.75 [0.85, 26.43]	2015
Ning, S.2015	26	31	22	31	1.6%	2.13 [0.62, 7.29]	2015
Li, J.-c.2016	31	35	26	35	1.3%	2.68 [0.74, 9.73]	2016
Ma, Y. 2016	28	33	28	35	1.9%	1.40 [0.40, 4.94]	2016
Lv, W.2018	24	30	25	30	2.2%	0.80 [0.22, 2.97]	2018
Geng,K.2020	39	45	31	45	1.9%	2.94 [1.01, 8.53]	2020
**Subtotal (95% CI)**		**994**		**957**	**43.7%**	**1.87 [1.46, 2.38]**	
Total events	861		746				
Heterogeneity: Chi² = 12.28, df = 25 (P = 0.98); I² = 0%							
Test for overall effect: Z = 5.04 (P < 0.00001)							
**Treatment time(21 to 28 days/cycle)**							
Fan,S.2011	35	41	29	38	2.0%	1.81 [0.58, 5.68]	2011
Wen, H.2014	43	45	36	45	0.7%	5.38 [1.09, 26.49]	2014
Li, J.2016	45	47	34	47	0.7%	8.60 [1.82, 40.69]	2016
Zhang, S.2016	14	19	14	19	1.7%	1.00 [0.24, 4.24]	2016
Su, S.2017	37	40	26	39	0.9%	6.17 [1.60, 23.83]	2017
Huang, W.2017	36	39	30	40	1.0%	4.00 [1.01, 15.87]	2017
Zhou, D.2018	50	58	38	58	2.4%	3.29 [1.31, 8.27]	2018
Zhao,J.2019	39	43	34	43	1.4%	2.58 [0.73, 9.14]	2019
Su,D.2019	39	41	33	41	0.7%	4.73 [0.94, 23.82]	2019
Xu, Y.2020	43	45	33	45	0.7%	7.82 [1.64, 37.36]	2020
Xu,Z.2020	33	40	24	40	1.9%	3.14 [1.12, 8.82]	2020
**Subtotal (95% CI)**		**458**		**455**	**14.0%**	**3.59 [2.46, 5.24]**	
Total events	414		331				
Heterogeneity: Chi² = 7.91, df = 10 (P = 0.64); I² = 0%							
Test for overall effect: Z = 6.64 (P < 0.00001)							
**Treatment time(others)**							
Han, R.2015	32	36	27	36	1.3%	2.67 [0.74, 9.63]	2015
Ma, M.2017	37	42	29	42	1.6%	3.32 [1.06, 10.37]	2017
Liu, H.2019	39	44	30	44	1.5%	3.64 [1.18, 11.23]	2019
**Subtotal (95% CI)**		**122**		**122**	**4.4%**	**3.23 [1.64, 6.37]**	
Total events	108		86				
Heterogeneity: Chi² = 0.13, df = 2 (P = 0.94); I² = 0%							
Test for overall effect: Z = 3.38 (P = 0.0007)							
**Total (95% CI)**		**2406**		**2355**	**100.0%**	**2.29 [1.97, 2.67]**	
Total events	2095		1766				
Heterogeneity: Chi² = 44.59, df = 60 (P = 0.93); I² = 0%							
Test for overall effect: Z = 10.58 (P < 0.00001)							
Test for subgroup differences: Chi² = 9.29, df = 3 (P = 0.03), I² = 67.7%							

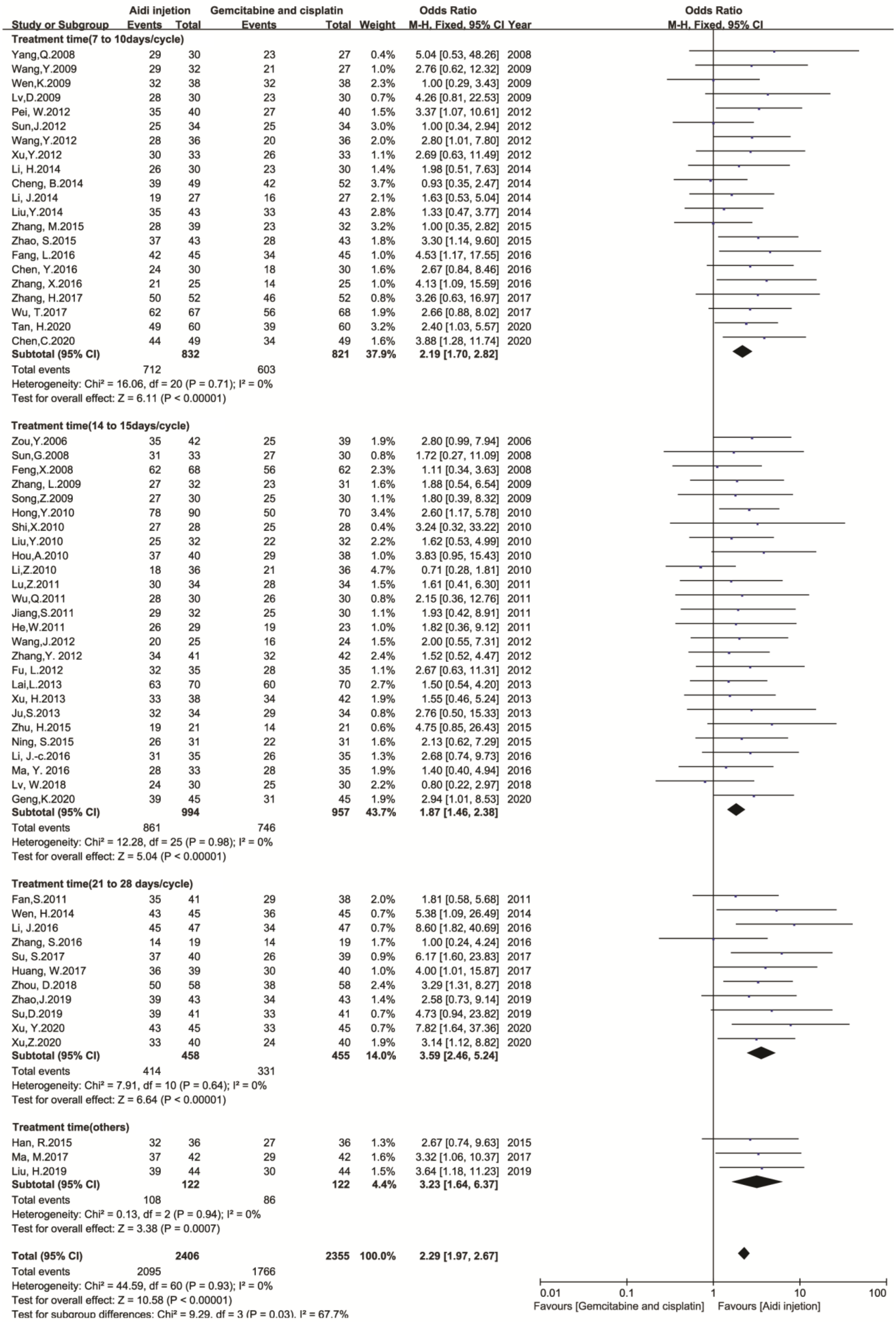

Figure S77 Subgroups analysis of disease control rate by treatment time

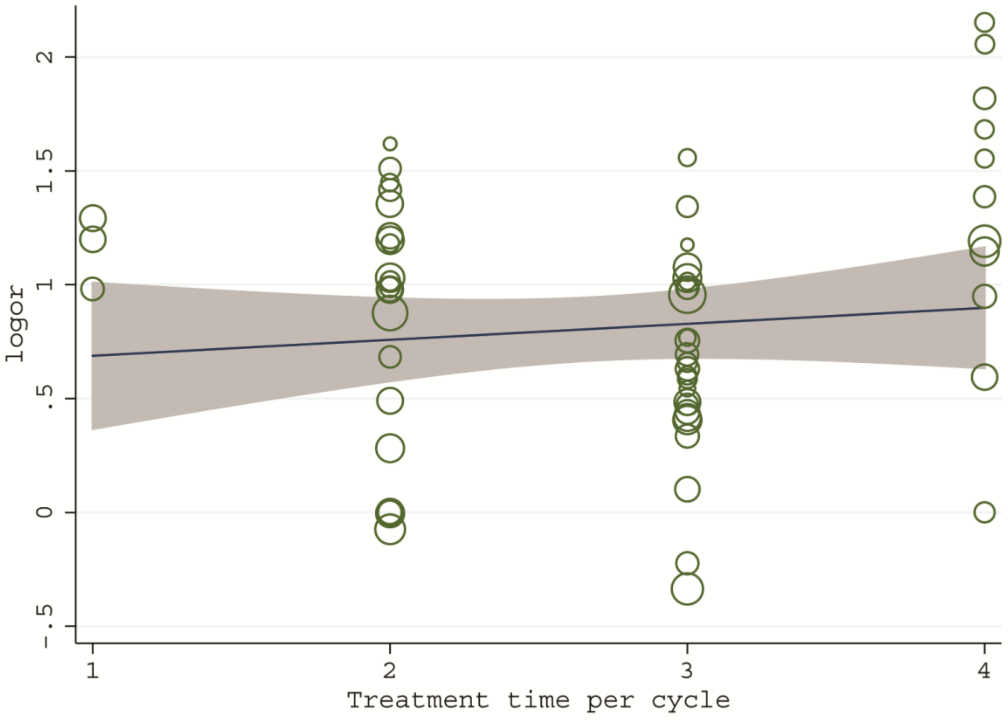

Figure S78 Meta-regression of disease control rate by treatment time

Study or Subgroup	Aidi injection Events	Aidi injection Total	Gemcitabine and cisplatin Events	Gemcitabine and cisplatin Total	Weight	Odds Ratio M-H, Fixed, 95% CI	Year
**Treatment time(7 to 10days/cycle)**							
Wang,Y.2009	26	32	22	27	1.0%	0.98 [0.26, 3.67]	2009
Wen,K.2009	23	38	31	38	2.8%	0.35 [0.12, 0.99]	2009
Sun,J.2012	23	34	30	34	2.2%	0.28 [0.08, 0.99]	2012
Xu,Y.2012	23	33	33	33	2.4%	0.03 [0.00, 0.60]	2012
Wang,Y.2012	19	36	26	36	2.8%	0.43 [0.16, 1.15]	2012
Liu,Y.2014	27	43	36	43	3.1%	0.33 [0.12, 0.91]	2014
Li, J.2014	5	27	6	27	1.1%	0.80 [0.21, 3.00]	2014
Cheng, B.2014	34	49	47	52	3.2%	0.24 [0.08, 0.73]	2014
Zhao, S.2015	7	43	10	43	1.9%	0.64 [0.22, 1.88]	2015
Zhang, M.2015	26	39	28	32	2.4%	0.29 [0.08, 0.99]	2015
Li, X.2015	2	20	8	20	1.7%	0.17 [0.03, 0.92]	2015
Fang, L.2016	5	45	13	45	2.7%	0.31 [0.10, 0.95]	2016
Zhang, H.2017	13	52	22	52	3.8%	0.45 [0.20, 1.05]	2017
Wu, T.2017	10	67	21	68	4.1%	0.39 [0.17, 0.92]	2017
Zhang, H.2018	13	30	22	30	2.9%	0.28 [0.09, 0.82]	2018
Chen,C.2020	33	49	43	49	3.2%	0.29 [0.10, 0.82]	2020
Tan, H.2020	51	60	57	60	2.0%	0.30 [0.08, 1.16]	2020
**Subtotal (95% CI)**		**697**		**689**	**43.5%**	**0.35 [0.27, 0.46]**	
Total events	340		455				
Heterogeneity: Chi² = 10.01, df = 16 (P = 0.87); I² = 0%							
Test for overall effect: Z = 7.63 (P < 0.00001)							
**Treatment time(14 to 15days/cycle)**							
Feng,X.2008	64	68	60	62	0.9%	0.53 [0.09, 3.02]	2008
Zhang, L.2009	5	32	14	31	2.8%	0.22 [0.07, 0.74]	2009
Hou,A.2010	14	40	22	38	3.4%	0.39 [0.16, 0.98]	2010
Hong,Y.2010	26	90	31	70	5.7%	0.51 [0.27, 0.98]	2010
Lu,Z.2011	11	34	20	34	3.1%	0.33 [0.12, 0.90]	2011
Wang,J.2012	7	25	9	24	1.5%	0.65 [0.19, 2.16]	2012
Fu, L.2012	24	35	32	35	2.3%	0.20 [0.05, 0.81]	2012
Zhang,Y. 2012	12	41	17	42	2.7%	0.61 [0.24, 1.52]	2012
Xu, H.2013	13	38	22	42	3.2%	0.47 [0.19, 1.17]	2013
Ning, S.2015	16	31	16	31	1.8%	1.00 [0.37, 2.71]	2015
Zhu, H.2015	6	21	15	21	2.5%	0.16 [0.04, 0.61]	2015
Ma, Y. 2016	7	33	15	35	2.7%	0.36 [0.12, 1.05]	2016
Li, J.-c.2016	14	35	26	35	3.6%	0.23 [0.08, 0.64]	2016
Wu, Y.2017	3	109	9	109	2.0%	0.31 [0.08, 1.19]	2017
Guo, X.2020	7	51	16	51	3.2%	0.35 [0.13, 0.94]	2020
**Subtotal (95% CI)**		**683**		**660**	**41.4%**	**0.40 [0.31, 0.53]**	
Total events	229		324				
Heterogeneity: Chi² = 10.54, df = 14 (P = 0.72); I² = 0%							
Test for overall effect: Z = 6.78 (P < 0.00001)							
**Treatment time(21 to 28 days/cycle)**							
Li, J.2016	12	47	19	47	3.3%	0.51 [0.21, 1.21]	2016
Zhang, S.2016	2	19	4	19	0.8%	0.44 [0.07, 2.76]	2016
Su, S.2017	2	40	2	39	0.4%	0.97 [0.13, 7.28]	2017
Huang, W.2017	10	39	12	40	2.0%	0.80 [0.30, 2.16]	2017
Zhou, D.2018	11	58	13	58	2.4%	0.81 [0.33, 1.99]	2018
Xu,Z.2020	2	40	6	40	1.3%	0.30 [0.06, 1.58]	2020
**Subtotal (95% CI)**		**243**		**243**	**10.3%**	**0.62 [0.39, 1.00]**	
Total events	39		56				
Heterogeneity: Chi² = 1.88, df = 5 (P = 0.87); I² = 0%							
Test for overall effect: Z = 1.97 (P = 0.05)							
**Treatment time(others)**							
Han, R.2015	7	36	11	36	2.0%	0.55 [0.18, 1.63]	2015
Ma, M.2017	11	42	16	42	2.7%	0.58 [0.23, 1.46]	2017
**Subtotal (95% CI)**		**78**		**78**	**4.8%**	**0.56 [0.28, 1.14]**	
Total events	18		27				
Heterogeneity: Chi² = 0.00, df = 1 (P = 0.95); I² = 0%							
Test for overall effect: Z = 1.59 (P = 0.11)							
**Total (95% CI)**		**1701**		**1670**	**100.0%**	**0.41 [0.35, 0.49]**	
Total events	626		862				
Heterogeneity: Chi² = 26.97, df = 39 (P = 0.93); I² = 0%							
Test for overall effect: Z = 10.32 (P < 0.00001)							
Test for subgroup differences: Chi² = 5.13, df = 3 (P = 0.16), I² = 41.5%							

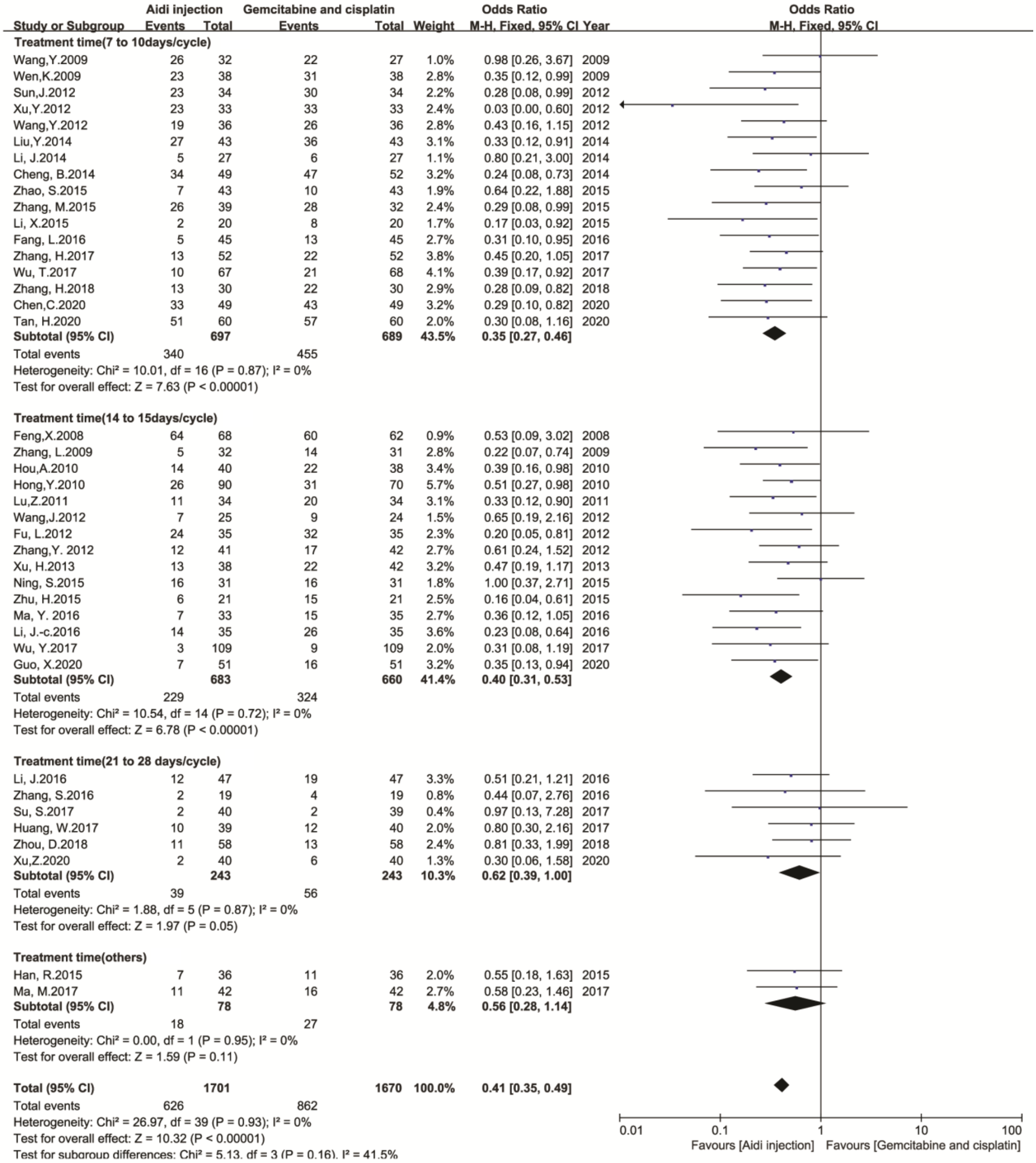

Figure S79 Subgroups analysis of neutropenia by treatment time

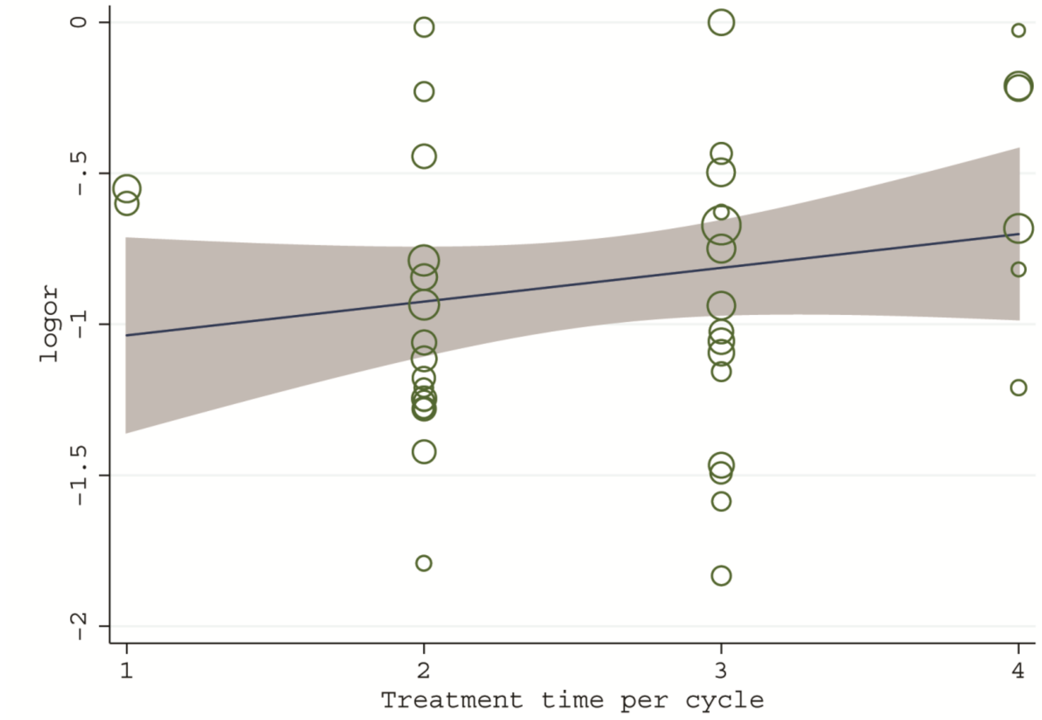

Figure S80 Meta-regression of neutropenia by treatment time

	Aidi injetion		Gemcitabine and cisplatin			Odds Ratio	
Study or Subgroup	Events	Total	Events	Total	Weight	M-H, Fixed, 95% CI	Year
**Treatment time(7 to 10days/cycle)**							
Wang,Y.2009	5	32	5	27	0.8%	0.81 [0.21, 3.18]	2009
Lv,D.2009	10	30	19	30	2.2%	0.29 [0.10, 0.84]	2009
Wen,K.2009	20	38	29	38	2.4%	0.34 [0.13, 0.92]	2009
Sun,J.2012	28	34	29	34	0.9%	0.80 [0.22, 2.94]	2012
Xu,Y.2012	7	33	18	33	2.4%	0.22 [0.08, 0.66]	2012
Wang,Y.2012	21	36	28	36	2.0%	0.40 [0.14, 1.12]	2012
Li, H.2014	18	30	22	30	1.5%	0.55 [0.18, 1.62]	2014
Cheng, B.2014	7	49	7	52	1.0%	1.07 [0.35, 3.31]	2014
Liu,Y.2014	24	43	32	43	2.4%	0.43 [0.17, 1.08]	2014
Zhao, S.2015	5	43	10	43	1.5%	0.43 [0.13, 1.40]	2015
Li, X.2015	15	20	15	20	0.6%	1.00 [0.24, 4.18]	2015
Zhang, M.2015	32	39	27	32	0.9%	0.85 [0.24, 2.97]	2015
Fang, L.2016	6	45	14	45	2.1%	0.34 [0.12, 0.99]	2016
Wu, T.2017	11	67	23	68	3.3%	0.38 [0.17, 0.87]	2017
Zhang, H.2017	16	52	29	52	3.4%	0.35 [0.16, 0.79]	2017
Zhang, H.2018	7	40	18	40	2.5%	0.26 [0.09, 0.72]	2018
Chen,C.2020	40	49	42	49	1.3%	0.74 [0.25, 2.18]	2020
Tan, H.2020	47	60	54	60	2.0%	0.40 [0.14, 1.14]	2020
**Subtotal (95% CI)**		**740**		**732**	**33.3%**	**0.44 [0.35, 0.57]**	
Total events	319		421				
Heterogeneity: Chi^2 = 11.38, df = 17 (P = 0.84); I^2 = 0%							
Test for overall effect: Z = 6.48 (P < 0.00001)							
**Treatment time(14 to 15days/cycle)**							
Zou,Y.2006	32	42	29	39	1.2%	1.10 [0.40, 3.03]	2006
Feng,X.2008	55	68	54	62	1.9%	0.63 [0.24, 1.63]	2008
Song,Z.2009	10	30	11	30	1.3%	0.86 [0.30, 2.50]	2009
Zhang, L.2009	11	32	20	31	2.3%	0.29 [0.10, 0.81]	2009
Liu,Y.2010	7	32	14	32	1.9%	0.36 [0.12, 1.07]	2010
Hong,Y.2010	20	90	29	70	4.4%	0.40 [0.20, 0.80]	2010
Li,Z.2010	16	36	20	36	1.9%	0.64 [0.25, 1.62]	2010
Hou,A.2010	5	40	12	38	1.8%	0.31 [0.10, 0.99]	2010
Shi,X.2010	12	28	20	28	2.0%	0.30 [0.10, 0.91]	2010
He,W.2011	12	29	13	23	1.5%	0.54 [0.18, 1.64]	2011
Lu,Z.2011	10	34	19	34	2.3%	0.33 [0.12, 0.90]	2011
Wang,J.2012	5	25	8	24	1.1%	0.50 [0.14, 1.83]	2012
Zhang,Y. 2012	9	41	14	42	1.9%	0.56 [0.21, 1.50]	2012
Cai,H.2013	6	19	8	19	0.9%	0.63 [0.17, 2.40]	2013
Lai,L.2013	20	70	28	70	3.4%	0.60 [0.30, 1.21]	2013
Ju,S.2013	13	34	14	34	1.5%	0.88 [0.33, 2.34]	2013
Xu, H.2013	20	38	30	42	2.3%	0.44 [0.18, 1.12]	2013
Zhu, H.2015	7	21	16	21	1.8%	0.16 [0.04, 0.60]	2015
Ning, S.2015	12	31	16	31	1.7%	0.59 [0.22, 1.62]	2015
Ma, Y. 2016	5	33	13	35	1.8%	0.30 [0.09, 0.98]	2016
Li, J.-c.2016	7	35	19	35	2.6%	0.21 [0.07, 0.61]	2016
Wu, Y.2017	37	109	63	109	7.1%	0.38 [0.22, 0.65]	2017
Geng,K.2020	21	45	30	45	2.7%	0.44 [0.19, 1.03]	2020
Guo, X.2020	5	51	13	51	2.0%	0.32 [0.10, 0.97]	2020
**Subtotal (95% CI)**		**1013**		**981**	**53.3%**	**0.45 [0.37, 0.55]**	
Total events	357		513				
Heterogeneity: Chi^2 = 16.66, df = 23 (P = 0.83); I^2 = 0%							
Test for overall effect: Z = 8.08 (P < 0.00001)							
**Treatment time(21 to 28 days/cycle)**							
Wen, H.2014	23	45	39	45	3.3%	0.16 [0.06, 0.45]	2014
Zhang, S.2016	2	19	3	19	0.5%	0.63 [0.09, 4.26]	2016
Li, J.2016	15	47	27	47	3.2%	0.35 [0.15, 0.81]	2016
Su, S.2017	4	40	3	39	0.5%	1.33 [0.28, 6.39]	2017
Zhou, D.2018	29	58	31	58	2.7%	0.87 [0.42, 1.81]	2018
Su,D.2019	5	41	15	41	2.3%	0.24 [0.08, 0.75]	2019
Xu,Z.2020	2	40	7	40	1.1%	0.25 [0.05, 1.28]	2020
**Subtotal (95% CI)**		**290**		**289**	**13.4%**	**0.42 [0.29, 0.63]**	
Total events	80		125				
Heterogeneity: Chi^2 = 10.89, df = 6 (P = 0.09); I^2 = 45%							
Test for overall effect: Z = 4.29 (P < 0.0001)							
**Total (95% CI)**		**2043**		**2002**	**100.0%**	**0.45 [0.39, 0.51]**	
Total events	756		1059				
Heterogeneity: Chi^2 = 38.98, df = 48 (P = 0.82); I^2 = 0%							
Test for overall effect: Z = 11.21 (P < 0.00001)							
Test for subgroup differences: Chi^2 = 0.09, df = 2 (P = 0.96), I^2 = 0%							

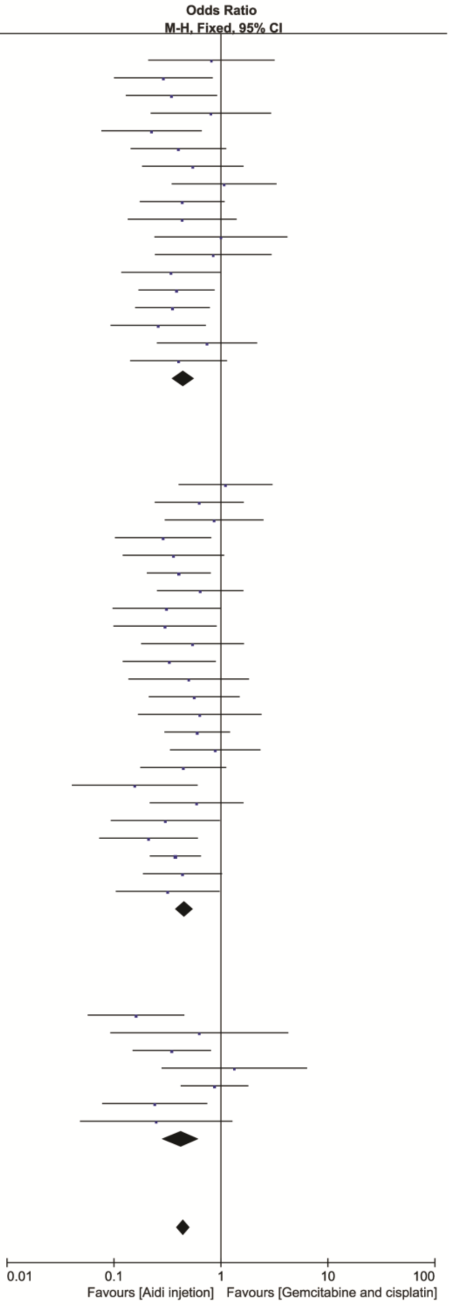

Figure S81 Subgroups analysis of gastrointestinal reaction by treatment time

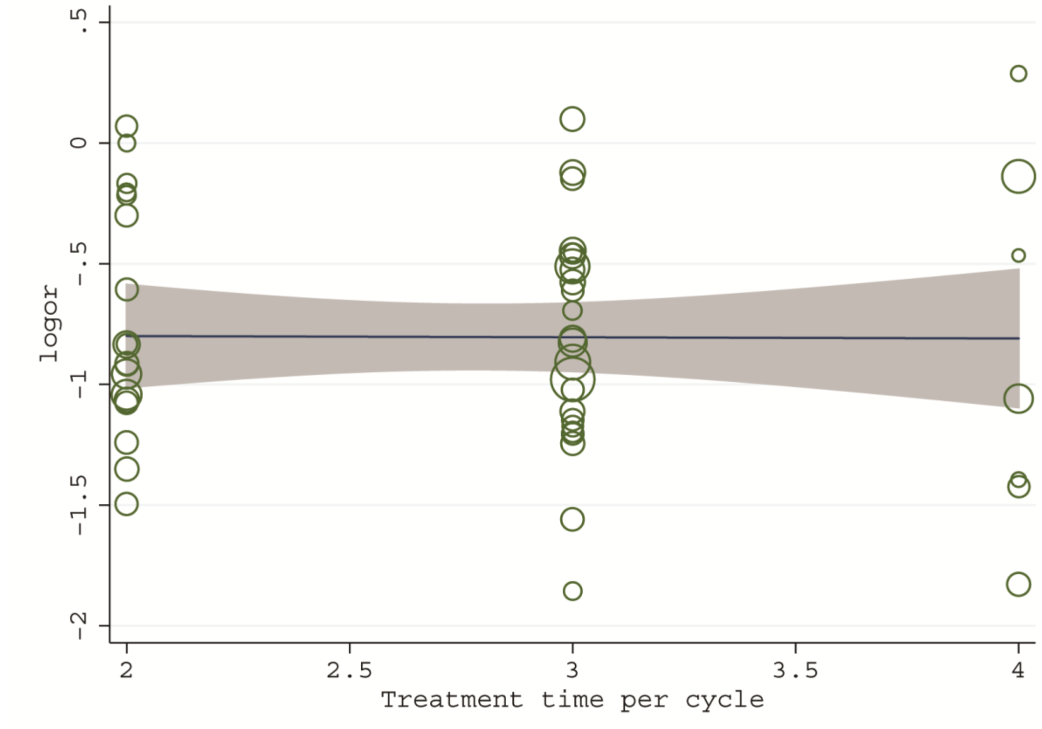

Figure S82 Meta-regression of gastrointestinal reaction by treatment time

Study or Subgroup	Aidi injetion Events	Aidi injetion Total	Gemcitabine and cisplatin Events	Gemcitabine and cisplatin Total	Weight	Odds Ratio M-H, Fixed, 95% CI	Year
**Treatment cycle(One cycle)**							
Zou,Y.2006	24	42	13	39	1.4%	2.67 [1.08, 6.58]	2006
Ma, Y. 2016	19	33	19	35	1.9%	1.14 [0.44, 2.98]	2016
Zhang, S.2016	11	19	8	19	0.8%	1.89 [0.52, 6.85]	2016
Li, J.2016	23	47	9	47	1.1%	4.05 [1.61, 10.20]	2016
Li, J.-c.2016	21	35	15	35	1.4%	2.00 [0.77, 5.18]	2016
**Subtotal (95% CI)**		**176**		**175**	**6.5%**	**2.22 [1.44, 3.43]**	
Total events	98		64				
Heterogeneity: Chi² = 3.73, df = 4 (P = 0.44); I² = 0%							
Test for overall effect: Z = 3.61 (P = 0.0003)							
**Treatment cycle(Two cycles)**							
Yang,Q.2008	23	30	11	27	0.6%	4.78 [1.52, 14.98]	2008
Sun,G.2008	17	33	15	30	1.8%	1.06 [0.40, 2.86]	2008
Feng,X.2008	30	68	25	62	3.5%	1.17 [0.58, 2.35]	2008
Lv,D.2009	22	30	11	30	0.7%	4.75 [1.58, 14.25]	2009
Song,Z.2009	10	30	9	30	1.4%	1.17 [0.39, 3.47]	2009
Wen,K.2009	16	38	15	38	2.1%	1.12 [0.45, 2.79]	2009
Wang,Y.2009	17	32	12	27	1.4%	1.42 [0.51, 3.96]	2009
Zhang, L.2009	15	32	14	31	1.8%	1.07 [0.40, 2.89]	2009
Shi,X.2010	13	28	10	28	1.3%	1.56 [0.53, 4.56]	2010
Hong,Y.2010	57	90	32	70	3.1%	2.05 [1.09, 3.88]	2010
Li,Z.2010	16	36	12	36	1.6%	1.60 [0.62, 4.16]	2010
Hou,A.2010	16	40	14	38	2.0%	1.14 [0.46, 2.85]	2010
Jiang,S.2011	15	32	12	30	1.6%	1.32 [0.48, 3.63]	2011
Ding,P.2011	9	18	10	22	1.1%	1.20 [0.34, 4.18]	2011
Lu,Z.2011	18	34	17	34	1.9%	1.13 [0.43, 2.91]	2011
Wu,Q.2011	17	30	11	30	1.1%	2.26 [0.80, 6.36]	2011
Sun,J.2012	15	34	13	34	1.7%	1.28 [0.48, 3.36]	2012
Fu, L.2012	19	35	16	35	1.7%	1.41 [0.55, 3.61]	2012
Pei, W.2012	15	40	11	40	1.6%	1.58 [0.62, 4.07]	2012
Ju,S.2013	19	34	12	34	1.3%	2.32 [0.87, 6.16]	2013
Li, H.2014	16	30	12	30	1.3%	1.71 [0.62, 4.77]	2014
Wen, H.2014	28	45	17	45	1.5%	2.71 [1.16, 6.36]	2014
Cheng, B.2014	12	49	16	52	2.8%	0.73 [0.30, 1.76]	2014
Liu,Y.2014	18	43	17	43	2.3%	1.10 [0.47, 2.60]	2014
Zhang, M.2015	17	39	12	32	1.8%	1.29 [0.50, 3.35]	2015
Chen, Y.2016	15	30	13	30	1.5%	1.31 [0.47, 3.61]	2016
Fang, L.2016	35	45	27	45	1.4%	2.33 [0.93, 5.87]	2016
Su, S.2017	35	40	23	39	0.7%	4.87 [1.57, 15.13]	2017
Liu, H.2019	19	44	12	44	1.6%	2.03 [0.83, 4.95]	2019
Zhao,J.2019	31	43	22	43	1.5%	2.47 [1.01, 6.04]	2019
Tan, H.2020	24	60	18	60	2.6%	1.56 [0.73, 3.31]	2020
Xu,Z.2020	25	40	14	40	1.2%	3.10 [1.24, 7.71]	2020
**Subtotal (95% CI)**		**1252**		**1209**	**53.5%**	**1.65 [1.40, 1.94]**	
Total events	654		485				
Heterogeneity: Chi² = 26.66, df = 31 (P = 0.69); I² = 0%							
Test for overall effect: Z = 6.02 (P < 0.00001)							
**Treatment cycle(Three cycles)**							
Zhao,H.2008	11	30	6	20	1.1%	1.35 [0.40, 4.53]	2008
Wang,J.2012	14	25	10	24	1.1%	1.78 [0.57, 5.53]	2012
Zhang,Y. 2012	21	41	15	42	1.7%	1.89 [0.78, 4.55]	2012
Xu, H.2013	19	38	18	42	2.0%	1.33 [0.55, 3.22]	2013
Han, R.2015	14	36	10	36	1.4%	1.65 [0.61, 4.45]	2015
Ning, S.2015	19	31	11	31	1.0%	2.88 [1.03, 8.07]	2015
Huang, W.2017	17	39	11	40	1.5%	2.04 [0.80, 5.21]	2017
Xu, Y.2020	16	45	12	45	1.8%	1.52 [0.62, 3.73]	2020
**Subtotal (95% CI)**		**285**		**280**	**11.6%**	**1.75 [1.24, 2.47]**	
Total events	131		93				
Heterogeneity: Chi² = 1.68, df = 7 (P = 0.98); I² = 0%							
Test for overall effect: Z = 3.18 (P = 0.001)							
**Treatment cycle(Four cycles)**							
Liu,Y.2010	16	32	10	32	1.2%	2.20 [0.79, 6.10]	2010
Xu,Y.2012	16	33	16	33	2.0%	1.00 [0.38, 2.63]	2012
Li, J.2014	11	27	10	27	1.4%	1.17 [0.39, 3.49]	2014
Zhang, X.2016	18	25	8	25	0.5%	5.46 [1.63, 18.36]	2016
Ma, M.2017	19	42	12	42	1.6%	2.07 [0.84, 5.10]	2017
Zhang, H.2017	42	52	26	52	1.2%	4.20 [1.75, 10.11]	2017
Wu, T.2017	53	67	40	68	2.0%	2.65 [1.24, 5.68]	2017
Geng,K.2020	27	45	17	45	1.6%	2.47 [1.06, 5.77]	2020
**Subtotal (95% CI)**		**323**		**324**	**11.4%**	**2.32 [1.68, 3.22]**	
Total events	202		139				
Heterogeneity: Chi² = 8.31, df = 7 (P = 0.31); I² = 16%							
Test for overall effect: Z = 5.09 (P < 0.00001)							
**Treatment cycle(Others)**							
Fan,S.2011	22	41	12	38	1.4%	2.51 [1.00, 6.29]	2011
He,W.2011	15	29	10	23	1.3%	1.39 [0.46, 4.18]	2011
Wang,Y.2012	16	36	14	36	1.8%	1.26 [0.49, 3.21]	2012
Lai,L.2013	45	70	40	70	3.4%	1.35 [0.68, 2.67]	2013
Zhao, S.2015	19	43	12	43	1.6%	2.05 [0.83, 5.02]	2015
Zhu, H.2015	15	21	7	21	0.5%	5.00 [1.35, 18.55]	2015
Lv, W.2018	16	30	15	30	1.7%	1.14 [0.41, 3.15]	2018
Zhou, D.2018	30	58	22	58	2.5%	1.75 [0.84, 3.67]	2018
Su,D.2019	30	41	17	41	1.1%	3.85 [1.52, 9.75]	2019
Chen,C.2020	26	49	16	49	1.8%	2.33 [1.03, 5.29]	2020
**Subtotal (95% CI)**		**418**		**409**	**17.0%**	**1.91 [1.44, 2.52]**	
Total events	234		165				
Heterogeneity: Chi² = 7.96, df = 9 (P = 0.54); I² = 0%							
Test for overall effect: Z = 4.53 (P < 0.00001)							
**Total (95% CI)**		**2454**		**2397**	**100.0%**	**1.82 [1.62, 2.04]**	
Total events	1319		946				
Heterogeneity: Chi² = 52.84, df = 62 (P = 0.79); I² = 0%							
Test for overall effect: Z = 10.08 (P < 0.00001)							
Test for subgroup differences: Chi² = 4.58, df = 4 (P = 0.33), I² = 12.6%							

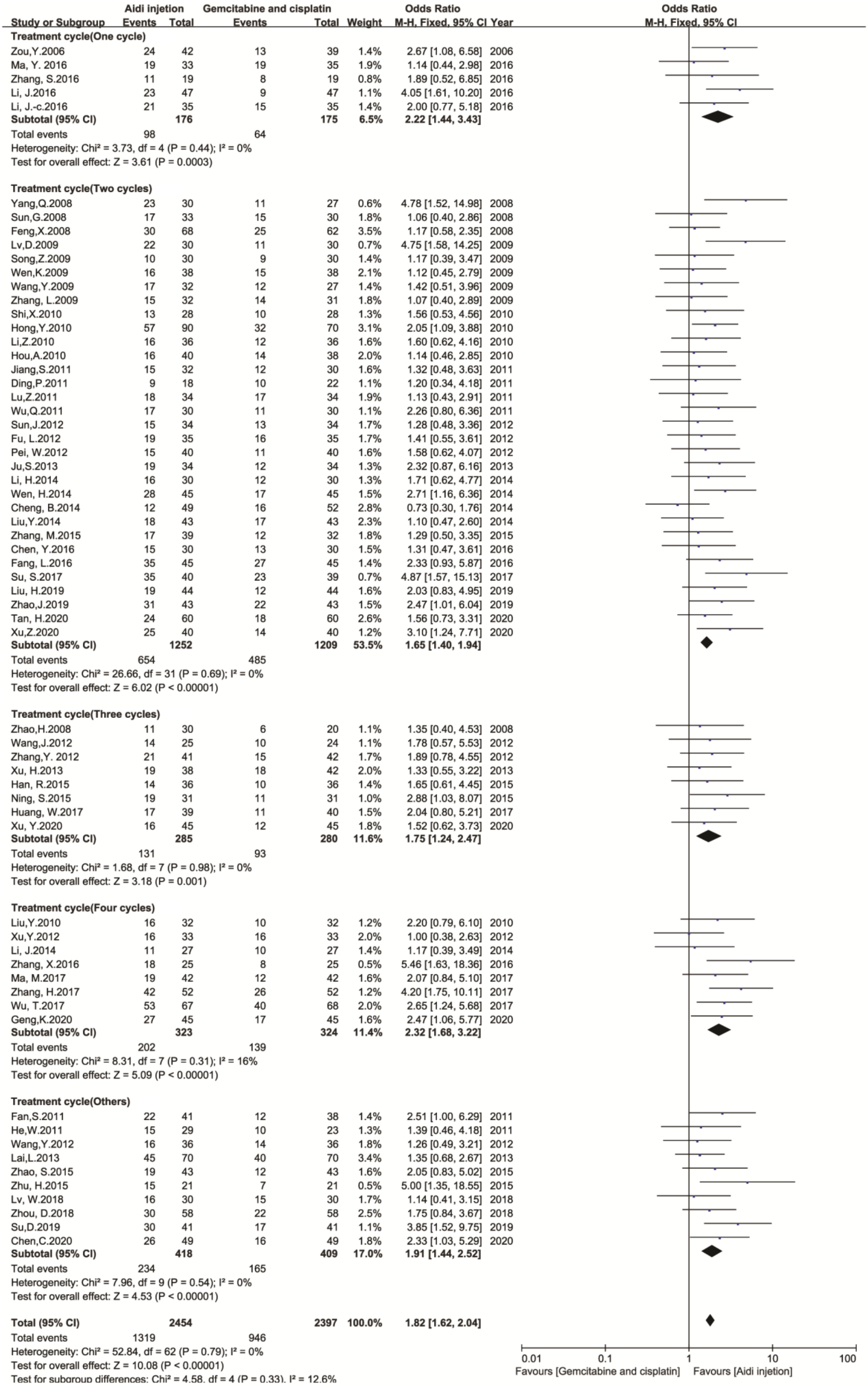

Figure S83 Subgroups analysis of objective response rate by treatment cycle

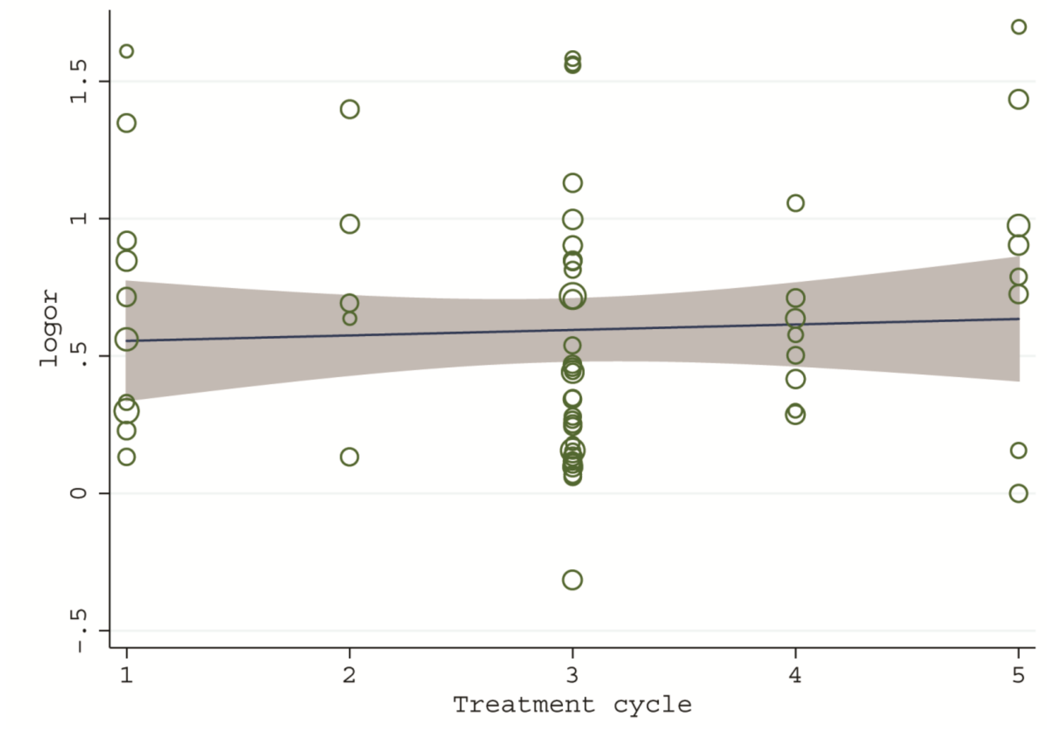

Figure S84 Meta-regression of objective response rate by treatment cycle

Study or Subgroup	Aidi injetion Events	Aidi injetion Total	Gemcitabine and cisplatin Events	Gemcitabine and cisplatin Total	Weight	Odds Ratio M-H, Fixed, 95% CI	Year
**Treatment cycle(One cycle)**							
Zou,Y.2006	35	42	25	39	1.9%	2.80 [0.99, 7.94]	2006
Ma, Y. 2016	28	33	28	35	1.9%	1.40 [0.40, 4.94]	2016
Li, J.-c.2016	31	35	26	35	1.3%	2.68 [0.74, 9.73]	2016
Li, J.2016	45	47	34	47	0.7%	8.60 [1.82, 40.69]	2016
Zhang, S.2016	14	19	14	19	1.7%	1.00 [0.24, 4.24]	2016
**Subtotal (95% CI)**		**176**		**175**	**7.4%**	**2.54 [1.46, 4.41]**	
Total events	153		127				
Heterogeneity: Chi² = 4.86, df = 4 (P = 0.30); I² = 18%							
Test for overall effect: Z = 3.31 (P = 0.0009)							
**Treatment cycle(Two cycles)**							
Yang,Q.2008	29	30	23	27	0.4%	5.04 [0.53, 48.26]	2008
Feng,X.2008	62	68	56	62	2.3%	1.11 [0.34, 3.63]	2008
Sun,G.2008	31	33	27	30	0.8%	1.72 [0.27, 11.09]	2008
Song,Z.2009	27	30	25	30	1.1%	1.80 [0.39, 8.32]	2009
Wen,K.2009	32	38	32	38	2.3%	1.00 [0.29, 3.43]	2009
Wang,Y.2009	29	32	21	27	1.0%	2.76 [0.62, 12.32]	2009
Lv,D.2009	28	30	23	30	0.7%	4.26 [0.81, 22.53]	2009
Zhang, L.2009	27	32	23	31	1.6%	1.88 [0.54, 6.54]	2009
Hou,A.2010	37	40	29	38	1.0%	3.83 [0.95, 15.43]	2010
Li,Z.2010	18	36	21	36	4.7%	0.71 [0.28, 1.81]	2010
Shi,X.2010	27	28	25	28	0.4%	3.24 [0.32, 33.22]	2010
Hong,Y.2010	78	90	50	70	3.4%	2.60 [1.17, 5.78]	2010
Lu,Z.2011	30	34	28	34	1.5%	1.61 [0.41, 6.30]	2011
Jiang,S.2011	29	32	25	30	1.1%	1.93 [0.42, 8.91]	2011
Wu,Q.2011	28	30	26	30	0.8%	2.15 [0.36, 12.76]	2011
Sun,J.2012	25	34	25	34	3.0%	1.00 [0.34, 2.94]	2012
Fu, L.2012	32	35	28	35	1.1%	2.67 [0.63, 11.31]	2012
Pei, W.2012	35	40	27	40	1.5%	3.37 [1.07, 10.61]	2012
Ju,S.2013	32	34	29	34	0.8%	2.76 [0.50, 15.33]	2013
Li, H.2014	26	30	23	30	1.4%	1.98 [0.51, 7.63]	2014
Wen, H.2014	43	45	36	45	0.7%	5.38 [1.09, 26.49]	2014
Cheng, B.2014	39	49	42	52	3.7%	0.93 [0.35, 2.47]	2014
Liu,Y.2014	35	43	33	43	2.8%	1.33 [0.47, 3.77]	2014
Zhang, M.2015	28	39	23	32	3.2%	1.00 [0.35, 2.82]	2015
Chen, Y.2016	24	30	18	30	1.6%	2.67 [0.84, 8.46]	2016
Fang, L.2016	42	45	34	45	1.0%	4.53 [1.17, 17.55]	2016
Su, S.2017	37	40	26	39	0.9%	6.17 [1.60, 23.83]	2017
Zhao,J.2019	39	43	34	43	1.4%	2.58 [0.73, 9.14]	2019
Liu, H.2019	39	44	30	44	1.5%	3.64 [1.18, 11.23]	2019
Xu,Z.2020	33	40	24	40	1.9%	3.14 [1.12, 8.82]	2020
Tan, H.2020	49	60	39	60	3.2%	2.40 [1.03, 5.57]	2020
**Subtotal (95% CI)**		**1234**		**1187**	**52.8%**	**2.08 [1.67, 2.58]**	
Total events	1070		905				
Heterogeneity: Chi² = 25.36, df = 30 (P = 0.71); I² = 0%							
Test for overall effect: Z = 6.63 (P < 0.00001)							
**Treatment cycle(Three cycles)**							
Zhang,Y. 2012	34	41	32	42	2.4%	1.52 [0.52, 4.47]	2012
Wang,J.2012	20	25	16	24	1.5%	2.00 [0.55, 7.31]	2012
Xu, H.2013	33	38	34	42	1.9%	1.55 [0.46, 5.24]	2013
Ning, S.2015	26	31	22	31	1.6%	2.13 [0.62, 7.29]	2015
Han, R.2015	32	36	27	36	1.3%	2.67 [0.74, 9.63]	2015
Huang, W.2017	36	39	30	40	1.0%	4.00 [1.01, 15.87]	2017
Xu, Y.2020	43	45	33	45	0.7%	7.82 [1.64, 37.36]	2020
**Subtotal (95% CI)**		**255**		**260**	**10.4%**	**2.48 [1.55, 3.96]**	
Total events	224		194				
Heterogeneity: Chi² = 4.07, df = 6 (P = 0.67); I² = 0%							
Test for overall effect: Z = 3.78 (P = 0.0002)							
**Treatment cycle(Four cycles)**							
Liu,Y.2010	25	32	22	32	2.2%	1.62 [0.53, 4.99]	2010
Xu,Y.2012	30	33	26	33	1.1%	2.69 [0.63, 11.49]	2012
Li, J.2014	19	27	16	27	2.1%	1.63 [0.53, 5.04]	2014
Zhang, X.2016	21	25	14	25	1.0%	4.13 [1.09, 15.59]	2016
Zhang, H.2017	50	52	46	52	0.8%	3.26 [0.63, 16.97]	2017
Wu, T.2017	62	67	56	68	1.9%	2.66 [0.88, 8.02]	2017
Ma, M.2017	37	42	29	42	1.6%	3.32 [1.06, 10.37]	2017
Geng,K.2020	39	45	31	45	1.9%	2.94 [1.01, 8.53]	2020
**Subtotal (95% CI)**		**323**		**324**	**12.4%**	**2.59 [1.69, 3.95]**	
Total events	283		240				
Heterogeneity: Chi² = 2.09, df = 7 (P = 0.95); I² = 0%							
Test for overall effect: Z = 4.39 (P < 0.0001)							
**Treatment cycle(Others)**							
Fan,S.2011	35	41	29	38	2.0%	1.81 [0.58, 5.68]	2011
He,W.2011	26	29	19	23	1.0%	1.82 [0.36, 9.12]	2011
Wang,Y.2012	28	36	20	36	2.0%	2.80 [1.01, 7.80]	2012
Lai,L.2013	63	70	60	70	2.7%	1.50 [0.54, 4.20]	2013
Zhu, H.2015	19	21	14	21	0.6%	4.75 [0.85, 26.43]	2015
Zhao, S.2015	37	43	28	43	1.8%	3.30 [1.14, 9.60]	2015
Lv, W.2018	24	30	25	30	2.2%	0.80 [0.22, 2.97]	2018
Zhou, D.2018	50	58	38	58	2.4%	3.29 [1.31, 8.27]	2018
Su,D.2019	39	41	33	41	0.7%	4.73 [0.94, 23.82]	2019
Chen,C.2020	44	49	34	49	1.6%	3.88 [1.28, 11.74]	2020
**Subtotal (95% CI)**		**418**		**409**	**16.9%**	**2.53 [1.75, 3.64]**	
Total events	365		300				
Heterogeneity: Chi² = 6.69, df = 9 (P = 0.67); I² = 0%							
Test for overall effect: Z = 4.98 (P < 0.00001)							
**Total (95% CI)**		**2406**		**2355**	**100.0%**	**2.29 [1.97, 2.67]**	
Total events	2095		1766				
Heterogeneity: Chi² = 44.59, df = 60 (P = 0.93); I² = 0%							
Test for overall effect: Z = 10.58 (P < 0.00001)							
Test for subgroup differences: Chi² = 1.62, df = 4 (P = 0.80), I² = 0%							

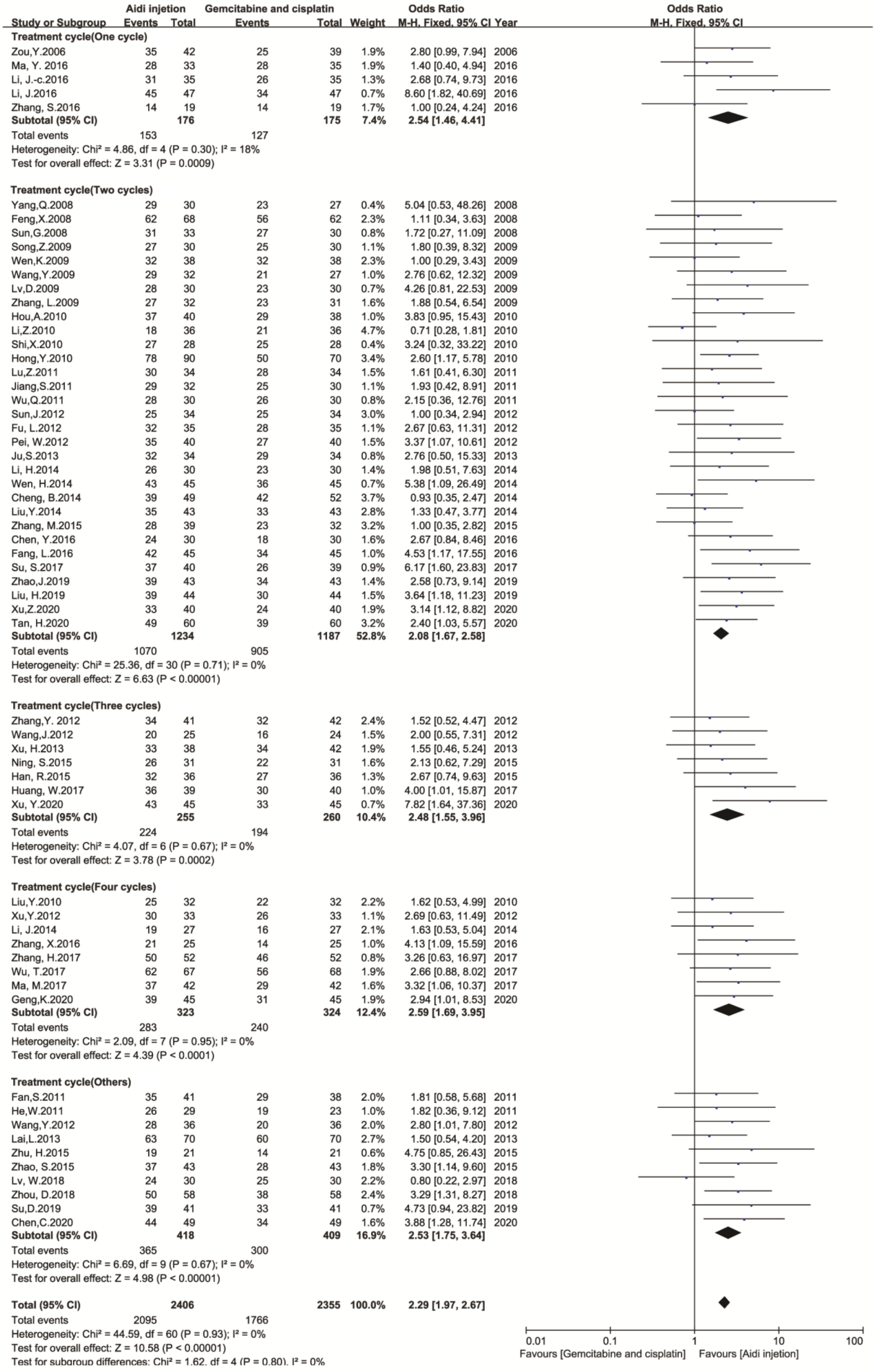

Figure S85 Subgroups analysis of disease control rate by treatment cycles

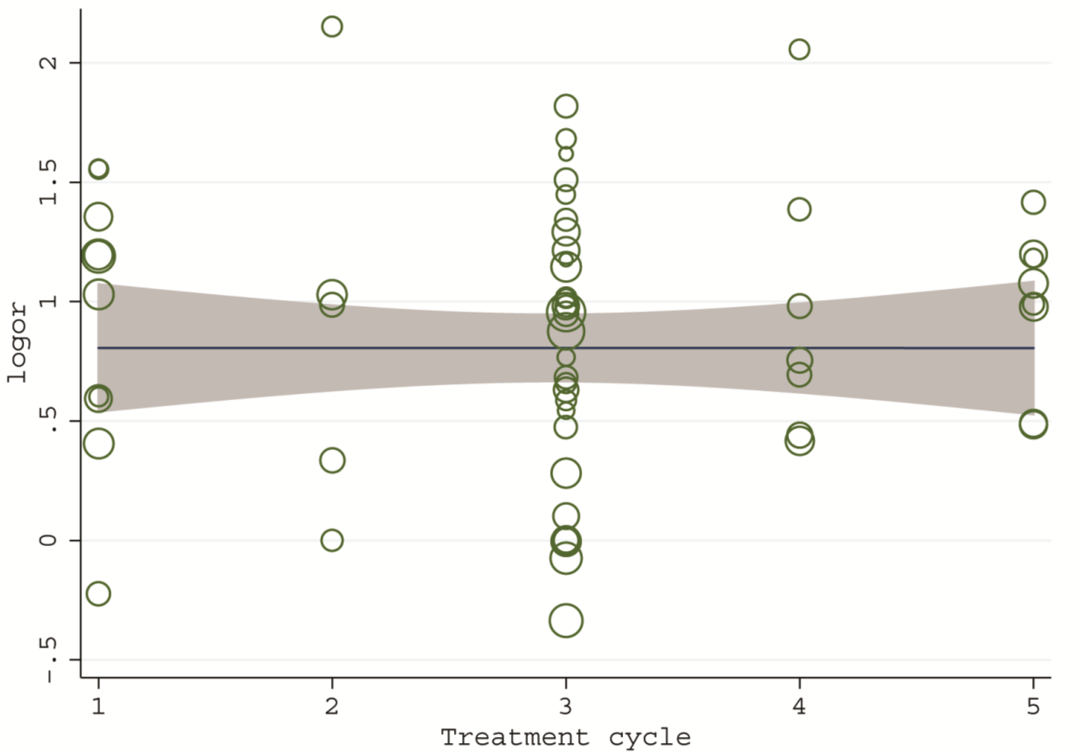

Figure S86 Meta-regression of disease control rate by treatment cycle

Study or Subgroup	Aidi injection Events	Aidi injection Total	Gemcitabine and cisplatin Events	Gemcitabine and cisplatin Total	Weight	Odds Ratio M-H, Fixed, 95% CI	Year
**Treatment cycle(One cycle)**							
Zhang, S.2016	2	19	4	19	0.8%	0.44 [0.07, 2.76]	2016
Li, J.-c.2016	14	35	26	35	3.6%	0.23 [0.08, 0.64]	2016
Ma, Y. 2016	7	33	15	35	2.7%	0.36 [0.12, 1.05]	2016
Li, J.2016	12	47	19	47	3.3%	0.51 [0.21, 1.21]	2016
**Subtotal (95% CI)**		**134**		**136**	**10.4%**	**0.37 [0.21, 0.63]**	
Total events	35		64				
Heterogeneity: Chi² = 1.35, df = 3 (P = 0.72); I² = 0%							
Test for overall effect: Z = 3.66 (P = 0.0002)							
**Treatment cycle(Two cycles)**							
Feng,X.2008	64	68	60	62	0.9%	0.53 [0.09, 3.02]	2008
Wen,K.2009	23	38	31	38	2.8%	0.35 [0.12, 0.99]	2009
Zhang, L.2009	5	32	14	31	2.8%	0.22 [0.07, 0.74]	2009
Wang,Y.2009	26	32	22	27	1.0%	0.98 [0.26, 3.67]	2009
Hou,A.2010	14	40	22	38	3.4%	0.39 [0.16, 0.98]	2010
Hong,Y.2010	26	90	31	70	5.7%	0.51 [0.27, 0.98]	2010
Lu,Z.2011	11	34	20	34	3.1%	0.33 [0.12, 0.90]	2011
Fu, L.2012	24	35	32	35	2.3%	0.20 [0.05, 0.81]	2012
Sun,J.2012	23	34	30	34	2.2%	0.28 [0.08, 0.99]	2012
Cheng, B.2014	34	49	47	52	3.2%	0.24 [0.08, 0.73]	2014
Liu,Y.2014	27	43	36	43	3.1%	0.33 [0.12, 0.91]	2014
Zhang, M.2015	26	39	28	32	2.4%	0.29 [0.08, 0.99]	2015
Li, X.2015	2	20	8	20	1.7%	0.17 [0.03, 0.92]	2015
Fang, L.2016	5	45	13	45	2.7%	0.31 [0.10, 0.95]	2016
Su, S.2017	2	40	2	39	0.4%	0.97 [0.13, 7.28]	2017
Zhang, H.2018	13	30	22	30	2.9%	0.28 [0.09, 0.82]	2018
Tan, H.2020	51	60	57	60	2.0%	0.30 [0.08, 1.16]	2020
Xu,Z.2020	2	40	6	40	1.3%	0.30 [0.06, 1.58]	2020
**Subtotal (95% CI)**		**769**		**730**	**44.0%**	**0.35 [0.27, 0.45]**	
Total events	378		481				
Heterogeneity: Chi² = 7.77, df = 17 (P = 0.97); I² = 0%							
Test for overall effect: Z = 7.78 (P < 0.00001)							
**Treatment cycle(Three cycles)**							
Zhang,Y. 2012	12	41	17	42	2.7%	0.61 [0.24, 1.52]	2012
Wang,J.2012	7	25	9	24	1.5%	0.65 [0.19, 2.16]	2012
Xu, H.2013	13	38	22	42	3.2%	0.47 [0.19, 1.17]	2013
Han, R.2015	7	36	11	36	2.0%	0.55 [0.18, 1.63]	2015
Ning, S.2015	16	31	16	31	1.8%	1.00 [0.37, 2.71]	2015
Huang, W.2017	10	39	12	40	2.0%	0.80 [0.30, 2.16]	2017
Wu, Y.2017	3	109	9	109	2.0%	0.31 [0.08, 1.19]	2017
**Subtotal (95% CI)**		**319**		**324**	**15.4%**	**0.61 [0.41, 0.90]**	
Total events	68		96				
Heterogeneity: Chi² = 2.55, df = 6 (P = 0.86); I² = 0%							
Test for overall effect: Z = 2.51 (P = 0.01)							
**Treatment cycle(Four cycles)**							
Xu,Y.2012	23	33	33	33	2.4%	0.03 [0.00, 0.60]	2012
Li, J.2014	5	27	6	27	1.1%	0.80 [0.21, 3.00]	2014
Zhang, H.2017	13	52	22	52	3.8%	0.45 [0.20, 1.05]	2017
Wu, T.2017	10	67	21	68	4.1%	0.39 [0.17, 0.92]	2017
Ma, M.2017	11	42	16	42	2.7%	0.58 [0.23, 1.46]	2017
Guo, X.2020	7	51	16	51	3.2%	0.35 [0.13, 0.94]	2020
**Subtotal (95% CI)**		**272**		**273**	**17.4%**	**0.40 [0.27, 0.61]**	
Total events	69		114				
Heterogeneity: Chi² = 4.60, df = 5 (P = 0.47); I² = 0%							
Test for overall effect: Z = 4.36 (P < 0.0001)							
**Treatment cycle(Others)**							
Wang,Y.2012	19	36	26	36	2.8%	0.43 [0.16, 1.15]	2012
Zhao, S.2015	7	43	10	43	1.9%	0.64 [0.22, 1.88]	2015
Zhu, H.2015	6	21	15	21	2.5%	0.16 [0.04, 0.61]	2015
Zhou, D.2018	11	58	13	58	2.4%	0.81 [0.33, 1.99]	2018
Chen,C.2020	33	49	43	49	3.2%	0.29 [0.10, 0.82]	2020
**Subtotal (95% CI)**		**207**		**207**	**12.9%**	**0.45 [0.28, 0.70]**	
Total events	76		107				
Heterogeneity: Chi² = 5.06, df = 4 (P = 0.28); I² = 21%							
Test for overall effect: Z = 3.46 (P = 0.0005)							
**Total (95% CI)**		**1701**		**1670**	**100.0%**	**0.41 [0.35, 0.49]**	
Total events	626		862				
Heterogeneity: Chi² = 26.97, df = 39 (P = 0.93); I² = 0%							
Test for overall effect: Z = 10.32 (P < 0.00001)							
Test for subgroup differences: Chi² = 5.81, df = 4 (P = 0.21), I² = 31.1%							

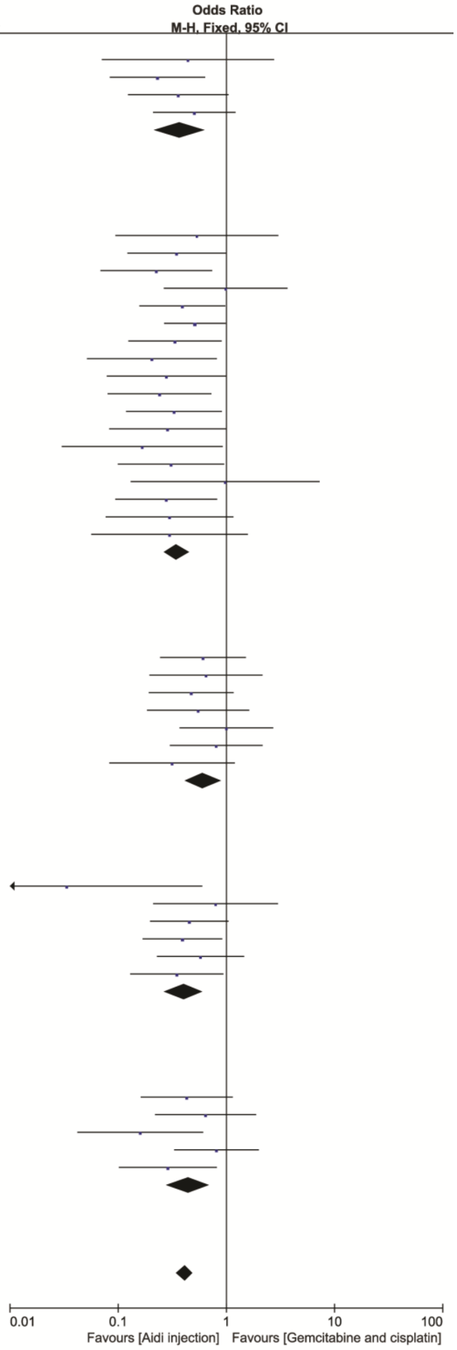

Figure S87 Subgroups analysis of neutropenia by treatment cycle

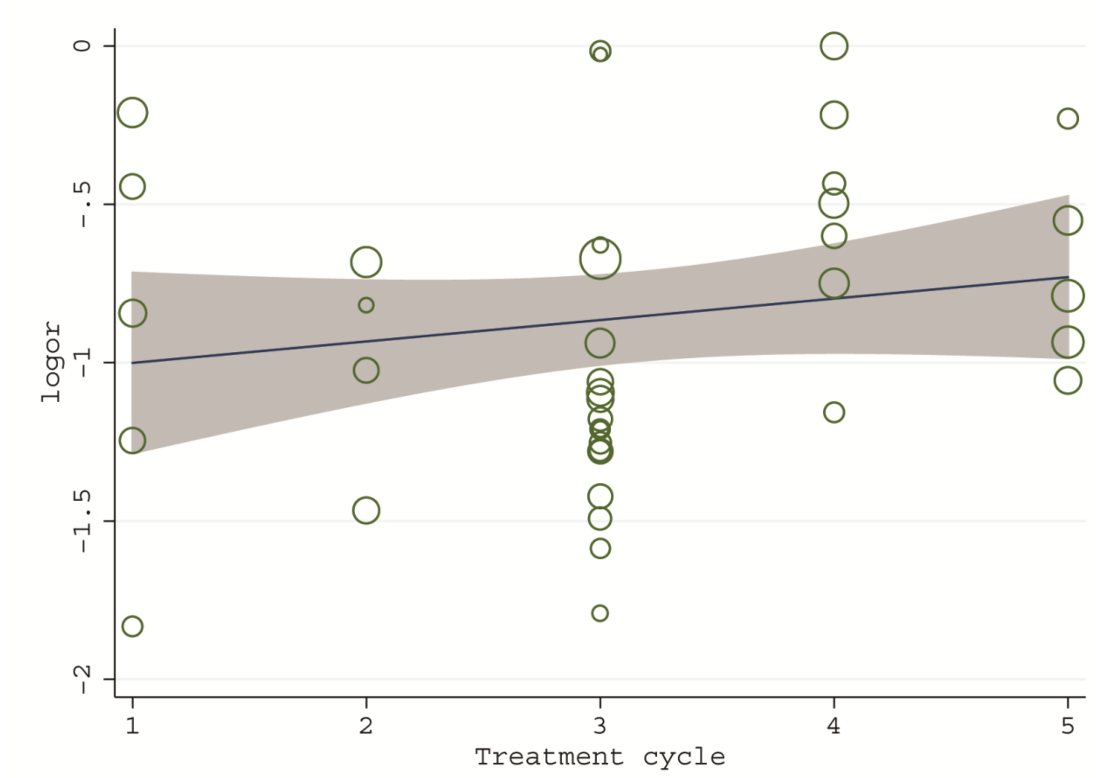

Figure S88 Meta-regression of neutropenia by treatment cycle

Study or Subgroup	Aidi injetion Events	Aidi injetion Total	Gemcitabine and cisplatin Events	Gemcitabine and cisplatin Total	Weight	Odds Ratio M-H, Fixed, 95% CI	Year
**Treatment cycle(One cycle)**							
Zou,Y.2006	32	42	29	39	1.2%	1.10 [0.40, 3.03]	2006
Ma, Y. 2016	5	33	13	35	1.8%	0.30 [0.09, 0.98]	2016
Zhang, S.2016	2	19	3	19	0.5%	0.63 [0.09, 4.26]	2016
Li, J.2016	15	47	27	47	3.2%	0.35 [0.15, 0.81]	2016
Li, J.-c.2016	7	35	19	35	2.6%	0.21 [0.07, 0.61]	2016
**Subtotal (95% CI)**		**176**		**175**	**9.3%**	**0.41 [0.26, 0.66]**	
Total events	61		91				
Heterogeneity: Chi² = 5.80, df = 4 (P = 0.21); I² = 31%							
Test for overall effect: Z = 3.65 (P = 0.0003)							
**Treatment cycle(Two cycles)**							
Feng,X.2008	55	68	54	62	1.9%	0.63 [0.24, 1.63]	2008
Wen,K.2009	20	38	29	38	2.4%	0.34 [0.13, 0.92]	2009
Lv,D.2009	10	30	19	30	2.2%	0.29 [0.10, 0.84]	2009
Song,Z.2009	10	30	11	30	1.3%	0.86 [0.30, 2.50]	2009
Zhang, L.2009	11	32	20	31	2.3%	0.29 [0.10, 0.81]	2009
Wang,Y.2009	5	32	5	27	0.8%	0.81 [0.21, 3.18]	2009
Hong,Y.2010	20	90	29	70	4.4%	0.40 [0.20, 0.80]	2010
Li,Z.2010	16	36	20	36	1.9%	0.64 [0.25, 1.62]	2010
Shi,X.2010	12	28	20	28	2.0%	0.30 [0.10, 0.91]	2010
Hou,A.2010	5	40	12	38	1.8%	0.31 [0.10, 0.99]	2010
Lu,Z.2011	10	34	19	34	2.3%	0.33 [0.12, 0.90]	2011
Sun,J.2012	28	34	29	34	0.9%	0.80 [0.22, 2.94]	2012
Cai,H.2013	6	19	8	19	0.9%	0.63 [0.17, 2.40]	2013
Ju,S.2013	13	34	14	34	1.5%	0.88 [0.33, 2.34]	2013
Li, H.2014	18	30	22	30	1.5%	0.55 [0.18, 1.62]	2014
Wen, H.2014	23	45	39	45	3.3%	0.16 [0.06, 0.45]	2014
Liu,Y.2014	24	43	32	43	2.4%	0.43 [0.17, 1.08]	2014
Cheng, B.2014	7	49	7	52	1.0%	1.07 [0.35, 3.31]	2014
Li, X.2015	15	20	15	20	0.6%	1.00 [0.24, 4.18]	2015
Zhang, M.2015	32	39	27	32	0.9%	0.85 [0.24, 2.97]	2015
Fang, L.2016	6	45	14	45	2.1%	0.34 [0.12, 0.99]	2016
Su, S.2017	4	40	3	39	0.5%	1.33 [0.28, 6.39]	2017
Zhang, H.2018	7	40	18	40	2.5%	0.26 [0.09, 0.72]	2018
Tan, H.2020	47	60	54	60	2.0%	0.40 [0.14, 1.14]	2020
Xu,Z.2020	2	40	7	40	1.1%	0.25 [0.05, 1.28]	2020
**Subtotal (95% CI)**		**996**		**957**	**44.4%**	**0.46 [0.37, 0.57]**	
Total events	406		527				
Heterogeneity: Chi² = 21.13, df = 24 (P = 0.63); I² = 0%							
Test for overall effect: Z = 7.21 (P < 0.00001)							
**Treatment cycle(Three cycles)**							
Zhang,Y. 2012	9	41	14	42	1.9%	0.56 [0.21, 1.50]	2012
Wang,J.2012	5	25	8	24	1.1%	0.50 [0.14, 1.83]	2012
Xu, H.2013	20	38	30	42	2.3%	0.44 [0.18, 1.12]	2013
Ning, S.2015	12	31	16	31	1.7%	0.59 [0.22, 1.62]	2015
Wu, Y.2017	37	109	63	109	7.1%	0.38 [0.22, 0.65]	2017
**Subtotal (95% CI)**		**244**		**248**	**14.1%**	**0.45 [0.31, 0.65]**	
Total events	83		131				
Heterogeneity: Chi² = 0.93, df = 4 (P = 0.92); I² = 0%							
Test for overall effect: Z = 4.21 (P < 0.0001)							
**Treatment cycle(Four cycles)**							
Liu,Y.2010	7	32	14	32	1.9%	0.36 [0.12, 1.07]	2010
Xu,Y.2012	7	33	18	33	2.4%	0.22 [0.08, 0.66]	2012
Wu, T.2017	11	67	23	68	3.3%	0.38 [0.17, 0.87]	2017
Zhang, H.2017	16	52	29	52	3.4%	0.35 [0.16, 0.79]	2017
Guo, X.2020	5	51	13	51	2.0%	0.32 [0.10, 0.97]	2020
Geng,K.2020	21	45	30	45	2.7%	0.44 [0.19, 1.03]	2020
**Subtotal (95% CI)**		**280**		**281**	**15.8%**	**0.35 [0.24, 0.51]**	
Total events	67		127				
Heterogeneity: Chi² = 1.00, df = 5 (P = 0.96); I² = 0%							
Test for overall effect: Z = 5.41 (P < 0.00001)							
**Treatment cycle(Others)**							
He,W.2011	12	29	13	23	1.5%	0.54 [0.18, 1.64]	2011
Wang,Y.2012	21	36	28	36	2.0%	0.40 [0.14, 1.12]	2012
Lai,L.2013	20	70	28	70	3.4%	0.60 [0.30, 1.21]	2013
Zhu, H.2015	7	21	16	21	1.8%	0.16 [0.04, 0.60]	2015
Zhao, S.2015	5	43	10	43	1.5%	0.43 [0.13, 1.40]	2015
Zhou, D.2018	29	58	31	58	2.7%	0.87 [0.42, 1.81]	2018
Su,D.2019	5	41	15	41	2.3%	0.24 [0.08, 0.75]	2019
Chen,C.2020	40	49	42	49	1.3%	0.74 [0.25, 2.18]	2020
**Subtotal (95% CI)**		**347**		**341**	**16.5%**	**0.51 [0.37, 0.72]**	
Total events	139		183				
Heterogeneity: Chi² = 7.66, df = 7 (P = 0.36); I² = 9%							
Test for overall effect: Z = 3.91 (P < 0.0001)							
**Total (95% CI)**		**2043**		**2002**	**100.0%**	**0.45 [0.39, 0.51]**	
Total events	756		1059				
Heterogeneity: Chi² = 38.98, df = 48 (P = 0.82); I² = 0%							
Test for overall effect: Z = 11.21 (P < 0.00001)							
Test for subgroup differences: Chi² = 2.39, df = 4 (P = 0.67), I² = 0%							

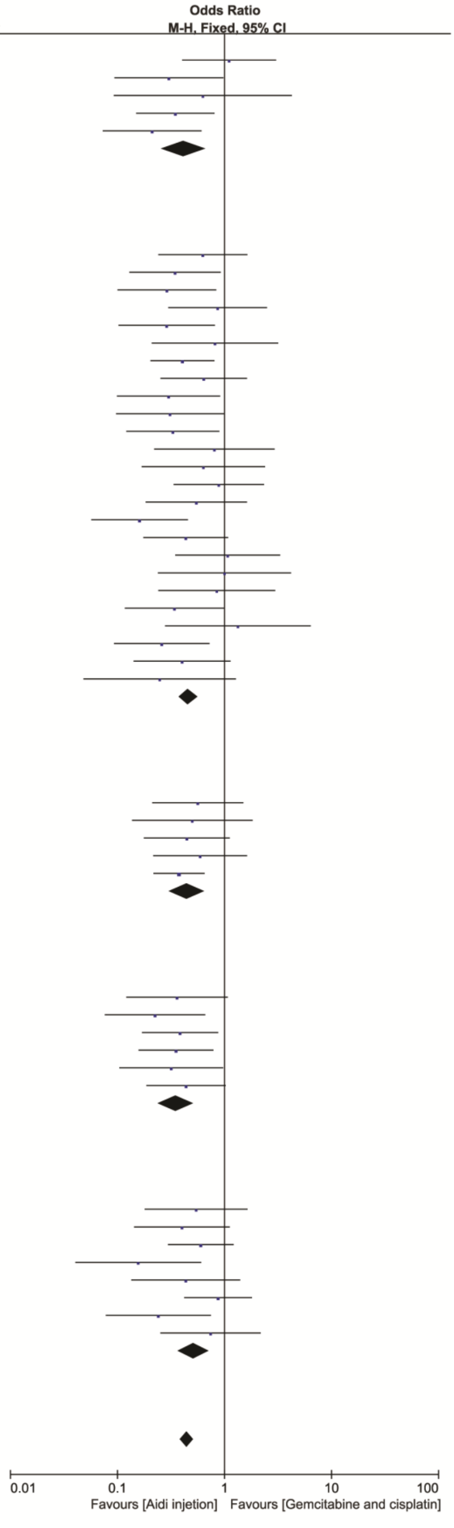

Figure S89 Subgroups analysis of gastrointestinal reaction by treatment cycle

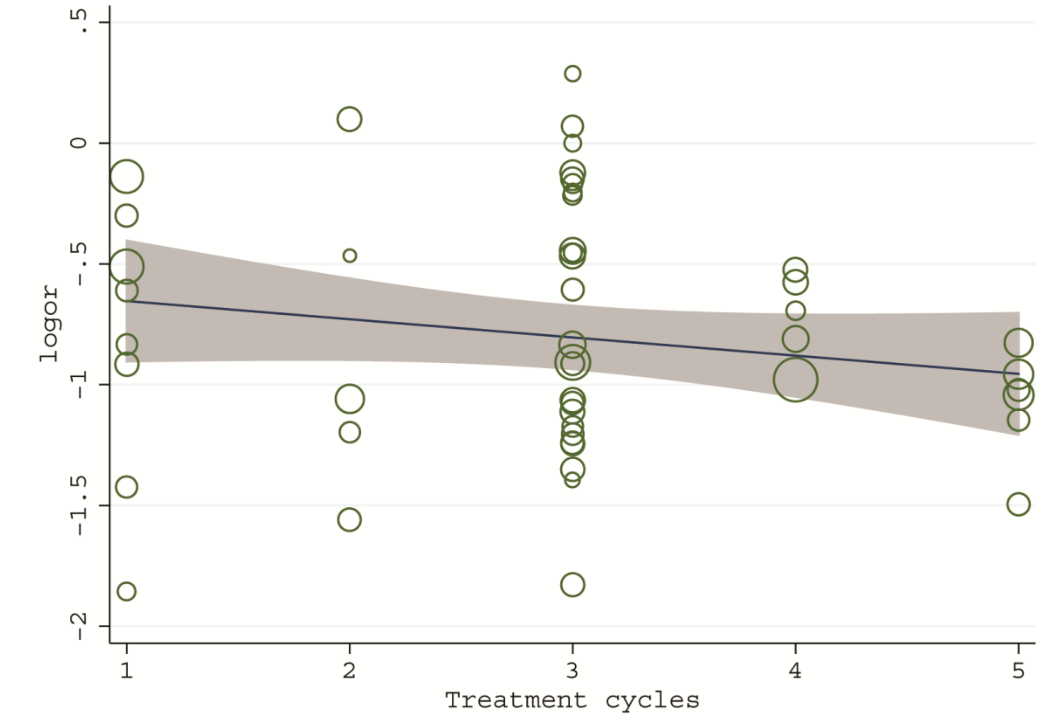

Figure S90 Meta-regression of gastrointestinal reaction by treatment cycle

Study or Subgroup	Aidi injection Events	Aidi injection Total	Gemcitabine and cisplatin Events	Gemcitabine and cisplatin Total	Weight	Odds Ratio M-H, Fixed, 95% CI	Year
**Cisplatin (20-30mg/m^2)**							
Zou,Y.2006	24	42	13	39	1.4%	2.67 [1.08, 6.58]	2006
Sun,G.2008	17	33	15	30	1.8%	1.06 [0.40, 2.86]	2008
Wang,Y.2009	17	32	12	27	1.4%	1.42 [0.51, 3.96]	2009
Song,Z.2009	10	30	9	30	1.4%	1.17 [0.39, 3.47]	2009
Liu,Y.2010	16	32	10	32	1.2%	2.20 [0.79, 6.10]	2010
Li,Z.2010	16	36	12	36	1.6%	1.60 [0.62, 4.16]	2010
Shi,X.2010	13	28	10	28	1.3%	1.56 [0.53, 4.56]	2010
Hong,Y.2010	57	90	32	70	3.1%	2.05 [1.09, 3.88]	2010
Hou,A.2010	16	40	14	38	2.0%	1.14 [0.46, 2.85]	2010
Wu,Q.2011	17	30	11	30	1.1%	2.26 [0.80, 6.36]	2011
Fan,S.2011	22	41	12	38	1.4%	2.51 [1.00, 6.29]	2011
Zhang,Y. 2012	21	41	15	42	1.7%	1.89 [0.78, 4.55]	2012
Wang,J.2012	14	25	10	24	1.1%	1.78 [0.57, 5.53]	2012
Pei, W.2012	15	40	11	40	1.6%	1.58 [0.62, 4.07]	2012
Fu, L.2012	19	35	16	35	1.7%	1.41 [0.55, 3.61]	2012
Sun,J.2012	15	34	13	34	1.7%	1.28 [0.48, 3.36]	2012
Lai,L.2013	45	70	40	70	3.4%	1.35 [0.68, 2.67]	2013
Xu, H.2013	19	38	18	42	2.0%	1.33 [0.55, 3.22]	2013
Cheng, B.2014	12	49	16	52	2.8%	0.73 [0.30, 1.76]	2014
Zhu, H.2015	15	21	7	21	0.5%	5.00 [1.35, 18.55]	2015
Zhao, S.2015	19	43	12	43	1.6%	2.05 [0.83, 5.02]	2015
Zhang, M.2015	17	39	12	32	1.8%	1.29 [0.50, 3.35]	2015
Zhang, S.2016	11	19	8	19	0.8%	1.89 [0.52, 6.85]	2016
Chen, Y.2016	15	30	13	30	1.5%	1.31 [0.47, 3.61]	2016
Ma, Y. 2016	19	33	19	35	1.9%	1.14 [0.44, 2.98]	2016
Li, J.2016	23	47	9	47	1.1%	4.05 [1.61, 10.20]	2016
Wu, T.2017	53	67	40	68	2.0%	2.65 [1.24, 5.68]	2017
Zhang, H.2017	42	52	26	52	1.2%	4.20 [1.75, 10.11]	2017
Huang, W.2017	17	39	11	40	1.5%	2.04 [0.80, 5.21]	2017
Su, S.2017	35	40	23	39	0.7%	4.87 [1.57, 15.13]	2017
Zhou, D.2018	30	58	22	58	2.5%	1.75 [0.84, 3.67]	2018
Lv, W.2018	16	30	15	30	1.7%	1.14 [0.41, 3.15]	2018
Liu, H.2019	19	44	12	44	1.6%	2.03 [0.83, 4.95]	2019
Chen,C.2020	26	49	16	49	1.8%	2.33 [1.03, 5.29]	2020
Geng,K.2020	27	45	17	45	1.6%	2.47 [1.06, 5.77]	2020
Tan, H.2020	24	60	18	60	2.6%	1.56 [0.73, 3.31]	2020
Xu, Y.2020	16	45	12	45	1.8%	1.52 [0.62, 3.73]	2020
Xu,Z.2020	25	40	14	40	1.2%	3.10 [1.24, 7.71]	2020
**Subtotal (95% CI)**		**1567**		**1534**	**62.9%**	**1.83 [1.58, 2.12]**	
Total events	834		595				
Heterogeneity: Chi² = 28.77, df = 37 (P = 0.83); I² = 0%							
Test for overall effect: Z = 8.12 (P < 0.00001)							
**Cisplatin (40-50mg/m^2)**							
Ding,P.2011	9	18	10	22	1.1%	1.20 [0.34, 4.18]	2011
Wang,Y.2012	16	36	14	36	1.8%	1.26 [0.49, 3.21]	2012
Ju,S.2013	19	34	12	34	1.3%	2.32 [0.87, 6.16]	2013
Li, H.2014	16	30	12	30	1.3%	1.71 [0.62, 4.77]	2014
Wen, H.2014	28	45	17	45	1.5%	2.71 [1.16, 6.36]	2014
Liu,Y.2014	18	43	17	43	2.3%	1.10 [0.47, 2.60]	2014
Su,D.2019	30	41	17	41	1.1%	3.85 [1.52, 9.75]	2019
**Subtotal (95% CI)**		**247**		**251**	**10.4%**	**1.88 [1.32, 2.69]**	
Total events	136		99				
Heterogeneity: Chi² = 5.90, df = 6 (P = 0.43); I² = 0%							
Test for overall effect: Z = 3.48 (P = 0.0005)							
**Cisplatin (60-80mg/m^2)**							
Zhao,H.2008	11	30	6	20	1.1%	1.35 [0.40, 4.53]	2008
Yang,Q.2008	23	30	11	27	0.6%	4.78 [1.52, 14.98]	2008
Feng,X.2008	30	68	25	62	3.5%	1.17 [0.58, 2.35]	2008
Wen,K.2009	16	38	15	38	2.1%	1.12 [0.45, 2.79]	2009
Lv,D.2009	22	30	11	30	0.7%	4.75 [1.58, 14.25]	2009
Zhang, L.2009	15	32	14	31	1.8%	1.07 [0.40, 2.89]	2009
Jiang,S.2011	15	32	12	30	1.6%	1.32 [0.48, 3.63]	2011
Lu,Z.2011	18	34	17	34	1.9%	1.13 [0.43, 2.91]	2011
He,W.2011	15	29	10	23	1.3%	1.39 [0.46, 4.18]	2011
Li, J.2014	11	27	10	27	1.4%	1.17 [0.39, 3.49]	2014
Ning, S.2015	19	31	11	31	1.0%	2.88 [1.03, 8.07]	2015
Li, J.-c.2016	21	35	15	35	1.4%	2.00 [0.77, 5.18]	2016
Fang, L.2016	35	45	27	45	1.4%	2.33 [0.93, 5.87]	2016
Zhang, X.2016	18	25	8	25	0.5%	5.46 [1.63, 18.36]	2016
Ma, M.2017	19	42	12	42	1.6%	2.07 [0.84, 5.10]	2017
Zhao,J.2019	31	43	22	43	1.5%	2.47 [1.01, 6.04]	2019
**Subtotal (95% CI)**		**571**		**543**	**23.2%**	**1.83 [1.43, 2.32]**	
Total events	319		226				
Heterogeneity: Chi² = 16.63, df = 15 (P = 0.34); I² = 10%							
Test for overall effect: Z = 4.89 (P < 0.00001)							
**Cisplatin (others)**							
Xu,Y.2012	16	33	16	33	2.0%	1.00 [0.38, 2.63]	2012
Han, R.2015	14	36	10	36	1.4%	1.65 [0.61, 4.45]	2015
**Subtotal (95% CI)**		**69**		**69**	**3.4%**	**1.28 [0.64, 2.55]**	
Total events	30		26				
Heterogeneity: Chi² = 0.51, df = 1 (P = 0.48); I² = 0%							
Test for overall effect: Z = 0.70 (P = 0.48)							
**Total (95% CI)**		**2454**		**2397**	**100.0%**	**1.82 [1.62, 2.04]**	
Total events	1319		946				
Heterogeneity: Chi² = 52.84, df = 62 (P = 0.79); I² = 0%							
Test for overall effect: Z = 10.08 (P < 0.00001)							
Test for subgroup differences: Chi² = 1.06, df = 3 (P = 0.79), I² = 0%							

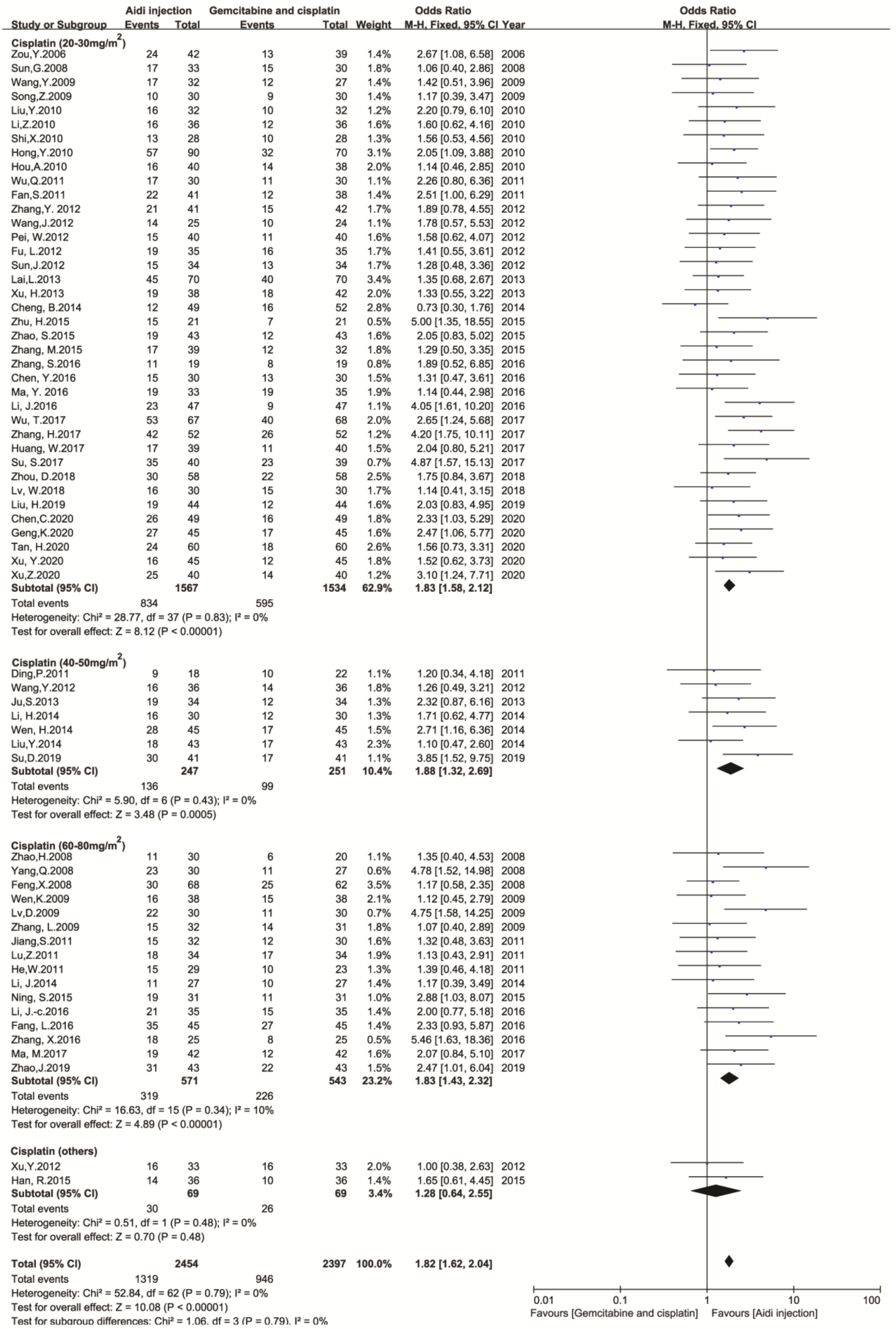

Figure S91 Subgroups analysis of objective response rate by cisplatin dosage

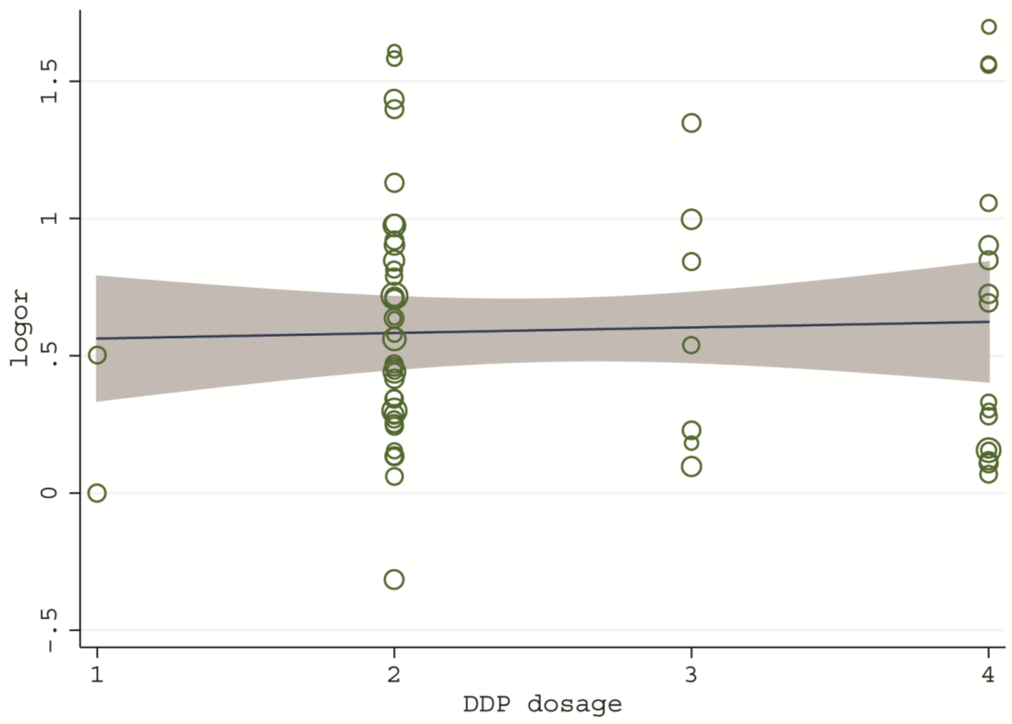

Figure S92 Meta-regression of objective response rate by cisplatin dosage

Study or Subgroup	Aidi injection Events	Aidi injection Total	Gemcitabine and cisplatin Events	Gemcitabine and cisplatin Total	Weight	Odds Ratio M-H, Fixed, 95% CI	Year
**Cisplatin (20-30mg/m²)**							
Zou,Y.2006	35	42	25	39	1.9%	2.80 [0.99, 7.94]	2006
Sun,G.2008	31	33	27	30	0.8%	1.72 [0.27, 11.09]	2008
Song,Z.2009	27	30	25	30	1.1%	1.80 [0.39, 8.32]	2009
Wang,Y.2009	29	32	21	27	1.0%	2.76 [0.62, 12.32]	2009
Hong,Y.2010	78	90	50	70	3.4%	2.60 [1.17, 5.78]	2010
Hou,A.2010	37	40	29	38	1.0%	3.83 [0.95, 15.43]	2010
Shi,X.2010	27	28	25	28	0.4%	3.24 [0.32, 33.22]	2010
Li,Z.2010	18	36	21	36	4.7%	0.71 [0.28, 1.81]	2010
Liu,Y.2010	25	32	22	32	2.2%	1.62 [0.53, 4.99]	2010
Fan,S.2011	35	41	29	38	2.0%	1.81 [0.58, 5.68]	2011
Wu,Q.2011	28	30	26	30	0.8%	2.15 [0.36, 12.76]	2011
Fu, L.2012	32	35	28	35	1.1%	2.67 [0.63, 11.31]	2012
Sun,J.2012	25	34	25	34	3.0%	1.00 [0.34, 2.94]	2012
Wang,J.2012	20	25	16	24	1.5%	2.00 [0.55, 7.31]	2012
Pei, W.2012	35	40	27	40	1.5%	3.37 [1.07, 10.61]	2012
Zhang,Y. 2012	34	41	32	42	2.4%	1.52 [0.52, 4.47]	2012
Xu, H.2013	33	38	34	42	1.9%	1.55 [0.46, 5.24]	2013
Lai,L.2013	63	70	60	70	2.7%	1.50 [0.54, 4.20]	2013
Cheng, B.2014	39	49	42	52	3.7%	0.93 [0.35, 2.47]	2014
Zhu, H.2015	19	21	14	21	0.6%	4.75 [0.85, 26.43]	2015
Zhang, M.2015	28	39	23	32	3.2%	1.00 [0.35, 2.82]	2015
Zhao, S.2015	37	43	28	43	1.8%	3.30 [1.14, 9.60]	2015
Chen, Y.2016	24	30	18	30	1.6%	2.67 [0.84, 8.46]	2016
Li, J.2016	45	47	34	47	0.7%	8.60 [1.82, 40.69]	2016
Zhang, S.2016	14	19	14	19	1.7%	1.00 [0.24, 4.24]	2016
Ma, Y. 2016	28	33	28	35	1.9%	1.40 [0.40, 4.94]	2016
Su, S.2017	37	40	26	39	0.9%	6.17 [1.60, 23.83]	2017
Zhang, H.2017	50	52	46	52	0.8%	3.26 [0.63, 16.97]	2017
Huang, W.2017	36	39	30	40	1.0%	4.00 [1.01, 15.87]	2017
Wu, T.2017	62	67	56	68	1.9%	2.66 [0.88, 8.02]	2017
Lv, W.2018	24	30	25	30	2.2%	0.80 [0.22, 2.97]	2018
Zhou, D.2018	50	58	38	58	2.4%	3.29 [1.31, 8.27]	2018
Liu, H.2019	39	44	30	44	1.5%	3.64 [1.18, 11.23]	2019
Tan, H.2020	49	60	39	60	3.2%	2.40 [1.03, 5.57]	2020
Xu, Y.2020	43	45	33	45	0.7%	7.82 [1.64, 37.36]	2020
Xu,Z.2020	33	40	24	40	1.9%	3.14 [1.12, 8.82]	2020
Chen,C.2020	44	49	34	49	1.6%	3.88 [1.28, 11.74]	2020
Geng,K.2020	39	45	31	45	1.9%	2.94 [1.01, 8.53]	2020
**Subtotal (95% CI)**		**1567**		**1534**	**68.3%**	**2.26 [1.87, 2.72]**	
Total events	1352		1135				
Heterogeneity: Chi² = 33.99, df = 37 (P = 0.61); I² = 0%							
Test for overall effect: Z = 8.57 (P < 0.00001)							
**Cisplatin (40-50mg/m²)**							
Wang,Y.2012	28	36	20	36	2.0%	2.80 [1.01, 7.80]	2012
Ju,S.2013	32	34	29	34	0.8%	2.76 [0.50, 15.33]	2013
Li, H.2014	26	30	23	30	1.4%	1.98 [0.51, 7.63]	2014
Liu,Y.2014	35	43	33	43	2.8%	1.33 [0.47, 3.77]	2014
Wen, H.2014	43	45	36	45	0.7%	5.38 [1.09, 26.49]	2014
Su,D.2019	39	41	33	41	0.7%	4.73 [0.94, 23.82]	2019
**Subtotal (95% CI)**		**229**		**229**	**8.4%**	**2.56 [1.52, 4.31]**	
Total events	203		174				
Heterogeneity: Chi² = 3.09, df = 5 (P = 0.69); I² = 0%							
Test for overall effect: Z = 3.54 (P = 0.0004)							
**Cisplatin (60-80mg/m²)**							
Feng,X.2008	62	68	56	62	2.3%	1.11 [0.34, 3.63]	2008
Yang,Q.2008	29	30	23	27	0.4%	5.04 [0.53, 48.26]	2008
Lv,D.2009	28	30	23	30	0.7%	4.26 [0.81, 22.53]	2009
Wen,K.2009	32	38	32	38	2.3%	1.00 [0.29, 3.43]	2009
Zhang, L.2009	27	32	23	31	1.6%	1.88 [0.54, 6.54]	2009
He,W.2011	26	29	19	23	1.0%	1.82 [0.36, 9.12]	2011
Lu,Z.2011	30	34	28	34	1.5%	1.61 [0.41, 6.30]	2011
Jiang,S.2011	29	32	25	30	1.1%	1.93 [0.42, 8.91]	2011
Li, J.2014	19	27	16	27	2.1%	1.63 [0.53, 5.04]	2014
Ning, S.2015	26	31	22	31	1.6%	2.13 [0.62, 7.29]	2015
Li, J.-c.2016	31	35	26	35	1.3%	2.68 [0.74, 9.73]	2016
Zhang, X.2016	21	25	14	25	1.0%	4.13 [1.09, 15.59]	2016
Fang, L.2016	42	45	34	45	1.0%	4.53 [1.17, 17.55]	2016
Ma, M.2017	37	42	29	42	1.6%	3.32 [1.06, 10.37]	2017
Zhao,J.2019	39	43	34	43	1.4%	2.58 [0.73, 9.14]	2019
**Subtotal (95% CI)**		**541**		**523**	**20.9%**	**2.25 [1.61, 3.15]**	
Total events	478		404				
Heterogeneity: Chi² = 7.21, df = 14 (P = 0.93); I² = 0%							
Test for overall effect: Z = 4.72 (P < 0.00001)							
**Cisplatin (others)**							
Xu,Y.2012	30	33	26	33	1.1%	2.69 [0.63, 11.49]	2012
Han, R.2015	32	36	27	36	1.3%	2.67 [0.74, 9.63]	2015
**Subtotal (95% CI)**		**69**		**69**	**2.4%**	**2.68 [1.02, 7.01]**	
Total events	62		53				
Heterogeneity: Chi² = 0.00, df = 1 (P = 0.99); I² = 0%							
Test for overall effect: Z = 2.01 (P = 0.04)							
**Total (95% CI)**		**2406**		**2355**	**100.0%**	**2.29 [1.97, 2.67]**	
Total events	2095		1766				
Heterogeneity: Chi² = 44.59, df = 60 (P = 0.93); I² = 0%							
Test for overall effect: Z = 10.58 (P < 0.00001)							
Test for subgroup differences: Chi² = 0.31, df = 3 (P = 0.96), I² = 0%							

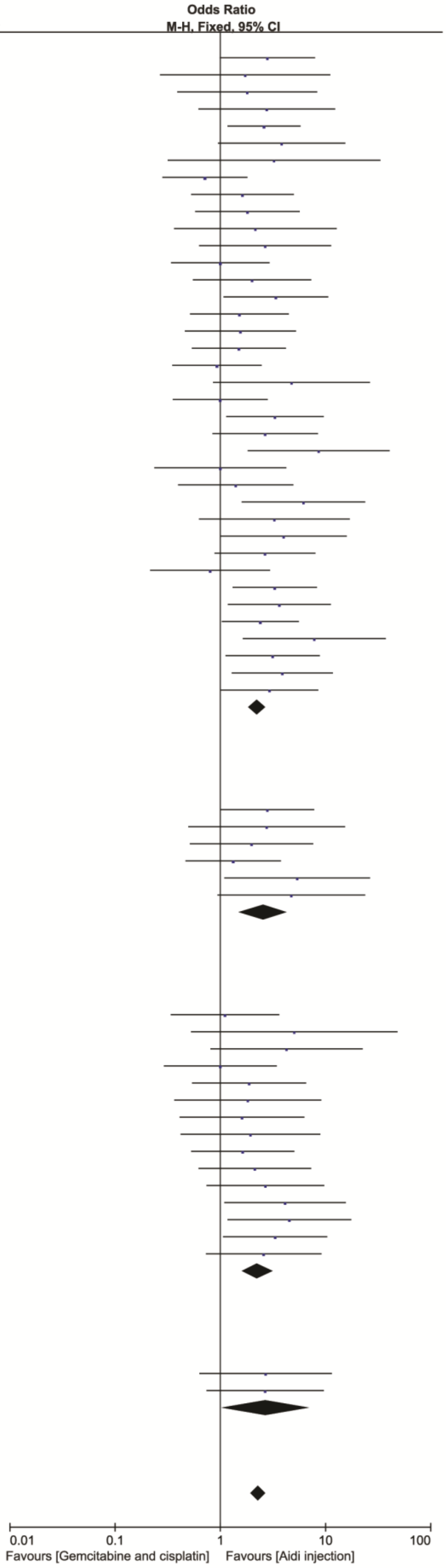

Figure S93 Subgroups analysis of disease control rate by cisplatin dosage

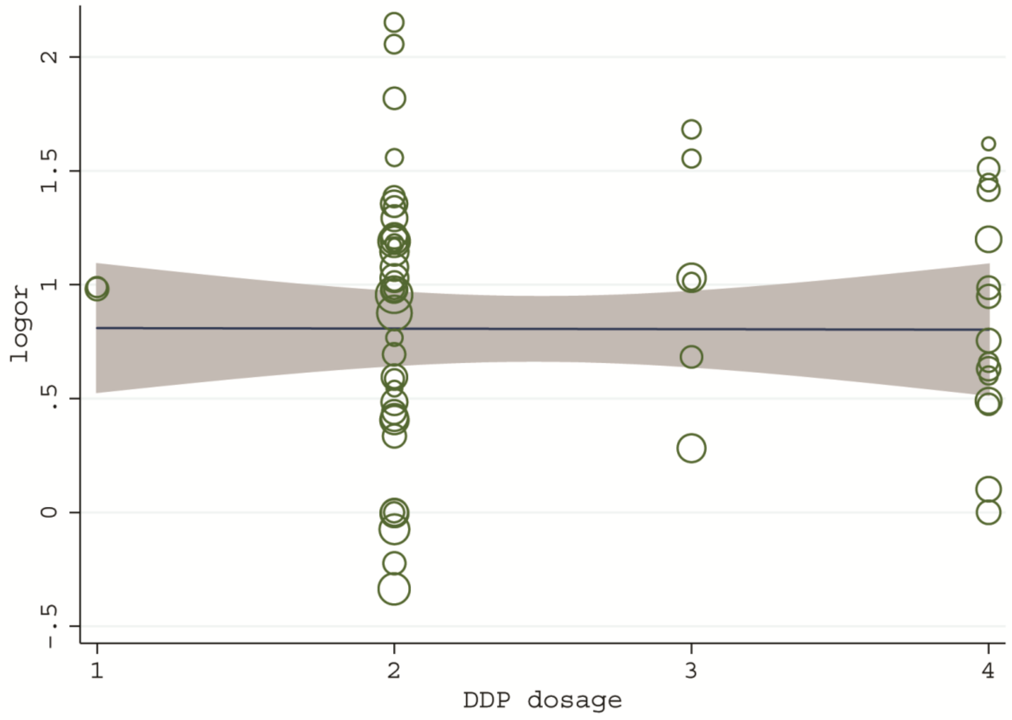

Figure S94 Meta-regression of disease control rate by cisplatin dosage

Study or Subgroup	Aidi injection Events	Aidi injection Total	Gemcitabine and cisplatin Events	Gemcitabine and cisplatin Total	Weight	Odds Ratio M-H, Fixed, 95% CI	Year
**Cisplatin (20-30mg/m^2)**							
Wang,Y.2009	26	32	22	27	1.0%	0.98 [0.26, 3.67]	2009
Hou,A.2010	14	40	22	38	3.4%	0.39 [0.16, 0.98]	2010
Hong,Y.2010	26	90	31	70	5.7%	0.51 [0.27, 0.98]	2010
Wang,J.2012	7	25	9	24	1.5%	0.65 [0.19, 2.16]	2012
Sun,J.2012	23	34	30	34	2.2%	0.28 [0.08, 0.99]	2012
Fu, L.2012	24	35	32	35	2.3%	0.20 [0.05, 0.81]	2012
Zhang,Y. 2012	12	41	17	42	2.7%	0.61 [0.24, 1.52]	2012
Xu, H.2013	13	38	22	42	3.2%	0.47 [0.19, 1.17]	2013
Cheng, B.2014	34	49	47	52	3.2%	0.24 [0.08, 0.73]	2014
Zhao, S.2015	7	43	10	43	1.9%	0.64 [0.22, 1.88]	2015
Zhang, M.2015	26	39	28	32	2.4%	0.29 [0.08, 0.99]	2015
Zhu, H.2015	6	21	15	21	2.5%	0.16 [0.04, 0.61]	2015
Zhang, S.2016	2	19	4	19	0.8%	0.44 [0.07, 2.76]	2016
Ma, Y. 2016	7	33	15	35	2.7%	0.36 [0.12, 1.05]	2016
Li, J.2016	12	47	19	47	3.3%	0.51 [0.21, 1.21]	2016
Zhang, H.2017	13	52	22	52	3.8%	0.45 [0.20, 1.05]	2017
Huang, W.2017	10	39	12	40	2.0%	0.80 [0.30, 2.16]	2017
Su, S.2017	2	40	2	39	0.4%	0.97 [0.13, 7.28]	2017
Wu, T.2017	10	67	21	68	4.1%	0.39 [0.17, 0.92]	2017
Zhou, D.2018	11	58	13	58	2.4%	0.81 [0.33, 1.99]	2018
Zhang, H.2018	13	30	22	30	2.9%	0.28 [0.09, 0.82]	2018
Chen,C.2020	33	49	43	49	3.2%	0.29 [0.10, 0.82]	2020
Guo, X.2020	7	51	16	51	3.2%	0.35 [0.13, 0.94]	2020
Tan, H.2020	51	60	57	60	2.0%	0.30 [0.08, 1.16]	2020
Xu,Z.2020	2	40	6	40	1.3%	0.30 [0.06, 1.58]	2020
**Subtotal (95% CI)**		**1072**		**1048**	**64.4%**	**0.43 [0.35, 0.53]**	
Total events	391		537				
Heterogeneity: Chi² = 14.74, df = 24 (P = 0.93); I² = 0%							
Test for overall effect: Z = 7.95 (P < 0.00001)							
**Cisplatin (40-50mg/m^2)**							
Wang,Y.2012	19	36	26	36	2.8%	0.43 [0.16, 1.15]	2012
Liu,Y.2014	27	43	36	43	3.1%	0.33 [0.12, 0.91]	2014
**Subtotal (95% CI)**		**79**		**79**	**5.9%**	**0.38 [0.19, 0.76]**	
Total events	46		62				
Heterogeneity: Chi² = 0.14, df = 1 (P = 0.71); I² = 0%							
Test for overall effect: Z = 2.71 (P = 0.007)							
**Cisplatin (60-80mg/m^2)**							
Feng,X.2008	64	68	60	62	0.9%	0.53 [0.09, 3.02]	2008
Wen,K.2009	23	38	31	38	2.8%	0.35 [0.12, 0.99]	2009
Zhang, L.2009	5	32	14	31	2.8%	0.22 [0.07, 0.74]	2009
Lu,Z.2011	11	34	20	34	3.1%	0.33 [0.12, 0.90]	2011
Li, J.2014	5	27	6	27	1.1%	0.80 [0.21, 3.00]	2014
Ning, S.2015	16	31	16	31	1.8%	1.00 [0.37, 2.71]	2015
Li, X.2015	2	20	8	20	1.7%	0.17 [0.03, 0.92]	2015
Li, J.-c.2016	14	35	26	35	3.6%	0.23 [0.08, 0.64]	2016
Fang, L.2016	5	45	13	45	2.7%	0.31 [0.10, 0.95]	2016
Ma, M.2017	11	42	16	42	2.7%	0.58 [0.23, 1.46]	2017
Wu, Y.2017	3	109	9	109	2.0%	0.31 [0.08, 1.19]	2017
**Subtotal (95% CI)**		**481**		**474**	**25.2%**	**0.39 [0.28, 0.55]**	
Total events	159		219				
Heterogeneity: Chi² = 8.54, df = 10 (P = 0.58); I² = 0%							
Test for overall effect: Z = 5.37 (P < 0.00001)							
**Cisplatin (others)**							
Xu,Y.2012	23	33	33	33	2.4%	0.03 [0.00, 0.60]	2012
Han, R.2015	7	36	11	36	2.0%	0.55 [0.18, 1.63]	2015
**Subtotal (95% CI)**		**69**		**69**	**4.4%**	**0.27 [0.11, 0.68]**	
Total events	30		44				
Heterogeneity: Chi² = 3.63, df = 1 (P = 0.06); I² = 72%							
Test for overall effect: Z = 2.78 (P = 0.005)							
**Total (95% CI)**		**1701**		**1670**	**100.0%**	**0.41 [0.35, 0.49]**	
Total events	626		862				
Heterogeneity: Chi² = 26.97, df = 39 (P = 0.93); I² = 0%							
Test for overall effect: Z = 10.32 (P < 0.00001)							
Test for subgroup differences: Chi² = 1.12, df = 3 (P = 0.77), I² = 0%							

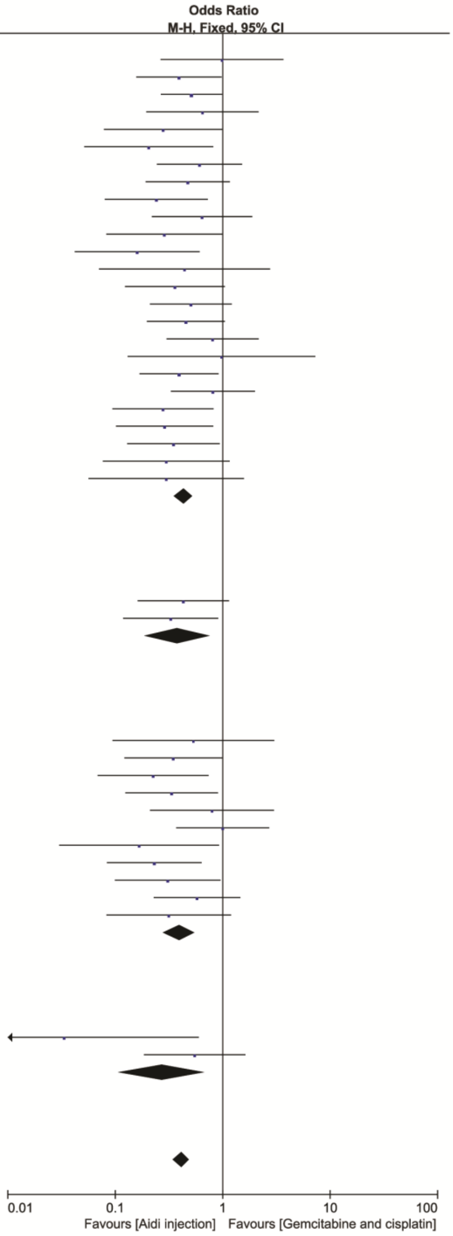

Figure S95 Subgroups analysis of neutropenia by cisplatin dosage

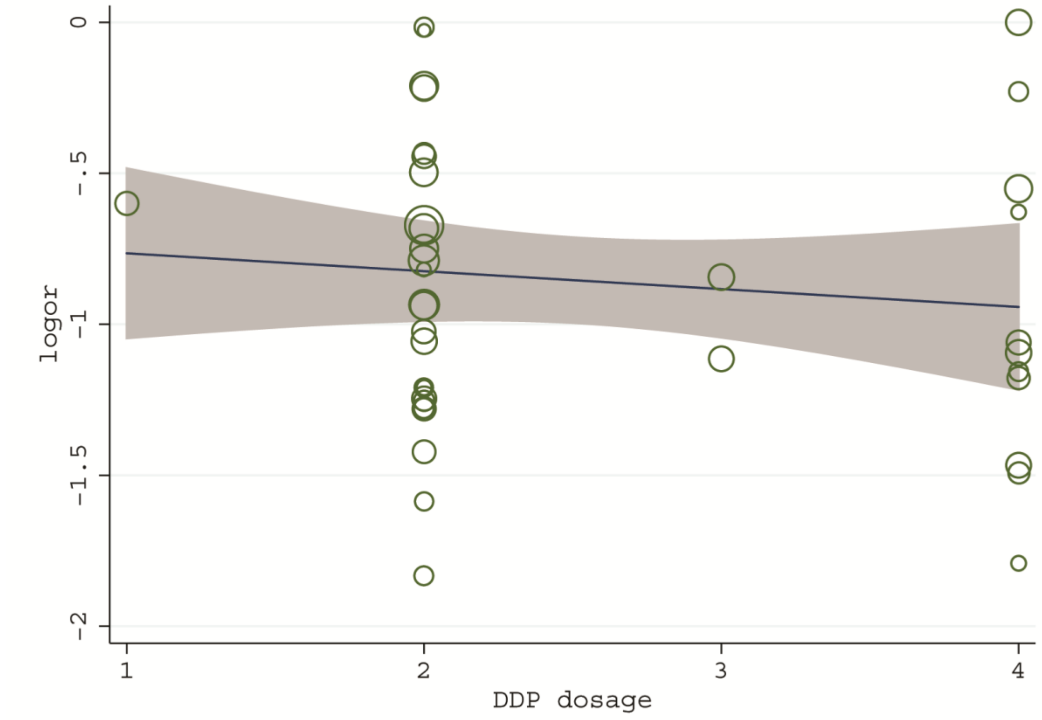

Figure S96 Meta-regression of neutropenia by cisplatin dosage

Study or Subgroup	Aidi injection Events	Aidi injection Total	Gemcitabine and cisplatin Events	Gemcitabine and cisplatin Total	Weight	Odds Ratio M-H, Fixed, 95% CI	Year
**Cisplatin (20-30mg/m^2)**							
Zou,Y.2006	32	42	29	39	1.2%	1.10 [0.40, 3.03]	2006
Wang,Y.2009	5	32	5	27	0.8%	0.81 [0.21, 3.18]	2009
Song,Z.2009	10	30	11	30	1.3%	0.86 [0.30, 2.50]	2009
Hou,A.2010	5	40	12	38	1.8%	0.31 [0.10, 0.99]	2010
Liu,Y.2010	7	32	14	32	1.9%	0.36 [0.12, 1.07]	2010
Hong,Y.2010	20	90	29	70	4.4%	0.40 [0.20, 0.80]	2010
Li,Z.2010	16	36	20	36	1.9%	0.64 [0.25, 1.62]	2010
Shi,X.2010	12	28	20	28	2.0%	0.30 [0.10, 0.91]	2010
Sun,J.2012	28	34	29	34	0.9%	0.80 [0.22, 2.94]	2012
Zhang,Y. 2012	9	41	14	42	1.9%	0.56 [0.21, 1.50]	2012
Wang,J.2012	5	25	8	24	1.1%	0.50 [0.14, 1.83]	2012
Cai,H.2013	6	19	8	19	0.9%	0.63 [0.17, 2.40]	2013
Xu, H.2013	20	38	30	42	2.3%	0.44 [0.18, 1.12]	2013
Lai,L.2013	20	70	28	70	3.4%	0.60 [0.30, 1.21]	2013
Cheng, B.2014	7	49	7	52	1.0%	1.07 [0.35, 3.31]	2014
Zhu, H.2015	7	21	16	21	1.8%	0.16 [0.04, 0.60]	2015
Zhao, S.2015	5	43	10	43	1.5%	0.43 [0.13, 1.40]	2015
Zhang, M.2015	32	39	27	32	0.9%	0.85 [0.24, 2.97]	2015
Ma, Y. 2016	5	33	13	35	1.8%	0.30 [0.09, 0.98]	2016
Li, J.2016	15	47	27	47	3.2%	0.35 [0.15, 0.81]	2016
Zhang, S.2016	2	19	3	19	0.5%	0.63 [0.09, 4.26]	2016
Zhang, H.2017	16	52	29	52	3.4%	0.35 [0.16, 0.79]	2017
Wu, T.2017	11	67	23	68	3.3%	0.38 [0.17, 0.87]	2017
Su, S.2017	4	40	3	39	0.5%	1.33 [0.28, 6.39]	2017
Zhang, H.2018	7	40	18	40	2.5%	0.26 [0.09, 0.72]	2018
Zhou, D.2018	29	58	31	58	2.7%	0.87 [0.42, 1.81]	2018
Chen,C.2020	40	49	42	49	1.3%	0.74 [0.25, 2.18]	2020
Geng,K.2020	21	45	30	45	2.7%	0.44 [0.19, 1.03]	2020
Guo, X.2020	5	51	13	51	2.0%	0.32 [0.10, 0.97]	2020
Tan, H.2020	47	60	54	60	2.0%	0.40 [0.14, 1.14]	2020
Xu,Z.2020	2	40	7	40	1.1%	0.25 [0.05, 1.28]	2020
**Subtotal (95% CI)**		**1310**		**1282**	**58.1%**	**0.49 [0.41, 0.59]**	
Total events	450		610				
Heterogeneity: Chi² = 22.67, df = 30 (P = 0.83); I² = 0%							
Test for overall effect: Z = 7.68 (P < 0.00001)							
**Cisplatin (40-50mg/m^2)**							
Wang,Y.2012	21	36	28	36	2.0%	0.40 [0.14, 1.12]	2012
Ju,S.2013	13	34	14	34	1.5%	0.88 [0.33, 2.34]	2013
Li, H.2014	18	30	22	30	1.5%	0.55 [0.18, 1.62]	2014
Liu,Y.2014	24	43	32	43	2.4%	0.43 [0.17, 1.08]	2014
Wen, H.2014	23	45	39	45	3.3%	0.16 [0.06, 0.45]	2014
Su,D.2019	5	41	15	41	2.3%	0.24 [0.08, 0.75]	2019
**Subtotal (95% CI)**		**229**		**229**	**12.9%**	**0.39 [0.26, 0.59]**	
Total events	104		150				
Heterogeneity: Chi² = 6.63, df = 5 (P = 0.25); I² = 25%							
Test for overall effect: Z = 4.52 (P < 0.00001)							
**Cisplatin (60-80mg/m^2)**							
Feng,X.2008	55	68	54	62	1.9%	0.63 [0.24, 1.63]	2008
Wen,K.2009	20	38	29	38	2.4%	0.34 [0.13, 0.92]	2009
Lv,D.2009	10	30	19	30	2.2%	0.29 [0.10, 0.84]	2009
Zhang, L.2009	11	32	20	31	2.3%	0.29 [0.10, 0.81]	2009
Lu,Z.2011	10	34	19	34	2.3%	0.33 [0.12, 0.90]	2011
He,W.2011	12	29	13	23	1.5%	0.54 [0.18, 1.64]	2011
Ning, S.2015	12	31	16	31	1.7%	0.59 [0.22, 1.62]	2015
Li, X.2015	15	20	15	20	0.6%	1.00 [0.24, 4.18]	2015
Fang, L.2016	6	45	14	45	2.1%	0.34 [0.12, 0.99]	2016
Li, J.-c.2016	7	35	19	35	2.6%	0.21 [0.07, 0.61]	2016
Wu, Y.2017	37	109	63	109	7.1%	0.38 [0.22, 0.65]	2017
**Subtotal (95% CI)**		**471**		**458**	**26.6%**	**0.39 [0.29, 0.52]**	
Total events	195		281				
Heterogeneity: Chi² = 5.78, df = 10 (P = 0.83); I² = 0%							
Test for overall effect: Z = 6.51 (P < 0.00001)							
**Cisplatin (others)**							
Xu,Y.2012	7	33	18	33	2.4%	0.22 [0.08, 0.66]	2012
**Subtotal (95% CI)**		**33**		**33**	**2.4%**	**0.22 [0.08, 0.66]**	
Total events	7		18				
Heterogeneity: Not applicable							
Test for overall effect: Z = 2.71 (P = 0.007)							
**Total (95% CI)**		**2043**		**2002**	**100.0%**	**0.45 [0.39, 0.51]**	
Total events	756		1059				
Heterogeneity: Chi² = 38.98, df = 48 (P = 0.82); I² = 0%							
Test for overall effect: Z = 11.21 (P < 0.00001)							
Test for subgroup differences: Chi² = 3.95, df = 3 (P = 0.27), I² = 24.0%							

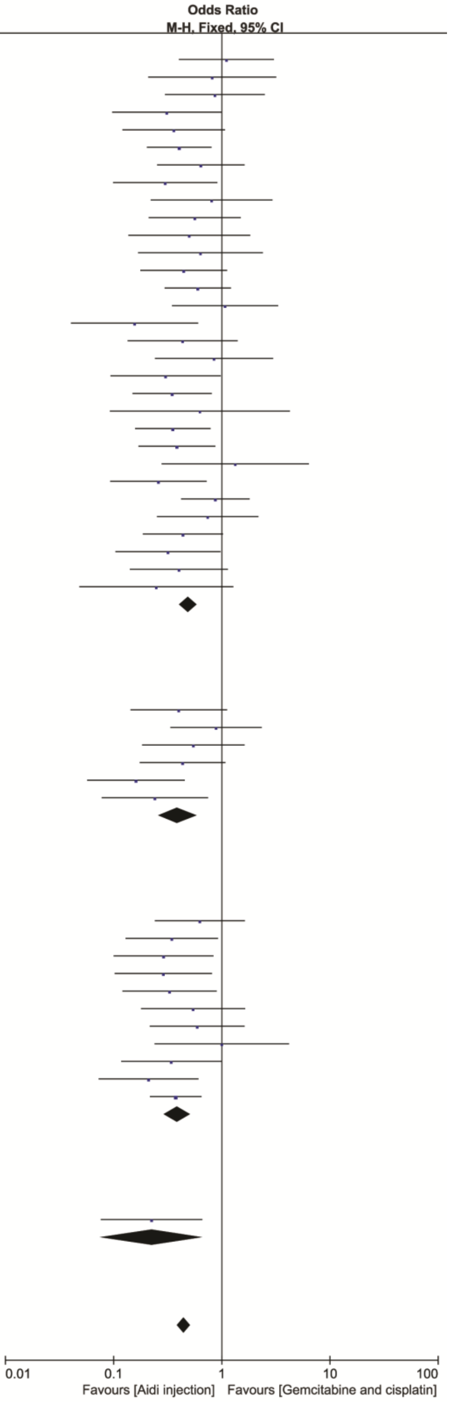

Figure S97 Subgroups analysis of gastrointestinal reaction by cisplatin dosage

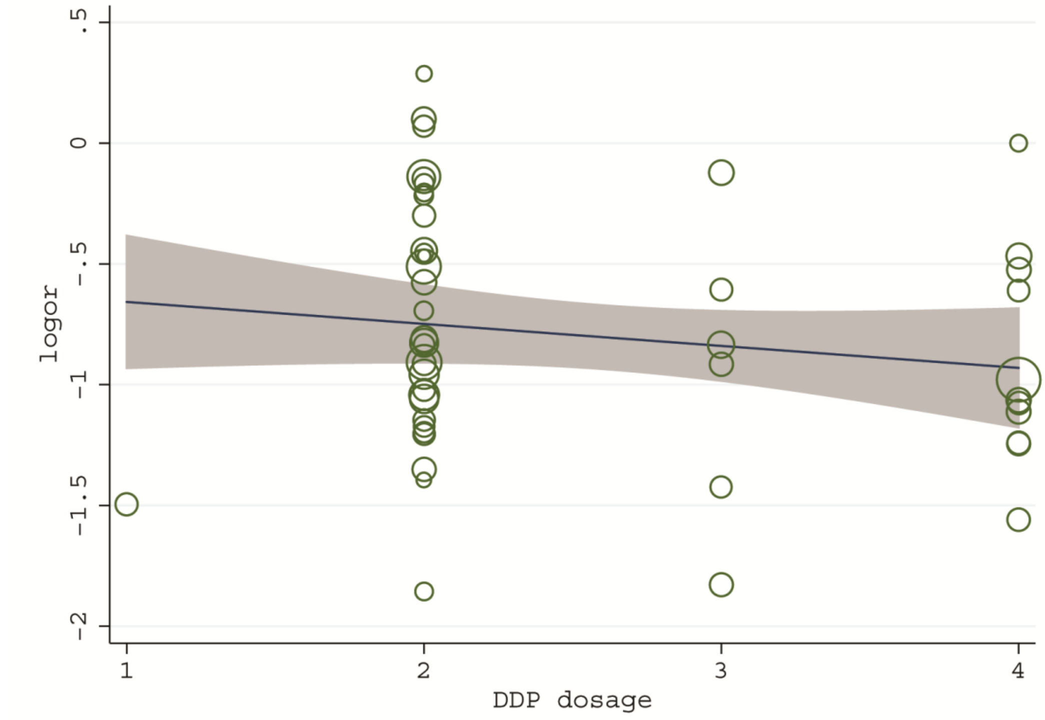

Figure S98 Meta-regression of gastrointestinal reaction by cisplatin dosage

Study or Subgroup	Aidi injection Events	Aidi injection Total	Gemcitabine and cisplatin Events	Gemcitabine and cisplatin Total	Weight	Odds Ratio M-H, Fixed, 95% CI	Year
**WHO**							
Zou,Y.2006	24	42	13	39	1.4%	2.67 [1.08, 6.58]	2006
Sun,G.2008	17	33	15	30	1.8%	1.06 [0.40, 2.86]	2008
Zhao,H.2008	11	30	6	20	1.1%	1.35 [0.40, 4.53]	2008
Yang,Q.2008	23	30	11	27	0.6%	4.78 [1.52, 14.98]	2008
Feng,X.2008	30	68	25	62	3.5%	1.17 [0.58, 2.35]	2008
Wang,Y.2009	17	32	12	27	1.4%	1.42 [0.51, 3.96]	2009
Song,Z.2009	10	30	9	30	1.4%	1.17 [0.39, 3.47]	2009
Wen,K.2009	16	38	15	38	2.1%	1.12 [0.45, 2.79]	2009
Lv,D.2009	22	30	11	30	0.7%	4.75 [1.58, 14.25]	2009
Zhang, L.2009	15	32	14	31	1.8%	1.07 [0.40, 2.89]	2009
Liu,Y.2010	16	32	10	32	1.2%	2.20 [0.79, 6.10]	2010
Li,Z.2010	16	36	12	36	1.6%	1.60 [0.62, 4.16]	2010
Shi,X.2010	13	28	10	28	1.3%	1.56 [0.53, 4.56]	2010
Hong,Y.2010	57	90	32	70	3.1%	2.05 [1.09, 3.88]	2010
Hou,A.2010	16	40	14	38	2.0%	1.14 [0.46, 2.85]	2010
Wu,Q.2011	17	30	11	30	1.1%	2.26 [0.80, 6.36]	2011
Fan,S.2011	22	41	12	38	1.4%	2.51 [1.00, 6.29]	2011
Jiang,S.2011	15	32	12	30	1.6%	1.32 [0.48, 3.63]	2011
Lu,Z.2011	18	34	17	34	1.9%	1.13 [0.43, 2.91]	2011
He,W.2011	15	29	10	23	1.3%	1.39 [0.46, 4.18]	2011
Zhang,Y. 2012	21	41	15	42	1.7%	1.89 [0.78, 4.55]	2012
Wang,J.2012	14	25	10	24	1.1%	1.78 [0.57, 5.53]	2012
Fu, L.2012	19	35	16	35	1.7%	1.41 [0.55, 3.61]	2012
Ju,S.2013	19	34	12	34	1.3%	2.32 [0.87, 6.16]	2013
Lai,L.2013	45	70	40	70	3.4%	1.35 [0.68, 2.67]	2013
Xu, H.2013	19	38	18	42	2.0%	1.33 [0.55, 3.22]	2013
Li, H.2014	16	30	12	30	1.3%	1.71 [0.62, 4.77]	2014
Liu,Y.2014	18	43	17	43	2.3%	1.10 [0.47, 2.60]	2014
Han, R.2015	14	36	10	36	1.4%	1.65 [0.61, 4.45]	2015
Zhu, H.2015	15	21	7	21	0.5%	5.00 [1.35, 18.55]	2015
Zhao, S.2015	19	43	12	43	1.6%	2.05 [0.83, 5.02]	2015
Ning, S.2015	19	31	11	31	1.0%	2.88 [1.03, 8.07]	2015
Zhang, S.2016	11	19	8	19	0.8%	1.89 [0.52, 6.85]	2016
Chen, Y.2016	15	30	13	30	1.5%	1.31 [0.47, 3.61]	2016
Ma, Y. 2016	19	33	19	35	1.9%	1.14 [0.44, 2.98]	2016
Li, J.2016	23	47	9	47	1.1%	4.05 [1.61, 10.20]	2016
Li, J.-c.2016	21	35	15	35	1.4%	2.00 [0.77, 5.18]	2016
Fang, L.2016	35	45	27	45	1.4%	2.33 [0.93, 5.87]	2016
Wu, T.2017	53	67	40	68	2.0%	2.65 [1.24, 5.68]	2017
Zhang, H.2017	42	52	26	52	1.2%	4.20 [1.75, 10.11]	2017
Ma, M.2017	19	42	12	42	1.6%	2.07 [0.84, 5.10]	2017
Lv, W.2018	16	30	15	30	1.7%	1.14 [0.41, 3.15]	2018
Zhao,J.2019	31	43	22	43	1.5%	2.47 [1.01, 6.04]	2019
Geng,K.2020	27	45	17	45	1.6%	2.47 [1.06, 5.77]	2020
Xu, Y.2020	16	45	12	45	1.8%	1.52 [0.62, 3.73]	2020
**Subtotal (95% CI)**		**1737**		**1680**	**70.9%**	**1.81 [1.58, 2.08]**	
Total events	956		686				
Heterogeneity: Chi² = 33.09, df = 44 (P = 0.89); I² = 0%							
Test for overall effect: Z = 8.43 (P < 0.00001)							
**RECIST**							
Xu,Y.2012	16	33	16	33	2.0%	1.00 [0.38, 2.63]	2012
Pei, W.2012	15	40	11	40	1.6%	1.58 [0.62, 4.07]	2012
Sun,J.2012	15	34	13	34	1.7%	1.28 [0.48, 3.36]	2012
Wang,Y.2012	16	36	14	36	1.8%	1.26 [0.49, 3.21]	2012
Cheng, B.2014	12	49	16	52	2.8%	0.73 [0.30, 1.76]	2014
Li, J.2014	11	27	10	27	1.4%	1.17 [0.39, 3.49]	2014
Wen, H.2014	28	45	17	45	1.5%	2.71 [1.16, 6.36]	2014
Zhang, M.2015	17	39	12	32	1.8%	1.29 [0.50, 3.35]	2015
Zhang, X.2016	18	25	8	25	0.5%	5.46 [1.63, 18.36]	2016
Huang, W.2017	17	39	11	40	1.5%	2.04 [0.80, 5.21]	2017
Su, S.2017	35	40	23	39	0.7%	4.87 [1.57, 15.13]	2017
Zhou, D.2018	30	58	22	58	2.5%	1.75 [0.84, 3.67]	2018
Su,D.2019	30	41	17	41	1.1%	3.85 [1.52, 9.75]	2019
Xu,Z.2020	25	40	14	40	1.2%	3.10 [1.24, 7.71]	2020
Chen,C.2020	26	49	16	49	1.8%	2.33 [1.03, 5.29]	2020
Tan, H.2020	24	60	18	60	2.6%	1.56 [0.73, 3.31]	2020
**Subtotal (95% CI)**		**655**		**651**	**26.4%**	**1.85 [1.47, 2.31]**	
Total events	335		238				
Heterogeneity: Chi² = 19.25, df = 15 (P = 0.20); I² = 22%							
Test for overall effect: Z = 5.34 (P < 0.00001)							
**Other**							
Ding,P.2011	9	18	10	22	1.1%	1.20 [0.34, 4.18]	2011
Liu, H.2019	19	44	12	44	1.6%	2.03 [0.83, 4.95]	2019
**Subtotal (95% CI)**		**62**		**66**	**2.7%**	**1.70 [0.82, 3.50]**	
Total events	28		22				
Heterogeneity: Chi² = 0.45, df = 1 (P = 0.50); I² = 0%							
Test for overall effect: Z = 1.44 (P = 0.15)							
**Total (95% CI)**		**2454**		**2397**	**100.0%**	**1.82 [1.62, 2.04]**	
Total events	1319		946				
Heterogeneity: Chi² = 52.84, df = 62 (P = 0.79); I² = 0%							
Test for overall effect: Z = 10.08 (P < 0.00001)							
Test for subgroup differences: Chi² = 0.06, df = 2 (P = 0.97), I² = 0%							

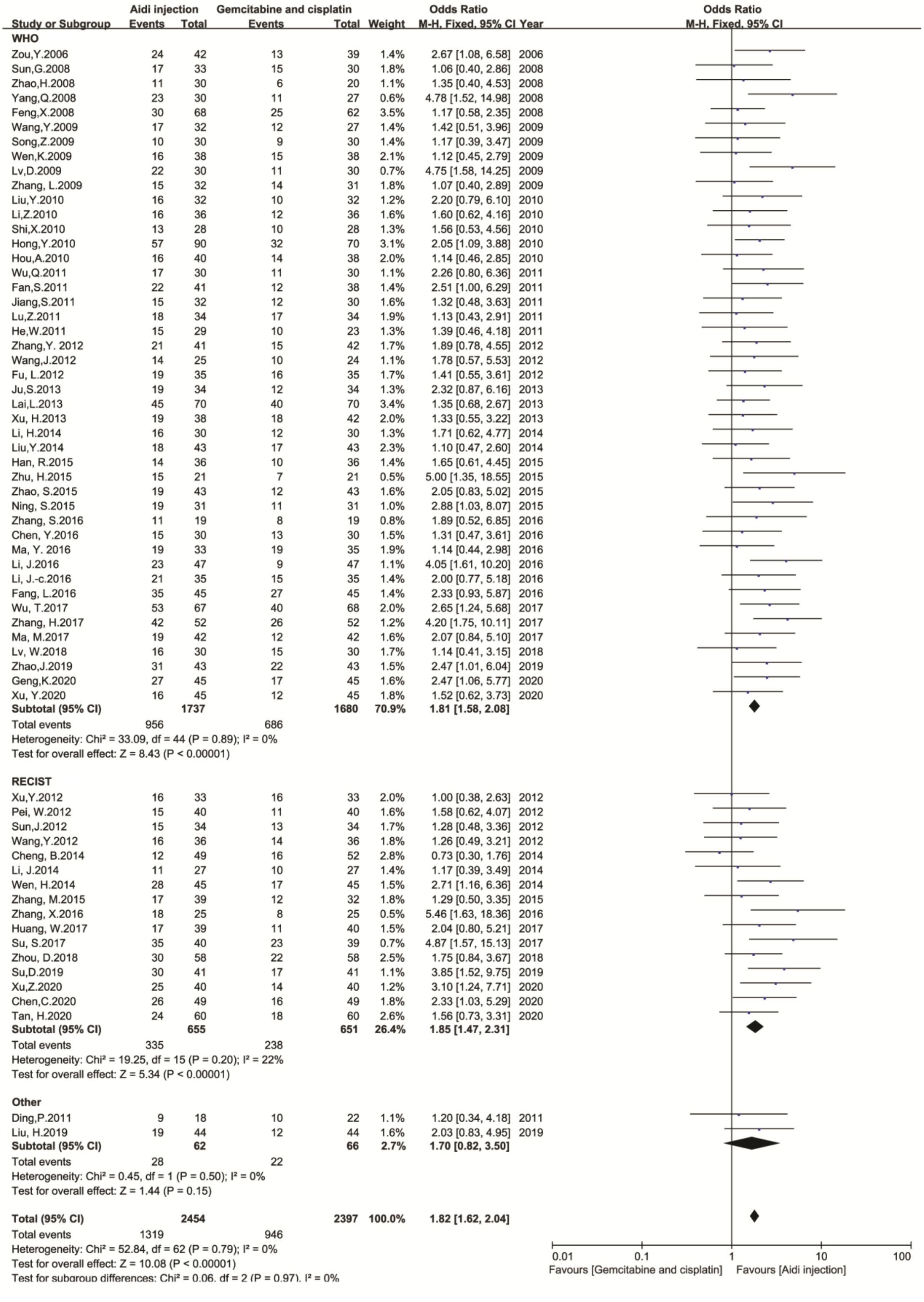

Figure S99 Subgroups analysis of objective response rate by evaluation criteria

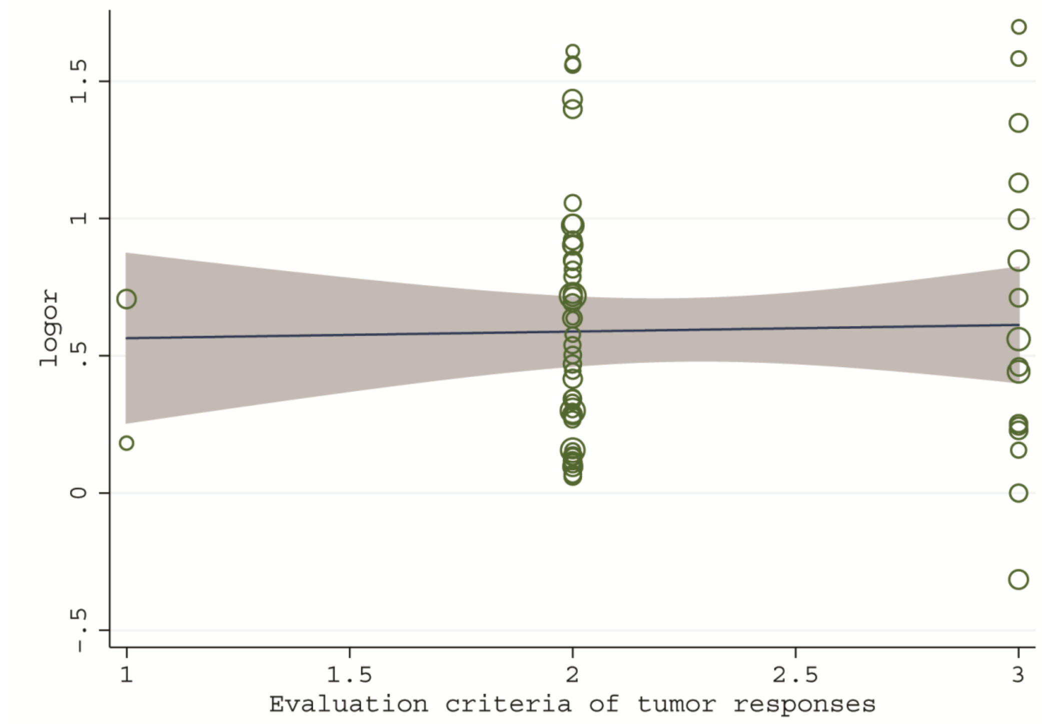

Figure S100 Meta-regression of objective response rate by evaluation criteria

Study or Subgroup	Aidi injection Events	Aidi injection Total	Gemcitabine and cisplatin Events	Gemcitabine and cisplatin Total	Weight	Odds Ratio M-H, Fixed, 95% CI	Year
**WHO**							
Zou,Y.2006	35	42	25	39	1.9%	2.80 [0.99, 7.94]	2006
Yang,Q.2008	29	30	23	27	0.4%	5.04 [0.53, 48.26]	2008
Sun,G.2008	31	33	27	30	0.8%	1.72 [0.27, 11.09]	2008
Feng,X.2008	62	68	56	62	2.3%	1.11 [0.34, 3.63]	2008
Lv,D.2009	28	30	23	30	0.7%	4.26 [0.81, 22.53]	2009
Wen,K.2009	32	38	32	38	2.3%	1.00 [0.29, 3.43]	2009
Zhang, L.2009	27	32	23	31	1.6%	1.88 [0.54, 6.54]	2009
Song,Z.2009	27	30	25	30	1.1%	1.80 [0.39, 8.32]	2009
Wang,Y.2009	29	32	21	27	1.0%	2.76 [0.62, 12.32]	2009
Li,Z.2010	18	36	21	36	4.7%	0.71 [0.28, 1.81]	2010
Liu,Y.2010	25	32	22	32	2.2%	1.62 [0.53, 4.99]	2010
Hong,Y.2010	78	90	50	70	3.4%	2.60 [1.17, 5.78]	2010
Hou,A.2010	37	40	29	38	1.0%	3.83 [0.95, 15.43]	2010
Shi,X.2010	27	28	25	28	0.4%	3.24 [0.32, 33.22]	2010
He,W.2011	26	29	19	23	1.0%	1.82 [0.36, 9.12]	2011
Lu,Z.2011	30	34	28	34	1.5%	1.61 [0.41, 6.30]	2011
Jiang,S.2011	29	32	25	30	1.1%	1.93 [0.42, 8.91]	2011
Fan,S.2011	35	41	29	38	2.0%	1.81 [0.58, 5.68]	2011
Wu,Q.2011	28	30	26	30	0.8%	2.15 [0.36, 12.76]	2011
Fu, L.2012	32	35	28	35	1.1%	2.67 [0.63, 11.31]	2012
Wang,J.2012	20	25	16	24	1.5%	2.00 [0.55, 7.31]	2012
Zhang,Y. 2012	34	41	32	42	2.4%	1.52 [0.52, 4.47]	2012
Xu, H.2013	33	38	34	42	1.9%	1.55 [0.46, 5.24]	2013
Lai,L.2013	63	70	60	70	2.7%	1.50 [0.54, 4.20]	2013
Ju,S.2013	32	34	29	34	0.8%	2.76 [0.50, 15.33]	2013
Li, H.2014	26	30	23	30	1.4%	1.98 [0.51, 7.63]	2014
Liu,Y.2014	35	43	33	43	2.8%	1.33 [0.47, 3.77]	2014
Ning, S.2015	26	31	22	31	1.6%	2.13 [0.62, 7.29]	2015
Zhu, H.2015	19	21	14	21	0.6%	4.75 [0.85, 26.43]	2015
Zhao, S.2015	37	43	28	43	1.8%	3.30 [1.14, 9.60]	2015
Han, R.2015	32	36	27	36	1.3%	2.67 [0.74, 9.63]	2015
Li, J.-c.2016	31	35	26	35	1.3%	2.68 [0.74, 9.73]	2016
Fang, L.2016	42	45	34	45	1.0%	4.53 [1.17, 17.55]	2016
Chen, Y.2016	24	30	18	30	1.6%	2.67 [0.84, 8.46]	2016
Li, J.2016	45	47	34	47	0.7%	8.60 [1.82, 40.69]	2016
Zhang, S.2016	14	19	14	19	1.7%	1.00 [0.24, 4.24]	2016
Ma, Y. 2016	28	33	28	35	1.9%	1.40 [0.40, 4.94]	2016
Ma, M.2017	37	42	29	42	1.6%	3.32 [1.06, 10.37]	2017
Zhang, H.2017	50	52	46	52	0.8%	3.26 [0.63, 16.97]	2017
Wu, T.2017	62	67	56	68	1.9%	2.66 [0.88, 8.02]	2017
Lv, W.2018	24	30	25	30	2.2%	0.80 [0.22, 2.97]	2018
Zhao,J.2019	39	43	34	43	1.4%	2.58 [0.73, 9.14]	2019
Xu, Y.2020	43	45	33	45	0.7%	7.82 [1.64, 37.36]	2020
Geng,K.2020	39	45	31	45	1.9%	2.94 [1.01, 8.53]	2020
**Subtotal (95% CI)**		**1707**		**1660**	**68.4%**	**2.17 [1.80, 2.62]**	
Total events	1500		1283				
Heterogeneity: Chi² = 27.21, df = 43 (P = 0.97); I² = 0%							
Test for overall effect: Z = 8.07 (P < 0.00001)							
**RECIST**							
Pei, W.2012	35	40	27	40	1.5%	3.37 [1.07, 10.61]	2012
Sun,J.2012	25	34	25	34	3.0%	1.00 [0.34, 2.94]	2012
Wang,Y.2012	28	36	20	36	2.0%	2.80 [1.01, 7.80]	2012
Xu,Y.2012	30	33	26	33	1.1%	2.69 [0.63, 11.49]	2012
Wen, H.2014	43	45	36	45	0.7%	5.38 [1.09, 26.49]	2014
Cheng, B.2014	39	49	42	52	3.7%	0.93 [0.35, 2.47]	2014
Li, J.2014	19	27	16	27	2.1%	1.63 [0.53, 5.04]	2014
Zhang, M.2015	28	39	23	32	3.2%	1.00 [0.35, 2.82]	2015
Zhang, X.2016	21	25	14	25	1.0%	4.13 [1.09, 15.59]	2016
Huang, W.2017	36	39	30	40	1.0%	4.00 [1.01, 15.87]	2017
Su, S.2017	37	40	26	39	0.9%	6.17 [1.60, 23.83]	2017
Zhou, D.2018	50	58	38	58	2.4%	3.29 [1.31, 8.27]	2018
Su,D.2019	39	41	33	41	0.7%	4.73 [0.94, 23.82]	2019
Chen,C.2020	44	49	34	49	1.6%	3.88 [1.28, 11.74]	2020
Tan, H.2020	49	60	39	60	3.2%	2.40 [1.03, 5.57]	2020
Xu,Z.2020	33	40	24	40	1.9%	3.14 [1.12, 8.82]	2020
**Subtotal (95% CI)**		**655**		**651**	**30.0%**	**2.50 [1.90, 3.30]**	
Total events	556		453				
Heterogeneity: Chi² = 15.92, df = 15 (P = 0.39); I² = 6%							
Test for overall effect: Z = 6.55 (P < 0.00001)							
**Other**							
Liu, H.2019	39	44	30	44	1.5%	3.64 [1.18, 11.23]	2019
**Subtotal (95% CI)**		**44**		**44**	**1.5%**	**3.64 [1.18, 11.23]**	
Total events	39		30				
Heterogeneity: Not applicable							
Test for overall effect: Z = 2.25 (P = 0.02)							
**Total (95% CI)**		**2406**		**2355**	**100.0%**	**2.29 [1.97, 2.67]**	
Total events	2095		1766				
Heterogeneity: Chi² = 44.59, df = 60 (P = 0.93); I² = 0%							
Test for overall effect: Z = 10.58 (P < 0.00001)							
Test for subgroup differences: Chi² = 1.38, df = 2 (P = 0.50), I² = 0%							

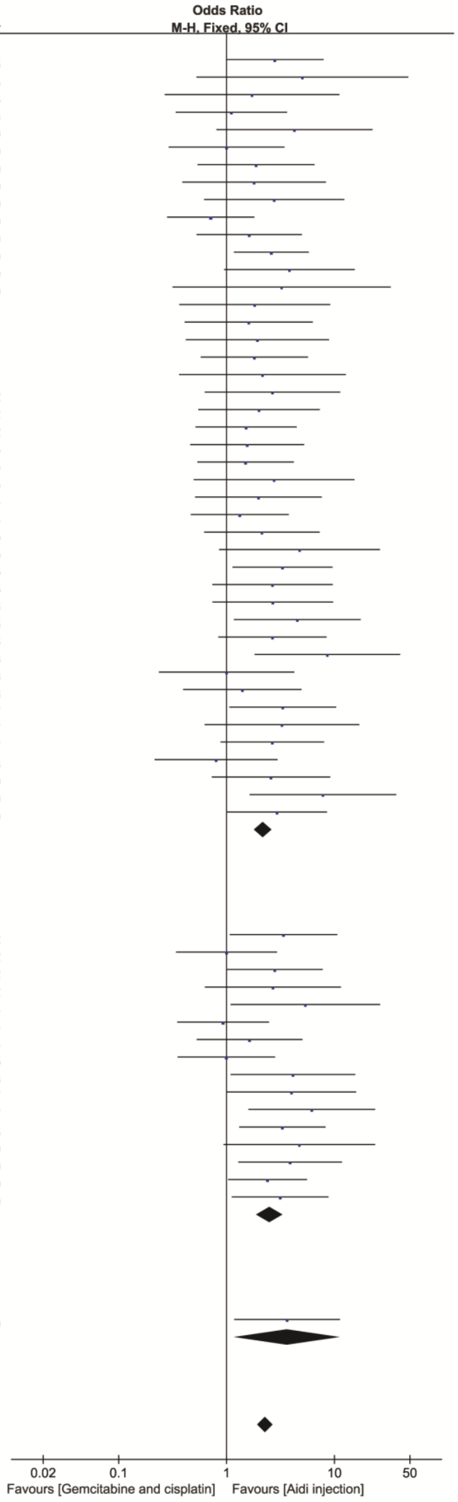

Figure S101 Subgroups analysis of disease control rate by evaluation criteria

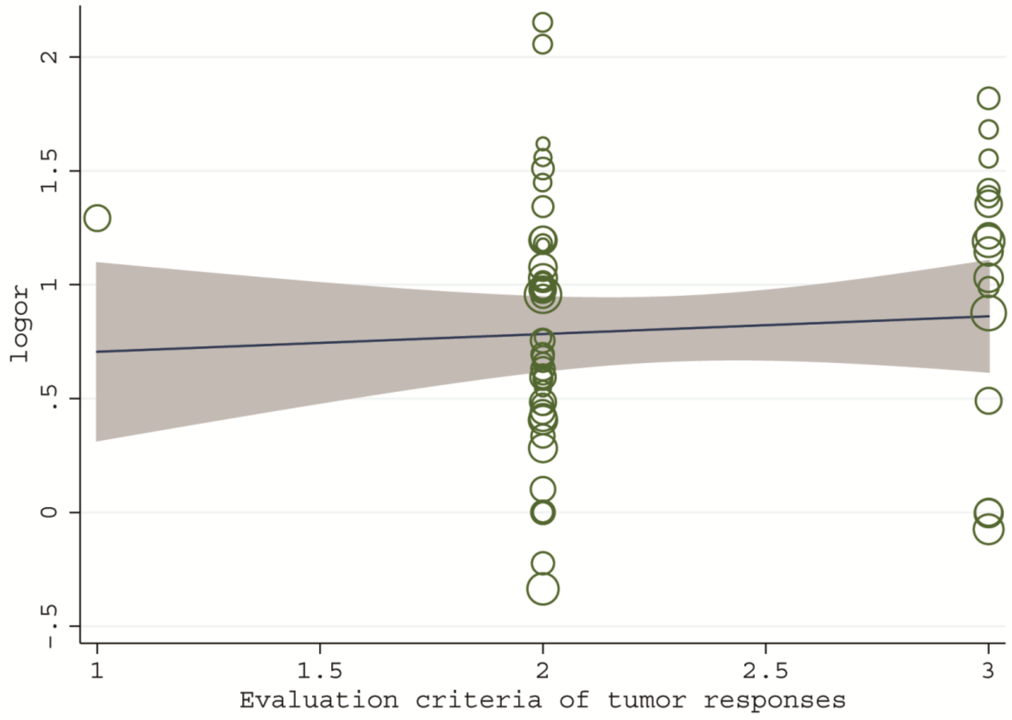

Figure S102 Meta-regression of disease control rate by evaluation criteria

Study or Subgroup	Aidi injection Events	Aidi injection Total	Gemcitabine and cisplatin Events	Gemcitabine and cisplatin Total	Weight	Odds Ratio M-H, Fixed, 95% CI	Year
**WHO**							
Feng,X.2008	64	68	60	62	0.9%	0.53 [0.09, 3.02]	2008
Wen,K.2009	23	38	31	38	2.8%	0.35 [0.12, 0.99]	2009
Zhang, L.2009	5	32	14	31	2.8%	0.22 [0.07, 0.74]	2009
Wang,Y.2009	26	32	22	27	1.0%	0.98 [0.26, 3.67]	2009
Hou,A.2010	14	40	22	38	3.4%	0.39 [0.16, 0.98]	2010
Hong,Y.2010	26	90	31	70	5.7%	0.51 [0.27, 0.98]	2010
Xu,Y.2012	23	33	33	33	2.4%	0.03 [0.00, 0.60]	2012
Wang,Y.2012	19	36	26	36	2.8%	0.43 [0.16, 1.15]	2012
Xu, H.2013	13	38	22	42	3.2%	0.47 [0.19, 1.17]	2013
Cheng, B.2014	34	49	47	52	3.2%	0.24 [0.08, 0.73]	2014
Liu,Y.2014	27	43	36	43	3.1%	0.33 [0.12, 0.91]	2014
Zhu, H.2015	6	21	15	21	2.5%	0.16 [0.04, 0.61]	2015
Ning, S.2015	16	31	16	31	1.8%	1.00 [0.37, 2.71]	2015
Zhao, S.2015	7	43	10	43	1.9%	0.64 [0.22, 1.88]	2015
Li, J.-c.2016	14	35	26	35	3.6%	0.23 [0.08, 0.64]	2016
Huang, W.2017	10	39	12	40	2.0%	0.80 [0.30, 2.16]	2017
Guo, X.2020	7	51	16	51	3.2%	0.35 [0.13, 0.94]	2020
Tan, H.2020	51	60	57	60	2.0%	0.30 [0.08, 1.16]	2020
**Subtotal (95% CI)**		**779**		**753**	**48.4%**	**0.40 [0.32, 0.51]**	
Total events	385		496				
Heterogeneity: Chi² = 16.42, df = 17 (P = 0.49); I² = 0%							
Test for overall effect: Z = 7.31 (P < 0.00001)							
**NCI-CTCAE**							
Sun,J.2012	23	34	30	34	2.2%	0.28 [0.08, 0.99]	2012
Li, J.2014	5	27	6	27	1.1%	0.80 [0.21, 3.00]	2014
Zhang, M.2015	26	39	28	32	2.4%	0.29 [0.08, 0.99]	2015
Fang, L.2016	5	45	13	45	2.7%	0.31 [0.10, 0.95]	2016
**Subtotal (95% CI)**		**145**		**138**	**8.4%**	**0.36 [0.20, 0.66]**	
Total events	59		77				
Heterogeneity: Chi² = 1.73, df = 3 (P = 0.63); I² = 0%							
Test for overall effect: Z = 3.29 (P = 0.0010)							
**Other**							
Lu,Z.2011	11	34	20	34	3.1%	0.33 [0.12, 0.90]	2011
Fu, L.2012	24	35	32	35	2.3%	0.20 [0.05, 0.81]	2012
Zhang,Y. 2012	12	41	17	42	2.7%	0.61 [0.24, 1.52]	2012
Wang,J.2012	7	25	9	24	1.5%	0.65 [0.19, 2.16]	2012
Han, R.2015	7	36	11	36	2.0%	0.55 [0.18, 1.63]	2015
Li, X.2015	2	20	8	20	1.7%	0.17 [0.03, 0.92]	2015
Zhang, S.2016	2	19	4	19	0.8%	0.44 [0.07, 2.76]	2016
Li, J.2016	12	47	19	47	3.3%	0.51 [0.21, 1.21]	2016
Ma, Y. 2016	7	33	15	35	2.7%	0.36 [0.12, 1.05]	2016
Wu, T.2017	10	67	21	68	4.1%	0.39 [0.17, 0.92]	2017
Wu, Y.2017	3	109	9	109	2.0%	0.31 [0.08, 1.19]	2017
Zhang, H.2017	13	52	22	52	3.8%	0.45 [0.20, 1.05]	2017
Ma, M.2017	11	42	16	42	2.7%	0.58 [0.23, 1.46]	2017
Su, S.2017	2	40	2	39	0.4%	0.97 [0.13, 7.28]	2017
Zhang, H.2018	13	30	22	30	2.9%	0.28 [0.09, 0.82]	2018
Zhou, D.2018	11	58	13	58	2.4%	0.81 [0.33, 1.99]	2018
Xu,Z.2020	2	40	6	40	1.3%	0.30 [0.06, 1.58]	2020
Chen,C.2020	33	49	43	49	3.2%	0.29 [0.10, 0.82]	2020
**Subtotal (95% CI)**		**777**		**779**	**43.2%**	**0.43 [0.34, 0.56]**	
Total events	182		289				
Heterogeneity: Chi² = 8.53, df = 17 (P = 0.95); I² = 0%							
Test for overall effect: Z = 6.51 (P < 0.00001)							
**Total (95% CI)**		**1701**		**1670**	**100.0%**	**0.41 [0.35, 0.49]**	
Total events	626		862				
Heterogeneity: Chi² = 26.97, df = 39 (P = 0.93); I² = 0%							
Test for overall effect: Z = 10.32 (P < 0.00001)							
Test for subgroup differences: Chi² = 0.35, df = 2 (P = 0.84), I² = 0%							

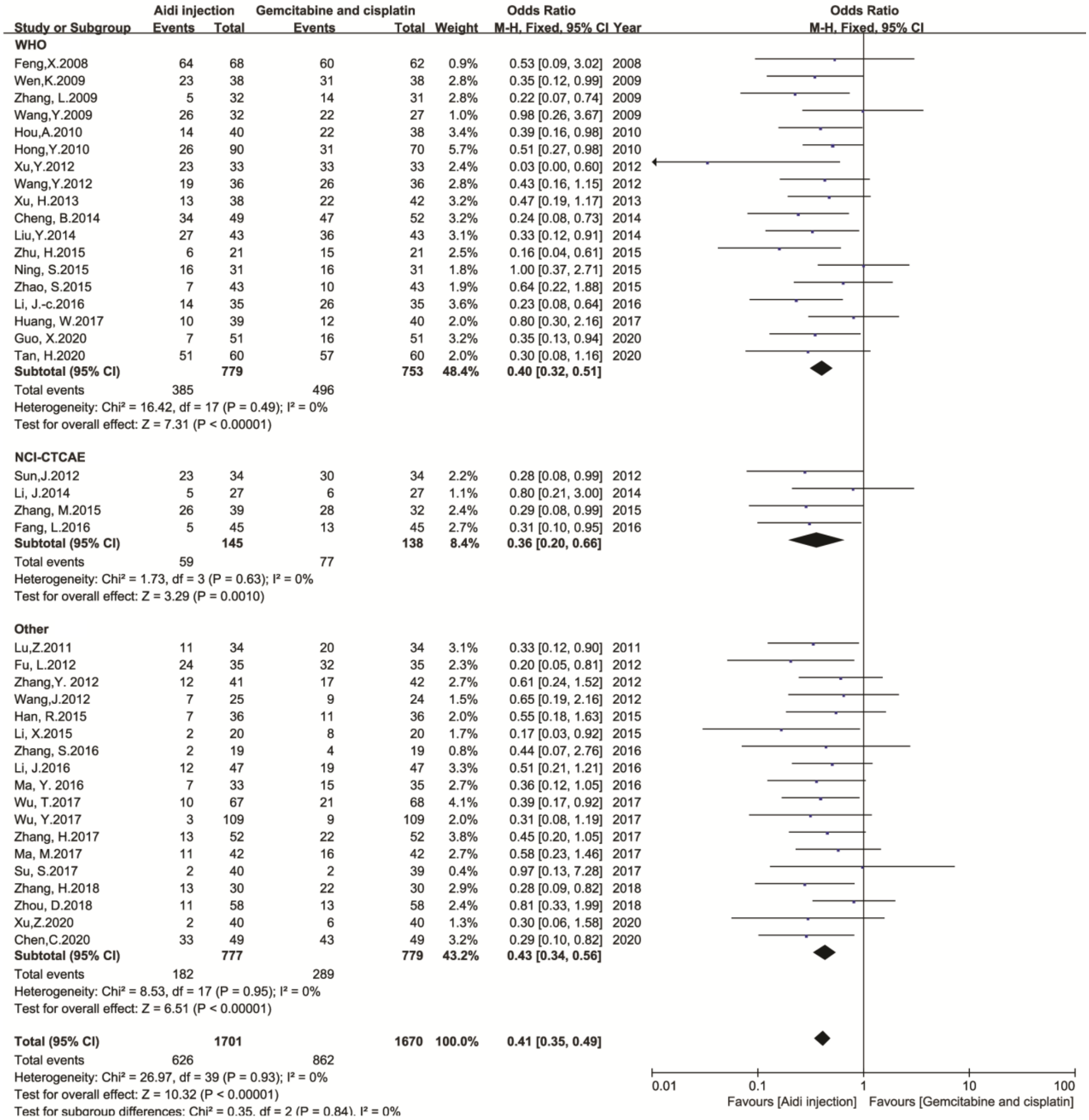

Figure S103 Subgroups analysis of neutropenia by evaluation criteria

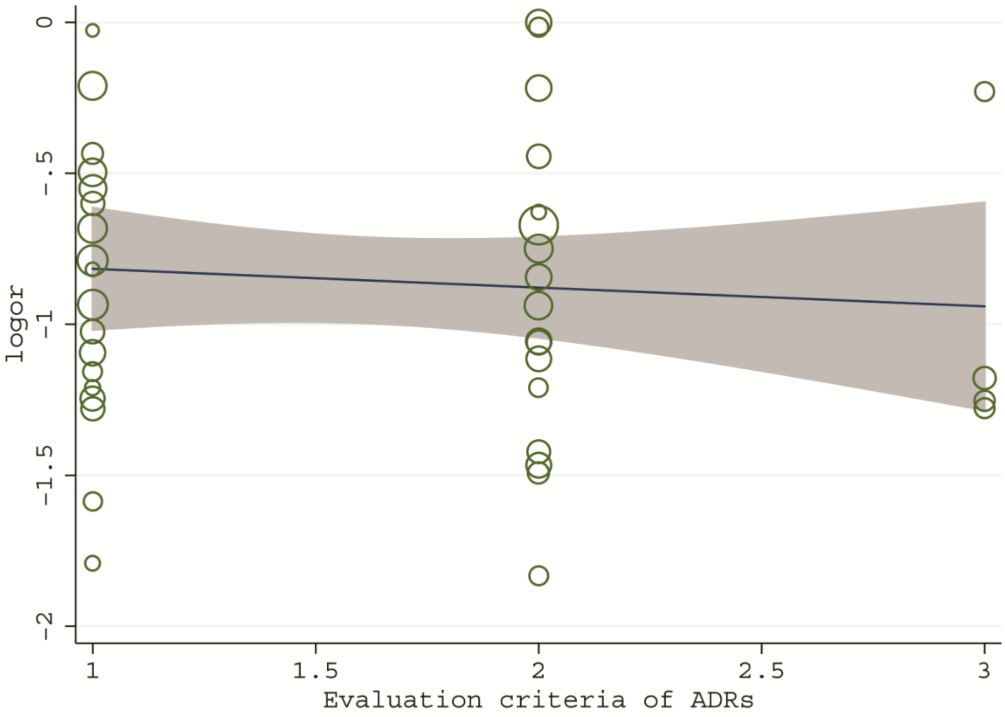

Figure S104 Meta-regression of neutropenia by evaluation criteria

Study or Subgroup	Aidi injection Events	Aidi injection Total	Gemcitabine and cisplatin Events	Gemcitabine and cisplatin Total	Weight	Odds Ratio M-H, Fixed, 95% CI	Year
**WHO**							
Zou,Y.2006	32	42	29	39	1.2%	1.10 [0.40, 3.03]	2006
Feng,X.2008	55	68	54	62	1.9%	0.63 [0.24, 1.63]	2008
Wen,K.2009	20	38	29	38	2.4%	0.34 [0.13, 0.92]	2009
Lv,D.2009	10	30	19	30	2.2%	0.29 [0.10, 0.84]	2009
Zhang, L.2009	11	32	20	31	2.3%	0.29 [0.10, 0.81]	2009
Wang,Y.2009	5	32	5	27	0.8%	0.81 [0.21, 3.18]	2009
Song,Z.2009	10	30	11	30	1.3%	0.86 [0.30, 2.50]	2009
Hou,A.2010	5	40	12	38	1.8%	0.31 [0.10, 0.99]	2010
Hong,Y.2010	20	90	29	70	4.4%	0.40 [0.20, 0.80]	2010
Li,Z.2010	16	36	20	36	1.9%	0.64 [0.25, 1.62]	2010
Shi,X.2010	12	28	20	28	2.0%	0.30 [0.10, 0.91]	2010
He,W.2011	12	29	13	23	1.5%	0.54 [0.18, 1.64]	2011
Wang,Y.2012	21	36	28	36	2.0%	0.40 [0.14, 1.12]	2012
Xu,Y.2012	7	33	18	33	2.4%	0.22 [0.08, 0.66]	2012
Xu, H.2013	20	38	30	42	2.3%	0.44 [0.18, 1.12]	2013
Lai,L.2013	20	70	28	70	3.4%	0.60 [0.30, 1.21]	2013
Cheng, B.2014	7	49	7	52	1.0%	1.07 [0.35, 3.31]	2014
Liu,Y.2014	24	43	32	43	2.4%	0.43 [0.17, 1.08]	2014
Ning, S.2015	12	31	16	31	1.7%	0.59 [0.22, 1.62]	2015
Zhu, H.2015	7	21	16	21	1.8%	0.16 [0.04, 0.60]	2015
Zhao, S.2015	5	43	10	43	1.5%	0.43 [0.13, 1.40]	2015
Li, J.-c.2016	7	35	19	35	2.6%	0.21 [0.07, 0.61]	2016
Guo, X.2020	5	51	13	51	2.0%	0.32 [0.10, 0.97]	2020
Tan, H.2020	47	60	54	60	2.0%	0.40 [0.14, 1.14]	2020
**Subtotal (95% CI)**		**1005**		**969**	**48.7%**	**0.45 [0.36, 0.55]**	
Total events	390		532				
Heterogeneity: Chi² = 18.57, df = 23 (P = 0.73); I² = 0%							
Test for overall effect: Z = 7.81 (P < 0.00001)							
**NCI-CTCAE**							
Sun,J.2012	28	34	29	34	0.9%	0.80 [0.22, 2.94]	2012
Wen, H.2014	23	45	39	45	3.3%	0.16 [0.06, 0.45]	2014
Zhang, M.2015	32	39	27	32	0.9%	0.85 [0.24, 2.97]	2015
Fang, L.2016	6	45	14	45	2.1%	0.34 [0.12, 0.99]	2016
**Subtotal (95% CI)**		**163**		**156**	**7.1%**	**0.38 [0.22, 0.66]**	
Total events	89		109				
Heterogeneity: Chi² = 5.52, df = 3 (P = 0.14); I² = 46%							
Test for overall effect: Z = 3.40 (P = 0.0007)							
**Other**							
Liu,Y.2010	7	32	14	32	1.9%	0.36 [0.12, 1.07]	2010
Lu,Z.2011	10	34	19	34	2.3%	0.33 [0.12, 0.90]	2011
Zhang,Y. 2012	9	41	14	42	1.9%	0.56 [0.21, 1.50]	2012
Wang,J.2012	5	25	8	24	1.1%	0.50 [0.14, 1.83]	2012
Cai,H.2013	6	19	8	19	0.9%	0.63 [0.17, 2.40]	2013
Ju,S.2013	13	34	14	34	1.5%	0.88 [0.33, 2.34]	2013
Li, H.2014	18	30	22	30	1.5%	0.55 [0.18, 1.62]	2014
Li, X.2015	15	20	15	20	0.6%	1.00 [0.24, 4.18]	2015
Zhang, S.2016	2	19	3	19	0.5%	0.63 [0.09, 4.26]	2016
Li, J.2016	15	47	27	47	3.2%	0.35 [0.15, 0.81]	2016
Ma, Y. 2016	5	33	13	35	1.8%	0.30 [0.09, 0.98]	2016
Zhang, H.2017	16	52	29	52	3.4%	0.35 [0.16, 0.79]	2017
Su, S.2017	4	40	3	39	0.5%	1.33 [0.28, 6.39]	2017
Wu, T.2017	11	67	23	68	3.3%	0.38 [0.17, 0.87]	2017
Wu, Y.2017	37	109	63	109	7.1%	0.38 [0.22, 0.65]	2017
Zhang, H.2018	7	40	18	40	2.5%	0.26 [0.09, 0.72]	2018
Zhou, D.2018	29	58	31	58	2.7%	0.87 [0.42, 1.81]	2018
Su,D.2019	5	41	15	41	2.3%	0.24 [0.08, 0.75]	2019
Chen,C.2020	40	49	42	49	1.3%	0.74 [0.25, 2.18]	2020
Geng,K.2020	21	45	30	45	2.7%	0.44 [0.19, 1.03]	2020
Xu,Z.2020	2	40	7	40	1.1%	0.25 [0.05, 1.28]	2020
**Subtotal (95% CI)**		**875**		**877**	**44.2%**	**0.46 [0.37, 0.56]**	
Total events	277		418				
Heterogeneity: Chi² = 14.63, df = 20 (P = 0.80); I² = 0%							
Test for overall effect: Z = 7.30 (P < 0.00001)							
**Total (95% CI)**		**2043**		**2002**	**100.0%**	**0.45 [0.39, 0.51]**	
Total events	756		1059				
Heterogeneity: Chi² = 38.98, df = 48 (P = 0.82); I² = 0%							
Test for overall effect: Z = 11.21 (P < 0.00001)							
Test for subgroup differences: Chi² = 0.35, df = 2 (P = 0.84), I² = 0%							

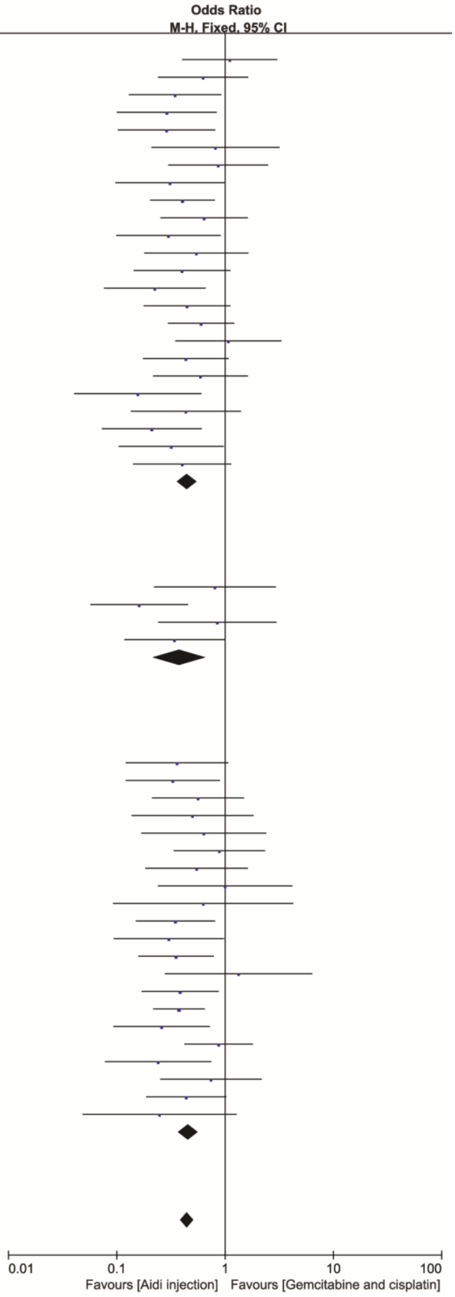

Figure S105 Subgroups analysis of gastrointestinal reaction by evaluation criteria

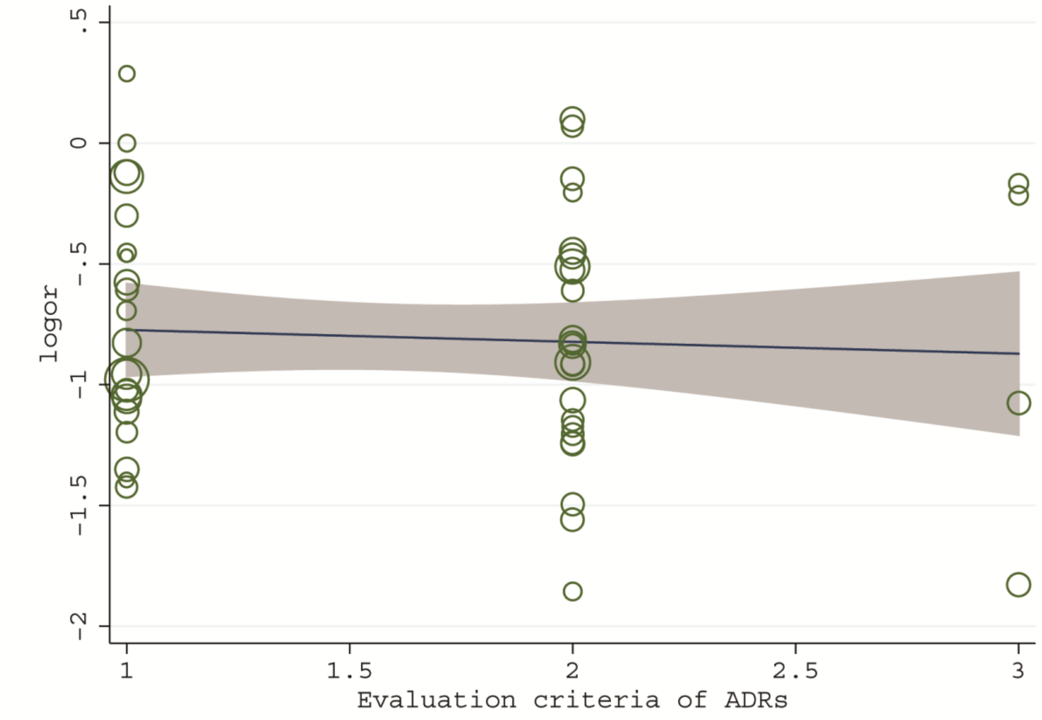

Figure S106 Meta-regression of gastrointestinal reaction by evaluation criteria
