# Supplementary material for: The Optimal Adjuvant Strategy of Aidi Injection With Gemcitabine and Cisplatin in Advanced Non–small Cell Lung Cancer: A Meta-analysis of 70 Randomized Controlled Trials
Source: Front Pharmacol. 2021 May 28;12:582447. doi: 10.3389/fphar.2021.582447 (PMC8194277; doi:10.3389/fphar.2021.582447)
Supplement: Supplementary file 3 [file DataSheet1.pdf]

Supplementary material 4. Subgroup analysis results of peripheral blood lymphocytes levels (Figures S1-24)

Study or Subgroup	Aidi injection Mean	SD	Total	Gemcitabine and cisplatin Mean	SD	Total	Weight	Std. Mean Difference IV, Random, 95% CI	Year
**KPS score (≥60)**									
Tan, H.2020	55.64	7.85	60	50.15	8.41	60	6.9%	0.67 [0.30, 1.04]	
Jiang,S.2011	49.2	11.6	32	36.6	10.2	30	6.5%	1.14 [0.60, 1.68]	2011
Li, X.2015	59.19	4.9	20	57.61	4.84	20	6.3%	0.32 [-0.31, 0.94]	2015
Li, J.2016	62.3	3.2	47	58.4	3	47	6.8%	1.25 [0.80, 1.69]	2016
Ma, M.2017	62.37	5.43	42	52.75	6.52	42	6.6%	1.59 [1.10, 2.08]	2017
Huang, W.2017	57.48	6.35	39	42.06	3.56	40	6.2%	2.98 [2.33, 3.63]	2017
**Subtotal (95% CI)**			**240**			**239**	**39.4%**	**1.31 [0.68, 1.94]**	
Heterogeneity: Tau² = 0.54; Chi² = 46.86, df = 5 (P < 0.00001); I² = 89%									
Test for overall effect: Z = 4.09 (P < 0.0001)									
**KPS score (≥70)**									
Zhao, S.2015	65.19	6.85	43	53.84	4.3	43	6.6%	1.97 [1.45, 2.49]	2015
Lv, W.2018	67	6	30	65	7	30	6.6%	0.30 [-0.21, 0.81]	2018
**Subtotal (95% CI)**			**73**			**73**	**13.2%**	**1.13 [-0.50, 2.76]**	
Heterogeneity: Tau² = 1.32; Chi² = 20.13, df = 1 (P < 0.00001); I² = 95%									
Test for overall effect: Z = 1.36 (P = 0.17)									
**KPS score (Others)**									
Zhao,J.2019	49.02	7.25	43	44.06	6.81	43	6.8%	0.70 [0.26, 1.13]	
Xu,Z.2020	60.75	4.51	40	54.69	5.37	40	6.7%	1.21 [0.73, 1.69]	
Zhang, H.2014	67.05	5.42	64	68.26	5.21	64	7.0%	-0.23 [-0.57, 0.12]	2014
Han, R.2015	58.29	2.57	36	49.85	3.79	36	6.3%	2.58 [1.95, 3.21]	2015
Wu, Y.2017	59.19	4.9	109	57.61	4.84	109	7.1%	0.32 [0.06, 0.59]	2017
Liu, H.2019	60.76	4.88	44	54.47	5.89	44	6.7%	1.15 [0.70, 1.61]	2019
Guo, X.2020	53.58	5.19	51	53.26	5.18	51	6.9%	0.06 [-0.33, 0.45]	2020
**Subtotal (95% CI)**			**387**			**387**	**47.5%**	**0.79 [0.23, 1.36]**	
Heterogeneity: Tau² = 0.53; Chi² = 81.88, df = 6 (P < 0.00001); I² = 93%									
Test for overall effect: Z = 2.77 (P = 0.006)									
**Total (95% CI)**			**700**			**699**	**100.0%**	**1.04 [0.63, 1.46]**	
Heterogeneity: Tau² = 0.60; Chi² = 179.53, df = 14 (P < 0.00001); I² = 92%									
Test for overall effect: Z = 4.96 (P < 0.00001)									
Test for subgroup differences: Chi² = 1.45, df = 2 (P = 0.49), I² = 0%									

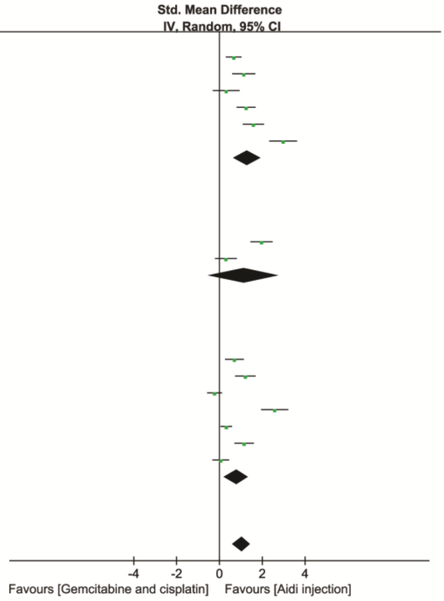

Figure S1 Subgroups analysis of $CD3^+$ T cells by KPS score

Study or Subgroup	Aidi injection Mean	SD	Total	Gemcitabine and cisplatin Mean	SD	Total	Weight	Std. Mean Difference IV, Random, 95% CI	Year
**KPS score (≥60)**									
Tan, H.2020	36.26	5.48	60	30.48	4.17	60	6.6%	1.18 [0.79, 1.57]	
Jiang,S.2011	37.6	5.4	32	26.3	6.5	30	5.9%	1.87 [1.27, 2.48]	2011
Li, X.2015	38.11	5.31	20	36.76	4.69	20	5.8%	0.26 [-0.36, 0.89]	2015
Li, J.2016	35.2	3.1	47	29.3	2.7	47	6.2%	2.01 [1.51, 2.51]	2016
Ma, M.2017	22.36	5.04	42	16.45	2.58	42	6.3%	1.46 [0.98, 1.95]	2017
Huang, W.2017	33.34	5.18	39	28.92	3.93	40	6.3%	0.95 [0.49, 1.42]	2017
**Subtotal (95% CI)**			**240**			**239**	**37.1%**	**1.30 [0.84, 1.75]**	
Heterogeneity: Tau² = 0.26; Chi² = 24.83, df = 5 (P = 0.0002); I² = 80%									
Test for overall effect: Z = 5.59 (P < 0.00001)									
**KPS score (≥70)**									
Zhao, S.2015	35.11	1.53	43	30.25	2.66	43	6.1%	2.22 [1.68, 2.76]	2015
Lv, W.2018	36	3	30	30	2	30	5.7%	2.32 [1.66, 2.99]	2018
**Subtotal (95% CI)**			**73**			**73**	**11.7%**	**2.26 [1.84, 2.68]**	
Heterogeneity: Tau² = 0.00; Chi² = 0.06, df = 1 (P = 0.81); I² = 0%									
Test for overall effect: Z = 10.55 (P < 0.00001)									
**KPS score (Others)**									
Xu,Z.2020	36.36	4.25	40	32.96	2.05	40	6.3%	1.01 [0.54, 1.48]	
Zhao,J.2019	39.91	5.08	43	34.82	5.47	43	6.4%	0.96 [0.51, 1.40]	
Zhang, H.2014	38.11	3.15	64	34.23	3.43	64	6.6%	1.17 [0.80, 1.55]	2014
Han, R.2015	34.16	5.25	36	23.28	5.36	36	6.0%	2.03 [1.45, 2.60]	2015
Wu, Y.2017	38.11	5.31	109	36.76	4.69	109	6.9%	0.27 [0.00, 0.54]	2017
Su, S.2017	42.9	4.89	40	33.32	5.16	39	6.1%	1.89 [1.35, 2.42]	2017
Liu, H.2019	36.01	4.13	44	33.38	2.55	44	6.5%	0.76 [0.33, 1.19]	2019
Guo, X.2020	47.26	4.28	51	40.17	2.35	51	6.3%	2.04 [1.56, 2.52]	2020
**Subtotal (95% CI)**			**427**			**426**	**51.1%**	**1.24 [0.76, 1.73]**	
Heterogeneity: Tau² = 0.43; Chi² = 71.69, df = 7 (P < 0.00001); I² = 90%									
Test for overall effect: Z = 5.06 (P < 0.00001)									
**Total (95% CI)**			**740**			**738**	**100.0%**	**1.38 [1.04, 1.72]**	
Heterogeneity: Tau² = 0.42; Chi² = 129.01, df = 15 (P < 0.00001); I² = 88%									
Test for overall effect: Z = 7.97 (P < 0.00001)									
Test for subgroup differences: Chi² = 13.17, df = 2 (P = 0.001), I² = 84.8%									

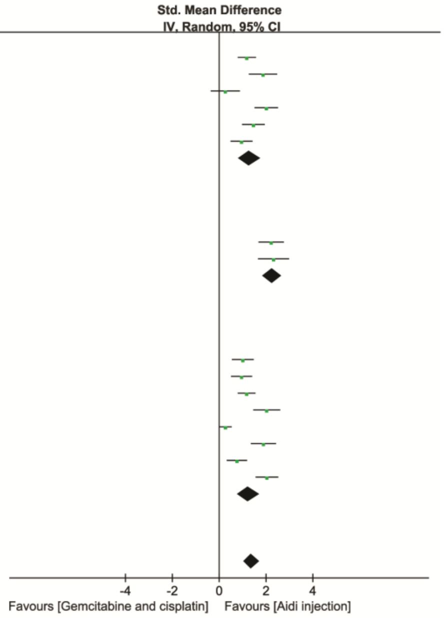

Figure S2 Subgroups analysis of $CD3^+ CD4^+$ T cells by KPS score

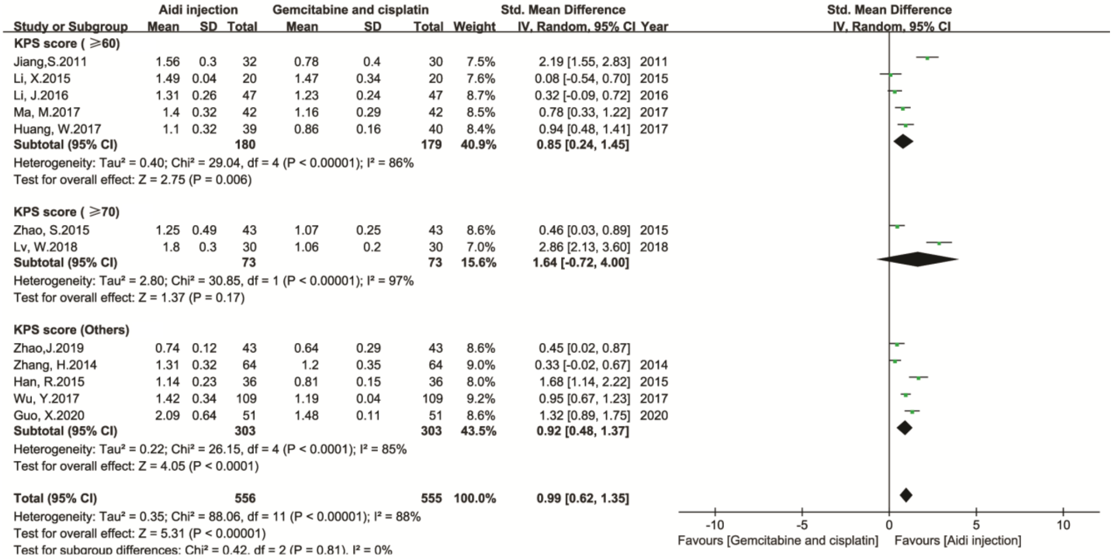

Figure S3 Subgroups analysis of $CD4^{+}/CD8^{+}$ T cells by KPS score

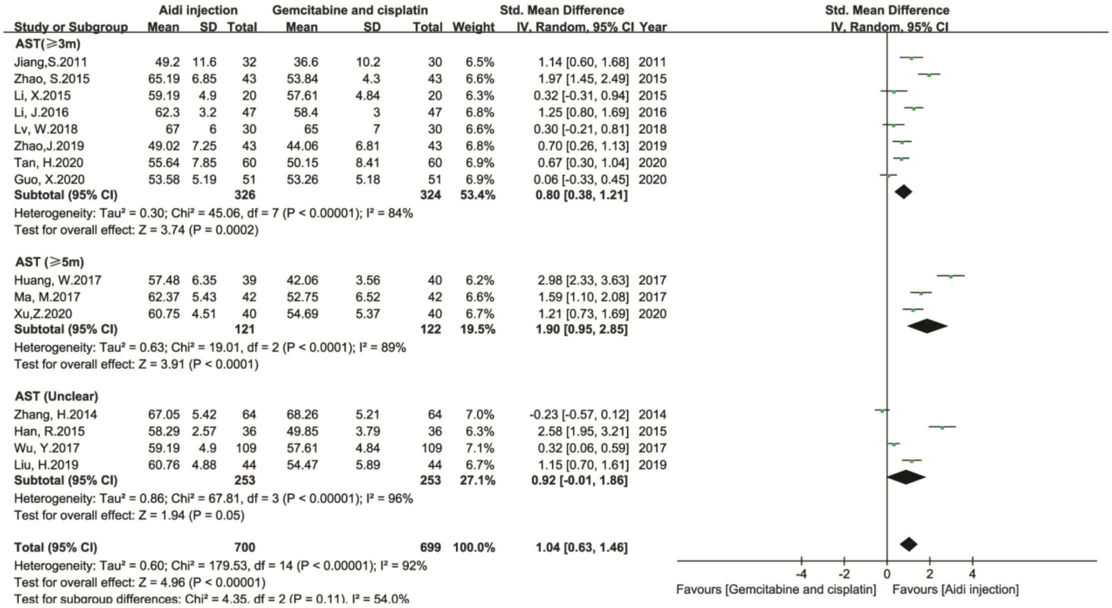

Figure S4 Subgroups analysis of $CD3^{+}$ T cells by anticipated survival time

Study or Subgroup	Aidi injection Mean	SD	Total	Gemcitabine and cisplatin Mean	SD	Total	Weight	Std. Mean Difference IV, Random, 95% CI	Year
**AST(≥3m)**									
Jiang,S.2011	37.6	5.4	32	26.3	6.5	30	5.9%	1.87 [1.27, 2.48]	2011
Zhao, S.2015	35.11	1.53	43	30.25	2.66	43	6.1%	2.22 [1.68, 2.76]	2015
Li, X.2015	38.11	5.31	20	36.76	4.69	20	5.8%	0.26 [-0.36, 0.89]	2015
Li, J.2016	35.2	3.1	47	29.3	2.7	47	6.2%	2.01 [1.51, 2.51]	2016
Su, S.2017	42.9	4.89	40	33.32	5.16	39	6.1%	1.89 [1.35, 2.42]	2017
Lv, W.2018	36	3	30	30	2	30	5.7%	2.32 [1.66, 2.99]	2018
Zhao,J.2019	39.91	5.08	43	34.82	5.47	43	6.4%	0.96 [0.51, 1.40]	2019
Tan, H.2020	36.26	5.48	60	30.48	4.17	60	6.6%	1.18 [0.79, 1.57]	2020
Guo, X.2020	47.26	4.28	51	40.17	2.35	51	6.3%	2.04 [1.56, 2.52]	2020
**Subtotal (95% CI)**			**366**			**363**	**55.1%**	**1.63 [1.21, 2.05]**	
Heterogeneity: Tau² = 0.34; Chi² = 47.40, df = 8 (P < 0.00001); I² = 83%									
Test for overall effect: Z = 7.62 (P < 0.00001)									
**AST (≥5m)**									
Ma, M.2017	22.36	5.04	42	16.45	2.58	42	6.3%	1.46 [0.98, 1.95]	2017
Huang, W.2017	33.34	5.18	39	28.92	3.93	40	6.3%	0.95 [0.49, 1.42]	2017
Xu,Z.2020	36.36	4.25	40	32.96	2.05	40	6.3%	1.01 [0.54, 1.48]	2020
**Subtotal (95% CI)**			**121**			**122**	**19.0%**	**1.14 [0.82, 1.45]**	
Heterogeneity: Tau² = 0.02; Chi² = 2.62, df = 2 (P = 0.27); I² = 24%									
Test for overall effect: Z = 7.13 (P < 0.00001)									
**AST (Unclear)**									
Zhang, H.2014	38.11	3.15	64	34.23	3.43	64	6.6%	1.17 [0.80, 1.55]	2014
Han, R.2015	34.16	5.25	36	23.28	5.36	36	6.0%	2.03 [1.45, 2.60]	2015
Wu, Y.2017	38.11	5.31	109	36.76	4.69	109	6.9%	0.27 [0.00, 0.54]	2017
Liu, H.2019	36.01	4.13	44	33.38	2.55	44	6.5%	0.76 [0.33, 1.19]	2019
**Subtotal (95% CI)**			**253**			**253**	**26.0%**	**1.03 [0.35, 1.71]**	
Heterogeneity: Tau² = 0.44; Chi² = 36.40, df = 3 (P < 0.00001); I² = 92%									
Test for overall effect: Z = 2.95 (P = 0.003)									
**Total (95% CI)**			**740**			**738**	**100.0%**	**1.38 [1.04, 1.72]**	
Heterogeneity: Tau² = 0.42; Chi² = 129.01, df = 15 (P < 0.00001); I² = 88%									
Test for overall effect: Z = 7.97 (P < 0.00001)									
Test for subgroup differences: Chi² = 4.06, df = 2 (P = 0.13), I² = 50.8%									

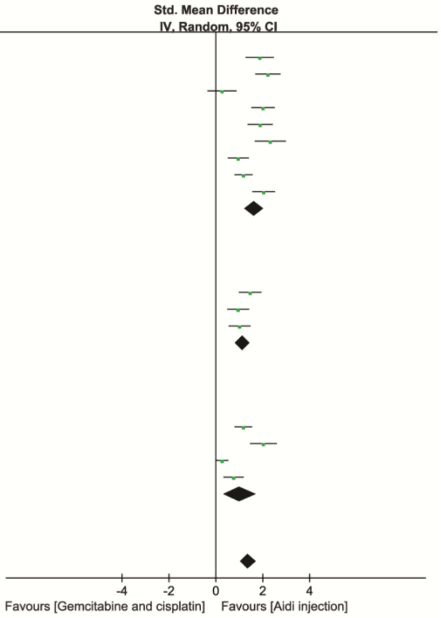

Figure S5 Subgroups analysis of $CD3^{+}$ $CD4^{+}$ T cells by anticipated survival time

Study or Subgroup	Aidi injection Mean	SD	Total	Gemcitabine and cisplatin Mean	SD	Total	Weight	Std. Mean Difference IV, Random, 95% CI	Year
**AST(≥3m)**									
Jiang,S.2011	1.56	0.3	32	0.78	0.4	30	7.5%	2.19 [1.55, 2.83]	2011
Zhao, S.2015	1.25	0.49	43	1.07	0.25	43	8.6%	0.46 [0.03, 0.89]	2015
Li, X.2015	1.49	0.04	20	1.47	0.34	20	7.6%	0.08 [-0.54, 0.70]	2015
Li, J.2016	1.31	0.26	47	1.23	0.24	47	8.7%	0.32 [-0.09, 0.72]	2016
Lv, W.2018	1.8	0.3	30	1.06	0.2	30	7.0%	2.86 [2.13, 3.60]	2018
Zhao,J.2019	0.74	0.12	43	0.64	0.29	43	8.6%	0.45 [0.02, 0.87]	2019
Guo, X.2020	2.09	0.64	51	1.48	0.11	51	8.6%	1.32 [0.89, 1.75]	2020
**Subtotal (95% CI)**			**266**			**264**	**56.7%**	**1.06 [0.42, 1.71]**	
Heterogeneity: Tau² = 0.68; Chi² = 69.63, df = 6 (P < 0.00001); I² = 91%									
Test for overall effect: Z = 3.25 (P = 0.001)									
**AST (≥5m)**									
Ma, M.2017	1.4	0.32	42	1.16	0.29	42	8.5%	0.78 [0.33, 1.22]	2017
Huang, W.2017	1.1	0.32	39	0.86	0.16	40	8.4%	0.94 [0.48, 1.41]	2017
**Subtotal (95% CI)**			**81**			**82**	**17.0%**	**0.86 [0.54, 1.18]**	
Heterogeneity: Tau² = 0.00; Chi² = 0.25, df = 1 (P = 0.62); I² = 0%									
Test for overall effect: Z = 5.22 (P < 0.00001)									
**AST (Unclear)**									
Zhang, H.2014	1.31	0.32	64	1.2	0.35	64	9.0%	0.33 [-0.02, 0.67]	2014
Han, R.2015	1.14	0.23	36	0.81	0.15	36	8.0%	1.68 [1.14, 2.22]	2015
Wu, Y.2017	1.42	0.34	109	1.19	0.04	109	9.2%	0.95 [0.67, 1.23]	2017
**Subtotal (95% CI)**			**209**			**209**	**26.3%**	**0.96 [0.30, 1.61]**	
Heterogeneity: Tau² = 0.29; Chi² = 18.17, df = 2 (P = 0.0001); I² = 89%									
Test for overall effect: Z = 2.87 (P = 0.004)									
**Total (95% CI)**			**556**			**555**	**100.0%**	**0.99 [0.62, 1.35]**	
Heterogeneity: Tau² = 0.35; Chi² = 88.06, df = 11 (P < 0.00001); I² = 88%									
Test for overall effect: Z = 5.31 (P < 0.00001)									
Test for subgroup differences: Chi² = 0.34, df = 2 (P = 0.84), I² = 0%									

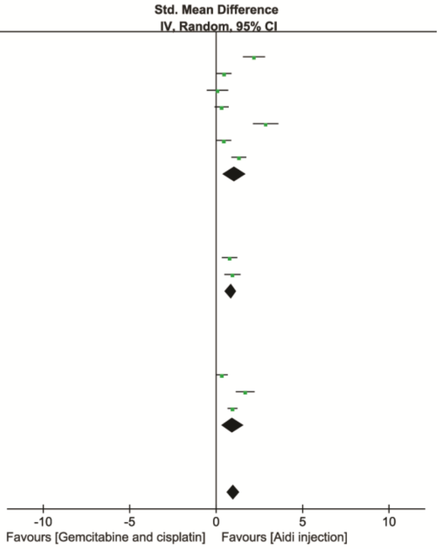

Figure S6 Subgroups analysis of $CD4^{+}/CD8^{+}$ T cells by anticipated survival time

Study or Subgroup	Aidi injection Mean	SD	Total	Gemcitabine and cisplatin Mean	SD	Total	Weight	Std. Mean Difference IV, Random, 95% CI	Year
**Primary treatment (PT)**									
Jiang,S.2011	49.2	11.6	32	36.6	10.2	30	6.5%	1.14 [0.60, 1.68]	2011
Zhao,J.2019	49.02	7.25	43	44.06	6.81	43	6.8%	0.70 [0.26, 1.13]	2019
**Subtotal (95% CI)**			**75**			**73**	**13.3%**	**0.89 [0.46, 1.31]**	
Heterogeneity: Tau² = 0.03; Chi² = 1.53, df = 1 (P = 0.22); I² = 35%									
Test for overall effect: Z = 4.09 (P < 0.0001)									
**Unclear**									
Zhang, H.2014	67.05	5.42	64	68.26	5.21	64	7.0%	-0.23 [-0.57, 0.12]	2014
Li, X.2015	59.19	4.9	20	57.61	4.84	20	6.3%	0.32 [-0.31, 0.94]	2015
Han, R.2015	58.29	2.57	36	49.85	3.79	36	6.3%	2.58 [1.95, 3.21]	2015
Zhao, S.2015	65.19	6.85	43	53.84	4.3	43	6.6%	1.97 [1.45, 2.49]	2015
Li, J.2016	62.3	3.2	47	58.4	3	47	6.8%	1.25 [0.80, 1.69]	2016
Wu, Y.2017	59.19	4.9	109	57.61	4.84	109	7.1%	0.32 [0.06, 0.59]	2017
Ma, M.2017	62.37	5.43	42	52.75	6.52	42	6.6%	1.59 [1.10, 2.08]	2017
Huang, W.2017	57.48	6.35	39	42.06	3.56	40	6.2%	2.98 [2.33, 3.63]	2017
Lv, W.2018	67	6	30	65	7	30	6.6%	0.30 [-0.21, 0.81]	2018
Liu, H.2019	60.76	4.88	44	54.47	5.89	44	6.7%	1.15 [0.70, 1.61]	2019
Guo, X.2020	53.58	5.19	51	53.26	5.18	51	6.9%	0.06 [-0.33, 0.45]	2020
Tan, H.2020	55.64	7.85	60	50.15	8.41	60	6.9%	0.67 [0.30, 1.04]	2020
Xu,Z.2020	60.75	4.51	40	54.69	5.37	40	6.7%	1.21 [0.73, 1.69]	2020
**Subtotal (95% CI)**			**625**			**626**	**86.7%**	**1.07 [0.60, 1.54]**	
Heterogeneity: Tau² = 0.69; Chi² = 177.76, df = 12 (P < 0.00001); I² = 93%									
Test for overall effect: Z = 4.44 (P < 0.00001)									
**Total (95% CI)**			**700**			**699**	**100.0%**	**1.04 [0.63, 1.46]**	
Heterogeneity: Tau² = 0.60; Chi² = 179.53, df = 14 (P < 0.00001); I² = 92%									
Test for overall effect: Z = 4.96 (P < 0.00001)									
Test for subgroup differences: Chi² = 0.31, df = 1 (P = 0.58), I² = 0%									

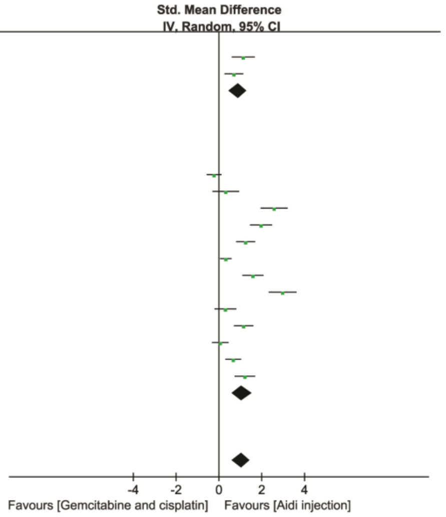

Figure S7 Subgroups analysis of CD3^{+} T cells by treatment process

Study or Subgroup	Aidi injection Mean	SD	Total	Gemcitabine and cisplatin Mean	SD	Total	Weight	Std. Mean Difference IV, Random, 95% CI	Year
**Primary treatment (PT)**									
Jiang,S.2011	37.6	5.4	32	26.3	6.5	30	5.9%	1.87 [1.27, 2.48]	2011
Zhao,J.2019	39.91	5.08	43	34.82	5.47	43	6.4%	0.96 [0.51, 1.40]	2019
**Subtotal (95% CI)**			**75**			**73**	**12.3%**	**1.39 [0.49, 2.29]**	
Heterogeneity: Tau² = 0.35; Chi² = 5.73, df = 1 (P = 0.02); I² = 83%									
Test for overall effect: Z = 3.04 (P = 0.002)									
**Unclear**									
Zhang, H.2014	38.11	3.15	64	34.23	3.43	64	6.6%	1.17 [0.80, 1.55]	2014
Zhao, S.2015	35.11	1.53	43	30.25	2.66	43	6.1%	2.22 [1.68, 2.76]	2015
Li, X.2015	38.11	5.31	20	36.76	4.69	20	5.8%	0.26 [-0.36, 0.89]	2015
Han, R.2015	34.16	5.25	36	23.28	5.36	36	6.0%	2.03 [1.45, 2.60]	2015
Li, J.2016	35.2	3.1	47	29.3	2.7	47	6.2%	2.01 [1.51, 2.51]	2016
Ma, M.2017	22.36	5.04	42	16.45	2.58	42	6.3%	1.46 [0.98, 1.95]	2017
Su, S.2017	42.9	4.89	40	33.32	5.16	39	6.1%	1.89 [1.35, 2.42]	2017
Huang, W.2017	33.34	5.18	39	28.92	3.93	40	6.3%	0.95 [0.49, 1.42]	2017
Wu, Y.2017	38.11	5.31	109	36.76	4.69	109	6.9%	0.27 [0.00, 0.54]	2017
Lv, W.2018	36	3	30	30	2	30	5.7%	2.32 [1.66, 2.99]	2018
Liu, H.2019	36.01	4.13	44	33.38	2.55	44	6.5%	0.76 [0.33, 1.19]	2019
Guo, X.2020	47.26	4.28	51	40.17	2.35	51	6.3%	2.04 [1.56, 2.52]	2020
Tan, H.2020	36.26	5.48	60	30.48	4.17	60	6.6%	1.18 [0.79, 1.57]	2020
Xu,Z.2020	36.36	4.25	40	32.96	2.05	40	6.3%	1.01 [0.54, 1.48]	2020
**Subtotal (95% CI)**			**665**			**665**	**87.7%**	**1.38 [1.01, 1.76]**	
Heterogeneity: Tau² = 0.45; Chi² = 123.01, df = 13 (P < 0.00001); I² = 89%									
Test for overall effect: Z = 7.19 (P < 0.00001)									
**Total (95% CI)**			**740**			**738**	**100.0%**	**1.38 [1.04, 1.72]**	
Heterogeneity: Tau² = 0.42; Chi² = 129.01, df = 15 (P < 0.00001); I² = 88%									
Test for overall effect: Z = 7.97 (P < 0.00001)									
Test for subgroup differences: Chi² = 0.00, df = 1 (P = 0.99), I² = 0%									

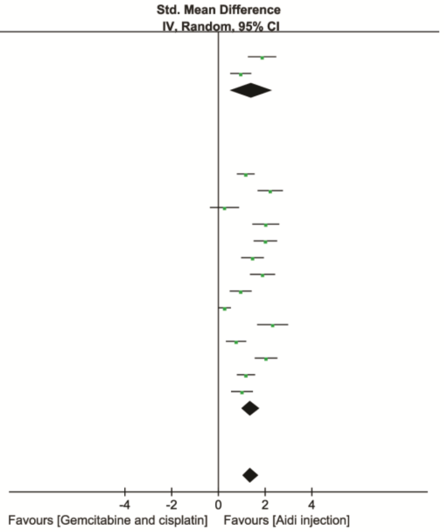

Figure S8 Subgroups analysis of CD3^{+} CD4^{+} T cells by treatment process

Study or Subgroup	Aidi injection Mean	SD	Total	Gemcitabine and cisplatin Mean	SD	Total	Weight	Std. Mean Difference IV, Random, 95% CI	Year
**Primary treatment (PT)**									
Jiang,S.2011	1.56	0.3	32	0.78	0.4	30	7.5%	2.19 [1.55, 2.83]	2011
Zhao,J.2019	0.74	0.12	43	0.64	0.29	43	8.6%	0.45 [0.02, 0.87]	2019
**Subtotal (95% CI)**			**75**			**73**	**16.2%**	**1.30 [-0.41, 3.01]**	
Heterogeneity: Tau² = 1.44; Chi² = 19.76, df = 1 (P < 0.00001); I² = 95%									
Test for overall effect: Z = 1.49 (P = 0.14)									
**Unclear**									
Zhang, H.2014	1.31	0.32	64	1.2	0.35	64	9.0%	0.33 [-0.02, 0.67]	2014
Li, X.2015	1.49	0.04	20	1.47	0.34	20	7.6%	0.08 [-0.54, 0.70]	2015
Zhao, S.2015	1.25	0.49	43	1.07	0.25	43	8.6%	0.46 [0.03, 0.89]	2015
Han, R.2015	1.14	0.23	36	0.81	0.15	36	8.0%	1.68 [1.14, 2.22]	2015
Li, J.2016	1.31	0.26	47	1.23	0.24	47	8.7%	0.32 [-0.09, 0.72]	2016
Ma, M.2017	1.4	0.32	42	1.16	0.29	42	8.5%	0.78 [0.33, 1.22]	2017
Wu, Y.2017	1.42	0.34	109	1.19	0.04	109	9.2%	0.95 [0.67, 1.23]	2017
Huang, W.2017	1.1	0.32	39	0.86	0.16	40	8.4%	0.94 [0.48, 1.41]	2017
Lv, W.2018	1.8	0.3	30	1.06	0.2	30	7.0%	2.86 [2.13, 3.60]	2018
Guo, X.2020	2.09	0.64	51	1.48	0.11	51	8.6%	1.32 [0.89, 1.75]	2020
**Subtotal (95% CI)**			**481**			**482**	**83.8%**	**0.93 [0.56, 1.31]**	
Heterogeneity: Tau² = 0.32; Chi² = 67.61, df = 9 (P < 0.00001); I² = 87%									
Test for overall effect: Z = 4.83 (P < 0.00001)									
**Total (95% CI)**			**556**			**555**	**100.0%**	**0.99 [0.62, 1.35]**	
Heterogeneity: Tau² = 0.35; Chi² = 88.06, df = 11 (P < 0.00001); I² = 88%									
Test for overall effect: Z = 5.31 (P < 0.00001)									
Test for subgroup differences: Chi² = 0.17, df = 1 (P = 0.68), I² = 0%									

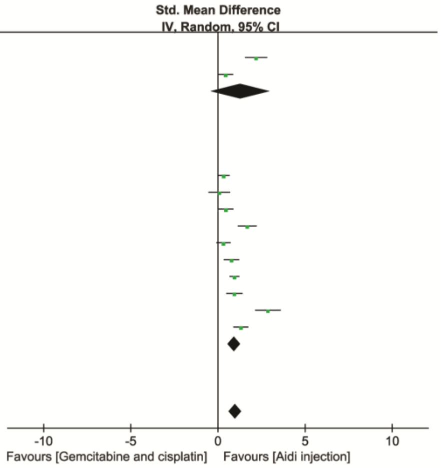

Figure S9 Subgroups analysis of CD4^{+}/CD8^{+} T cells by treatment process

Study or Subgroup	Aidi injection Mean	SD	Total	Gemcitabine and cisplatin Mean	SD	Total	Weight	Std. Mean Difference IV, Random, 95% CI	Year
**Age (≥60)**									
Jiang,S.2011	49.2	11.6	32	36.6	10.2	30	6.5%	1.14 [0.60, 1.68]	2011
Tan, H.2020	55.64	7.85	60	50.15	8.41	60	6.9%	0.67 [0.30, 1.04]	2020
**Subtotal (95% CI)**			**92**			**90**	**13.5%**	**0.86 [0.41, 1.31]**	
Heterogeneity: Tau² = 0.05; Chi² = 1.96, df = 1 (P = 0.16); I² = 49%									
Test for overall effect: Z = 3.76 (P = 0.0002)									
**Unclear**									
Zhang, H.2014	67.05	5.42	64	68.26	5.21	64	7.0%	-0.23 [-0.57, 0.12]	2014
Zhao, S.2015	65.19	6.85	43	53.84	4.3	43	6.6%	1.97 [1.45, 2.49]	2015
Han, R.2015	58.29	2.57	36	49.85	3.79	36	6.3%	2.58 [1.95, 3.21]	2015
Li, X.2015	59.19	4.9	20	57.61	4.84	20	6.3%	0.32 [-0.31, 0.94]	2015
Li, J.2016	62.3	3.2	47	58.4	3	47	6.8%	1.25 [0.80, 1.69]	2016
Huang, W.2017	57.48	6.35	39	42.06	3.56	40	6.2%	2.98 [2.33, 3.63]	2017
Ma, M.2017	62.37	5.43	42	52.75	6.52	42	6.6%	1.59 [1.10, 2.08]	2017
Wu, Y.2017	59.19	4.9	109	57.61	4.84	109	7.1%	0.32 [0.06, 0.59]	2017
Lv, W.2018	67	6	30	65	7	30	6.6%	0.30 [-0.21, 0.81]	2018
Liu, H.2019	60.76	4.88	44	54.47	5.89	44	6.7%	1.15 [0.70, 1.61]	2019
Zhao,J.2019	49.02	7.25	43	44.06	6.81	43	6.8%	0.70 [0.26, 1.13]	2019
Guo, X.2020	53.58	5.19	51	53.26	5.18	51	6.9%	0.06 [-0.33, 0.45]	2020
Xu,Z.2020	60.75	4.51	40	54.69	5.37	40	6.7%	1.21 [0.73, 1.69]	2020
**Subtotal (95% CI)**			**608**			**609**	**86.5%**	**1.07 [0.59, 1.55]**	
Heterogeneity: Tau² = 0.71; Chi² = 177.54, df = 12 (P < 0.00001); I² = 93%									
Test for overall effect: Z = 4.39 (P < 0.0001)									
**Total (95% CI)**			**700**			**699**	**100.0%**	**1.04 [0.63, 1.46]**	
Heterogeneity: Tau² = 0.60; Chi² = 179.53, df = 14 (P < 0.00001); I² = 92%									
Test for overall effect: Z = 4.96 (P < 0.00001)									
Test for subgroup differences: Chi² = 0.39, df = 1 (P = 0.53), I² = 0%									

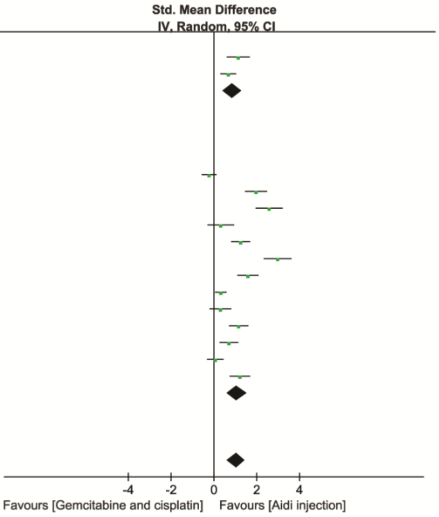

Figure S10 Subgroups analysis of $CD3^+$ T cells by age

Study or Subgroup	Aidi injection Mean	SD	Total	Gemcitabine and cisplatin Mean	SD	Total	Weight	Std. Mean Difference IV, Random, 95% CI	Year
**Age (≥60)**									
Jiang,S.2011	37.6	5.4	32	26.3	6.5	30	5.9%	1.87 [1.27, 2.48]	2011
Tan, H.2020	36.26	5.48	60	30.48	4.17	60	6.6%	1.18 [0.79, 1.57]	2020
**Subtotal (95% CI)**			**92**			**90**	**12.5%**	**1.49 [0.81, 2.16]**	
Heterogeneity: Tau² = 0.17; Chi² = 3.59, df = 1 (P = 0.06); I² = 72%									
Test for overall effect: Z = 4.31 (P < 0.0001)									
**Unclear**									
Zhang, H.2014	38.11	3.15	64	34.23	3.43	64	6.6%	1.17 [0.80, 1.55]	2014
Li, X.2015	38.11	5.31	20	36.76	4.69	20	5.8%	0.26 [-0.36, 0.89]	2015
Zhao, S.2015	35.11	1.53	43	30.25	2.66	43	6.1%	2.22 [1.68, 2.76]	2015
Han, R.2015	34.16	5.25	36	23.28	5.36	36	6.0%	2.03 [1.45, 2.60]	2015
Li, J.2016	35.2	3.1	47	29.3	2.7	47	6.2%	2.01 [1.51, 2.51]	2016
Su, S.2017	42.9	4.89	40	33.32	5.16	39	6.1%	1.89 [1.35, 2.42]	2017
Ma, M.2017	22.36	5.04	42	16.45	2.58	42	6.3%	1.46 [0.98, 1.95]	2017
Wu, Y.2017	38.11	5.31	109	36.76	4.69	109	6.9%	0.27 [0.00, 0.54]	2017
Huang, W.2017	33.34	5.18	39	28.92	3.93	40	6.3%	0.95 [0.49, 1.42]	2017
Lv, W.2018	36	3	30	30	2	30	5.7%	2.32 [1.66, 2.99]	2018
Liu, H.2019	36.01	4.13	44	33.38	2.55	44	6.5%	0.76 [0.33, 1.19]	2019
Zhao,J.2019	39.91	5.08	43	34.82	5.47	43	6.4%	0.96 [0.51, 1.40]	2019
Guo, X.2020	47.26	4.28	51	40.17	2.35	51	6.3%	2.04 [1.56, 2.52]	2020
Xu,Z.2020	36.36	4.25	40	32.96	2.05	40	6.3%	1.01 [0.54, 1.48]	2020
**Subtotal (95% CI)**			**648**			**648**	**87.5%**	**1.37 [0.98, 1.75]**	
Heterogeneity: Tau² = 0.47; Chi² = 123.92, df = 13 (P < 0.00001); I² = 90%									
Test for overall effect: Z = 7.01 (P < 0.00001)									
**Total (95% CI)**			**740**			**738**	**100.0%**	**1.38 [1.04, 1.72]**	
Heterogeneity: Tau² = 0.42; Chi² = 129.01, df = 15 (P < 0.00001); I² = 88%									
Test for overall effect: Z = 7.97 (P < 0.00001)									
Test for subgroup differences: Chi² = 0.09, df = 1 (P = 0.76), I² = 0%									

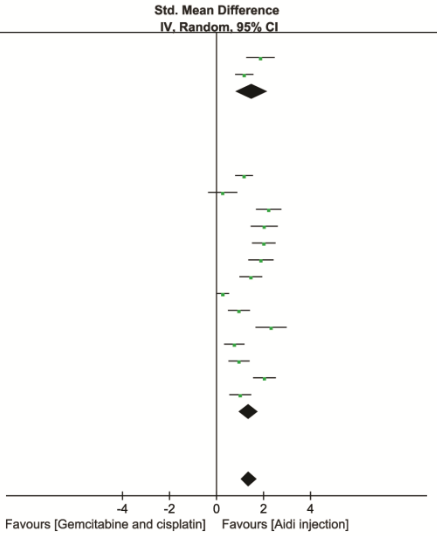

Figure S11 Subgroups analysis of $CD3^+$ $CD4^+$ T cells by age

Study or Subgroup	Aidi injection Mean	SD	Total	Gemcitabine and cisplatin Mean	SD	Total	Weight	Std. Mean Difference IV, Random, 95% CI
**Age (≥60)**								
Jiang,S.2011	1.56	0.3	32	0.78	0.4	30	7.5%	2.19 [1.55, 2.83]
**Subtotal (95% CI)**			**32**			**30**	**7.5%**	**2.19 [1.55, 2.83]**
Heterogeneity: Not applicable								
Test for overall effect: Z = 6.73 (P < 0.00001)								
**Unclear**								
Guo, X.2020	2.09	0.64	51	1.48	0.11	51	8.6%	1.32 [0.89, 1.75]
Han, R.2015	1.14	0.23	36	0.81	0.15	36	8.0%	1.68 [1.14, 2.22]
Huang, W.2017	1.1	0.32	39	0.86	0.16	40	8.4%	0.94 [0.48, 1.41]
Li, J.2016	1.31	0.26	47	1.23	0.24	47	8.7%	0.32 [-0.09, 0.72]
Li, X.2015	1.49	0.04	20	1.47	0.34	20	7.6%	0.08 [-0.54, 0.70]
Lv, W.2018	1.8	0.3	30	1.06	0.2	30	7.0%	2.86 [2.13, 3.60]
Ma, M.2017	1.4	0.32	42	1.16	0.29	42	8.5%	0.78 [0.33, 1.22]
Wu, Y.2017	1.42	0.34	109	1.19	0.04	109	9.2%	0.95 [0.67, 1.23]
Zhang, H.2014	1.31	0.32	64	1.2	0.35	64	9.0%	0.33 [-0.02, 0.67]
Zhao,J.2019	0.74	0.12	43	0.64	0.29	43	8.6%	0.45 [0.02, 0.87]
Zhao, S.2015	1.25	0.49	43	1.07	0.25	43	8.6%	0.46 [0.03, 0.89]
**Subtotal (95% CI)**			**524**			**525**	**92.5%**	**0.89 [0.54, 1.24]**
Heterogeneity: Tau² = 0.29; Chi² = 70.39, df = 10 (P < 0.00001); I² = 86%								
Test for overall effect: Z = 4.98 (P < 0.00001)								
**Total (95% CI)**			**556**			**555**	**100.0%**	**0.99 [0.62, 1.35]**
Heterogeneity: Tau² = 0.35; Chi² = 88.06, df = 11 (P < 0.00001); I² = 88%								
Test for overall effect: Z = 5.31 (P < 0.00001)								
Test for subgroup differences: Chi² = 12.32, df = 1 (P = 0.0004), I² = 91.9%								

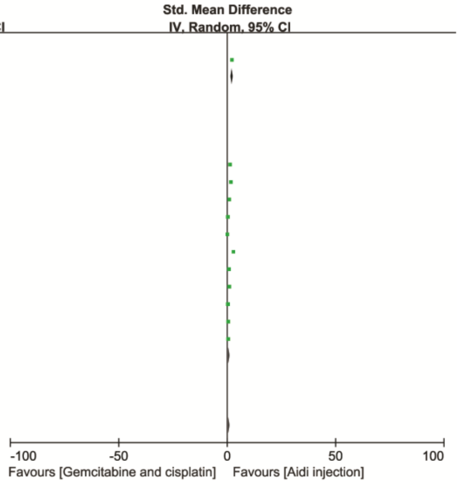

Figure S12 Subgroups analysis of $CD4^+$/$CD8^+$ T cells by age

Study or Subgroup	Aidi injection Mean	SD	Total	Gemcitabine and cisplatin Mean	SD	Total	Weight	Std. Mean Difference IV, Random, 95% CI	Year
**50-60ml**									
Zhang, H.2014	67.05	5.42	64	68.26	5.21	64	7.0%	-0.23 [-0.57, 0.12]	2014
Han, R.2015	58.29	2.57	36	49.85	3.79	36	6.3%	2.58 [1.95, 3.21]	2015
Li, X.2015	59.19	4.9	20	57.61	4.84	20	6.3%	0.32 [-0.31, 0.94]	2015
Huang, W.2017	57.48	6.35	39	42.06	3.56	40	6.2%	2.98 [2.33, 3.63]	2017
Ma, M.2017	62.37	5.43	42	52.75	6.52	42	6.6%	1.59 [1.10, 2.08]	2017
Wu, Y.2017	59.19	4.9	109	57.61	4.84	109	7.1%	0.32 [0.06, 0.59]	2017
Lv, W.2018	67	6	30	65	7	30	6.6%	0.30 [-0.21, 0.81]	2018
Liu, H.2019	60.76	4.88	44	54.47	5.89	44	6.7%	1.15 [0.70, 1.61]	2019
Zhao,J.2019	49.02	7.25	43	44.06	6.81	43	6.8%	0.70 [0.26, 1.13]	2019
Tan, H.2020	55.64	7.85	60	50.15	8.41	60	6.9%	0.67 [0.30, 1.04]	2020
Guo, X.2020	53.58	5.19	51	53.26	5.18	51	6.9%	0.06 [-0.33, 0.45]	2020
**Subtotal (95% CI)**			**538**			**539**	**73.5%**	**0.92 [0.42, 1.42]**	
Heterogeneity: Tau² = 0.65; Chi² = 145.49, df = 10 (P < 0.00001); I² = 93%									
Test for overall effect: Z = 3.63 (P = 0.0003)									
**90-100ml**									
Jiang,S.2011	49.2	11.6	32	36.6	10.2	30	6.5%	1.14 [0.60, 1.68]	2011
Zhao, S.2015	65.19	6.85	43	53.84	4.3	43	6.6%	1.97 [1.45, 2.49]	2015
**Subtotal (95% CI)**			**75**			**73**	**13.1%**	**1.56 [0.74, 2.37]**	
Heterogeneity: Tau² = 0.27; Chi² = 4.73, df = 1 (P = 0.03); I² = 79%									
Test for overall effect: Z = 3.75 (P = 0.0002)									
**1.1.3 Others**									
Li, J.2016	62.3	3.2	47	58.4	3	47	6.8%	1.25 [0.80, 1.69]	2016
Xu,Z.2020	60.75	4.51	40	54.69	5.37	40	6.7%	1.21 [0.73, 1.69]	2020
**Subtotal (95% CI)**			**87**			**87**	**13.4%**	**1.23 [0.90, 1.56]**	
Heterogeneity: Tau² = 0.00; Chi² = 0.01, df = 1 (P = 0.91); I² = 0%									
Test for overall effect: Z = 7.41 (P < 0.00001)									
**Total (95% CI)**			**700**			**699**	**100.0%**	**1.04 [0.63, 1.46]**	
Heterogeneity: Tau² = 0.60; Chi² = 179.53, df = 14 (P < 0.00001); I² = 92%									
Test for overall effect: Z = 4.96 (P < 0.00001)									
Test for subgroup differences: Chi² = 1.95, df = 2 (P = 0.38), I² = 0%									

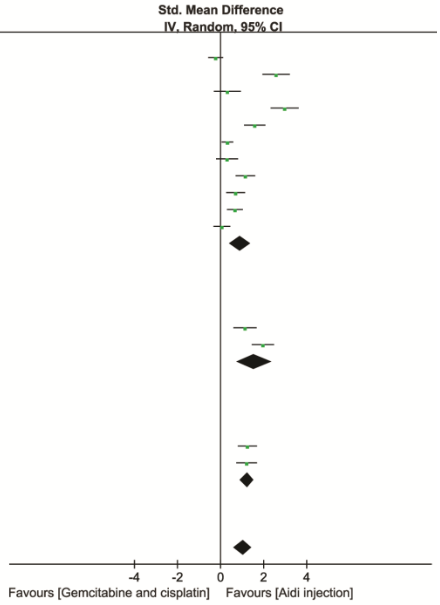

Figure S13 Subgroups analysis of $CD3^+$ T cells by dosage of Aidi injection

Study or Subgroup	Aidi injection Mean	SD	Total	Gemcitabine and cisplatin Mean	SD	Total	Weight	Std. Mean Difference IV, Random, 95% CI	Year
**50-60ml**									
Zhang, H.2014	38.11	3.15	64	34.23	3.43	64	6.6%	1.17 [0.80, 1.55]	2014
Li, X.2015	38.11	5.31	20	36.76	4.69	20	5.8%	0.26 [-0.36, 0.89]	2015
Han, R.2015	34.16	5.25	36	23.28	5.36	36	6.0%	2.03 [1.45, 2.60]	2015
Ma, M.2017	22.36	5.04	42	16.45	2.58	42	6.3%	1.46 [0.98, 1.95]	2017
Huang, W.2017	33.34	5.18	39	28.92	3.93	40	6.3%	0.95 [0.49, 1.42]	2017
Su, S.2017	42.9	4.89	40	33.32	5.16	39	6.1%	1.89 [1.35, 2.42]	2017
Wu, Y.2017	38.11	5.31	109	36.76	4.69	109	6.9%	0.27 [0.00, 0.54]	2017
Lv, W.2018	36	3	30	30	2	30	5.7%	2.32 [1.66, 2.99]	2018
Liu, H.2019	36.01	4.13	44	33.38	2.55	44	6.5%	0.76 [0.33, 1.19]	2019
Zhao,J.2019	39.91	5.08	43	34.82	5.47	43	6.4%	0.96 [0.51, 1.40]	2019
Tan, H.2020	36.26	5.48	60	30.48	4.17	60	6.6%	1.18 [0.79, 1.57]	2020
Guo, X.2020	47.26	4.28	51	40.17	2.35	51	6.3%	2.04 [1.56, 2.52]	2020
**Subtotal (95% CI)**			**578**			**578**	**75.5%**	**1.26 [0.87, 1.64]**	
Heterogeneity: Tau² = 0.40; Chi² = 95.59, df = 11 (P < 0.00001); I² = 88%									
Test for overall effect: Z = 6.44 (P < 0.00001)									
**90-100ml**									
Jiang,S.2011	37.6	5.4	32	26.3	6.5	30	5.9%	1.87 [1.27, 2.48]	2011
Zhao, S.2015	35.11	1.53	43	30.25	2.66	43	6.1%	2.22 [1.68, 2.76]	2015
**Subtotal (95% CI)**			**75**			**73**	**12.0%**	**2.06 [1.66, 2.47]**	
Heterogeneity: Tau² = 0.00; Chi² = 0.70, df = 1 (P = 0.40); I² = 0%									
Test for overall effect: Z = 10.03 (P < 0.00001)									
**Others**									
Li, J.2016	35.2	3.1	47	29.3	2.7	47	6.2%	2.01 [1.51, 2.51]	2016
Xu,Z.2020	36.36	4.25	40	32.96	2.05	40	6.3%	1.01 [0.54, 1.48]	2020
**Subtotal (95% CI)**			**87**			**87**	**12.6%**	**1.51 [0.52, 2.49]**	
Heterogeneity: Tau² = 0.44; Chi² = 8.28, df = 1 (P = 0.004); I² = 88%									
Test for overall effect: Z = 3.00 (P = 0.003)									
**Total (95% CI)**			**740**			**738**	**100.0%**	**1.38 [1.04, 1.72]**	
Heterogeneity: Tau² = 0.42; Chi² = 129.01, df = 15 (P < 0.00001); I² = 88%									
Test for overall effect: Z = 7.97 (P < 0.00001)									
Test for subgroup differences: Chi² = 8.21, df = 2 (P = 0.02), I² = 75.6%									

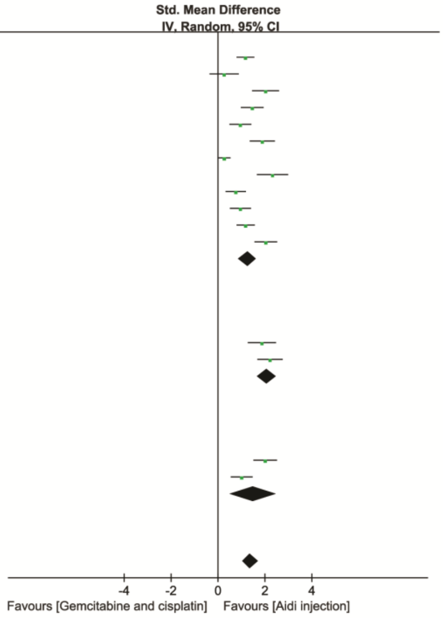

Figure S14 Subgroups analysis of $CD3^+$ $CD4^+$ T cells by dosage of Aidi injection

Study or Subgroup	Aidi injection Mean	SD	Total	Gemcitabine and cisplatin Mean	SD	Total	Weight	Std. Mean Difference IV, Random, 95% CI	Year
**50-60ml**									
Zhang, H.2014	1.31	0.32	64	1.2	0.35	64	9.0%	0.33 [-0.02, 0.67]	2014
Li, X.2015	1.49	0.04	20	1.47	0.34	20	7.6%	0.08 [-0.54, 0.70]	2015
Han, R.2015	1.14	0.23	36	0.81	0.15	36	8.0%	1.68 [1.14, 2.22]	2015
Ma, M.2017	1.4	0.32	42	1.16	0.29	42	8.5%	0.78 [0.33, 1.22]	2017
Wu, Y.2017	1.42	0.34	109	1.19	0.04	109	9.2%	0.95 [0.67, 1.23]	2017
Huang, W.2017	1.1	0.32	39	0.86	0.16	40	8.4%	0.94 [0.48, 1.41]	2017
Lv, W.2018	1.8	0.3	30	1.06	0.2	30	7.0%	2.86 [2.13, 3.60]	2018
Zhao,J.2019	0.74	0.12	43	0.64	0.29	43	8.6%	0.45 [0.02, 0.87]	2019
Guo, X.2020	2.09	0.64	51	1.48	0.11	51	8.6%	1.32 [0.89, 1.75]	2020
**Subtotal (95% CI)**			**434**			**435**	**75.1%**	**1.01 [0.60, 1.41]**	
Heterogeneity: Tau² = 0.33; Chi² = 61.05, df = 8 (P < 0.00001); I² = 87%									
Test for overall effect: Z = 4.84 (P < 0.00001)									
**90-100ml**									
Jiang,S.2011	1.56	0.3	32	0.78	0.4	30	7.5%	2.19 [1.55, 2.83]	2011
Zhao, S.2015	1.25	0.49	43	1.07	0.25	43	8.6%	0.46 [0.03, 0.89]	2015
**Subtotal (95% CI)**			**75**			**73**	**16.2%**	**1.31 [-0.39, 3.00]**	
Heterogeneity: Tau² = 1.42; Chi² = 19.48, df = 1 (P < 0.0001); I² = 95%									
Test for overall effect: Z = 1.51 (P = 0.13)									
**Others**									
Li, J.2016	1.31	0.26	47	1.23	0.24	47	8.7%	0.32 [-0.09, 0.72]	2016
**Subtotal (95% CI)**			**47**			**47**	**8.7%**	**0.32 [-0.09, 0.72]**	
Heterogeneity: Not applicable									
Test for overall effect: Z = 1.53 (P = 0.13)									
**Total (95% CI)**			**556**			**555**	**100.0%**	**0.99 [0.62, 1.35]**	
Heterogeneity: Tau² = 0.35; Chi² = 88.06, df = 11 (P < 0.00001); I² = 88%									
Test for overall effect: Z = 5.31 (P < 0.00001)									
Test for subgroup differences: Chi² = 6.04, df = 2 (P = 0.05), I² = 66.9%									

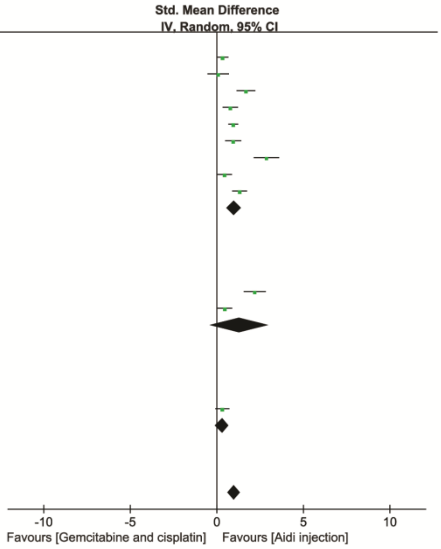

Figure S15 Subgroups analysis of $CD4^+/CD8^+$ T cells by dosage of Aidi injection

Study or Subgroup	Aidi injection Mean	SD	Total	Gemcitabine and cisplatin Mean	SD	Total	Weight	Std. Mean Difference IV, Random, 95% CI	Year
**7 to 10days**									
Zhang, H.2014	67.05	5.42	64	68.26	5.21	64	7.0%	-0.23 [-0.57, 0.12]	2014
Zhao, S.2015	65.19	6.85	43	53.84	4.3	43	6.6%	1.97 [1.45, 2.49]	2015
Li, X.2015	59.19	4.9	20	57.61	4.84	20	6.3%	0.32 [-0.31, 0.94]	2015
Tan, H.2020	55.64	7.85	60	50.15	8.41	60	6.9%	0.67 [0.30, 1.04]	2020
**Subtotal (95% CI)**			**187**			**187**	**26.8%**	**0.67 [-0.22, 1.57]**	
Heterogeneity: Tau² = 0.77; Chi² = 48.61, df = 3 (P < 0.00001); I² = 94%									
Test for overall effect: Z = 1.48 (P = 0.14)									
**14 to 15days**									
Jiang,S.2011	49.2	11.6	32	36.6	10.2	30	6.5%	1.14 [0.60, 1.68]	2011
Wu, Y.2017	59.19	4.9	109	57.61	4.84	109	7.1%	0.32 [0.06, 0.59]	2017
Lv, W.2018	67	6	30	65	7	30	6.6%	0.30 [-0.21, 0.81]	2018
Guo, X.2020	53.58	5.19	51	53.26	5.18	51	6.9%	0.06 [-0.33, 0.45]	2020
**Subtotal (95% CI)**			**222**			**220**	**27.1%**	**0.42 [0.04, 0.80]**	
Heterogeneity: Tau² = 0.10; Chi² = 10.36, df = 3 (P = 0.02); I² = 71%									
Test for overall effect: Z = 2.16 (P = 0.03)									
**21 to 28days**									
Li, J.2016	62.3	3.2	47	58.4	3	47	6.8%	1.25 [0.80, 1.69]	2016
Huang, W.2017	57.48	6.35	39	42.06	3.56	40	6.2%	2.98 [2.33, 3.63]	2017
Zhao,J.2019	49.02	7.25	43	44.06	6.81	43	6.8%	0.70 [0.26, 1.13]	2019
Xu,Z.2020	60.75	4.51	40	54.69	5.37	40	6.7%	1.21 [0.73, 1.69]	2020
**Subtotal (95% CI)**			**169**			**170**	**26.4%**	**1.50 [0.69, 2.32]**	
Heterogeneity: Tau² = 0.63; Chi² = 33.11, df = 3 (P < 0.00001); I² = 91%									
Test for overall effect: Z = 3.61 (P = 0.0003)									
**Others**									
Han, R.2015	58.29	2.57	36	49.85	3.79	36	6.3%	2.58 [1.95, 3.21]	2015
Ma, M.2017	62.37	5.43	42	52.75	6.52	42	6.6%	1.59 [1.10, 2.08]	2017
Liu, H.2019	60.76	4.88	44	54.47	5.89	44	6.7%	1.15 [0.70, 1.61]	2019
**Subtotal (95% CI)**			**122**			**122**	**19.6%**	**1.75 [0.98, 2.51]**	
Heterogeneity: Tau² = 0.38; Chi² = 12.91, df = 2 (P = 0.002); I² = 85%									
Test for overall effect: Z = 4.47 (P < 0.00001)									
**Total (95% CI)**			**700**			**699**	**100.0%**	**1.04 [0.63, 1.46]**	
Heterogeneity: Tau² = 0.60; Chi² = 179.53, df = 14 (P < 0.00001); I² = 92%									
Test for overall effect: Z = 4.96 (P < 0.00001)									
Test for subgroup differences: Chi² = 12.62, df = 3 (P = 0.006), I² = 76.2%									

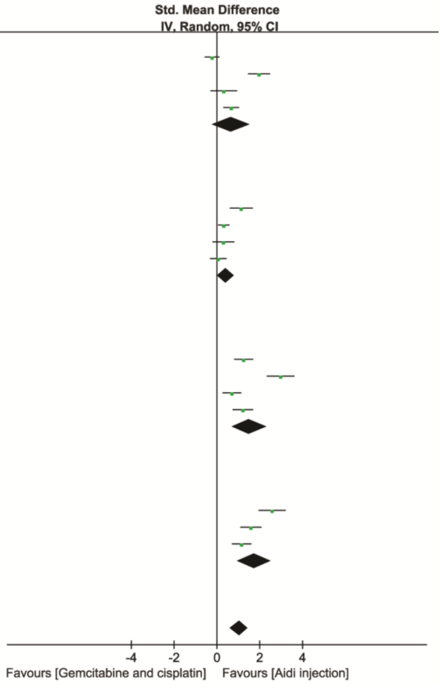

Figure S16 Subgroups analysis of $CD3^+$ T cells by treatment time

Study or Subgroup	Aidi injection Mean	Aidi injection SD	Aidi injection Total	Gemcitabine and cisplatin Mean	Gemcitabine and cisplatin SD	Gemcitabine and cisplatin Total	Weight	Std. Mean Difference IV, Random, 95% CI	Year
**7 to 10days**									
Zhang, H.2014	38.11	3.15	64	34.23	3.43	64	6.6%	1.17 [0.80, 1.55]	2014
Zhao, S.2015	35.11	1.53	43	30.25	2.66	43	6.1%	2.22 [1.68, 2.76]	2015
Li, X.2015	38.11	5.31	20	36.76	4.69	20	5.8%	0.26 [-0.36, 0.89]	2015
Tan, H.2020	36.26	5.48	60	30.48	4.17	60	6.6%	1.18 [0.79, 1.57]	2020
**Subtotal (95% CI)**			**187**			**187**	**25.1%**	**1.22 [0.58, 1.85]**	
Heterogeneity: Tau² = 0.36; Chi² = 22.19, df = 3 (P < 0.0001); I² = 86%									
Test for overall effect: Z = 3.77 (P = 0.0002)									
**14 to 15days**									
Jiang,S.2011	37.6	5.4	32	26.3	6.5	30	5.9%	1.87 [1.27, 2.48]	2011
Wu, Y.2017	38.11	5.31	109	36.76	4.69	109	6.9%	0.27 [0.00, 0.54]	2017
Lv, W.2018	36	3	30	30	2	30	5.7%	2.32 [1.66, 2.99]	2018
Guo, X.2020	47.26	4.28	51	40.17	2.35	51	6.3%	2.04 [1.56, 2.52]	2020
**Subtotal (95% CI)**			**222**			**220**	**24.7%**	**1.61 [0.47, 2.75]**	
Heterogeneity: Tau² = 1.28; Chi² = 69.99, df = 3 (P < 0.00001); I² = 96%									
Test for overall effect: Z = 2.76 (P = 0.006)									
**21 to 28days**									
Li, J.2016	35.2	3.1	47	29.3	2.7	47	6.2%	2.01 [1.51, 2.51]	2016
Huang, W.2017	33.34	5.18	39	28.92	3.93	40	6.3%	0.95 [0.49, 1.42]	2017
Su, S.2017	42.9	4.89	40	33.32	5.16	39	6.1%	1.89 [1.35, 2.42]	2017
Zhao,J.2019	39.91	5.08	43	34.82	5.47	43	6.4%	0.96 [0.51, 1.40]	2019
Xu,Z.2020	36.36	4.25	40	32.96	2.05	40	6.3%	1.01 [0.54, 1.48]	2020
**Subtotal (95% CI)**			**209**			**209**	**31.5%**	**1.35 [0.89, 1.81]**	
Heterogeneity: Tau² = 0.22; Chi² = 18.33, df = 4 (P = 0.001); I² = 78%									
Test for overall effect: Z = 5.75 (P < 0.00001)									
**Others**									
Han, R.2015	34.16	5.25	36	23.28	5.36	36	6.0%	2.03 [1.45, 2.60]	2015
Ma, M.2017	22.36	5.04	42	16.45	2.58	42	6.3%	1.46 [0.98, 1.95]	2017
Liu, H.2019	36.01	4.13	44	33.38	2.55	44	6.5%	0.76 [0.33, 1.19]	2019
**Subtotal (95% CI)**			**122**			**122**	**18.7%**	**1.40 [0.68, 2.11]**	
Heterogeneity: Tau² = 0.34; Chi² = 12.60, df = 2 (P = 0.002); I² = 84%									
Test for overall effect: Z = 3.83 (P = 0.0001)									
**Total (95% CI)**			**740**			**738**	**100.0%**	**1.38 [1.04, 1.72]**	
Heterogeneity: Tau² = 0.42; Chi² = 129.01, df = 15 (P < 0.00001); I² = 88%									
Test for overall effect: Z = 7.97 (P < 0.00001)									
Test for subgroup differences: Chi² = 0.37, df = 3 (P = 0.95), I² = 0%									

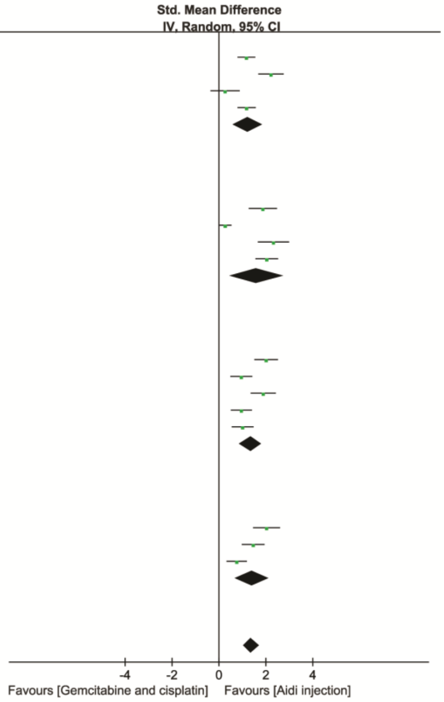

Figure S17 Subgroups analysis of $CD3^+$ $CD4^+$ T cells by treatment time

Study or Subgroup	Aidi injection Mean	Aidi injection SD	Aidi injection Total	Gemcitabine and cisplatin Mean	Gemcitabine and cisplatin SD	Gemcitabine and cisplatin Total	Weight	Std. Mean Difference IV, Random, 95% CI
**7 to 10days**								
Li, X.2015	1.49	0.04	20	1.47	0.34	20	7.6%	0.08 [-0.54, 0.70]
Zhang, H.2014	1.31	0.32	64	1.2	0.35	64	9.0%	0.33 [-0.02, 0.67]
Zhao, S.2015	1.25	0.49	43	1.07	0.25	43	8.6%	0.46 [0.03, 0.89]
**Subtotal (95% CI)**			**127**			**127**	**25.2%**	**0.33 [0.08, 0.58]**
Heterogeneity: Tau² = 0.00; Chi² = 0.97, df = 2 (P = 0.62); I² = 0%								
Test for overall effect: Z = 2.62 (P = 0.009)								
**14 to 15days**								
Guo, X.2020	2.09	0.64	51	1.48	0.11	51	8.6%	1.32 [0.89, 1.75]
Jiang,S.2011	1.56	0.3	32	0.78	0.4	30	7.5%	2.19 [1.55, 2.83]
Lv, W.2018	1.8	0.3	30	1.06	0.2	30	7.0%	2.86 [2.13, 3.60]
Wu, Y.2017	1.42	0.34	109	1.19	0.04	109	9.2%	0.95 [0.67, 1.23]
**Subtotal (95% CI)**			**222**			**220**	**32.4%**	**1.77 [1.01, 2.54]**
Heterogeneity: Tau² = 0.54; Chi² = 30.99, df = 3 (P < 0.00001); I² = 90%								
Test for overall effect: Z = 4.53 (P < 0.00001)								
**21 to 28days**								
Huang, W.2017	1.1	0.32	39	0.86	0.16	40	8.4%	0.94 [0.48, 1.41]
Li, J.2016	1.31	0.26	47	1.23	0.24	47	8.7%	0.32 [-0.09, 0.72]
Zhao,J.2019	0.74	0.12	43	0.64	0.29	43	8.6%	0.45 [0.02, 0.87]
**Subtotal (95% CI)**			**129**			**130**	**25.8%**	**0.55 [0.19, 0.92]**
Heterogeneity: Tau² = 0.05; Chi² = 4.21, df = 2 (P = 0.12); I² = 52%								
Test for overall effect: Z = 3.00 (P = 0.003)								
**Others**								
Han, R.2015	1.14	0.23	36	0.81	0.15	36	8.0%	1.68 [1.14, 2.22]
Ma, M.2017	1.4	0.32	42	1.16	0.29	42	8.5%	0.78 [0.33, 1.22]
**Subtotal (95% CI)**			**78**			**78**	**16.6%**	**1.22 [0.33, 2.10]**
Heterogeneity: Tau² = 0.34; Chi² = 6.38, df = 1 (P = 0.01); I² = 84%								
Test for overall effect: Z = 2.70 (P = 0.007)								
**Total (95% CI)**			**556**			**555**	**100.0%**	**0.99 [0.62, 1.35]**
Heterogeneity: Tau² = 0.35; Chi² = 88.06, df = 11 (P < 0.00001); I² = 88%								
Test for overall effect: Z = 5.31 (P < 0.00001)								
Test for subgroup differences: Chi² = 14.87, df = 3 (P = 0.002), I² = 79.8%								

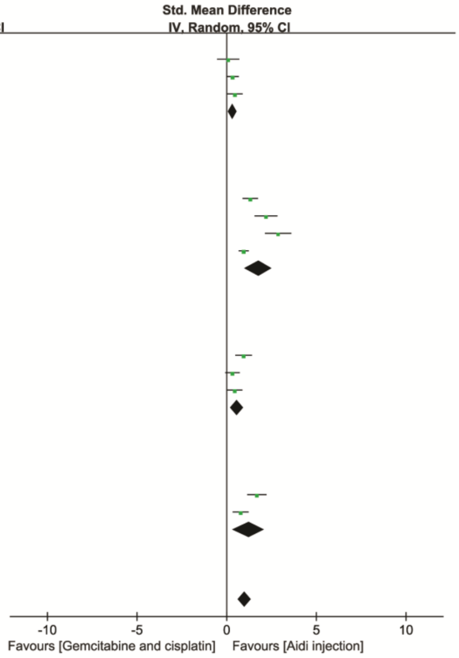

Figure S18 Subgroups analysis of $CD4^+$/$CD8^+$ T cells by treatment time

Study or Subgroup	Aidi injection Mean	Aidi injection SD	Aidi injection Total	Gemcitabine and cisplatin Mean	Gemcitabine and cisplatin SD	Gemcitabine and cisplatin Total	Weight	Std. Mean Difference IV, Random, 95% CI	Year
**One cycle**									
Li, J.2016	62.3	3.2	47	58.4	3	47	6.8%	1.25 [0.80, 1.69]	2016
**Subtotal (95% CI)**			**47**			**47**	**6.8%**	**1.25 [0.80, 1.69]**	
Heterogeneity: Not applicable									
Test for overall effect: Z = 5.51 (P < 0.00001)									
**Two cycles**									
Jiang,S.2011	49.2	11.6	32	36.6	10.2	30	6.5%	1.14 [0.60, 1.68]	2011
Li, X.2015	59.19	4.9	20	57.61	4.84	20	6.3%	0.32 [-0.31, 0.94]	2015
Zhao,J.2019	49.02	7.25	43	44.06	6.81	43	6.8%	0.70 [0.26, 1.13]	2019
Liu, H.2019	60.76	4.88	44	54.47	5.89	44	6.7%	1.15 [0.70, 1.61]	2019
Tan, H.2020	55.64	7.85	60	50.15	8.41	60	6.9%	0.67 [0.30, 1.04]	2020
Xu,Z.2020	60.75	4.51	40	54.69	5.37	40	6.7%	1.21 [0.73, 1.69]	2020
**Subtotal (95% CI)**			**239**			**237**	**40.0%**	**0.87 [0.61, 1.13]**	
Heterogeneity: Tau² = 0.05; Chi² = 9.10, df = 5 (P = 0.10); I² = 45%									
Test for overall effect: Z = 6.59 (P < 0.00001)									
**Three cycles**									
Han, R.2015	58.29	2.57	36	49.85	3.79	36	6.3%	2.58 [1.95, 3.21]	2015
Wu, Y.2017	59.19	4.9	109	57.61	4.84	109	7.1%	0.32 [0.06, 0.59]	2017
Huang, W.2017	57.48	6.35	39	42.06	3.56	40	6.2%	2.98 [2.33, 3.63]	2017
**Subtotal (95% CI)**			**184**			**185**	**19.6%**	**1.94 [0.05, 3.83]**	
Heterogeneity: Tau² = 2.72; Chi² = 83.97, df = 2 (P < 0.00001); I² = 98%									
Test for overall effect: Z = 2.01 (P = 0.04)									
**Four cycles**									
Ma, M.2017	62.37	5.43	42	52.75	6.52	42	6.6%	1.59 [1.10, 2.08]	2017
Guo, X.2020	53.58	5.19	51	53.26	5.18	51	6.9%	0.06 [-0.33, 0.45]	2020
**Subtotal (95% CI)**			**93**			**93**	**13.5%**	**0.82 [-0.68, 2.31]**	
Heterogeneity: Tau² = 1.12; Chi² = 22.74, df = 1 (P < 0.00001); I² = 96%									
Test for overall effect: Z = 1.07 (P = 0.28)									
**Others**									
Zhang, H.2014	67.05	5.42	64	68.26	5.21	64	7.0%	-0.23 [-0.57, 0.12]	2014
Zhao, S.2015	65.19	6.85	43	53.84	4.3	43	6.6%	1.97 [1.45, 2.49]	2015
Lv, W.2018	67	6	30	65	7	30	6.6%	0.30 [-0.21, 0.81]	2018
**Subtotal (95% CI)**			**137**			**137**	**20.2%**	**0.67 [-0.61, 1.95]**	
Heterogeneity: Tau² = 1.22; Chi² = 47.62, df = 2 (P < 0.00001); I² = 96%									
Test for overall effect: Z = 1.03 (P = 0.30)									
**Total (95% CI)**			**700**			**699**	**100.0%**	**1.04 [0.63, 1.46]**	
Heterogeneity: Tau² = 0.60; Chi² = 179.53, df = 14 (P < 0.00001); I² = 92%									
Test for overall effect: Z = 4.96 (P < 0.00001)									
Test for subgroup differences: Chi² = 3.28, df = 4 (P = 0.51), I² = 0%									

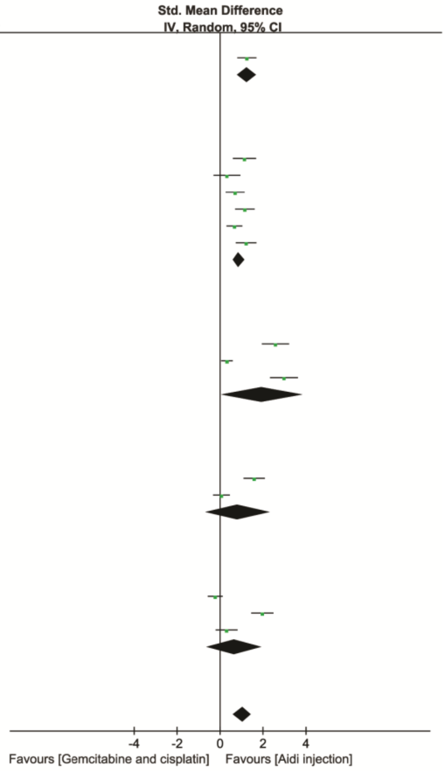

Figure S19 Subgroups analysis of $CD3^+$ T cells by treatment cycle

Study or Subgroup	Aidi injection Mean	Aidi injection SD	Aidi injection Total	Gemcitabine and cisplatin Mean	Gemcitabine and cisplatin SD	Gemcitabine and cisplatin Total	Weight	Std. Mean Difference IV, Random, 95% CI	Year
**One cycle**									
Li, J.2016	35.2	3.1	47	29.3	2.7	47	6.2%	2.01 [1.51, 2.51]	2016
**Subtotal (95% CI)**			**47**			**47**	**6.2%**	**2.01 [1.51, 2.51]**	
Heterogeneity: Not applicable									
Test for overall effect: Z = 7.89 (P < 0.00001)									
**Two cycles**									
Jiang,S.2011	37.6	5.4	32	26.3	6.5	30	5.9%	1.87 [1.27, 2.48]	2011
Li, X.2015	38.11	5.31	20	36.76	4.69	20	5.8%	0.26 [-0.36, 0.89]	2015
Su, S.2017	42.9	4.89	40	33.32	5.16	39	6.1%	1.89 [1.35, 2.42]	2017
Zhao,J.2019	39.91	5.08	43	34.82	5.47	43	6.4%	0.96 [0.51, 1.40]	2019
Liu, H.2019	36.01	4.13	44	33.38	2.55	44	6.5%	0.76 [0.33, 1.19]	2019
Xu,Z.2020	36.36	4.25	40	32.96	2.05	40	6.3%	1.01 [0.54, 1.48]	2020
Tan, H.2020	36.26	5.48	60	30.48	4.17	60	6.6%	1.18 [0.79, 1.57]	2020
**Subtotal (95% CI)**			**279**			**276**	**43.6%**	**1.13 [0.75, 1.50]**	
Heterogeneity: Tau² = 0.19; Chi² = 24.63, df = 6 (P = 0.0004); I² = 76%									
Test for overall effect: Z = 5.92 (P < 0.00001)									
**Three cycles**									
Han, R.2015	34.16	5.25	36	23.28	5.36	36	6.0%	2.03 [1.45, 2.60]	2015
Wu, Y.2017	38.11	5.31	109	36.76	4.69	109	6.9%	0.27 [0.00, 0.54]	2017
Huang, W.2017	33.34	5.18	39	28.92	3.93	40	6.3%	0.95 [0.49, 1.42]	2017
**Subtotal (95% CI)**			**184**			**185**	**19.2%**	**1.06 [0.08, 2.03]**	
Heterogeneity: Tau² = 0.69; Chi² = 31.63, df = 2 (P < 0.00001); I² = 94%									
Test for overall effect: Z = 2.12 (P = 0.03)									
**Four cycles**									
Ma, M.2017	22.36	5.04	42	16.45	2.58	42	6.3%	1.46 [0.98, 1.95]	2017
Guo, X.2020	47.26	4.28	51	40.17	2.35	51	6.3%	2.04 [1.56, 2.52]	2020
**Subtotal (95% CI)**			**93**			**93**	**12.6%**	**1.75 [1.19, 2.31]**	
Heterogeneity: Tau² = 0.10; Chi² = 2.73, df = 1 (P = 0.10); I² = 63%									
Test for overall effect: Z = 6.09 (P < 0.00001)									
**Others**									
Zhang, H.2014	38.11	3.15	64	34.23	3.43	64	6.6%	1.17 [0.80, 1.55]	2014
Zhao, S.2015	35.11	1.53	43	30.25	2.66	43	6.1%	2.22 [1.68, 2.76]	2015
Lv, W.2018	36	3	30	30	2	30	5.7%	2.32 [1.66, 2.99]	2018
**Subtotal (95% CI)**			**137**			**137**	**18.4%**	**1.87 [1.07, 2.68]**	
Heterogeneity: Tau² = 0.43; Chi² = 14.42, df = 2 (P = 0.0007); I² = 86%									
Test for overall effect: Z = 4.57 (P < 0.00001)									
**Total (95% CI)**			**740**			**738**	**100.0%**	**1.38 [1.04, 1.72]**	
Heterogeneity: Tau² = 0.42; Chi² = 129.01, df = 15 (P < 0.00001); I² = 88%									
Test for overall effect: Z = 7.97 (P < 0.00001)									
Test for subgroup differences: Chi² = 10.21, df = 4 (P = 0.04), I² = 60.8%									

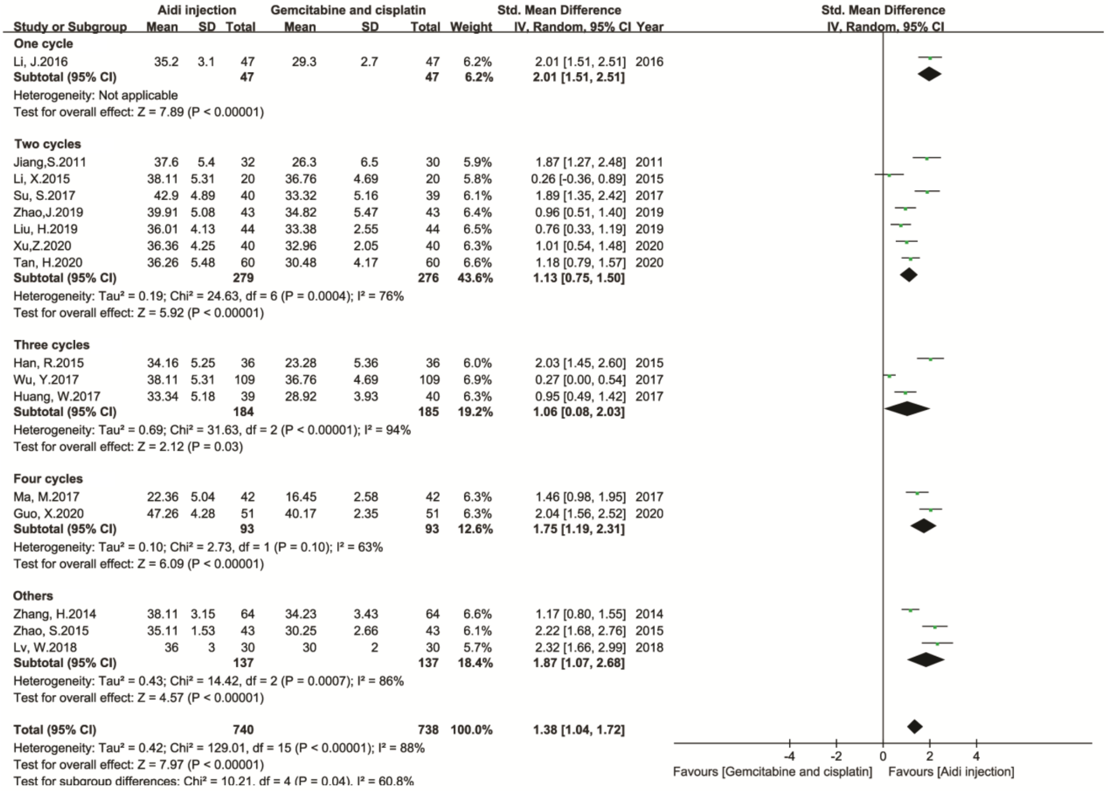

Figure S20 Subgroups analysis of $CD3^+$ $CD4^+$ T cells by treatment cycle

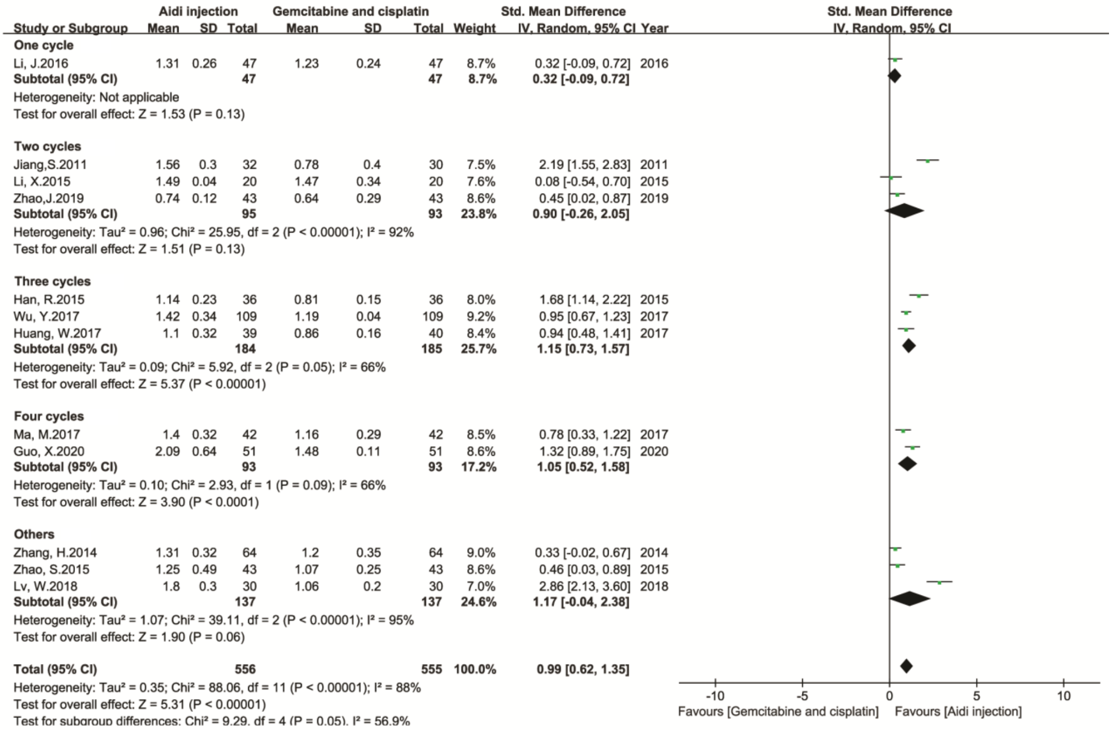

Figure S21 Subgroups analysis of $CD4^{+}/CD8^{+}$ T cells by treatment cycle

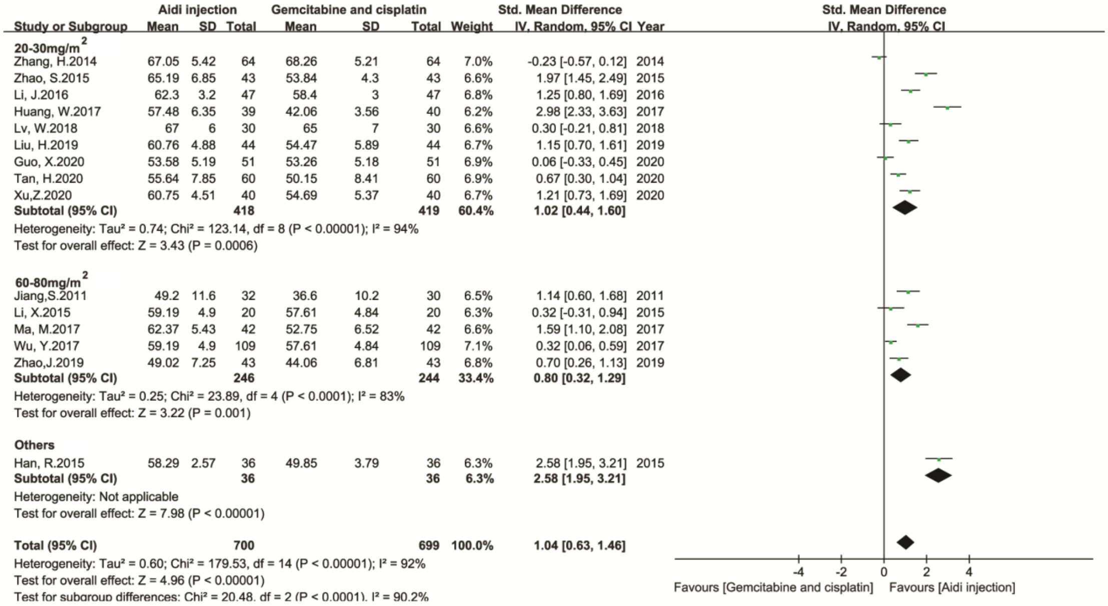

Figure S22 Subgroups analysis of $CD3^{+}$ T cells by cisplatin dosage

Study or Subgroup	Aidi injection Mean	SD	Total	Gemcitabine and cisplatin Mean	SD	Total	Weight	Std. Mean Difference IV, Random, 95% CI	Year
**20-30mg/m²**									
Zhang, H.2014	38.11	3.15	64	34.23	3.43	64	6.6%	1.17 [0.80, 1.55]	2014
Zhao, S.2015	35.11	1.53	43	30.25	2.66	43	6.1%	2.22 [1.68, 2.76]	2015
Li, J.2016	35.2	3.1	47	29.3	2.7	47	6.2%	2.01 [1.51, 2.51]	2016
Huang, W.2017	33.34	5.18	39	28.92	3.93	40	6.3%	0.95 [0.49, 1.42]	2017
Su, S.2017	42.9	4.89	40	33.32	5.16	39	6.1%	1.89 [1.35, 2.42]	2017
Lv, W.2018	36	3	30	30	2	30	5.7%	2.32 [1.66, 2.99]	2018
Liu, H.2019	36.01	4.13	44	33.38	2.55	44	6.5%	0.76 [0.33, 1.19]	2019
Xu,Z.2020	36.36	4.25	40	32.96	2.05	40	6.3%	1.01 [0.54, 1.48]	2020
Tan, H.2020	36.26	5.48	60	30.48	4.17	60	6.6%	1.18 [0.79, 1.57]	2020
Guo, X.2020	47.26	4.28	51	40.17	2.35	51	6.3%	2.04 [1.56, 2.52]	2020
**Subtotal (95% CI)**			**458**			**458**	**62.7%**	**1.53 [1.18, 1.88]**	
Heterogeneity: Tau² = 0.26; Chi² = 49.00, df = 9 (P < 0.00001); I² = 82%									
Test for overall effect: Z = 8.58 (P < 0.00001)									
**60-80mg/m²**									
Jiang,S.2011	37.6	5.4	32	26.3	6.5	30	5.9%	1.87 [1.27, 2.48]	2011
Li, X.2015	38.11	5.31	20	36.76	4.69	20	5.8%	0.26 [-0.36, 0.89]	2015
Wu, Y.2017	38.11	5.31	109	36.76	4.69	109	6.9%	0.27 [0.00, 0.54]	2017
Ma, M.2017	22.36	5.04	42	16.45	2.58	42	6.3%	1.46 [0.98, 1.95]	2017
Zhao,J.2019	39.91	5.08	43	34.82	5.47	43	6.4%	0.96 [0.51, 1.40]	2019
**Subtotal (95% CI)**			**246**			**244**	**31.3%**	**0.95 [0.33, 1.57]**	
Heterogeneity: Tau² = 0.44; Chi² = 37.20, df = 4 (P < 0.00001); I² = 89%									
Test for overall effect: Z = 3.00 (P = 0.003)									
**Others**									
Han, R.2015	34.16	5.25	36	23.28	5.36	36	6.0%	2.03 [1.45, 2.60]	2015
**Subtotal (95% CI)**			**36**			**36**	**6.0%**	**2.03 [1.45, 2.60]**	
Heterogeneity: Not applicable									
Test for overall effect: Z = 6.93 (P < 0.00001)									
**Total (95% CI)**			**740**			**738**	**100.0%**	**1.38 [1.04, 1.72]**	
Heterogeneity: Tau² = 0.42; Chi² = 129.01, df = 15 (P < 0.00001); I² = 88%									
Test for overall effect: Z = 7.97 (P < 0.00001)									
Test for subgroup differences: Chi² = 6.25, df = 2 (P = 0.04), I² = 68.0%									

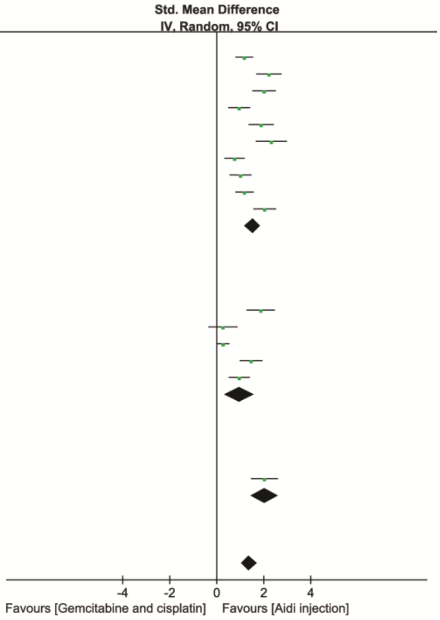

Figure S23 Subgroups analysis of $CD3^+$ $CD4^+$ T cells by cisplatin dosage

Study or Subgroup	Aidi injection Mean	SD	Total	Gemcitabine and cisplatin Mean	SD	Total	Weight	Std. Mean Difference IV, Random, 95% CI	Year
**20-30mg/m²**									
Zhang, H.2014	1.31	0.32	64	1.2	0.35	64	9.0%	0.33 [-0.02, 0.67]	2014
Zhao, S.2015	1.25	0.49	43	1.07	0.25	43	8.6%	0.46 [0.03, 0.89]	2015
Li, J.2016	1.31	0.26	47	1.23	0.24	47	8.7%	0.32 [-0.09, 0.72]	2016
Huang, W.2017	1.1	0.32	39	0.86	0.16	40	8.4%	0.94 [0.48, 1.41]	2017
Lv, W.2018	1.8	0.3	30	1.06	0.2	30	7.0%	2.86 [2.13, 3.60]	2018
Guo, X.2020	2.09	0.64	51	1.48	0.11	51	8.6%	1.32 [0.89, 1.75]	2020
**Subtotal (95% CI)**			**274**			**275**	**50.4%**	**0.99 [0.41, 1.57]**	
Heterogeneity: Tau² = 0.47; Chi² = 51.15, df = 5 (P < 0.00001); I² = 90%									
Test for overall effect: Z = 3.33 (P = 0.0009)									
**60-80mg/m²**									
Jiang,S.2011	1.56	0.3	32	0.78	0.4	30	7.5%	2.19 [1.55, 2.83]	2011
Li, X.2015	1.49	0.04	20	1.47	0.34	20	7.6%	0.08 [-0.54, 0.70]	2015
Wu, Y.2017	1.42	0.34	109	1.19	0.04	109	9.2%	0.95 [0.67, 1.23]	2017
Ma, M.2017	1.4	0.32	42	1.16	0.29	42	8.5%	0.78 [0.33, 1.22]	2017
Zhao,J.2019	0.74	0.12	43	0.64	0.29	43	8.6%	0.45 [0.02, 0.87]	2019
**Subtotal (95% CI)**			**246**			**244**	**41.6%**	**0.87 [0.35, 1.40]**	
Heterogeneity: Tau² = 0.29; Chi² = 26.81, df = 4 (P < 0.0001); I² = 85%									
Test for overall effect: Z = 3.27 (P = 0.001)									
**Others**									
Han, R.2015	1.14	0.23	36	0.81	0.15	36	8.0%	1.68 [1.14, 2.22]	2015
**Subtotal (95% CI)**			**36**			**36**	**8.0%**	**1.68 [1.14, 2.22]**	
Heterogeneity: Not applicable									
Test for overall effect: Z = 6.09 (P < 0.00001)									
**Total (95% CI)**			**556**			**555**	**100.0%**	**0.99 [0.62, 1.35]**	
Heterogeneity: Tau² = 0.35; Chi² = 88.06, df = 11 (P < 0.00001); I² = 88%									
Test for overall effect: Z = 5.31 (P < 0.00001)									
Test for subgroup differences: Chi² = 5.05, df = 2 (P = 0.08), I² = 60.4%									

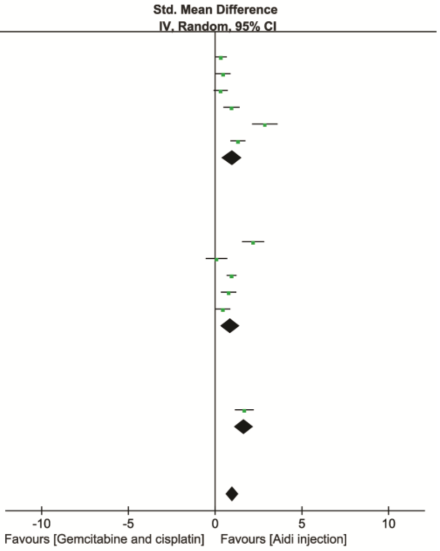

Figure S24 Subgroups analysis of $CD4^+/CD8^+$ T cells by cisplatin dosage
